# Synthesis, Antifungal Activity, and Molecular Simulation of Vanillin-Based 1,3,4-Oxadiazole-Thioether Derivatives as Potential Succinate Dehydrogenase Inhibitors

**DOI:** 10.3390/molecules31214004

**Published:** 2026-11-06

**Authors:** Wei-Wei Liu, Jing-Wen Wei, Zi-Ruo Ye, Ying-Hao Li, Yu-Cheng Cui, Gui-Shan Lin, Wen-Gui Duan, Xian-Li Ma

**Affiliations:** 1 School of Chemistry and Chemical Engineering, Guangxi University, Nanning 530004, China; 18334237503@163.com (W.-W.L.); bs15778813477@163.com (J.-W.W.); yzr1737389025@163.com (Z.-R.Y.); w1356722091@163.com (Y.-H.L.); cuiyc1109@126.com (Y.-C.C.); gslin@gxu.edu.cn (G.-S.L.); 2 Guangxi Key Laboratory for Pharmaceutical Molecular Discovery and Druggability Optimization, School of Pharmacy, Guilin Medical University, Guilin 541199, China

**Keywords:** vanillin, 1,3,4-oxadiazole-thioether, antifungal activity, structure–activity relationship, succinate dehydrogenase inhibitor, action mechanism

## Abstract

To explore potent natural product-based antifungal agents, thirty-two new vanillin-based 1,3,4-oxadiazole-thioether compounds were rationally designed and synthesized in this study. Their chemical structures were comprehensively confirmed by FT-IR, ^1^H NMR, ^13^C NMR, and HRMS. The in vitro antifungal activity of the target compounds against eight phytopathogenic fungi was preliminarily assayed using the agar dilution method, with the commercial fungicide boscalid, a succinate dehydrogenase inhibitor (SDHI), as the positive control. Results indicated that compounds **6n**, **6o**, and **6r** displayed significant inhibitory activity against *Physalospora piricola* (*P. p.*). Further EC_50_ tests revealed that these three compounds were all more effective than boscalid. Subsequently, to explore the relationship between structures of the target compounds and their antifungal activity against *P. p.*, a valid and reliable three-dimensional quantitative structure–activity relationship (3D-QSAR) model was established via the CoMFA method. Also, the density functional theory (DFT) calculations, including ESP and FMOs, were calculated to further elucidate the structure–activity relationship. To investigate the action mechanism between the target compounds and the potential biological target succinate dehydrogenase (SDH), the inhibitory activity of compounds **6n**, **6o**, and **6r** against SDH was assessed in this work. The results displayed that their SDH inhibitory activities were superior to that of the positive control, boscalid. Molecular docking simulations revealed that compound **6n** exhibited a binding mode similar to that of boscalid. Therefore, the target compound **6n** might exert excellent antifungal activity through inhibition of SDH. Compound **6n** deserves in-depth research as a lead compound for developing novel fungicides.

## 1. Introduction

Phytopathogenic fungi pose a severe threat to the safe production of crops, frequently leading to substantial yield reductions [1,2]. Chemical fungicides serve as critical tools for the management of fungal diseases. However, their long-term application tends to induce pathogen resistance [3], which not only diminishes disease control efficacy but also elevates pesticide application costs and introduces environmental risks associated with pesticide residues [4]. Therefore, the development of efficient, eco-friendly, and broad-spectrum novel antifungal agents to address the issue of pathogen resistance has become an urgent priority. Against this backdrop, natural products, characterized by their wide availability and abundant reserves, provide a highly promising research direction for the development of new antifungal agents [5,6].

Vanillin, chemically named as 4-hydroxy-3-methoxybenzaldehyde, is mainly found from natural Vanilla planifolia. It is widely used in the food, beverage, cosmetic, and pharmaceutical industries [7]. Vanillin exhibits diverse biological activities. including antibacterial, antifungal, anti-inflammatory, and antioxidant properties [8,9,10]. Owing to its reactive aldehyde group, phenolic hydroxyl group, and benzene ring, vanillin can be modified to synthesize various bioactive derivatives, such as compounds bearing thiazole, hydrazone, 1,2,3-triazole, and sulfonyl hydrazide moieties [11,12,13].

1,3,4-Oxadiazoles are a class of heterocyclic compounds containing two nitrogen atoms and one oxygen atom. Owing to its remarkable biological and pharmacological activities, such as antioxidant [14,15], antifungal [16,17,18], antitumor [19,20,21], and anti-inflammatory [22,23] effects, it is a core structural motif in natural products and pharmaceutical molecules. Numerous reports have confirmed that 1,3,4-oxadiazole possesses favorable inhibitory activity against a variety of plant pathogenic fungi [24,25]. Meanwhile, thioether compounds demonstrate a broad spectrum of biological activities, including antifungal [26,27], insecticidal [28,29], herbicidal [30], and antiviral properties [31]. Furthermore, studies have demonstrated that introduction of electron-donating sulfur atoms into molecules and the linkage of heterocyclic active center and substituent groups via thioether bonds facilitate the enhancement of receptor–ligand affinity, thereby effectively improving the biological activity of the compounds [30,32].

Succinate dehydrogenase inhibitors (SDHIs) have emerged as a prominent target in the research and development of novel fungicides, attributed to their exceptional antifungal activity and broad-spectrum efficacy. SDHIs impede the growth of pathogenic fungi by interfering with electron transport in the mitochondrial respiratory chain, which blocks mitochondrial energy production and ultimately results in fungal cell death [33]. Among various commercially available SDHI fungicides, structural motifs such as pyrazole, furan, thiophene, and thiazole are commonly incorporated; however, no SDHI fungicides bearing the inherent 1,3,4-oxadiazole core structure have been developed yet. As a five-membered heterocycle containing both oxygen and nitrogen atoms, 1,3,4-oxadiazole can act as a bioisostere for pyrazole, furan, thiophene, and thiazole. Furthermore, prior studies have confirmed that 1,3,4-oxadiazole derivatives exhibit both antifungal activity and succinate dehydrogenase inhibitory activity. In light of these findings, the present study incorporates the 1,3,4-oxadiazole-thioether structural unit to construct a novel antifungal molecular scaffold.

Based on the prior research of our research group on the modification of natural products [4,34,35,36], a series of novel vanillin-based 1,3,4-oxadiazole-thioether derivatives were designed and synthesized. The target compounds were synthesized via a multi-step synthetic route starting from vanillin, and their chemical structures were fully characterized by FT-IR, NMR, and HRMS. Simultaneously, the in vitro antifungal activities of all the target compounds against eight typical phytopathogenic fungi were screened. The structure–activity relationships were analyzed through three-dimensional quantitative structure–activity relationship (3D-QSAR) modeling and DFT calculations. Furthermore, the potential antifungal mechanism was explored via succinate dehydrogenase (SDH) inhibition assays and molecular docking studies.

## 2. Results

### 2.1. Synthesis and Characterization

**Scheme 1:**
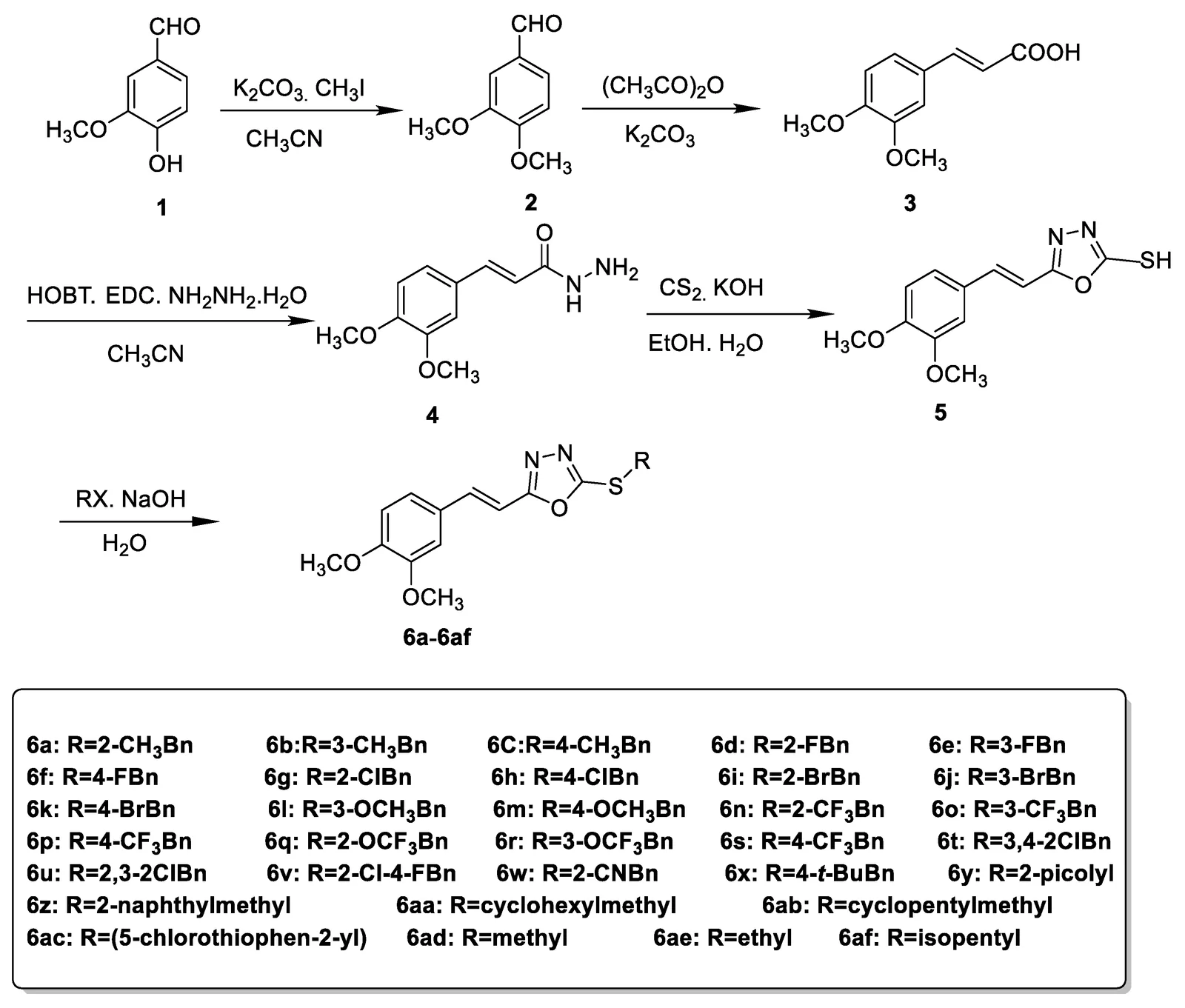
Synthetic route of vanillin-based 1,3,4 oxadiazole thioether derivatives **6a**–**6af**.

The synthetic route for target compounds **6a**–**6af** is illustrated in Scheme 1. Initially, natural vanillin (compound **1**) underwent O-alkylation of the phenolic hydroxyl group via the Williamson reaction to yield intermediate **2**. Subsequently, through the Perkin reaction, intermediate **2** was converted to the unsaturated carboxylic acid intermediate **3** with the phenylpropanoid C6-C3 active structural unit. Under catalysis of EDC/HOBt, intermediate **3** underwent N-acylation with hydrazine hydrate to give intermediate **4** bearing a hydrazide moiety. Intermediate **4** further reacted with carbon disulfide via a cyclization reaction to obtain the key intermediate **5**. Finally, nucleophilic substitution reactions of intermediate **5** with a series of halogenated substrates afforded thirty-two target derivatives **6a**–**6af**.

All the target compounds **6a**–**6af** were characterized by FT-IR, ^1^H NMR, ^13^C NMR, and HRMS. In FT-IR spectra, the absorption bands at 3089–3011 cm^−1^ were attributed to the stretching vibrations of C-H bonds in unsaturated aromatic rings or alkenes, while bands in the region 2984–2829 cm^−1^ were assigned to the saturated C-H stretching vibrations. The bands at 1655–1638 cm^−1^ were attributed to C=C and C=N stretching vibrations, while the bands at 1603–1471 cm^−1^ were attributed to aromatic-ring skeleton vibrations. The ranges at 1282–1253 cm^−1^ and 733–666 cm^−1^ were assigned to C-O-C and C-S-C stretching vibrations, respectively. In the ^1^H NMR spectra, *δ* 7.35–6.85 ppm, *δ* 7.41–6.82 ppm, and *δ* 5.00–3.24 ppm were assigned to characteristic protons of Ar-H, =C-H, and -S-CH_2_, respectively. In ^13^C NMR spectra, signals at *δ* 166.07–107.52 ppm were assigned to aromatic rings and unsaturated alkenes, whereas peaks at *δ* 56.01–19.22 ppm were attributed to saturated alkyl carbons. The [M+H]^+^ ion fragments of the target compounds **6a**–**6af** were observed in HRMS, which were consistent with their theoretical molecular weights. All the related characterization data and spectra can be found in the Supplementary Materials.

### 2.2. Antifungal Activity

The in vitro antifungal activities of compounds **6a**–**6af** against eight phytopathogenic fungi were evaluated at a concentration of 50 μg·mL^−1^, with the commercial fungicide boscalid used as the positive control. The tested pathogens included *Fusarium oxysporum f. sp. Cucumerinum (F. o.)*, *Cercospora arachidicola (C. a.)*, *Physalospora piricola (P. p.)*, *Alternaria solani (A. s.)*, *Gibberella zeae (G. z.)*, *Rhizoctonia solani (R. s.)*, *Bipolaris maydis (B. m.)*, and *Colleterichum orbicalare (C. o.)*. The experimental results are summarized in Table 1.

Bioactivity assay results demonstrated that the target compounds exerted varying degrees of antifungal activity against the eight tested fungal strains, with some compounds exhibiting potent inhibitory effects. The assays indicated that most target compounds exhibited favorable inhibition rates against *P. p*. In general, large electron-withdrawing substituents on the benzene ring afforded higher activity than that of electron-donating groups and small electron-withdrawing substituents. Compounds such as **6n**, **6o**, and **6r** showed better activity than that of **6a**, **6e**, and **6m**. Among aliphatic derivatives, straight-chain structures outperformed branched analogues, and compounds such as **6ad** and **6ae** showed better activity than that of **6x** and **6af**. Heterocyclic and naphthalene-ring derivatives exhibited relatively low activity. Meanwhile, for compounds bearing large electron-withdrawing substituents, substitution at the 2- and 3-positions produced the optimal antifungal activity. Collectively, compounds **6n**, **6r**, and **6o** displayed inhibition rates of 94.9%, 93.7%, and 86.1% against *P. p.*, respectively, which were significantly superior to that of the positive control boscalid.

In order to further evaluate the potential of the target compounds **6n**, **6r**, and **6o** as fungicide lead compounds, their EC_50_ values against *P. p*. were subsequently tested. As summarized in Table 2, compounds **6n**, **6r**, and **6o** showed significant inhibitory activity against *P. p.*, with EC_50_ values of 9.57 μg·mL^−1^, 11.80 μg·mL^−1^, and 15.64 μg·mL^−1^, respectively. All of these values were lower than that of the positive control boscalid with EC_50_ of 16.6 μg·mL^−1^. Thus, compounds **6n**, **6r**, and **6o** hold promising potential as lead candidates for the development of novel fungicides.

### 2.3. 3D-QSAR Analysis

**Figure 1:**
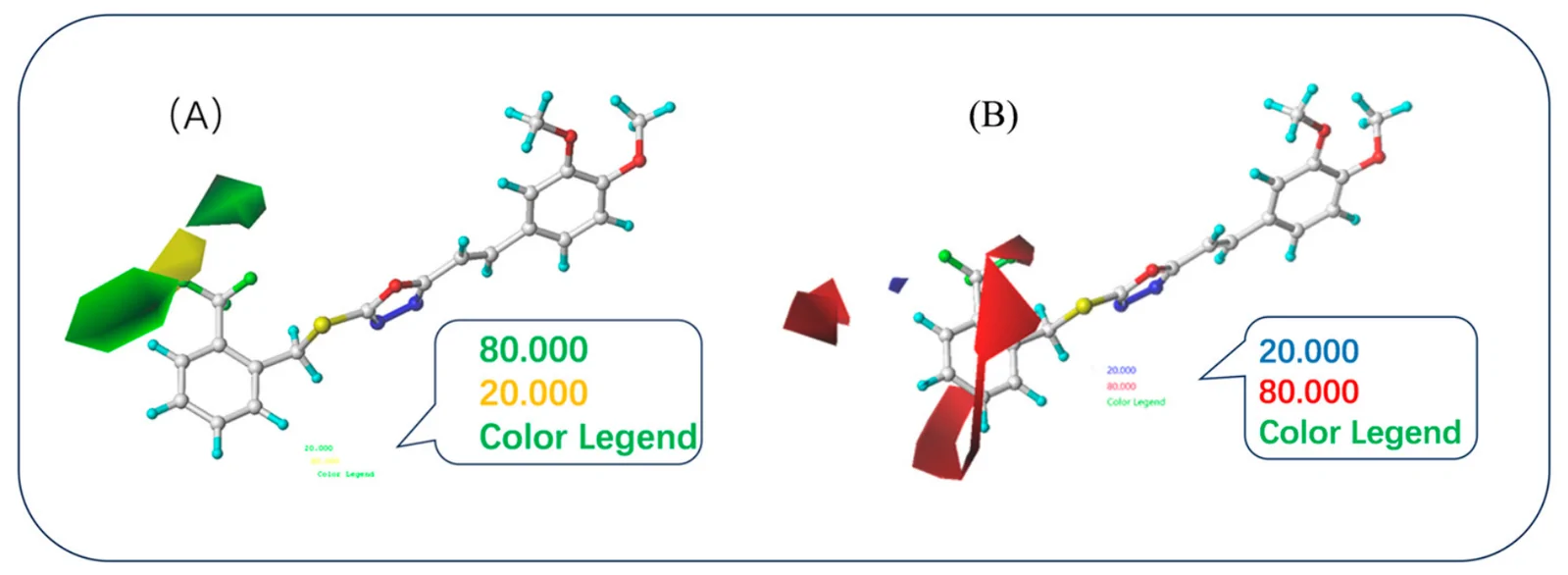
Contour maps of the steric (**A**) and electrostatic (**B**) contributions.

A 3D-QSAR study of the target compounds was performed using the CoMFA method to investigate the structure–activity relationship between molecular structures of the target compounds and their inhibitory activity against *P. p*. The statistical parameters of the constructed 3D-QSAR model were summarized in Table 3. The cross-validation coefficient (*q*^2^ = 0.528 > 0.5), correlation coefficient (*r*^2^ = 0.997 > 0.8), standard error (*S* = 0.056), and F value (*F* = 151.872 > 100) demonstrated that the model was reliable and valid. The contributions of steric and electrostatic effects of substituents were 55.8% and 44.2%, respectively, indicating that the steric effect of the R substituent played a dominant role in the antifungal activity against *P. p*. The three-dimensional contour maps are shown in Figure 1.

As shown in the contour map of Figure 1A, green blocks appeared near the ortho- and meta-positions of the benzene ring, indicating that introduction of bulky groups at these sites was beneficial for improving the biological activity of target compounds. For instance, compounds **6n** (R = 2-CF_3_Bn, 94.9%) and **6r** (R = 3-OCF_3_Bn, 93.7%) exhibited higher antifungal activities against P. p. than that of compounds **6a** (R = 2-CH_3_Bn, 29.1%), **6j** (R = 3-BrBn, 15.2%).

The electrostatic field contour map is presented in Figure 1B. Red blocks were observed around the *ortho-* and *meta*-positions of the benzene ring, which suggested that introduction of electron withdrawing substituents at these positions could enhance the biological activity of compounds. Consistently, compounds **6n** (R = 2-CF_3_Bn, 94.9%) and **6r** (R = 3-OCF_3_Bn, 93.7%) showed better inhibitory activities against *P. p*. than that of compounds **6a** (R = 2-CH_3_Bn, 29.1%) and **6b** (R = 3-CH_3_Bn, 35.4%).

**Figure 2:**
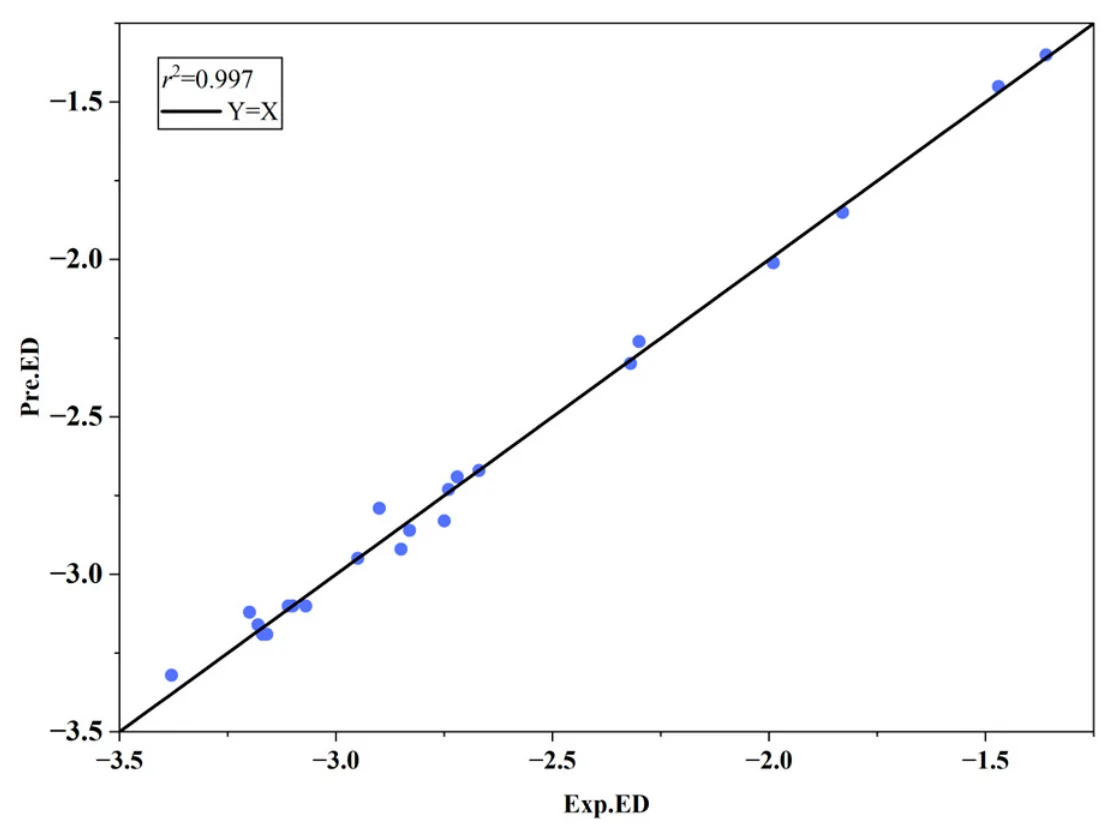
Predicted ED values of 3D QSAR model vs. experimental ED values.

Furthermore, a scatter plot of experimental and predicted activity values (experimental ED values versus predicted ED values) further verified the predictive ability of the 3D-QSAR model (Figure 2). All data points were distributed closely around the Y = X diagonal line, indicating that the model possessed good predictive performance.

### 2.4. DFT Analysis

**Figure 3:**
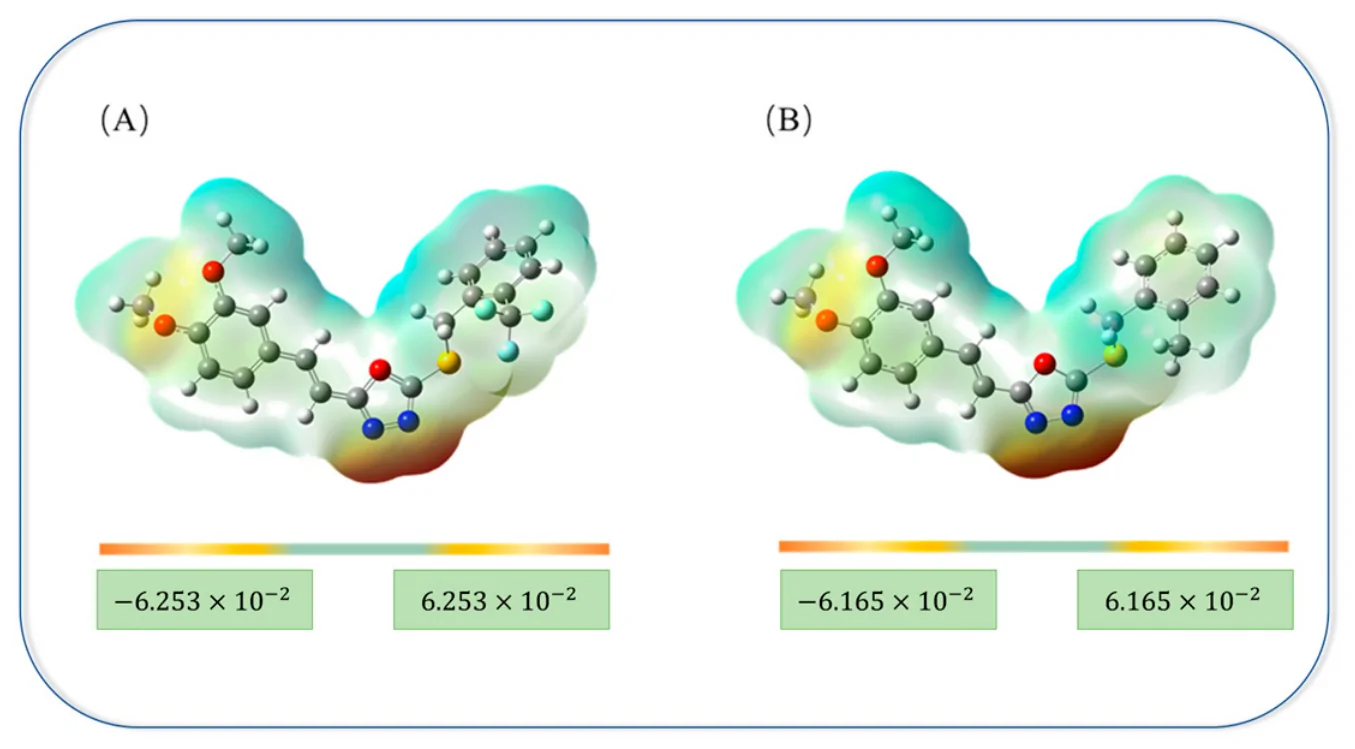
Electrostatic potential maps of the compounds **6n** (R = 2-CF_3_Bn, (**A**)) and **6a** (R = 2-CH_3_Bn, (**B**)).

To further elucidate the structure–activity relationship of the target compounds, compounds **6a** and **6n**, which bore electron-donating and strong electron-withdrawing substituents respectively and exhibited markedly different antifungal activities, were selected for electrostatic potential (ESP) and frontier molecular orbital (FMO) analyses in this study. In the ESP map, blue regions correspond to positive electrostatic potential, whereas red regions denote negative electrostatic potential. Notably, regions with negative electrostatic potential possess enhanced electrophilic attraction capacity and act as critical sites mediating the biological activity of the compounds. As shown in Figure 3, the minimum electrostatic potential of compound **6n** (R = 2-CF_3_Bn, (A)) was −6.253 × 10^−2^, which was lower than that of compound **6a** (R = 2-CH_3_Bn, (B)) (−6.165 × 10^−2^), indicating that **6n** was more prone to electrostatic attraction with positively charged amino acid residues on SDH, thereby enhancing the stability of the compound-target complex. Furthermore, the negative electrostatic potential regions of the compounds were predominantly localized on the oxygen atom of vanillin and the oxygen atom within the 1,3,4-oxadiazole heterocycle, suggesting that these negatively charged regions might play a crucial role in mediating the binding interaction between the compounds and the target protein.

**Figure 4:**
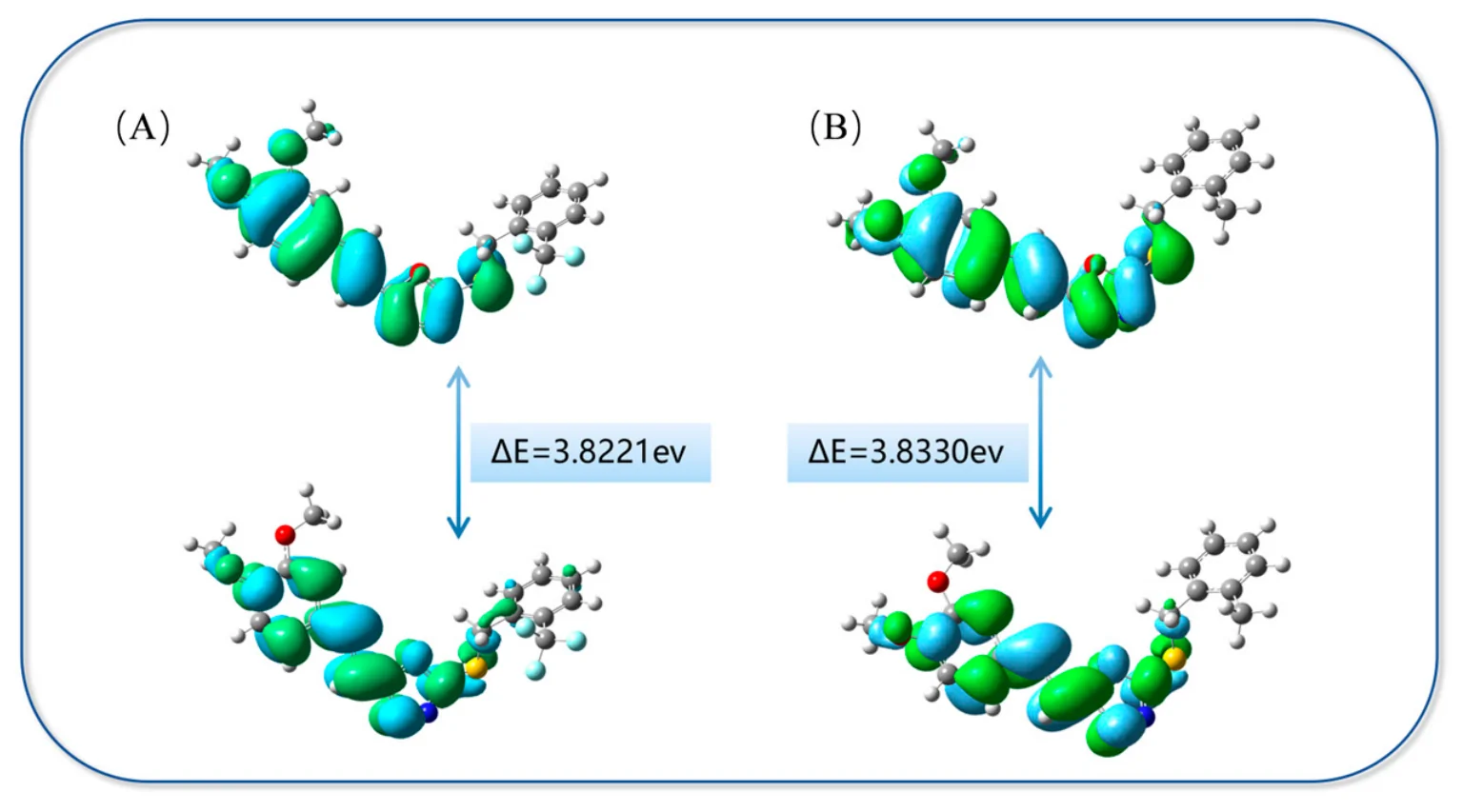
HOMO and LUMO of compounds **6n** (R = 2-CF_3_Bn, (**A**)) and **6a** (R = 2-CH_3_Bn, (**B**)).

According to FMO theory, the HOMO functions as an electron donor, while the LUMO serves as an electron acceptor. Together, they collectively determine the electron transfer capacity and interaction potential between a molecule and its target. As shown in Figure 4, the electron clouds of the HOMO and LUMO of both compounds were predominantly localized on the vanillin moiety and the 1,3,4-oxadiazole ring. This observation indicated that these two structural fragments acted as the primary carriers of the molecular frontier orbitals, serving as key reactive sites for electron donation and acceptance, and thus played a dominant role in mediating the antifungal activity of the compounds. Furthermore, the HOMO-LUMO energy gap of compound **6n** (3.8221 eV) was smaller than that of compound **6a** (3.8330 eV). implying that **6n** possessed higher chemical reactivity. A smaller energy gap promotes electronic transitions and charge transfer, which in turn results in higher polarizability and reactivity, as well as stronger interactions with the target [37]. The calculated ESP and FMO results were consistent with the trend in antifungal activity. The stronger electrostatic interaction and more favorable charge-transfer characteristics jointly accounted for the superior antifungal activity of **6n** over **6a**, providing electronic-structure insights into the structure–activity relationship of this series of SDHIs [38,39].

### 2.5. SDH-Inhibition Activity and Molecular Docking

To evaluate the potential of representative compounds **6o**, **6n**, and **6r** as SDHI fungicides, their inhibitory activities against SDH were determined. As presented in Table 4, the IC_50_ values of compounds **6o**, **6n**, and **6r** were 1.29, 2.09, and 2.34 μM, respectively, which were superior to that of the positive control boscalid with IC_50_ of 7.02 μM. A strong correlation was observed between the SDH inhibitory activity and the remarkable antifungal activity of the compounds, indicating that these compounds might exert their antifungal effects via SDH inhibition.

**Figure 5:**
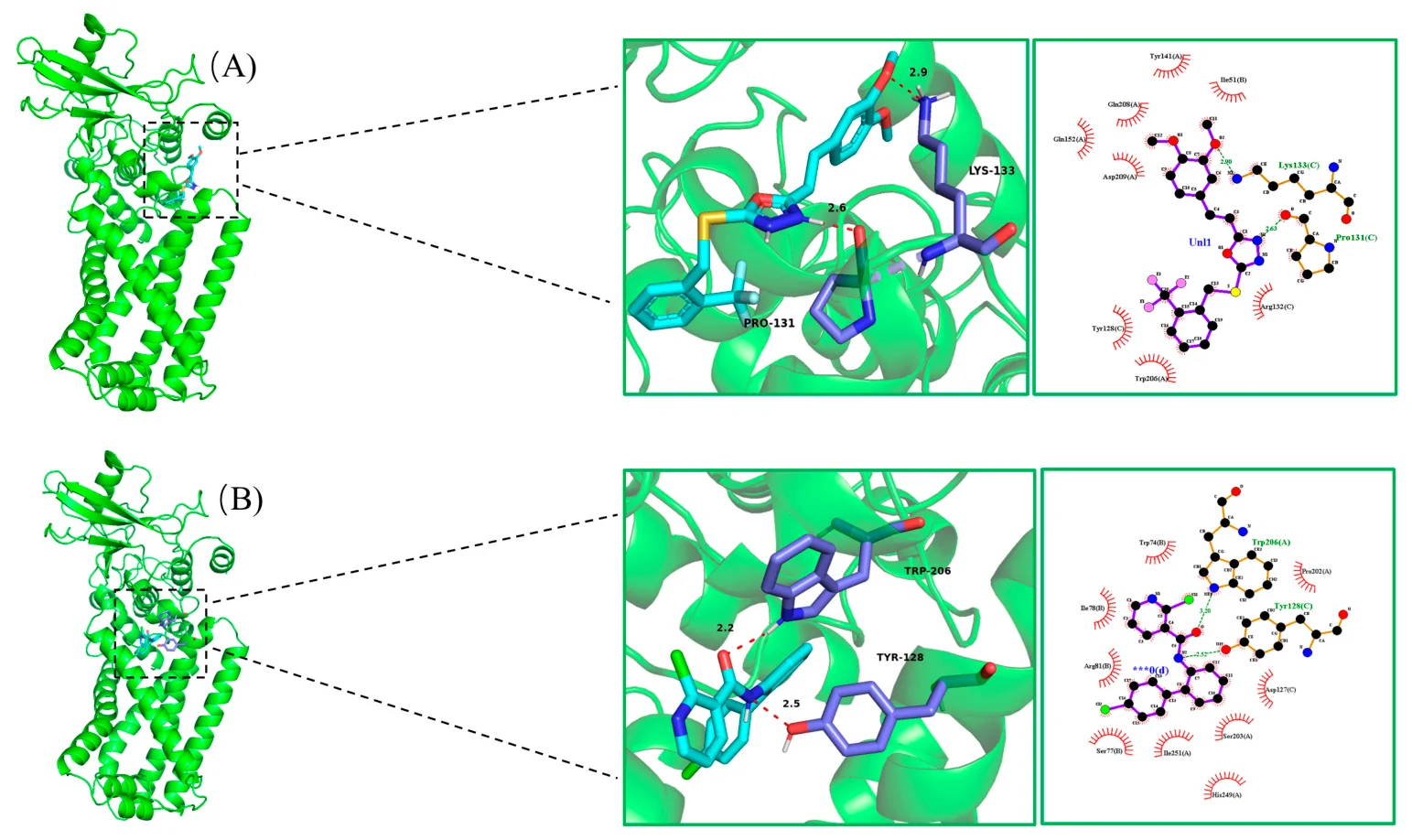
Interaction of compound 6n (**A**) and boscalid (**B**) at the active site of SDH.

Furthermore, molecular docking simulations were performed to explore the interaction modes of compound **6n** and the positive control boscalid with the SDH receptor protein, respectively. As illustrated in Figure 5, both compound **6n** and boscalid could embed into the active pocket of the SDH protein and formed multiple interactions with key amino acid residues.

At the active site of SDH, compound **6n** primarily interacted with partial amino acid residues of SDH via van der Waals forces and two hydrogen bond interactions. Notably, the binding energy of compound **6n** to SDH (−7.02 kcal/mol) was lower than that of the positive control boscalid (−6.91 kcal/mol), indicating that compound **6n** exhibited stronger binding affinity to SDH and formed a more stable complex with the protein. Furthermore, the nitrogen and oxygen atoms in compound **6n** could form two hydrogen bonds with key amino acid residues C-Pro131 and C-Lys133. Meanwhile, the core skeleton of compound **6n** was positioned in proximity to amino acid residues including A-Trp206, A-Asp209, A-Gln152, A-Gln208, C-Tyr128, and C-Arg132, thereby generating strong hydrophobic interactions. Notably, the number of interacting residues for compound **6n** was greater than that of the positive control boscalid.

## 3. Materials and Methods

### 3.1. Materials

Vanillin (GC purity ≥ 99.7%), the positive control boscalid (purity 98%+) and multi-substituted halides were purchased from Shanghai Titan Scientific Co., Ltd. (Shanghai, China). The SDH enzyme-linked immunosorbent assay (ELISA) test kit was purchased from Jiangsu Meimian Industrial Co., Ltd. (Jiangyin, China). Other chemical reagents and solvents involved in the experiments were all sourced from Lantian Nanning Experimental Instrument Co., Ltd. (Nanning, China) and used as received.

### 3.2. Synthesis of Intermediate (***2***)

At room temperature, vanillin (1.52 g, 10 mmol) was dissolved in 30 mL of acetonitrile. Anhydrous K_2_CO_3_ (1.38 g, 10 mmol) and iodomethane (2.13 g, 15 mmol) were added to the solution with stirring. The resulting mixture was heated under reflux for 3 h until the reaction was complete. After cooling to room temperature, the mixture was filtered, and the filtrate was diluted with 50 mL of water and extracted with ethyl acetate. The combined organic layers were dried over anhydrous Na_2_SO_4_. The solvent was removed under reduced pressure to prepare intermediate **2** as a white solid in 89% yield.

### 3.3. Synthesis of Intermediate (***3***)

To a 100 mL flask, we added intermediate **2** (1.66 g, 10 mmol), anhydrous K_2_CO_3_ (2.21 g, 16 mmol), and acetic anhydride (3.06 g, 30 mmol). The mixture was heated to 170 °C and stirred under reflux until completion as monitored by TLC. After cooling to room temperature, a small amount of water was added to submerge the solidified mass, which was broken up with a glass rod. The pH was adjusted to 10~11 with 10% aqueous NaOH. The aqueous mixture was extracted with ethyl acetate (3 × 30 mL). The aqueous layer was collected and acidified to pH 1–2 with 2 mol/L HCl. The resulting precipitate was collected by filtration and recrystallized from ethyl acetate to obtain intermediate **3** in 44.1% yield.

### 3.4. Synthesis of Intermediate (***4***)

Intermediate **3** (3.56 g, 20 mmol), HOBT (3.24 g, 24 mmol), and EDC (4.60 g, 24 mmol) were dissolved in 50 mL of acetonitrile, and the mixture was stirred for 2 h. An 80% hydrazine hydrate solution (2.00 g, 40.00 mmol) was added dropwise to the system under ice bath over 40 min. Upon completion of the dropwise addition, the reaction was immediately quenched with saturated aqueous NaHCO_3_ to prevent side reactions. The mixture was extracted with ethyl acetate (3 × 30 mL). The combined organic layers were dried over anhydrous MgSO_4_, and the solvent was removed under reduced pressure. The crude product was purified by recrystallization from ethanol to give an off-white solid in 80.2% yield.

### 3.5. Synthesis of Intermediate (***5***)

In a 100 mL round-bottom flask, intermediate **4** (3.88 g, 20 mmol) and potassium hydroxide (1.35 g, 24 mmol) were dissolved in 50 mL of absolute ethanol. Subsequently, 2 mL of carbon disulfide was slowly added dropwise at room temperature. The resulting mixture was heated under reflux with continuous stirring for 6–8 h. After the reaction solution was cooled to room temperature, its pH was adjusted to 2–3 with 10% dilute hydrochloric acid, and a white solid precipitate was formed. The solid was collected by suction filtration, washed with deionized water until the filtrate was neutral, and recrystallized from ethanol to prepare off-white intermediate **4** in 77.9% yield.

### 3.6. Synthesis of Vanillin-Based 1,3,4-Oxadiazole-Thioether Derivatives ***6a**–**6af***

The general procedure for compounds **6a**–**6af** is given below using **6a** as the representative. Intermediate **5** (0.39 g, 1.5 mmol) and NaOH (0.067 g, 1.65 mmol) were sequentially added to a two-necked flask containing 20 mL of water. The mixture was stirred at room temperature to form a clear yellow solution, followed by the slow dropwise addition of 2-methylbenzyl chloride (0.25 g, 1.8 mmol). The reaction progress was monitored by TLC. After completion of the reaction, the mixture was subjected to suction filtration. The obtained solid was recrystallized from ethyl acetate and petroleum ether to afford compound **6a**. All other compounds **6a**–**6af** were prepared according to the same procedure.

Compound (**6a**): *(E)-2-(3,4-dimethoxystyryl)-5-((2-methylbenzyl)thio)-1,3,4-oxadiazole*. White solid; yield, 85.2%; m.p. 103.5–05.7 °C; IR (KBr, cm^−1^): 3011.32 (Ar-H, C=C-H), 2970.06, 2941.17, 2838.00 (C-H), 1633.04 (C=N, C=C), 1600.03, 1583.52, 1509.24, 1476.23 (Ar-C=C), 1253.39 (C-O-C) 727.25 (C-S-C). ^1^H NMR (600 MHz, CDCl_3_) *δ* 7.41–7.35 (m, 2H,Ar-H, C_9_-H), 7.24–7.15 (m, 3H, Ar-H), 7.09 (dd, *J* = 8.3, 2.0 Hz, 1H,Ar-H), 7.05 (d, *J* = 2.0 Hz, 1H, Ar-H), 6.89–6.82 (m, 2H, Ar-H, C_10_-H), 4.53 (s, 2H, C_13_-H), 3.92 (s, 3H, C_7_-H), 3.91 (s, 3H, C_8_-H), 2.44 (s, 3H, C_20_-H). ^13^C NMR (151 MHz, CDCl_3_) *δ* 165.69, 163.27, 150.87, 149.35, 138.55, 137.15, 133.00, 130.78, 130.31, 128.58, 127.77, 126.44, 121.86, 111.18, 109.09, 107.42, 56.01, 55.93, 35.16, 19.22. HRMS (APCI, *m*/*z*): calcd. for C_20_H_20_N_2_O_3_S^+^ ([M+H]^+^) 369.1267, found 369.1262.

Compound (**6b**): *(E)-2-(3,4-dimethoxystyryl)-5-((3-methylbenzyl)thio)-1,3,4-oxadiazole.* White solid; yield, 90.2%; m.p. 95.1–99.4 °C; IR (KBr, cm^−1^): 3055.91 (Ar-H, C=C-H), 2954.24, 2935.56, 2838.04 (C-H), 1655.33, 1638.73 (C=N, C=C),1603.45, 1586.86, 1514.24, 1474.81 (Ar-C=C), 1277.69 (C-O-C), 736.14 (C-S-C). ^1^H NMR (600 MHz, CDCl_3_) *δ* 7.37 (d, *J* = 16.3 Hz, 1H, C_9_-H), 7.27–7.20 (m, 3H,Ar-H), 7.11 (d, *J* = 3.7 Hz, 1H, Ar-H), 7.08 (dd, *J* = 8.3, 2.0 Hz, 1H, Ar-H), 7.04 (d, *J* = 2.0 Hz, 1H, Ar-H), 6.87 (d, *J* = 8.3 Hz, 1H, Ar-H), 6.85 (d, *J* = 16.3 Hz, 1H, C_10_-H), 4.47 (s, 2H, C_13_-H), 3.92 (s, 3H, C_7_-H), 3.91 (s, 3H, C_8_-H), 2.34 (s, 3H, C_20_-H). ^13^C NMR (151 MHz, CDCl_3_) *δ* 165.68, 163.25, 150.87, 149.36, 138.58, 138.51, 135.37, 129.83, 128.91, 128.73, 127.78, 126.18, 121.84, 111.20, 109.11, 107.43, 56.01, 55.93, 36.84, 21.35. HRMS (APCI, *m*/*z*): calcd. for C_20_H_20_N_2_O_3_S^+^ ([M+H]^+^) 369.1267, found 369.1263.

Compound (**6c**)*: (E)-2-(3,4-dimethoxystyryl)-5-((4-methylbenzyl)thio)-1,3,4-oxadiazole*. White solid; yield, 88.6%; m.p.88.4–110.8 °C; IR (KBr, cm^−1^): 3077.82 (Ar-H, C=C-H), 2997.36, 2949.91, 2824.07 (C-H), 1639.23 (C=N, C=C), 1597.97, 1509.24, 1474.17 (Ar-C=C), 1278.16 (C-O-C), 707.62 (C-S-C). ^1^H NMR (600 MHz, CDCl_3_) *δ* 7.36 (d, *J* = 16.4 Hz, 1H, C_9_-H), 7.32 (d, *J* = 7.9 Hz, 2H, Ar-H), 7.14 (d, *J* = 7.8 Hz, 2H, Ar-H), 7.08 (dd, *J* = 8.3, 2.1 Hz, 1H Ar-H), 7.04 (d, *J* = 2.1 Hz, 1H, Ar-H), 6.87 (d, *J* = 8.3 Hz, 1H, Ar-H), 6.84 (d, *J* = 16.3 Hz, 1H, C_10_-H), 4.46 (s, 2H, C_13_-H), 3.92 (s, 3H, C_7_-H), 3.90 (s, 3H, C_8_-H), 2.32 (s, 3H, C_20_-H). ^13^C NMR (151 MHz, CDCl_3_) *δ* 165.66, 163.28, 150.85, 149.34, 138.52, 137.97, 132.42, 129.51, 129.07, 127.77, 121.85, 111.18, 109.09, 107.41, 56.01, 55.93, 36.65, 21.18. HRMS (APCI, *m*/*z*): calcd. for C_20_H_20_N_2_O_3_S^+^ ([M+H]^+^) 369.1267, found 369.1262.

Compound (**6d**)*: (E)-2-(3,4-dimethoxystyryl)-5-((2-fluorobenzyl)thio)-1,3,4-oxadiazole*. White solid; yield, 78.2%; m.p. 91.5–93.4 °C; IR (KBr, cm^−1^): 3048.46 (Ar-H, C=C-H), 2976.25, 2947.36, 2835.94 (C-H), 1635.10 (C=N, C=C), 1600.03, 1585.58, 1509.24, 1478.29 (Ar-C=C), 1276.09 (C-O-C), 677.74 (C-S-C). ^1^H NMR (600 MHz, CDCl_3_) *δ* 7.51 (td, *J* = 7.6, 1.8 Hz, 1H,Ar-H), 7.37 (d, *J* = 16.3 Hz, 1H, C9-H), 7.30–7.27 (m, 1H, Ar-H), 7.12–7.03 (m, 4H, Ar-H), 6.87 (d, *J* = 8.3 Hz, 1H, Ar-H), 6.83 (d, *J* = 16.3 Hz, 1H, C_10-_H), 4.52 (s, 2H, C_13_-H), 3.91 (s, 3H, C_7_-H), 3.90 (s, 3H, C_8_-H). ^13^C NMR (151 MHz, CDCl3) *δ* 165.86, 161.81, 161.79, 160.15, 150.88, 149.34, 138.65, 131.40 (d, *J* = 3.3 Hz), 130.10 (d, *J* = 8.1 Hz), 127.73, 124.38 (d, *J* = 3.8 Hz), 123.14 (d, *J* = 14.5 Hz), 121.88, 115.64 (d, *J* = 21.0 Hz), 111.17, 109.08, 107.34, 56.01, 55.93, 30.06 (d, *J* = 3.4 Hz). HRMS (APCI, *m*/*z*): calcd. for C19H17FN2O3S^+^ ([M+H]^+^) 373.1017, found 373.1011.

Compound (**6e**)*: (E)-2-(3,4-dimethoxystyryl)-5-((3-fluorobenzyl)thio)-1,3,4-oxadiazole*. White solid; yield, 89.4%; m.p. 94.8–98.1 °C; IR (KBr, cm^−1^): 3056.71 (Ar-H, C=C-H), 2978.31, 2943.23, 2842.13 (C-H), 1653.68,1637.17 (C=N, C=C), 1597.97, 1581.46, 1517.50, 1474.17 (Ar-C=C), 1276.09 (C-O-C), 708.69 (C-S-C). ^1^H NMR (600 MHz, CDCl_3_) *δ* 7.36 (d, *J* = 16.3 Hz, 1H, C_9_-H), 7.29 (td, *J* = 8.0, 5.8 Hz, 1H, Ar-H), 7.22 (d, *J* = 7.8 Hz, 1H, Ar-H), 7.17 (dt, *J* = 9.5, 2.1 Hz, 1H, Ar-H), 7.08 (dd, *J* = 8.3, 2.0 Hz, 1H, Ar-H), 7.04 (d, *J* = 2.0 Hz, 1H, Ar-H), 6.99 (td, *J* = 8.4, 2.6 Hz, 1H, Ar-H), 6.87 (d, *J* = 8.3 Hz, 1H, Ar-H), 6.83 (d, *J* = 16.3 Hz, 1H, C_10_-H), 4.47 (s, 2H, C_13_-H), 3.92 (s, 3H, C_7_-H), 3.90 (s, 3H, C_8_-H). 13C NMR (151 MHz, CDCl_3_) *δ* 165.85, 162.73, 150.90, 149.35, 138.71, 130.37, 130.31, 127.70, 124.83, 124.81, 121.87, 116.18, 116.03, 115.20, 115.06, 111.18, 109.10, 107.30, 56.01, 55.92, 36.16, 36.14. HRMS (APCI, *m*/*z*): calcd. for C_19_H_17_FN_2_O_3_S^+^ ([M+H]^+^) 373.1017, found 373.1011.

Compound (**6f**)*: (E)-2-(3,4-dimethoxystyryl)-5-((4-fluorobenzyl)thio)-1,3,4-oxadiazole*. White solid; yield, 74.1%; m.p. 101.2–103.5 °C; IR (KBr, cm^−1^): 3089.73(Ar-H, C=C-H), 2965.93, 2934.98, 2838.00(C-H), 1635.10 (C=N, C=C), 1602.09, 1581.46, 1513.37, 1472.10 (Ar-C=C), 1278.15 (C-O-C), 733.44 (C-S-C). ^1^H NMR (600 MHz, CDCl_3_) *δ* 7.43 (d, *J* = 5.3 Hz, 1H,Ar-H), 7.41 (d, *J* = 5.3 Hz, 1H,Ar-H), 7.36 (d, *J* = 16.4 Hz, 1H, C9-H), 7.08 (dd, *J* = 8.3, 2.0 Hz, 1H,Ar-H), 7.04 (d, *J* = 2.0 Hz, 1H,Ar-H), 7.02 (t, *J* = 8.6 Hz, 2H,Ar-H), 6.87 (d, *J* = 8.3 Hz, 1H,Ar-H), 6.84 (d, *J* = 16.3 Hz, 1H, C_10_-H), 4.46 (s, 2H, C_13_-H), 3.92 (s, 3H, C_7_-H), 3.91 (s, 3H, C_8_-H). ^13^C NMR (151 MHz, CDCl_3_) *δ* 165.80, 162.90, 162.48 (d, *J* = 247.5 Hz), 150.90, 149.35, 138.67, 131.49 (d, *J* = 3.3 Hz), 130.91 (d, *J* = 8.3 Hz), 127.71, 121.87, 115.75 (d, *J* = 21.6 Hz), 111.18, 109.09, 107.32, 56.01, 55.93, 36.02. HRMS (APCI, *m*/*z*): calcd. for C_19_H_17_FN_2_O_3_S^+^ ([M+H]^+^) 373.1017, found 373.1012.

Compound (**6g**)*: (E)-2-((2-chlorobenzyl)thio)-5-(3,4-dimethoxystyryl)-1,3,4-oxadiazole*. White solid; yield, 88.6%; m.p. 94.5–97.6 °C; IR (KBr, cm^−1^): 3055.13(Ar-H, C=C-H), 2968.48, 2941.66, 2836.45 (C-H), 1635.76 (C=N, C=C), 1600.69, 1586.25, 1514.04, 1478.97 (Ar-C=C), 1280.92 (C-O-C), 732.15 (C-S-C). ^1^H NMR (600 MHz, CDCl_3_) *δ* 7.59 (dd, *J* = 7.2, 2.1 Hz, 1H,Ar-H), 7.41–7.38 (m, 1H, Ar-H), 7.36 (d, *J* = 16.3 Hz, 1H, C_9_-H), 7.23 (dtd, *J* = 15.1, 7.5, 1.8 Hz, 2H, Ar-H), 7.07 (dd, *J* = 8.3, 2.2 Hz, 1H, Ar-H), 7.04 (d, *J* = 2.0 Hz, 1H, Ar-H), 6.86 (d, *J* = 7.7 Hz, 1H, Ar-H), 6.83 (d, *J* = 16.4 Hz, 1H, C_10_-H), 4.59 (s, 2H, C_13_-H), 3.91 (s, 3H, C_7_-H), 3.90 (s, 3H, C_8_-H). ^13^C NMR (151 MHz, CDCl_3_) *δ* 165.85, 163.07, 150.87, 149.34, 138.62, 134.36, 133.71, 131.50, 129.79, 129.64, 127.73, 127.13, 121.88, 111.17, 109.08, 107.34, 56.01, 55.93, 34.56. HRMS (APCI, *m*/*z*): calcd. for C_19_H_17_ClN_2_O_3_S^+^ ([M+H]^+^) 389.0721, found 389.0715.

Compound (**6h**)*: (E)-2-((4-chlorobenzyl)thio)-5-(3,4-dimethoxystyryl)-1,3,4-oxadiazole*. White solid; yield, 81.6%; m.p. 108.7–112.8 °C; IR (KBr, cm^−1^): 3067.51 (Ar-H, C=C-H), 2968.48, 2949.91, 2830.26 (C-H), 1641.95 (C=N, C=C), 1604.81, 1588.31, 1514.04, 1476.91 (Ar-C=C), 1268.54 (C-O-C), 728.02 (C-S-C). ^1^H NMR (600 MHz, CDCl_3_) *δ* 7.38 (d, *J* = 8.4 Hz, 2H,Ar-H), 7.35 (d, *J* = 16.4 Hz, 1H, C_9_-H), 7.32–7.28 (m, 2H, Ar-H), 7.08 (dd, *J* = 8.3, 2.0 Hz, 1H, Ar-H), 7.04 (d, *J* = 2.0 Hz, 1H, Ar-H), 6.87 (d, *J* = 8.3 Hz, 1H, Ar-H), 6.83 (d, *J* = 16.3 Hz, 1H, C_10_-H), 4.44 (s, 2H, C_13_-H), 3.92 (s, 3H, C_7_-H), 3.90 (s, 3H, C_8_-H). ^13^C NMR (151 MHz, CDCl_3_) *δ* 165.84, 162.75, 150.90, 149.34, 138.72, 134.30, 134.01, 130.53, 128.96, 127.68, 121.89, 111.17, 109.10, 107.27, 56.01, 55.93, 36.01. HRMS (APCI, *m*/*z*): calcd. for C_19_H_17_ClN_2_O_3_S^+^ ([M+H]^+^) 389.0721, found 389.0716.

Compound (**6i**)*: (E)-2-((2-bromobenzyl)thio)-5-(3,4-dimethoxystyryl)-1,3,4-oxadiazole*. White solid; yield, 83.2%; m.p. 96.8–100.1 °C; IR (KBr, cm^−1^): 3057.19 (Ar-H, C=C-H), 2966.42, 2945.79, 2836.45 (C-H), 1633.70 (C=N, C=C), 1598.62, 1582.12, 1518.17, 1483.09 (Ar-C=C), 1276.79 (C-O-C), 674.38 (C-S-C). ^1^H NMR (600 MHz, CDCl_3_) *δ* 7.61 (dd, *J* = 7.6, 1.7 Hz, 1H, Ar-H), 7.57 (dd, *J* = 8.0, 1.3 Hz, 1H, Ar-H), 7.36 (d, *J* = 16.3 Hz, 1H, C_9_-H), 7.28–7.24 (m, 1H, Ar-H), 7.15 (td, *J* = 7.7, 1.7 Hz, 1H, Ar-H), 7.07 (dd, *J* = 8.3, 2.0 Hz, 1H, Ar-H), 7.04 (d, *J* = 2.0 Hz, 1H, Ar-H), 6.86 (d, *J* = 8.3 Hz, 1H, Ar-H), 6.83 (d, *J* = 16.3 Hz, 1H, C_10_-H), 4.60 (s, 2H, C_13_-H), 3.91 (s, 3H, C_7_-H), 3.90 (s, 3H, C_8_-H). ^13^C NMR (151 MHz, CDCl_3_) *δ* 165.84, 163.00, 150.87, 149.34, 138.61, 135.42, 133.09, 131.55, 129.79, 127.76, 127.73, 124.73, 121.86, 111.18, 109.11, 107.35, 56.00, 55.93, 37.21. HRMS (APCI, *m*/*z*): calcd. for C_19_H_17_BrN_2_O_3_S^+^ ([M+H]^+^) 433.0216, found 433.0210.

Compound (**6j**)*: (E)-2-((3-bromobenzyl)thio)-5-(3,4-dimethoxystyryl)-1,3,4-oxadiazole*. White solid; yield, 78.9%; m.p. 97.8–101.3 °C; IR (KBr, cm^−1^): 3062.96 (Ar-H, C=C-H), 2964.87, 2935.65, 2835.48(C-H), 1645.91 (C=N, C=C), 1597.91, 1585.39, 1510.26, 1474.78 (Ar-C=C), 1280.70 (C-O-C), 736.00 (C-S-C). ^1^H NMR (600 MHz, CDCl_3_) *δ* 7.60 (t, *J* = 1.9 Hz, 1H,Ar-H), 7.42 (d, *J* = 7.9 Hz, 1H, Ar-H), 7.40–7.33 (m, 2H, Ar-H, C_9_-H), 7.20 (t, *J* = 7.9 Hz, 1H, Ar-H), 7.08 (dd, *J* = 8.4, 2.0 Hz, 1H, Ar-H), 7.04 (d, *J* = 2.0 Hz, 1H, Ar-H), 6.87 (d, *J* = 8.3 Hz, 1H, Ar-H), 6.83 (d, *J* = 16.3 Hz, 1H, C_10_-H), 4.43 (s, 2H, C_13_-H), 3.92 (s, 3H, C_7_-H), 3.90 (s, 3H, C_8_-H). ^13^C NMR (151 MHz, CDCl_3_) *δ* 165.86, 162.64, 150.90, 149.35, 138.73, 138.03, 132.09, 131.24, 130.34, 127.82, 127.70, 122.67, 121.89, 111.18, 109.12, 107.29, 56.01, 55.94, 35.99. HRMS (APCI, *m*/*z*): calcd. for C_19_H_17_BrN_2_O_3_S^+^ ([M+H]^+^) 433.0216, found 433.0212.

Compound (**6k**)*: (E)-2-((4-bromobenzyl)thio)-5-(3,4-dimethoxystyryl)-1,3,4-oxadiazole*. White solid; yield, 83.7%; m.p. 101.7–104.8 °C; IR (KBr, cm^−1^): 3014.96 (Ar-H, C=C-H), 2956.52, 2929.39, 2837.57(C-H), 1633.39 (C=N, C=C), 1600.00, 1583.30, 1518.61, 1481.04 (Ar-C=C), 1274.43 (C-O-C), 723.48 (C-S-C). ^1^H NMR (600 MHz, CDCl_3_) *δ* 7.46–7.43 (m, 2H,Ar-H), 7.35 (d, *J* = 16.3 Hz, 1H, C_9_-H), 7.32 (d, *J* = 2.0 Hz, 1H, Ar-H), 7.31 (d, *J* = 2.0 Hz, 1H, Ar-H), 7.07 (dd, *J* = 8.3, 2.0 Hz, 1H, Ar-H), 7.03 (d, *J* = 2.0 Hz, 1H, Ar-H), 6.86 (d, *J* = 8.3 Hz, 1H, Ar-H), 6.82 (d, *J* = 16.3 Hz, 1H, C_10_-H), 4.42 (s, 2H, C_13_-H), 3.91 (s, 3H, C_7_-H), 3.90 (s, 3H, C_8_-H). ^13^C NMR (151 MHz, CDCl_3_) *δ* 165.85, 162.72, 150.91, 149.35, 138.73, 134.83, 131.93, 130.85, 127.69, 122.14, 121.90, 111.18, 109.10, 107.27, 56.01, 55.94, 36.06. HRMS (APCI, *m*/*z*): calcd. for C_19_H_17_BrN_2_O_3_S^+^ ([M+H]^+^) 433.0216, found 433.0211.

Compound (**6l**)*: (E)-2-(3,4-dimethoxystyryl)-5-((3-methoxybenzyl)thio)-1,3,4-oxadiazole*. White solid; yield, 83.1%; m.p. 105.1–109.2 °C; IR (KBr, cm^−1^): 3058.78 (Ar-H, C=C-H), 2944.00, 2921.04, 2833.39 (C-H), 1652.17, 1637.57 (C=N, C=C), 1600.00, 1585.39, 1516.52, 1474.78 (Ar-C=C), 1274.43 (C-O-C), 713.04 (C-S-C). ^1^H NMR (600 MHz, CDCl_3_) *δ* 7.36 (d, *J* = 16.3 Hz, 1H, C_9_-H), 7.24 (t, *J* = 7.9 Hz, 1H, Ar-H), 7.08 (dd, *J* = 8.3, 2.0 Hz, 1H, Ar-H), 7.04 (d, *J* = 2.0 Hz, 1H, Ar-H), 7.01 (d, *J* = 7.6 Hz, 1H, Ar-H), 6.98 (s, 1H, Ar-H), 6.87 (d, *J* = 8.3 Hz, 1H, Ar-H), 6.85–6.82 (m, 2H, Ar-H, C_10_-H), 4.47 (s, 2H, C_13_-H), 3.91 (s, 3H, C_7_-H), 3.90 (s, 3H,C_8_-H), 3.79 (s, 3H, C_20_-H). ^13^C NMR (151 MHz, CDCl_3_) *δ* 165.72, 163.14, 159.84, 150.86, 149.34, 138.57, 137.00, 129.87, 127.75, 121.85, 121.39, 114.62, 113.76, 111.18, 109.10, 107.38, 56.00, 55.92, 55.28, 36.83. HRMS (APCI, *m*/*z*): calcd. for C_20_H_20_N_2_O_4_S^+^ ([M+H]^+^) 385.1217, found 385.1210.

Compound (**6m**)*: (E)-2-(3,4-dimethoxystyryl)-5-((4-methoxybenzyl)thio)-1,3,4-oxadiazole*. White solid; yield, 81.6%; m.p. 72.7–75.2 °C; IR (KBr, cm^−1^): 3055.91 (Ar-H, C=C-H), 2999.88, 2941.79, 2833.89 (C-H), 1642.88 (C=N, C=C), 1600.38, 1584.78, 1510.09, 1470.66 (Ar-C=C), 1265.24 (C-O-C), 731.99 (C-S-C). ^1^H NMR (600 MHz, CDCl_3_) *δ* 7.38–7.33 (m, 3H,Ar-H, C_9_-H), 7.07 (dd, *J* = 8.3, 2.0 Hz, 1H, Ar-H), 7.04 (d, *J* = 2.0 Hz, 1H, Ar-H), 6.88–6.85 (m, 2H, Ar-H), 6.85–6.81 (m, 2H, Ar-H, C_10_-H), 4.45 (s, 2H, C_13_-H), 3.91 (s, 3H, C_7_-H), 3.90 (s, 3H, C_8_-H), 3.77 (s, 3H, C_20_-H). ^13^C NMR (151 MHz, CDCl_3_) *δ* 165.65, 163.27, 159.45, 150.86, 149.35, 138.53, 130.42, 127.77, 127.42, 121.83, 114.22, 111.20, 109.14, 107.41, 56.00, 55.93, 55.30, 36.50. HRMS (APCI, *m*/*z*): calcd. for C_20_H_20_N_2_O_4_S^+^ ([M+H]^+^) 385.1217, found 385.1211.

Compound (**6n**)*: (E)-2-(3,4-dimethoxystyryl)-5-((2-(trifluoromethyl)benzyl)thio)-1,3,4-oxadiazole*. White solid; yield, 82.1%; m.p. 103.8–107.2 °C; IR (KBr, cm^−1^): 3065.44 (Ar-H, C=C-H), 2960.23, 2929.28, 2836.45 (C-H), 1648.14 (C=N, C=C), 1602.75, 1586.25, 1514.04, 1467.98 (Ar-C=C), 1276.09 (C-O-C), 681.86 (C-S-C). ^1^H NMR (600 MHz, CDCl_3_) *δ* 7.77 (d, *J* = 7.7 Hz, 1H, Ar-H), 7.67 (d, *J* = 7.9 Hz, 1H, Ar-H), 7.52 (t, *J* = 7.0 Hz, 1H, Ar-H), 7.41 (t, *J* = 7.7 Hz, 1H, Ar-H), 7.37 (d, *J* = 16.3 Hz, 1H, C_9_-H), 7.08 (dd, *J* = 8.3, 2.0 Hz, 1H, Ar-H), 7.05 (d, *J* = 2.0 Hz, 1H, Ar-H), 6.87 (d, *J* = 8.3 Hz, 1H, Ar-H), 6.85 (d, *J* = 16.3 Hz, 1H, C_10_-H), 4.69 (s, 2H, C_13_-H), 3.92 (s, 3H, C_7_-H), 3.91 (s, 3H, C_8_-H). ^13^C NMR (151 MHz, CDCl_3_) *δ* 165.95, 163.06, 150.91, 149.36, 138.74, 134.48, 132.42, 132.05, 128.34, 127.70, 126.39 (q, *J* = 5.7 Hz), 125.13, 123.31, 121.90, 111.17, 109.09, 107.28, 56.00, 55.92, 33.18 (d, *J* = 2.0 Hz). HRMS (APCI, *m*/*z*): calcd. For C_20_H_17_F_3_N_2_O_3_S^+^ ([M+H]^+^) 423.0985, found 423.0976.

Compound (**6o**)*: (E)-2-(3,4-dimethoxystyryl)-5-((3-(trifluoromethyl)benzyl)thio)-1,3,4-oxadiazole*. White solid; yield, 84.7%; m.p. 82.5–85.3 °C; IR (KBr, cm^−1^): 3057.98 (Ar-H, C=C-H), 2962.54, 2937.64, 2838.04 (C-H), 1640.81 (C=N, C=C), 1601.38, 1586.86, 1514.24, 1474.81 (Ar-C=C), 1275.62 (C-O-C), 702.94 (C-S-C). ^1^H NMR (600 MHz, CDCl_3_) *δ* 7.71 (s, 1H,Ar-H), 7.66 (d, *J* = 7.8 Hz, 1H, Ar-H), 7.55 (d, *J* = 7.9 Hz, 1H, Ar-H), 7.45 (t, *J* = 7.8 Hz, 1H, Ar-H), 7.36 (d, *J* = 16.4 Hz, 1H, C_9_-H), 7.07 (dd, *J* = 8.3, 2.0 Hz, 1H, Ar-H), 7.03 (d, *J* = 2.0 Hz, 1H, Ar-H), 6.87 (d, *J* = 8.3 Hz, 1H, Ar-H), 6.82 (d, *J* = 16.3 Hz, 1H, C_10_-H), 4.52 (s, 2H, C_13_-H), 3.91 (s, 3H, C_7_-H), 3.90 (s, 3H, C_8_-H). ^13^C NMR (151 MHz, CDCl_3_) *δ* 165.93, 162.49, 150.92, 149.35, 138.78, 136.92, 132.59, 131.15 (q, *J* = 32.4 Hz), 129.32, 127.67, 125.86 (q, *J* = 3.8 Hz), 124.93 (q, *J* = 3.8 Hz), 122.97, 121.87, 111.17, 109.11, 107.23, 55.98, 55.91, 36.07. HRMS (APCI, *m*/*z*): calcd. For C_20_H_17_F_3_N_2_O_3_S^+^ ([M+H]^+^) 423.0985, found 423.0977.

Compound (**6p**)*: (E)-2-(3,4-dimethoxystyryl)-5-((4-(trifluoromethyl)benzyl)thio)-1,3,4-oxadiazole*. White solid; yield, 86.5%; m.p. 93.1–96.8 °C; IR (KBr, cm^−1^): 3057.19(Ar-H, C=C-H), 2956.10, 2939.60, 2834.38 (C-H), 1652.26 (C=N, C=C), 1600.69, 1584.18, 1509.91, 1470.72 (Ar-C=C), 1262.35 (C-O-C), 725.96 (C-S-C).^1^H NMR (600 MHz, CDCl_3_) *δ* 7.59 (d, *J* = 2.1 Hz, 4H, Ar-H), 7.36 (d, *J* = 16.3 Hz, 1H, C_9_-H), 7.08 (dd, *J* = 8.3, 2.0 Hz, 1H, Ar-H), 7.04 (d, *J* = 2.0 Hz, 1H, Ar-H), 6.87 (d, *J* = 8.3 Hz, 1H, Ar-H), 6.83 (d, *J* = 16.3 Hz, 1H, C_10_-H), 4.51 (s, 2H, C_13_-H), 3.92 (s, 3H, C_7_-H), 3.91 (s, 3H, C_8_-H). ^13^C NMR (151 MHz, CDCl_3_) *δ* 165.95, 162.50, 150.94, 149.36, 139.96, 138.81, 130.27 (q, *J* = 32.4 Hz), 129.52, 127.66, 125.74 (q, *J* = 3.8 Hz), 121.90, 111.17, 109.09, 107.23, 56.00, 55.92, 35.98. HRMS (APCI, *m*/*z*): calcd. For C_20_H_17_F_3_N_2_O_3_S^+^ ([M+H]^+^) 423.0985, found 423.0977.

Compound (**6q**)*: (E)-2-(3,4-dimethoxystyryl)-5-((2-(trifluoromethoxy)benzyl)thio)-1,3,4-oxadiazole*. White solid; yield, 79.1%; m.p. 84.5–87.3 °C; IR (KBr, cm^−1^): 3057.98 (Ar-H, C=C-H), 2987.44, 2960.46, 2838.04 (C-H), 1638.73 (C=N, C=C), 1601.38, 1580.63, 1516.31, 1476.89(Ar-C=C), 1265.24 (C-O-C), 698.79 (C-S-C). ^1^H NMR (600 MHz, CDCl_3_) *δ* 7.63 (dd, *J* = 7.6, 1.7 Hz, 1H,Ar-H), 7.37 (d, *J* = 16.4 Hz, 1H, C_9_-H), 7.34 (dd, *J* = 8.3, 7.3, 1.7 Hz, 1H, Ar-H), 7.29–7.23 (m, 2H, Ar-H), 7.08 (dd, *J* = 8.3, 2.0 Hz, 1H, Ar-H), 7.04 (d, *J* = 2.0 Hz, 1H, Ar-H), 6.87 (d, *J* = 8.3 Hz, 1H, Ar-H), 6.84 (d, *J* = 16.4 Hz, 1H, C_10_-H), 4.54 (s, 2H, C_13_-H), 3.92 (s, 3H, C_7_-H), 3.91 (s, 3H, C_8_-H). ^13^C NMR (151 MHz, CDCl_3_) *δ* 165.91, 162.86, 150.91, 149.37, 147.60, 138.69, 131.68, 129.74, 128.37, 127.74, 126.92, 122.47–118.69 (q), 111.20, 109.12, 107.32, 56.00, 55.92, 30.93. HRMS (APCI, *m*/*z*): calcd. for C_20_H_17_F_3_N_2_O_4_S^+^ ([M+H]^+^) 439.0934, found 439.0927.

Compound (**6r**)*: (E)-2-(3,4-dimethoxystyryl)-5-((3-(trifluoromethoxy)benzyl)thio)-1,3,4-oxadiazole*. White solid; yield, 80.2%; m.p. 70.3–72.9 °C; IR (KBr, cm^−1^): 3033.74 (Ar-H, C=C-H), 2977.39, 2948.17, 2843.83 (C-H), 1652.17, 1633.39 (C=N, C=C), 1602.09, 1587.48, 1518.61, 1476.87 (Ar-C=C), 1249.39 (C-O-C), 698.43 (C-S-C). ^1^H NMR (600 MHz, CDCl_3_) *δ* 7.41–7.36 (m, 2H, Ar-H, C_9_-H), 7.35 (d, *J* = 2.9 Hz, 1H, Ar-H), 7.33 (d, *J* = 14.7 Hz, 1H, Ar-H), 7.15 (d, *J* = 8.0 Hz, 1H, Ar-H), 7.08 (dd, *J* = 8.4, 2.1 Hz, 1H, Ar-H), 7.04 (d, *J* = 2.1 Hz, 1H, Ar-H), 6.87 (d, *J* = 8.3 Hz, 1H, Ar-H), 6.83 (d, *J* = 16.4 Hz, 1H, C_10_-H), 4.48 (s, 2H, C_13_-H), 3.92 (s, 3H, C_7_-H), 3.90 (s, 3H, C_8_-H). ^13^C NMR (151 MHz, CDCl_3_) *δ* 165.90, 162.56, 150.93, 149.38 (d, *J* = 4.5 Hz), 138.75, 138.10, 130.18, 127.70, 127.53, 121.87, 121.72–119.30 (q), 111.19, 109.12, 107.27, 56.00, 55.91, 36.03. HRMS (APCI, *m*/*z*): calcd. for C20H17F3N2O4S^+^ ([M+H]^+^) 439.0934, found 439.0926.

Compound (**6s**)*: (E)-2-(3,4-dimethoxystyryl)-5-((4-(trifluoromethoxy)benzyl)thio)-1,3,4-oxadiazole*. White solid; yield, 76.5%; m.p. 78.9–82.3 °C; IR (KBr, cm^−1^): 3048.94 (Ar-H, C=C-H), 2958.17, 2931.35, 2836.45 (C-H), 1637.82 (C=N, C=C), 1600.69, 1586.25, 1518.17, 1478.97 (Ar-C=C), 1252.03 (C-O-C), 709.46 (C-S-C). ^1^H NMR (600 MHz, CDCl_3_) *δ* 7.48 (d, *J* = 8.6 Hz, 2H,Ar-H), 7.36 (d, *J* = 16.3 Hz, 1H, C_9_-H), 7.17 (d, *J* = 7.7 Hz, 2H, Ar-H), 7.08 (dd, *J* = 8.3, 2.0 Hz, 1H, Ar-H), 7.04 (d, *J* = 2.0 Hz, 1H, Ar-H), 6.87 (d, *J* = 8.3 Hz, 1H, Ar-H), 6.83 (d, *J* = 16.4 Hz, 1H, C_10_-H), 4.48 (s, 2H, C_13_-H), 3.91 (s, 3H, C_7_-H), 3.90 (s, 3H, C_8_-H). ^13^C NMR (151 MHz, CDCl_3_) *δ* 165.87, 162.71, 150.93, 149.15 (d, *J* = 67.3 Hz), 138.73, 134.54, 130.65, 127.69, 121.87, 121.21, 111.19, 109.12, 107.28, 55.99, 55.91, 35.82. HRMS (APCI, *m*/*z*): calcd. for C_20_H_17_F_3_N_2_O_4_S^+^ ([M+H]^+^) 439.0934, found 439.0928.

Compound (**6t**)*: (E)-2-((3,4-dichlorobenzyl)thio)-5-(3,4-dimethoxystyryl)-1,3,4-oxadiazole*. White solid; yield, 75.8%; m.p. 110.1–118.7 °C; IR (KBr, cm^−1^): 3048.94 (Ar-H, C=C-H), 2960.23, 2939.60, 2830.26 (C-H), 1639.89 (C=N, C=C), 1600.69, 1588.31, 1514.04, 1472.78 (Ar-C=C), 1270.60 (C-O-C), 740.40 (C-S-C). ^1^H NMR (600 MHz, CDCl_3_) *δ* 7.55 (d, *J* = 2.1 Hz, 1H,Ar-H), 7.39 (d, *J* = 8.3 Hz, 1H Ar-H), 7.36 (d, *J* = 16.3 Hz, 1H, C_9_-H), 7.30 (dd, *J* = 8.3, 2.1 Hz, 1H, Ar-H), 7.08 (dd, *J* = 8.3, 2.0 Hz, 1H, Ar-H), 7.04 (d, *J* = 2.0 Hz, 1H, Ar-H), 6.87 (d, *J* = 8.3 Hz, 1H, Ar-H), 6.82 (d, *J* = 16.3 Hz, 1H, C_10_-H), 4.41 (s, 2H, C_13_-H) 3.92 (s, 3H, C_7_-H), 3.91 (s, 3H, C_8_-H). ^13^C NMR (151 MHz, CDCl_3_) *δ* 165.96, 162.38, 150.94, 149.35, 138.84, 136.16, 132.77, 132.29, 131.02, 130.71, 128.54, 127.65, 121.90, 111.18, 109.12, 107.21, 56.01, 55.94, 35.42. HRMS (APCI, *m*/*z*): calcd. for C_19_H_16_Cl_2_N_2_O_3_S^+^ ([M+H]^+^) 423.0331, found 423.0325.

Compound (**6u**)*: (E)-2-((2,3-dichlorobenzyl)thio)-5-(3,4-dimethoxystyryl)-1,3,4-oxadiazole*. White solid; yield, 75.8%; m.p. 118.1–122.3 °C; IR (KBr, cm^−1^): 3076.38 (Ar-H, C=C-H), 2994.52, 2939.94, 2837.08 (C-H), 1634.29 (C=N, C=C), 1600.80, 1588.10, 1516.73, 1472.65 (Ar-C=C), 1273.24 (C-O-C), 725.36 (C-S-C). ^1^H NMR (600 MHz, CDCl_3_) *δ* 7.54 (dd, *J* = 7.7, 1.6 Hz, 1H,Ar-H), 7.40 (dd, *J* = 8.1, 1.6 Hz, 1H, Ar-H), 7.35 (d, *J* = 16.3 Hz, 1H, C_9_-H), 7.15 (t, *J* = 7.9 Hz, 1H, Ar-H), 7.07 (dd, *J* = 8.3, 2.0 Hz, 1H, Ar-H), 7.03 (d, *J* = 2.0 Hz, 1H, Ar-H), 6.86 (d, *J* = 8.3 Hz, 1H, Ar-H), 6.82 (d, *J* = 16.4 Hz, 1H, C_10_-H), 4.60 (s, 2H, C_13_-H), 3.91 (s, 3H, C_7_-H), 3.90 (s, 3H, C_8_-H). ^13^C NMR (151 MHz, CDCl_3_) *δ* 165.95, 162.80, 150.91, 149.34, 138.75, 136.14, 133.56, 132.68, 130.33, 129.65, 127.68, 127.43, 121.90, 111.17, 109.10, 107.24, 56.01, 55.93, 35.20. HRMS (APCI, *m*/*z*): calcd. for C_19_H_16_Cl_2_N_2_O_3_S^+^ ([M+H]^+^) 423.0331, found 423.0327.

Compound (**6v**)*: (E)-2-((2-chloro-4-fluorobenzyl)thio)-5-(3,4-dimethoxystyryl)-1,3,4-oxadiazole*. White solid; yield, 75.1%; m.p. 117.8–121.3 °C; IR (KBr, cm^−1^): 3059.25 (Ar-H, C=C-H), 2935.47, 2912.78, 2834.38 (C-H), 1654.33,1639.89 (C=N, C=C), 1600.69, 1588.31, 1514.04, 1474.84 (Ar-C=C), 1276.79 (C-O-C), 732.15 (C-S-C). ^1^H NMR (600 MHz, CDCl_3_) *δ* 7.61 (dd, *J* = 8.6, 6.0 Hz, 1H,Ar-H), 7.36 (d, *J* = 16.3 Hz, 1H, C_9_-H), 7.14 (dd, *J* = 8.4, 2.6 Hz, 1H, Ar-H), 7.07 (dd, *J* = 8.3, 2.0 Hz, 1H, Ar-H), 7.03 (d, *J* = 2.0 Hz, 1H, Ar-H), 6.94 (td, *J* = 8.3, 2.6 Hz, 1H, Ar-H), 6.86 (d, *J* = 8.3 Hz, 1H, Ar-H), 6.82 (d, *J* = 16.3 Hz, 1H, C_10_-H), 4.55 (s, 2H, C_13_-H), 3.91 (s, 3H, C_7_-H), 3.90 (s, 3H, C_8_-H). ^13^C NMR (151 MHz, CDCl_3_) *δ* 165.91, 162.99 (d, *J* = 36.3 Hz), 161.44, 150.90, 149.35, 138.72, 135.12 (d, *J* = 10.5 Hz), 132.64 (d, *J* = 9.0 Hz), 129.84 (d, *J* = 3.7 Hz), 127.68, 121.89, 117.22 (d, *J* = 24.9 Hz), 114.33 (d, *J* = 21.4 Hz), 111.17, 109.08, 107.26, 56.00, 55.92, 33.84. HRMS (APCI, *m*/*z*): calcd. For C_19_H_16_ClFN_2_O_3_S^+^ ([M+H]^+^) 407.0627, found 407.0621.

Compound (**6w**)*: (E)-4-(((5-(3,4-dimethoxystyryl)-1,3,4-oxadiazol-2-yl)thio)methyl)benzonitrile*. White solid; yield, 71.3%; m.p. 168.5–172.4 °C; IR (KBr, cm^−1^): 3070.09 (Ar-H, C=C-H), 2964.36, 2929.28, 2830.26 (C-H),2229.91 (-C≡N), 1639.89 (C=N, C=C), 1602.75, 1588.31, 1514.04, 1474.84 (Ar-C=C), 1268.54 (C-O-C), 666.13 (C-S-C).^1^H NMR (600 MHz, CDCl_3_) *δ* 7.64–7.55 (m, 4H,Ar-H), 7.35 (d, *J* = 16.4 Hz, 1H,C_9_-H), 7.07 (dd, *J* = 8.3, 2.0 Hz, 1H, Ar-H), 7.03 (d, *J* = 2.0 Hz, 1H, Ar-H), 6.87 (d, *J* = 8.3 Hz, 1H, Ar-H), 6.82 (d, *J* = 16.3 Hz, 1H, C_10_-H), 4.49 (s, 2H, C_13_-H), 3.91 (s, 3H, C_7_-H), 3.90 (s, 3H, C_8_-H). ^13^C NMR (151 MHz, CDCl_3_) *δ* 166.05, 162.22, 150.98, 149.36, 141.45, 138.95, 132.52, 129.92, 127.59, 121.97, 118.46, 111.95, 111.19, 109.09, 107.11, 56.02, 55.95, 35.96. HRMS (APCI, *m*/*z*): calcd. for C_20_H_17_N_3_O_3_S^+^ ([M+H]^+^) 380.1063, found 380.1056.

Compound (**6x**)*: (E)-2-((4-(tert-butyl)benzyl)thio)-5-(3,4-dimethoxystyryl)-1,3,4-oxadiazole*. White solid; yield, 71.3%; m.p. 117.1–122.3 °C; IR (KBr, cm^−1^): 3055.91 (Ar-H, C=C-H), 2958.39, 2931.41, 2840.12 (C-H), 1647.03 (C=N, C=C), 1601.38, 1584.78, 1508.01, 1472.74 (Ar-C=C), 1265.24 (C-O-C), 702.94 (C-S-C). ^1^H NMR (600 MHz, CDCl_3_) *δ* 7.40–7.34 (m, 5H, Ar-H, C_9_-H), 7.09 (dd, *J* = 8.4, 2.0 Hz, 1H, Ar-H), 7.05 (d, *J* = 2.0 Hz, 1H, Ar-H), 6.89–6.83 (m, 2H, Ar-H, C_8_-H), 4.49 (s, 2H, C_13_-H), 3.92 (s, 3H, C_7_-H), 3.91 (s, 3H, C_8_-H), 1.31 (s, 9H, C_20,21,22_-H). ^13^C NMR (151 MHz, CDCl_3_) *δ* 165.66, 163.34, 151.20, 150.85, 149.35, 138.48, 132.36, 128.88, 127.79, 125.79, 121.83, 111.18, 109.08, 107.47, 56.01, 55.93, 36.53, 34.61, 31.29. HRMS (APCI, *m*/*z*): calcd. for C_23_H_26_N_2_O_3_S^+^ ([M+H]^+^) 411.1737, found 411.1730.

Compound (**6y**)*: (E)-2-(3,4-dimethoxystyryl)-5-((pyridin-2-ylmethyl)thio)-1,3,4-oxadiazole*. White solid; yield, 76.4%; m.p. 105.3–109.5 °C; IR (KBr, cm^−1^): 3079.65 (Ar-H, C=C-H), 2984.17, 2929.39, 2833.39 (C-H), 1645.91 (C=N, C=C), 1600.00, 1583.30, 1506.17, 1474.78 (Ar-C=C), 1268.17 (C-O-C), 729.74 (C-S-C). ^1^H NMR (600 MHz, CDCl_3_) *δ* 8.56 (d, *J* = 4.9 Hz, 1H, Ar-H), 7.64 (td, *J* = 7.7, 1.8 Hz, 1H, Ar-H), 7.50 (d, *J* = 7.8 Hz, 1H, Ar-H), 7.35 (d, *J* = 16.3 Hz, 1H, C_9_-H), 7.19 (dd, *J* = 6.5, 4.8 Hz, 1H, Ar-H), 7.06 (dd, *J* = 8.3, 2.0 Hz, 1H, Ar-H), 7.02 (d, *J* = 2.0 Hz, 1H, Ar-H), 6.85 (d, *J* = 8.3 Hz, 1H Ar-H), 6.83 (s, 1H, C_10_-H), 4.62 (s, 2H, C_13_-H), 3.89 (s, 3H, C_7_-H), 3.88 (s, 3H, C_8_-H). ^13^C NMR (151 MHz, CDCl_3_) *δ* 165.81, 163.24, 155.47, 150.84, 149.68, 149.32, 138.60, 136.90, 127.72, 123.49, 122.78, 121.84, 111.16, 109.09, 107.32, 55.99, 55.91, 38.30. HRMS (APCI, *m*/*z*): calcd. for C_18_H_17_N_3_O_3_S^+^ ([M+H]^+^) 356.1063, found 356.1057.

Compound (**6z**)*: (E)-2-(3,4-dimethoxystyryl)-5-((naphthalen-2-ylmethyl)thio)-1,3,4-oxadiazole*. White solid; yield, 72.9%; m.p. 133.6–138.9 °C; IR (KBr, cm^−1^): 3047.00 (Ar-H, C=C-H), 2963.03, 2939.94, 2828.69 (C-H), 1648.98 (C=N, C=C), 1600.70, 1586.01, 1510.44, 1474.75 (Ar-C=C), 1264.84 (C-O-C), 731.66 (C-S-C). ^1^H NMR (600 MHz, CDCl_3_) *δ* 8.10 (d, *J* = 8.4 Hz, 1H, Ar-H), 7.88 (d, *J* = 7.5 Hz, 1H, Ar-H), 7.83 (d, *J* = 8.2 Hz, 1H, Ar-H), 7.63 (d, *J* = 5.9 Hz, 1H, Ar-H), 7.60–7.56 (m, 1H, Ar-H), 7.54–7.51 (m, 1H, Ar-H), 7.42 (dd, *J* = 8.3, 7.0 Hz, 1H, Ar-H), 7.35 (d, *J* = 16.4 Hz, 1H, C_9_-H), 7.07 (dd, *J* = 8.3, 2.0 Hz, 1H, Ar-H), 7.04 (d, *J* = 2.0 Hz, 1H, Ar-H), 6.87 (d, *J* = 2.1 Hz, 1H, Ar-H), 6.85 (d, *J* = 5.9 Hz, 1H, C_10_-H), 5.00 (s, 2H, C_13_-H), 3.92 (s, 3H, C_7_-H), 3.90 (s, 3H, C_8_-H). ^13^C NMR (151 MHz, CDCl_3_) *δ* 165.75, 163.36, 150.87, 149.34, 138.60, 133.98, 131.31, 130.69, 129.35, 129.05, 128.41, 127.75, 126.79, 126.17, 125.43, 123.40, 121.87, 111.18, 109.10, 107.38, 56.01, 55.93, 34.87. HRMS (APCI, *m*/*z*): calcd. For C_23_H_20_N_2_O_3_S^+^ ([M+H]^+^) 405.1267, found 405.1259.

Compound (**6aa**)*: (E)-2-((cyclohexylmethyl)thio)-5-(3,4-dimethoxystyryl)-1,3,4-oxadiazole*. White solid; yield, 76.3%; m.p. 83.8–86.7 °C; IR (KBr, cm^−1^): 3055.13 (Ar-H, C=C-H), 2963.80, 2927.78, 2829.20 (C-H), 1641.95 (C=N, C=C), 1600.69, 1582.12, 1506.71, 1472.52 (Ar-C=C), 1265.28 (C-O-C), 735.43 (C-S-C). ^1^H NMR (600 MHz, CDCl_3_) *δ* 7.36 (d, *J* = 16.3 Hz, 1H,C_9_-H), 7.07 (dd, *J* = 8.3, 2.0 Hz, 1H, Ar-H), 7.04 (d, *J* = 2.0 Hz, 1H, Ar-H), 6.87–6.82 (m, 2H, Ar-H, C_10_-H), 3.91 (s, 3H, C_7_-H), 3.89 (s, 3H, C_8_-H), 3.17 (d, *J* = 6.9 Hz, 2H, C_13_-H), 1.89 (d, *J* = 11.6 Hz, 2H, C_15_-H), 1.73 (dt, *J* = 13.6, 3.6 Hz, 3H, C_16,17_-H), 1.67–1.62 (m, 1H, C_14_-H), 1.29–1.20 (m, 2H, C_18_-H), 1.19–1.10 (m, 1H, C_17_-H), 1.02 (qd, *J* = 12.3, 3.5 Hz, 2H, C_19_-H). ^13^C NMR (151 MHz, CDCl_3_) *δ* 165.47, 164.24, 150.79, 149.32, 138.27, 127.82, 121.80, 111.16, 109.04, 107.52, 56.00, 55.91, 39.47, 37.47, 32.38, 26.11, 25.85. HRMS (APCI, *m*/*z*): calcd. for C_19_H_24_N_2_O_3_S^+^ ([M+H]^+^) 361.1580, found 361.1575.

Compound (**6ab**)*: (E)-2-((cyclopentylmethyl)thio)-5-(3,4-dimethoxystyryl)-1,3,4-oxadiazole*. White solid; yield, 75.6%; m.p. 68.1–75.8 °C; IR (KBr, cm^−1^): 3013.87 (Ar-H, C=C-H), 2968.48, 2937.54, 2832.32 (C-H), 1635.76 (C=N, C=C), 1598.62, 1588.31, 1516.10, 1478.97 (Ar-C=C), 1264.41 (C-O-C), 771.35 (C-S-C). ^1^H NMR (600 MHz, CDCl_3_) *δ* 7.37 (d, *J* = 16.4 Hz, 1H, C_9_-H), 7.07 (dd, *J* = 8.3, 2.0 Hz, 1H, Ar-H), 7.04 (d, *J* = 2.0 Hz, 1H, Ar-H), 6.88–6.82 (m, 2H, Ar-H, C_10_-H), 3.91 (s, 3H, C_7_-H), 3.89 (s, 3H, C_8_-H), 3.28 (d, *J* = 7.4 Hz, 2H, C_13_-H), 2.36–2.25 (m, 1H, C_14_-H), 1.93–1.85 (m, 2H, C_15_-H), 1.70–1.62 (m, 2H, C_18_-H), 1.61–1.53 (m, 2H, C_16_-H), 1.30 (dq, *J* = 12.7, 8.2 Hz, 2H, C_17_-H). ^13^C NMR (151 MHz, CDCl_3_) *δ* 165.47, 164.07, 150.77, 149.31, 138.24, 127.80, 121.77, 111.15, 109.03, 107.52, 55.99, 55.90, 39.37, 38.34, 32.22, 25.22. HRMS (APCI, *m*/*z*): calcd. for C_18_H_22_N_2_O_3_S^+^ ([M+H]^+^) 347.1424, found 347.1416.

Compound (**6ac**)*: (E)-2-(((5-chlorothiophen-2-yl)methyl)thio)-5-(3,4-dimethoxystyryl)-1,3,4-oxadiazole*. White solid; yield, 77.2%; m.p. 107.8–110.3 °C; IR (KBr, cm^−1^): 3051.00 (Ar-H, C=C-H), 2958.17, 2931.35, 2840.57 (C-H), 1641.95 (C=N, C=C), 1602.75, 1586.25, 1509.91, 1476.91 (Ar-C=C), 1268.54 (C-O-C), 703.27 (C-S-C). ^1^H NMR (600 MHz, CDCl_3_) *δ* 7.38 (d, *J* = 16.3 Hz, 1H, C_9_-H), 7.09 (dd, *J* = 8.4, 2.1 Hz, 1H, Ar-H), 7.05 (d, *J* = 2.0 Hz, 1H, Ar-H), 6.91–6.88 (m, 1H, Ar-H), 6.87 (d, *J* = 8.3 Hz, 1H, Ar-H), 6.84 (d, *J* = 16.3 Hz, 1H, C_10_-H), 6.73 (d, *J* = 3.8 Hz, 1H, Ar-H), 4.60 (s, 2H, C_13_-H), 3.92 (s, 3H, C_7_-H), 3.91 (s, 3H, C_8_-H). ^13^C NMR (151 MHz, CDCl_3_) *δ* 166.01, 162.33, 150.93, 149.36, 138.87, 136.81, 130.35, 127.68, 127.42, 125.95, 121.92, 111.18, 109.11, 107.24, 56.02, 55.94, 31.66. HRMS (APCI, *m*/*z*): calcd. for C_17_H_15_ClN_2_O_3_S_2_^+^ ([M+H]^+^) 395.0285, found 395.0280.

Compound (**6ad**)*: (E)-2-(3,4-dimethoxystyryl)-5-(methylthio)-1,3,4-oxadiazole*. White solid; yield, 85.3%; m.p.123.3–131.5 °C; IR (KBr, cm^−1^): 3046.40 (Ar-H, C=C-H), 2943.23, 2928.79, 2850.39 (C-H), 1639.23 (C=N, C=C), 1602.09, 1587.65, 1511.31, 1474.17 (Ar-C=C), 1282.28 (C-O-C), 710.75 (C-S-C). ^1^H NMR (600 MHz, CDCl_3_) *δ* 7.37 (d, *J* = 16.4 Hz, 1H, C_9_-H), 7.07 (dd, *J* = 8.2, 2.0 Hz, 1H, Ar-H), 7.04 (d, *J* = 2.0 Hz, 1H, Ar-H), 6.88–6.81 (m, 2H, Ar-H, C_10_-H), 3.91 (s, 3H, C_7_-H), 3.89 (s, 3H, C_8_-H), 2.73 (s, 3H, C_13_-H). ^13^C NMR (151 MHz, CDCl_3_) *δ* 165.70, 164.31, 150.82, 149.33, 138.38, 127.76, 121.80, 111.17, 109.06, 107.43, 56.00, 55.91, 14.57. HRMS (APCI, *m*/*z*): calcd. for C_13_H_14_N_2_O_3_S^+^ ([M+H]^+^) 279.0798, found 279.0793.

Compound (**6ae**)*: (E)-2-(3,4-dimethoxystyryl)-5-(ethylthio)-1,3,4-oxadiazole*. White solid; yield, 80.6%; m.p. 93.1–96.8 °C; IR (KBr, cm-1): 3053.07 (Ar-H, C=C-H), 2965.15, 2925.65, 2831.18 (C-H), 1644.92 (C=N, C=C), 1599.84, 1586.87, 1505.91, 1471.31 (Ar-C=C), 1264.76 (C-O-C), 605.39 (C-S-C). ^1^H NMR (600 MHz, CDCl_3_) *δ* 7.37 (d, *J* = 16.4 Hz, 1H, C_9_-H), 7.07 (dd, *J* = 8.3, 2.0 Hz, 1H,Ar-H), 7.04 (d, *J* = 2.0 Hz, 1H, Ar-H), 6.87 (d, *J* = 2.7 Hz, 1H, Ar-H), 6.85 (d, *J* = 5.4 Hz, 1H, C_10_-H), 3.91 (s, 3H, C_7_-H), 3.89 (s, 3H. C_8_-H), 3.28 (q, *J* = 7.4 Hz, 2H, C_13_-H), 1.48 (t, *J* = 7.4 Hz, 3H, C_14_-H). ^13^C NMR (151 MHz, CDCl_3_) *δ* 165.57, 163.64, 150.81, 149.32, 138.40, 127.78, 121.82, 111.16, 109.06, 107.46, 56.00, 55.92, 26.97, 14.74. HRMS (APCI, *m*/*z*): calcd. for C_14_H_16_N_2_O_3_S^+^ ([M+H]^+^) 293.0954, found 293.0948.

Compound (**6af**)*: (E)-2-(3,4-dimethoxystyryl)-5-(isopentylthio)-1,3,4-oxadiazole*. White solid; yield, 78.5%; m.p. 73.6–77.4 °C; IR (KBr, cm-1): 3051.00 (Ar-H, C=C-H), 2960.23, 2937.54, 2838.51 (C-H), 1644.01 (C=N, C=C), 1600.69, 1588.31, 1509.91, 1472.78 (Ar-C=C), 1266.48 (C-O-C), 728.02 (C-S-C). ^1^H NMR (600 MHz, CDCl_3_) *δ* 7.37 (d, *J* = 16.3 Hz, 1H, C_9_-H), 7.07 (dd, *J* = 8.3, 2.0 Hz, 1H, Ar-H), 7.04 (d, *J* = 1.9 Hz, 1H, Ar-H), 6.88–6.82 (m, 2H, Ar-H, C_10_-H), 3.91 (s, 3H, C_7_-H), 3.90 (s, 3H, C_8_-H), 3.30–3.24 (m, 2H, C_13_-H), 1.74 (dt, *J* = 13.0, 6.5 Hz, 1H, C_17_-H), 1.71–1.66 (m, 2H, C_14_-H), 0.94 (d, *J* = 6.5 Hz, 6H, C_15_,_16_-H). ^13^C NMR (151 MHz, CDCl_3_) *δ* 165.51, 163.84, 150.80, 149.33, 138.29, 127.80, 121.80, 111.16, 109.04, 107.51, 55.99, 55.91, 37.97, 30.69, 27.40, 22.15. HRMS (APCI, *m*/*z*): calcd. for C_17_H_23_N_2_O_3_S^+^ ([M+H]^+^) 335.1424, found 335.1418.

### 3.7. In Vitro Antifungal Activity

Using the commercially available fungicide boscalid as the positive control, in vitro antifungal activity of the target compounds **6a**–**6af** against eight strains of phytopathogenic fungi was assessed via the agar dilution method. Each test compound was dissolved in acetone and formulated into a test solution with a concentration of 500 μg/mL using Sophor-144 (0.2 g/L) as the diluent. A 1 mL aliquot of the drug-containing solution was mixed thoroughly with 9 mL of potato sucrose agar (PSA) medium to prepare drug-loaded culture plates with a final concentration of 50 μg/mL. Subsequently, three 0.4 cm-diameter agar plugs containing the mycelia of the test pathogen were inoculated onto each drug-loaded PSA plate. All plates were incubated in a constant-temperature incubator at 24 ± 1 °C under dark conditions for 48 h. Following incubation, the radial growth diameter of the mycelia was measured. Each compound was tested in triplicate, and the mean value was calculated. The relative inhibition of the circle of mycelium compared with the blank assay was calculated by the equation below:

$$\mathrm{Relative}\;\mathrm{inhibitory}\;\mathrm{rate}\;(\%)\;=\;\frac{CK-PT}{CK}\;\times \;100\%$$

where ***CK*** is the extended diameter of the circle of mycelium during the blank assay and ***PT*** is the extended diameter of the circle of mycelium during testing.

### 3.8. 3D-QSAR

According to the reported procedure [40], 36 three-dimensional quantitative structure–activity relationship (3D-QSAR) analysis of the target compounds was performed via the comparative molecular field analysis (CoMFA) method using Sybyl-X 2.1.1 software. Experimental ED values were used to characterize the antifungal activities of the target compounds against *P. p.* The ED value was calculated according to the formula $ED=lg\left\{I/\left[\left(100-I\right)\times MV\right]\right\}$, where I and MW represent the mycelial inhibition rate and molecular weight of the target compound, respectively. Compound **6n**, which exhibited the optimal antifungal activity, was chosen as the template molecule for model construction. Compounds **6a**–**6x** bearing substituted phenyl groups were employed to build the 3D-QSAR model, in which 22 target compounds were assigned to the training set, and the remaining 2 compounds were classified as the test set. Subsequently, molecular structure energy minimization optimization was performed on the compounds **6a**–**6x**. Partial least-squares (PLS) regression analysis was carried out to evaluate the validity, rationality and reliability of the constructed 3D-QSAR model.

### 3.9. DFT Calculation

Electrostatic potential (ESP) distribution and frontier molecular orbital (FMO) calculations for target compounds **6a** (R = 2-CH_3_ Bn) and **6n** (R = 2-CF_3_ Bn) were carried out using Gaussian 16 software based on density functional theory (DFT) at the B3LYP/6-31G(d,p) level of theory. The calculation results were visualized with GaussView 6.0.16 software. All the aforementioned theoretical calculations were performed on the High-Performance Computing Platform of Guangxi University.

### 3.10. SDH Inhibitory Activity Assay

The inhibitory activity of compounds **6n**, **6o**, **6r**, and the positive control boscalid against succinate dehydrogenase (SDH) was evaluated using an SDH ELISA kit. Each test compound was individually dissolved in DMSO, followed by serial dilution with buffer to achieve the specified concentrations. All subsequent experimental procedures were performed strictly in accordance with the manufacturer’s instructions for the kit and the methodologies reported in the literature [41].

### 3.11. Molecular Docking

Owing to the high respiratory activity of chicken heart tissue and the ready availability of this protein, whose crystal structure has been fully resolved, chicken-derived succinate dehydrogenase (SDH, PDB ID: 2WQY) was selected as the template in this study [42]. First, we downloaded the crystal structure of succinate dehydrogenase (SDH) from the PDB database. We used PyMOL 3.10 software to remove water molecules, irrelevant heteroatoms and the original ligand, while retaining the main-chain active structure of the protein. Afterwards, we performed receptor structure optimization via AutoDockTools-1.5.6. We constructed the three-dimensional configuration of selected target compounds as ligands in ChemDraw 26.0.0 and carried out energy minimization optimization. We converted the files into PDBQT format suitable for docking using Open Babel 3.2.0, and calculated Gasteiger charges simultaneously to ensure the ligands adopted the lowest-energy and stable conformations. We analyzed hydrogen bonds, hydrophobic interactions and other interactions by PyMOL 2.6.2 and LigPlot+ v2.2.5. The protein model built via homology modeling in this work can well characterize the structural features of key binding regions of the target protein.

### 3.12. Characterization

The ^l^H and ^13^C NMR spectra of vanillin-based 1,3,4-oxadiazole-thioether compounds **6a**~**6af** were recorded using an Avance III HD 600 MHz nuclear magnetic resonance spectrometer (Switzerland Bruker Co., Ltd., Zurich, Switzerland) with CDCl_3_ as the solvent and TMS as the internal standard. The FT-IR spectra were obtained on a Nicolet iS50 spectrometer (Thermo Scientific Co., Ltd., Waltham, MA, USA) employing the KBr pellet method. HRMS spectra were acquired on a Q Exactive mass spectrometer (Thermo Scientific Co., Ltd., Waltham, MA, USA). Melting points were determined using an MP420 automatic melting point analyzer (Hanon Instruments Co., Ltd., Jinan, China).

## 4. Conclusions

Thirty-two novel vanillin-based 1,3,4-oxadiazole-thioether derivatives (**6a**–**6f**) were designed and synthesized in this work, and their chemical structures were characterized by FT-IR, ^1^H NMR, ^13^C NMR, and HRMS. A preliminary screening of the antifungal activity of the target compounds was conducted at a concentration of 50 μg·mL^−1^. Bioactivity assays demonstrated that compounds **6n**, **6r**, and **6o** exhibited excellent inhibitory activity against *P. p.*, with EC_50_ values of 9.57 μg·mL^−1^, 11.8 μg·mL^−1^, and 15.64 μg·mL^−1^, respectively, all superior to that of the positive control boscalid with EC_50_ of 16.60 μg·mL^−1^. Furthermore, a reliable and valid 3D-QSAR model was constructed using the CoMFA method to explore the structure–activity relationship of molecular structures of the compounds with respect to their antifungal activity against *P. p.* Concurrently, DFT calculations, including ESP and FMO, were performed to further elucidate the structure–activity relationship. It was found from the SDH inhibition activity assay that compounds **6o**, **6n**, and **6r** exhibited significant SDH inhibitory activity, with IC_50_ values of 1.29 μM, 2.09 μM, and 2.34 μM, respectively, remarkably better than that of the positive control boscalid with an IC_50_ of 7.02 μM. Molecular docking simulation results revealed that both compound **6n** and boscalid could enter the active pocket of SDH, with analogous binding modes within the SDH active site. The results indicated that compound **6n** might exert its potent antifungal activity by targeting SDH. Therefore, compound **6n** deserves in-depth investigation as a lead candidate for the development of novel fungicides targeting SDH.

## Figures and Tables

**Table 1:** Antifungal activity of the target compounds **6a**–**6af** at the concentration of 50 μg/mL.

Compd.	Relative Inhibitory Rate (%)							
	*F. o.*	*C. a.*	*P. p.*	*A. s.*	*G. z.*	*R. s.*	*B. m.*	*C. o.*
**6a**	0.0	83.3 ± 0.8	29.1 ± 1.2	15.0 ± 1.0	9.1 ± 0.1	1.7 ± 1.3	51.1 ± 0.2	31.9 ± 0.3
**6b**	7.8 ± 2.1	33.3 ± 2.0	35.4 ± 0.3	10.0 ± 0.5	45.5 ± 0.2	25.0 ± 1.2	14.9 ± 0.3	10.6 ± 0.5
**6c**	5.9 ± 0.9	19.4 ± 0.6	22.8 ± 0.4	10.0 ± 0.9	18.2 ± 0.6	15.0 ± 0.5	8.5 ± 0.5	10.6 ± 0.6
**6d**	5.9 ± 1.4	22.2 ± 1.2	41.8 ± 2.0	15.0 ± 1.1	27.3 ± 0.5	10.0 ± 0.6	4.3 ± 0.9	4.3 ± 1.2
**6e**	7.8 ± 1.2	13.9 ± 0.4	19.0 ± 1.1	20.0 ± 2.0	2.3 ± 0.4	45.0 ± 0.6	2.1 ± 0.8	10.6 ± 0.6
**6f**	2.0 ± 1.5	30.6 ± 0.9	34.2 ± 0.4	10.0 ± 0.6	4.5 ± 1.1	8.3 ± 2.0	10.6 ± 0.2	8.5 ± 0.4
**6g**	2.0 ± 0.7	16.7 ± 0.5	45.6 ± 0.8	10.0 ± 1.1	22.7 ± 1.6	10.0 ± 1.4	8.5 ± 0.1	8.5 ± 0.6
**6h**	7.8 ± 0.6	19.4 ± 1.6	32.9 ± 0.6	20.0 ± 0.3	4.5 ± 2.0	13.3 ± 1.3	8.5 ± 1.2	10.6 ± 0.8
**6i**	7.8 ± 1.1	22.2 ± 1.1	25.3 ± 1.2	15.0 ± 0.4	50.0 ± 0.9	38.3 ± 0.8	10.6 ± 0.3	12.8 ± 1.5
**6j**	9.8 ± 2.0	22.2 ± 1.4	15.2 ± 1.5	20.0 ± 0.5	4.5 ± 2.0	1.7 ± 0.4	8.5 ± 1.5	12.8 ± 1.7
**6k**	29.4 ± 1.2	8.3 ± 2.0	41.8 ± 0.3	10.0 ± 0.8	18.2 ± 1.6	3.3 ± 0.7	10.6 ± 0.7	8.5 ± 0.6
**6l**	5.9 ± 0.3	19.4 ± 0.7	64.6 ± 2.0	10.0 ± 0.3	36.4 ± 0.6	21.7 ± 0.6	2.1 ± 2.1	12.8 ± 0.7
**6m**	9.8 ± 0.4	16.7 ± 1.2	79.7 ± 1.9	5.0 ± 1.2	50.0 ± 0.8	23.3 ± 0.8	4.3 ± 0.6	6.4 ± 2.1
**6n**	5.9 ± 0.2	27.8 ± 0.5	94.9 ± 0.2	15.0 ± 0.2	9.1 ± 0.5	38.3 ± 0.4	6.4 ± 0.8	14.9 ± 0.8
**6o**	7.8 ± 0.8	22.2 ± 0.8	86.1 ± 0.6	15.0 ± 0.1	36.4 ± 0.4	36.7 ± 0.6	4.3 ± 0.2	10.6 ± 0.2
**6p**	13.7 ± 0.6	27.8 ± 1.1	26.6 ± 0.8	15.0 ± 0.1	13.6 ± 0.2	38.3 ± 0.5	6.4 ± 0.6	10.6 ± 1.3
**6q**	5.9 ± 1.5	19.4 ± 0.9	22.8 ± 1.7	20.0 ± 2.0	40.9 ± 0.6	28.3 ± 1.2	12.8 ± 0.8	17.0 ± 1.5
**6r**	7.8 ± 1.0	22.2 ± 1.4	93.7 ± 2.3	15.0 ± 1.8	22.7 ± 0.5	23.3 ± 1.2	6.4 ± 0.6	12.8 ± 1.6
**6s**	7.8 ± 1.5	25.0 ± 2.0	69.6 ± 0.5	15.0 ± 0.4	36.4 ± 0.9	35.0 ± 1.1	8.5 ± 0.7	12.8 ± 1.7
**6t**	7.8 ± 2.1	19.4 ± 0.9	67.1 ± 0.9	10.0 ± 0.8	45.5 ± 0.3	13.3 ± 1.0	6.4 ± 0.6	10.6 ± 2.0
**6u**	7.8 ± 0.3	27.8 ± 0.6	22.8 ± 1.3	10.0 ± 1.1	31.8 ± 0.4	21.7 ± 0.2	0.0 ± 0.4	10.6 ± 0.9
**6v**	7.8 ± 0.6	19.4 ± 0.2	67.1 ± 0.3	15.0 ± 0.7	22.7 ± 1.1	26.7 ± 0.7	2.1 ± 1.8	4.3 ± 0.7
**6w**	0.0	13.9 ± 1.8	41.8 ± 1.4	15.0 ± 0.9	40.9 ± 1.3	20.0 ± 0.7	2.1 ± 1.6	6.4 ± 0.1
**6x**	7.8 ± 1.6	16.7 ± 0.4	21.5 ± 0.6	15.0 ± 1.8	40.9 ± 1.2	30.0 ± 0.7	0.0 ± 0.7	10.6 ± 0.3
**6y**	3.9 ± 2.3	16.7 ± 0.7	69.6 ± 0.4	15.0 ± 1.3	50.0 ± 2.0	46.7 ± 0.9	2.1 ± 0.6	12.8 ± 0.5
**6z**	2.0 ± 0.1	27.8 ± 0.1	24.1 ± 0.3	10.0 ± 0.7	50.0 ± 0.1	16.7 ± 0.6	12.8 ± 0.1	8.5 ± 0.6
**6aa**	9.8 ± 0.2	19.4 ± 0.8	11.4 ± 1.2	10.0 ± 1.4	40.9 ± 0.6	30.0 ± 0.4	14.9 ± 1.3	0.0
**6ab**	2.0 ± 1.1	16.7 ± 1.5	79.7 ± 1.6	5.0 ± 0.9	50.0 ± 0.4	40.0 ± 0.5	14.9 ± 1.6	17.0 ± 0.4
**6ac**	7.8 ± 1.5	22.2 ± 0.4	12.7 ± 1.7	5.0 ± 0.5	45.5 ± 0.3	11.7 ± 1.0	12.8 ± 1.2	12.8 ± 0.8
**6ad**	17.6 ± 2.0	47.2 ± 0.9	79.7 ± 0.6	35.0 ± 0.7	13.6 ± 0.2	65.0 ± 0.4	19.1 ± 0.6	23.4 ± 0.1
**6ae**	15.7 ± 0.3	19.4 ± 1.3	79.7 ± 0.5	40.0 ± 0.2	59.1 ± 0.1	87.0 ± 0.3	17.0 ± 0.4	12.8 ± 0.8
**6af**	9.8 ± 1.1	16.7 ± 1.2	3.8 ± 1.3	20.0 ± 1.1	22.7 ± 0.5	41.7 ± 0.5	4.3 ± 0.6	14.9 ± 0.2
**Boscalid** **^a^**	26.9 ± 1.3	82.4 ± 0.8	85.3 ± 1.5	65.9 ± 1.7	12 ± 0.6	46.5 ± 1.2	30.8 ± 1.2	25 ± 2.5

^a^: Positive control commercial sterilant. The experiment was repeated three times.

**Table 2:** EC_50_ values of compounds **6n**, **6r**, and **6o** against *P. p*.

Fungi	Compounds	EC_50_ (μg/mL)	Regression Equation	*R* ^2^
*P. p.*	**6n** (R = 2-CF_3_ Bn)	9.57	Y = 1.70x + 3.33	0.997
	**6r** (R = 3-OCF_3_ Bn)	11.80	y = 1.96x + 2.89	0.998
	**6o** (R = 3-CF_3_ Bn)	15.64	y = 1.97x + 2.65	0.997
	Boscalid	16.60	y = 1.84x + 3.18	0.996

**Table 3:** CoMFA statistical parameters of the 3D-QSAR model.

*q* ^2^	*r* ^2^	*S*	*F*	Contribution/%	
				Steric	Electrostatic
0.528	0.997	0.056	151.872	55.8%	44.2%

**Table 4:** SDH-inhibition activity of compounds **6o**, **6n** and **6r**.

Compounds	IC_50_ (μM)
**6o** (R = 3-CF_3_ Bn)	1.29
**6n** (R = 2-CF_3_ Bn)	2.09
**6r** (R = 3-OCF_3_ Bn)	2.34
Boscalid	7.02

## Data Availability

The original contributions presented in this study are included in the article/Supplementary Materials. Further inquiries can be directed to the corresponding authors.

## Supplementary Materials

The following supporting information can be downloaded at: https://www.mdpi.com/article/10.3390/molecules31214004/s1, Figures S1–S128: FT-IR, 1H-NMR, 13C-NMR, and HRMS spectra of compounds.
