# Supplementary material for: Synthesis, Antifungal Activity, and Molecular Simulation of Vanillin-Based 1,3,4-Oxadiazole-Thioether Derivatives as Potential Succinate Dehydrogenase Inhibitors
Source: Molecules. 2026 Nov 6;31(21):4004. doi: 10.3390/molecules31214004 (PMC13764773; doi:10.3390/molecules31214004)
Supplement: Supplementary file 1 [file molecules-31-04004-s001.zip › molecules-4605879-supplementary.pdf]

Synthesis, Antifungal Activity, and Molecular Simulation of Vanillin-Based 1,3,4-Oxadiazole-Thioether Derivatives as Potential Succinate Dehydrogenase Inhibitors

Table of Contents

1. Characterization data of the target compounds **6a-6af**
2. The FT-IR, ^{1}H NMR, ^{13}C NMR, and HRMS spectra of the target compounds **6a-6af**

1. Characterization data of the target compounds 6a-6af

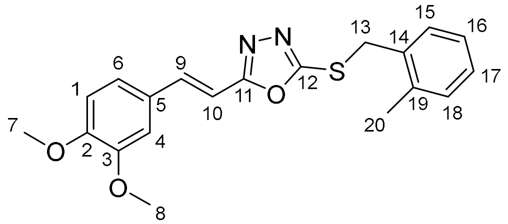

(E)-2-(3,4-dimethoxystyryl)-5-((2-methylbenzyl)thio)-1,3,4-oxadiazole(6a): White solid; yield, 85.2%; m.p. 103.5– 105.7°C; IR (KBr, cm^{-1}): 3011.32 (Ar-H, C=C-H), 2970.06, 2941.17, 2838.00 (C-H), 1633.04 (C=N, C=C), 1600.03, 1583.52, 1509.24, 1476.23 (Ar-C=C), 1253.39 (C-O-C) 727.25 (C-S-C). ^{1}H NMR (600 MHz, $CDCl_3$) δ 7.41 – 7.35 (m, 2H, Ar-H, C_9-H), 7.24 – 7.15 (m, 3H, Ar-H), 7.09 (dd, J = 8.3, 2.0 Hz, 1H,Ar-H), 7.05 (d, J = 2.0 Hz, 1H, Ar-H), 6.89 – 6.82 (m, 2H, Ar-H, C_{10}-H), 4.53 (s, 2H, C_{13}-H), 3.92 (s, 3H, C_7-H), 3.91 (s, 3H, C_8-H), 2.44 (s, 3H, C_{20}-H). ^{13}C NMR (151 MHz, $CDCl_3$) δ 165.69, 163.27, 150.87, 149.35, 138.55, 137.15, 133.00, 130.78, 130.31, 128.58, 127.77, 126.44, 121.86, 111.18, 109.09, 107.42, 56.01, 55.93, 35.16, 19.22. HRMS (APCI, m/z): calcd. for $C_{20}H_{20}N_2O_3S^+$ ($[M+H]^+$) 369.1267, found 369.1262.

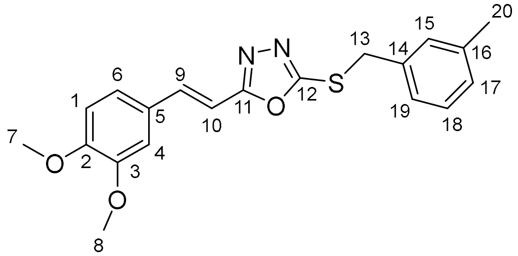

(E)-2-(3,4-dimethoxystyryl)-5-((3-methylbenzyl)thio)-1,3,4-oxadiazole(6b): White solid; yield, 90.2%; m.p. 95.1– 99.4°C; IR (KBr, cm^{-1}): 3055.91 (Ar-H, C=C-H), 2954.24, 2935.56, 2838.04 (C-H), 1655.33, 1638.73 (C=N, C=C),1603.45, 1586.86, 1514.24, 1474.81 (Ar-C=C), 1277.69 (C-O-C), 736.14 (C-S-C). ^{1}H NMR (600 MHz, $CDCl_3$) δ 7.37 (d, J = 16.3 Hz, 1H, C_9-H), 7.27 – 7.20 (m, 3H,Ar-H), 7.11 (d, J = 3.7 Hz, 1H, Ar-H), 7.08 (dd, J = 8.3, 2.0 Hz, 1H, Ar-H), 7.04 (d, J = 2.0 Hz, 1H, Ar-H), 6.87 (d, J = 8.3 Hz, 1H, Ar-H), 6.85 (d, J = 16.3 Hz, 1H, C_{10}-H), 4.47 (s, 2H, C_{13}-H), 3.92 (s, 3H, C_7-H), 3.91 (s, 3H, C_8-H), 2.34 (s, 3H, C_{20}-H). ^{13}C NMR (151 MHz, $CDCl_3$) δ 165.68, 163.25, 150.87, 149.36, 138.58, 138.51, 135.37, 129.83, 128.91, 128.73,

127.78, 126.18, 121.84, 111.20, 109.11, 107.43, 56.01, 55.93, 36.84, 21.35. HRMS (APCI, m/z): calcd. for $C_{20}H_{20}N_2O_3S^+$ ($[M+H]^+$) 369.1267, found 369.1263.

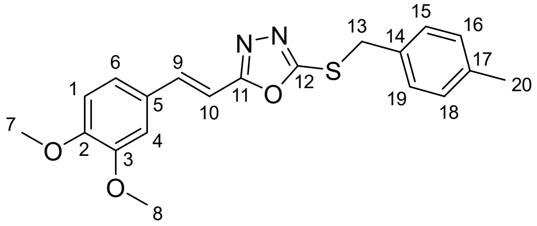

(E)-2-(3,4-dimethoxystyryl)-5-((4-methylbenzyl)thio)-1,3,4-oxadiazole(6c): White solid; yield, 88.6%; m.p.88.4 – 110.8 °C; IR (KBr, cm^{-1}): 3077.82 (Ar-H, C=C-H), 2997.36, 2949.91, 2824.07 (C-H), 1639.23 (C=N, C=C), 1597.97, 1509.24, 1474.17 (Ar-C=C), 1278.16 (C-O-C), 707.62 (C-S-C). ^1H NMR (600 MHz, $CDCl_3$) δ 7.36 (d, J = 16.4 Hz, 1H, C_9-H), 7.32 (d, J = 7.9 Hz, 2H, Ar-H), 7.14 (d, J = 7.8 Hz, 2H, Ar-H), 7.08 (dd, J = 8.3, 2.1 Hz, 1H Ar-H), 7.04 (d, J = 2.1 Hz, 1H, Ar-H), 6.87 (d, J = 8.3 Hz, 1H, Ar-H), 6.84 (d, J = 16.3 Hz, 1H, C_{10}-H), 4.46 (s, 2H, C_{13}-H), 3.92 (s, 3H, C_7-H), 3.90 (s, 3H, C_8-H), 2.32 (s, 3H, C_{20}-H). ^{13}C NMR (151 MHz, $CDCl_3$) δ 165.66, 163.28, 150.85, 149.34, 138.52, 137.97, 132.42, 129.51, 129.07, 127.77, 121.85, 111.18, 109.09, 107.41, 56.01, 55.93, 36.65, 21.18. HRMS (APCI, m/z): calcd. for $C_{20}H_{20}N_2O_3S^+$ ($[M+H]^+$) 369.1267, found 369.1262.

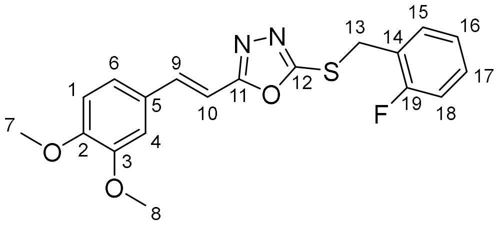

(E)-2-(3,4-dimethoxystyryl)-5-((2-fluorobenzyl)thio)-1,3,4-oxadiazole(6d): White solid; yield, 78.2%; m.p. 91.5 – 93.4 °C; IR (KBr, cm^{-1}): 3048.46 (Ar-H, C=C-H), 2976.25, 2947.36, 2835.94 (C-H), 1635.10 (C=N, C=C), 1600.03, 1585.58, 1509.24, 1478.29 (Ar-C=C), 1276.09 (C-O-C), 677.74 (C-S-C). ^1H NMR (600 MHz, $CDCl_3$) δ 7.51 (td, J = 7.6, 1.8 Hz, 1H,Ar-H), 7.37 (d, J = 16.3 Hz, 1H, C9-H), 7.30 – 7.27 (m, 1H, Ar-H), 7.12 – 7.03 (m, 4H, Ar-H), 6.87 (d, J = 8.3 Hz, 1H, Ar-H), 6.83 (d, J = 16.3 Hz, 1H, C_{10}-H), 4.52 (s, 2H, C_{13}-H), 3.91 (s, 3H, C_7-H), 3.90 (s, 3H, C_8-H). ^{13}C NMR (151 MHz, $CDCl_3$) δ 165.84, 162.90, 161.79, 160.15, 150.86, 149.33, 138.63, 131.40,

131.38, 130.11, 130.06, 127.72, 124.38, 124.35, 123.18, 123.08, 121.87, 115.69, 115.55, 111.16, 109.07, 107.34, 56.00, 55.92, 30.07, 30.05. HRMS (APCI, m/z): calcd. for C19H17FN2O3S$^+$ ([M+H]$^+$) 373.1017, found 373.1011.

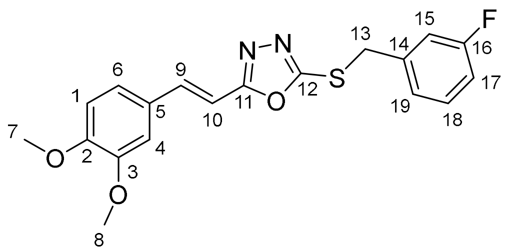

(E)-2-(3,4-dimethoxystyryl)-5-((3-fluorobenzyl)thio)-1,3,4-oxadiazole(6e):White solid; yield, 89.4%; m.p. 94.8 – 98.1 °C; IR (KBr, cm^{-1}): 3056.71 (Ar-H, C=C-H), 2978.31, 2943.23, 2842.13 (C-H), 1653.68,1637.17 (C=N, C=C), 1597.97, 1581.46, 1517.50, 1474.17 (Ar-C=C), 1276.09 (C-O-C), 708.69 (C-S-C). ^{1}H NMR (600 MHz, $CDCl_3$) δ 7.36 (d, J = 16.3 Hz, 1H, C_9-H), 7.29 (td, J = 8.0, 5.8 Hz, 1H, Ar-H), 7.22 (d, J = 7.8 Hz, 1H, Ar-H), 7.17 (dt, J = 9.5, 2.1 Hz, 1H, Ar-H), 7.08 (dd, J = 8.3, 2.0 Hz, 1H, Ar-H), 7.04 (d, J = 2.0 Hz, 1H, Ar-H), 6.99 (td, J = 8.4, 2.6 Hz, 1H, Ar-H), 6.87 (d, J = 8.3 Hz, 1H, Ar-H), 6.83 (d, J = 16.3 Hz, 1H, C_{10}-H), 4.47 (s, 2H, C_{13}-H), 3.92 (s, 3H, C_7-H), 3.90 (s, 3H, C_8-H). 13C NMR (151 MHz, $CDCl_3$) δ 165.85, 162.73, 150.90, 149.35, 138.71, 130.37, 130.31, 127.70, 124.83, 124.81, 121.87, 116.18, 116.03, 115.20, 115.06, 111.18, 109.10, 107.30, 56.01, 55.92, 36.16, 36.14. HRMS (APCI, m/z): calcd. for $C_{19}H_{17}FN_2O_3S^+$ ([M+H]$^+$) 373.1017, found 373.1011.

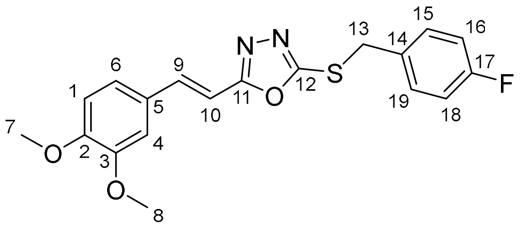

(E)-2-(3,4-dimethoxystyryl)-5-((4-fluorobenzyl)thio)-1,3,4-oxadiazole(6f): White solid; yield, 74.1%; m.p. 101.2 – 103.5 °C; IR (KBr, cm^{-1}): 3089.73(Ar-H, C=C-H), 2965.93, 2934.98, 2838.00 (C-H), 1635.10 (C=N, C=C), 1602.09, 1581.46, 1513.37, 1472.10 (Ar-C=C), 1278.15 (C-O-C), 733.44 (C-S-C). ^{1}H NMR (600 MHz, $CDCl_3$) δ 7.43 (d, J = 5.3 Hz, 1H,Ar-H), 7.41 (d, J = 5.3 Hz, 1H,Ar-H), 7.36 (d, J = 16.4 Hz, 1H,

C9-H), 7.08 (dd, J = 8.3, 2.0 Hz, 1H,Ar-H), 7.04 (d, J = 2.0 Hz, 1H,Ar-H), 7.02 (t, J = 8.6 Hz, 2H,Ar-H), 6.87 (d, J = 8.3 Hz, 1H,Ar-H), 6.84 (d, J = 16.3 Hz, 1H, C_{10}-H), 4.46 (s, 2H, C_{13}-H), 3.92 (s, 3H, C_7-H), 3.91 (s, 3H, C_8-H). ^{13}C NMR (151 MHz, $CDCl_3$) δ 165.80, 162.90, 162.48 (d, J = 247.5 Hz), 150.90, 149.35, 138.67, 131.49 (d, J = 3.3 Hz), 130.91 (d, J = 8.3 Hz), 127.71, 121.87, 115.75 (d, J = 21.6 Hz), 111.18, 109.09, 107.32, 56.01, 55.93, 36.02. HRMS (APCI, m/z): calcd. for $C_{19}H_{17}FN_2O_3S^+$ ($[M+H]^+$) 373.1017, found 373.1012.

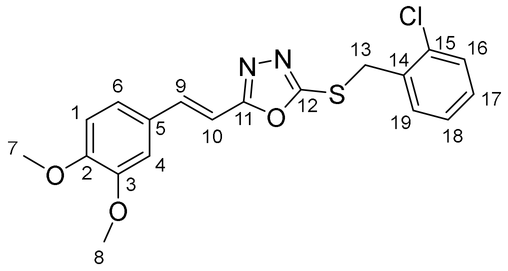

(E)-2-((2-chlorobenzyl)thio)-5-(3,4-dimethoxystyryl)-1,3,4-oxadiazole(6g): White solid; yield, 88.6%; m.p. 94.5– 97.6 °C; IR (KBr, cm^{-1}): 3055.13(Ar-H, C=C-H), 2968.48, 2941.66, 2836.45 (C-H), 1635.76 (C=N, C=C), 1600.69, 1586.25, 1514.04, 1478.97 (Ar-C=C), 1280.92 (C-O-C), 732.15 (C-S-C). ^{1}H NMR (600 MHz, $CDCl_3$) δ 7.59 (dd, J = 7.2, 2.1 Hz, 1H,Ar-H), 7.41 – 7.38 (m, 1H, Ar-H), 7.36 (d, J = 16.3 Hz, 1H, C_9-H), 7.23 (dtd, J = 15.1, 7.5, 1.8 Hz, 2H, Ar-H), 7.07 (dd, J = 8.3, 2.2 Hz, 1H, Ar-H), 7.04 (d, J = 2.0 Hz, 1H, Ar-H), 6.86 (d, J = 7.7 Hz, 1H, Ar-H), 6.83 (d, J = 16.4 Hz, 1H, C_{10}-H), 4.59 (s, 2H, C_{13}-H), 3.91 (s, 3H, C_7-H), 3.90 (s, 3H, C_8-H). ^{13}C NMR (151 MHz, $CDCl_3$) δ 165.85, 163.07, 150.87, 149.34, 138.62, 134.36, 133.71, 131.50, 129.79, 129.64, 127.73, 127.13, 121.88, 111.17, 109.08, 107.34, 56.01, 55.93, 34.56. HRMS (APCI, m/z): calcd. for $C_{19}H_{17}ClN_2O_3S^+$ ($[M+H]^+$) 389.0721, found 389.0715.

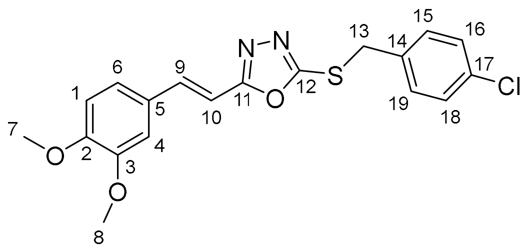

(E)-2-((4-chlorobenzyl)thio)-5-(3,4-dimethoxystyryl)-1,3,4-oxadiazole(6h): White solid; yield, 81.6%; m.p. 108.7– 112.8 °C; IR (KBr, cm^{-1}):IR (KBr, cm-1): 3067.51 (Ar-

H, C=C-H), 2968.48, 2949.91, 2830.26 (C-H), 1641.95 (C=N, C=C), 1604.81, 1588.31, 1514.04, 1476.91 (Ar-C=C), 1268.54 (C-O-C), 728.02 (C-S-C). ^{1}H NMR (600 MHz, $CDCl_3$) δ 7.38 (d, J = 8.4 Hz, 2H,Ar-H), 7.35 (d, J = 16.4 Hz, 1H, C_9-H), 7.32 – 7.28 (m, 2H, Ar-H), 7.08 (dd, J = 8.3, 2.0 Hz, 1H, Ar-H), 7.04 (d, J = 2.0 Hz, 1H, Ar-H), 6.87 (d, J = 8.3 Hz, 1H, Ar-H), 6.83 (d, J = 16.3 Hz, 1H, C_{10}-H), 4.44 (s, 2H, C_{13}-H), 3.92 (s, 3H, C_7-H), 3.90 (s, 3H, C_8-H). ^{13}C NMR (151 MHz, $CDCl_3$) δ 165.84, 162.75, 150.90, 149.34, 138.72, 134.30, 134.01, 130.53, 128.96, 127.68, 121.89, 111.17, 109.10, 107.27, 56.01, 55.93, 36.01. HRMS (APCI, m/z): calcd. for $C_{19}H_{17}ClN_2O_3S^+$ ($[M+H^{=}]^{+}$) 389.0721, found 389.0716.

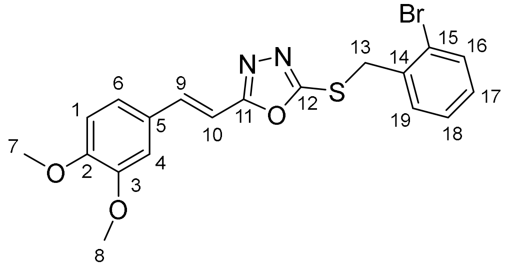

(E)-2-((2-bromobenzyl)thio)-5-(3,4-dimethoxystyryl)-1,3,4-oxadiazole(6i): White solid; yield, 83.2%; m.p. 96.8– 100.1°C; IR (KBr, cm^{-1}): 3057.19 (Ar-H, C=C-H), 2966.42, 2945.79, 2836.45 (C-H), 1633.70 (C=N, C=C), 1598.62, 1582.12, 1518.17, 1483.09 (Ar-C=C), 1276.79 (C-O-C), 674.38 (C-S-C). ^{1}H NMR (600 MHz, $CDCl_3$) δ 7.61 (dd, J = 7.6, 1.7 Hz, 1H, Ar-H), 7.57 (dd, J = 8.0, 1.3 Hz, 1H, Ar-H), 7.36 (d, J = 16.3 Hz, 1H, C_9-H), 7.28 – 7.24 (m, 1H, Ar-H), 7.15 (td, J = 7.7, 1.7 Hz, 1H, Ar-H), 7.07 (dd, J = 8.3, 2.0 Hz, 1H, Ar-H), 7.04 (d, J = 2.0 Hz, 1H, Ar-H), 6.86 (d, J = 8.3 Hz, 1H, Ar-H), 6.83 (d, J = 16.3 Hz, 1H, C_{10}-H), 4.60 (s, 2H, C_{13}-H), 3.91 (s, 3H, C_7-H), 3.90 (s, 3H, C_8-H). ^{13}C NMR (151 MHz, $CDCl_3$) δ 165.84, 163.00, 150.87, 149.34, 138.61, 135.42, 133.09, 131.55, 129.79, 127.76, 127.73, 124.73, 121.86, 111.18, 109.11, 107.35, 56.00, 55.93, 37.21. HRMS (APCI, m/z): calcd. for $C_{19}H_{17}BrN_2O_3S^+$ ($[M+H]^{+}$) 433.0216, found 433.0210.

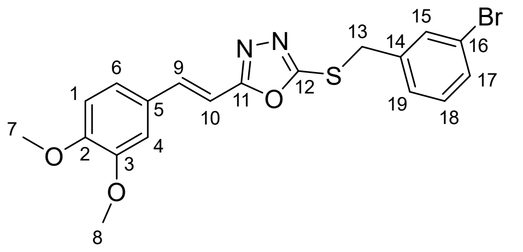

(E)-2-((3-bromobenzyl)thio)-5-(3,4-dimethoxystyryl)-1,3,4-oxadiazole(6j): White solid; yield, 78.9%; m.p. 97.8– 101.3 °C; IR (KBr, cm^{-1}): 3062.96 (Ar-H, C=C-H), 2964.87, 2935.65, 2835.48 (C-H), 1645.91 (C=N, C=C), 1597.91, 1585.39, 1510.26, 1474.78 (Ar-C=C), 1280.70 (C-O-C), 736.00 (C-S-C). ^{1}H NMR (600 MHz, $CDCl_3$) *δ* 7.60 (t, *J* = 1.9 Hz, 1H,Ar-H), 7.42 (d, *J* = 7.9 Hz, 1H, Ar-H), 7.40 – 7.33 (m, 2H, Ar-H, C_9-H), 7.20 (t, *J* = 7.9 Hz, 1H, Ar-H), 7.08 (dd, *J* = 8.4, 2.0 Hz, 1H, Ar-H), 7.04 (d, *J* = 2.0 Hz, 1H, Ar-H), 6.87 (d, *J* = 8.3 Hz, 1H, Ar-H), 6.83 (d, *J* = 16.3 Hz, 1H, C_{10}-H), 4.43 (s, 2H, C_{13}-H), 3.92 (s, 3H, C_7-H), 3.90 (s, 3H, C_8-H). ^{13}C NMR (151 MHz, $CDCl_3$) *δ* 165.86, 162.64, 150.90, 149.35, 138.73, 138.03, 132.09, 131.24, 130.34, 127.82, 127.70, 122.67, 121.89, 111.18, 109.12, 107.29, 56.01, 55.94, 35.99. HRMS (APCI, m/z): calcd. for $C_{19}H_{17}BrN_2O_3S^+$ ($[M+H]^+$) 433.0216, found 433.0212.

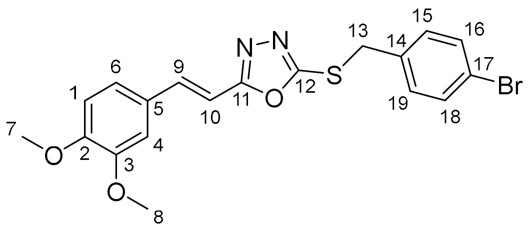

(E)-2-((4-bromobenzyl)thio)-5-(3,4-dimethoxystyryl)-1,3,4-oxadiazole(6k): White solid; yield, 83.7%; m.p. 101.7 – 104.8 °C; IR (KBr, cm^{-1}): 3014.96 (Ar-H, C=C-H), 2956.52, 2929.39, 2837.57(C-H), 1633.39 (C=N, C=C), 1600.00, 1583.30, 1518.61, 1481.04 (Ar-C=C), 1274.43 (C-O-C), 723.48 (C-S-C). ^{1}H NMR (600 MHz, $CDCl_3$) *δ* 7.46 – 7.43 (m, 2H,Ar-H), 7.35 (d, *J* = 16.3 Hz, 1H, C_9-H), 7.32 (d, *J* = 2.0 Hz, 1H, Ar-H), 7.31 (d, *J* = 2.0 Hz, 1H, Ar-H), 7.07 (dd, *J* = 8.3, 2.0 Hz, 1H, Ar-H), 7.03 (d, *J* = 2.0 Hz, 1H, Ar-H), 6.86 (d, *J* = 8.3 Hz, 1H, Ar-H), 6.82 (d, *J* = 16.3 Hz, 1H, C_{10}-H), 4.42 (s, 2H, C_{13}-H), 3.91 (s, 3H, C_7-H), 3.90 (s, 3H, C_8-H). ^{13}C NMR (151 MHz, $CDCl_3$) *δ* 165.85, 162.72, 150.91, 149.35, 138.73, 134.83, 131.93, 130.85, 127.69, 122.14, 121.90, 111.18, 109.10, 107.27, 56.01, 55.94, 36.06. HRMS (APCI, m/z): calcd. for

$C_{19}H_{17}BrN_2O_3S^+$ ([M+H]$^+$) 433.0216, found 433.0211.

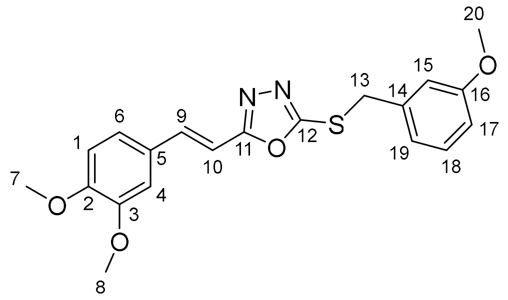

(E)-2-(3,4-dimethoxystyryl)-5-((3-methoxybenzyl)thio)-1,3,4-oxadiazole(6l):

White solid; yield, 83.1%; m.p. 105.1–109.2 °C; IR (KBr, cm^{-1}): 3058.78(Ar-H, C=C-H), 2944.00, 2921.04, 2833.39(C-H), 1652.17, 1637.57 (C=N, C=C),1600.00, 1585.39, 1516.52, 1474.78 (Ar-C=C), 1274.43 (C-O-C), 713.04 (C-S-C). ^{1}H NMR (600 MHz, $CDCl_3$) δ 7.36 (d, J = 16.3 Hz, 1H, C_9-H), 7.24 (t, J = 7.9 Hz, 1H, Ar-H), 7.08 (dd, J = 8.3, 2.0 Hz, 1H, Ar-H), 7.04 (d, J = 2.0 Hz, 1H, Ar-H), 7.01 (d, J = 7.6 Hz, 1H, Ar-H), 6.98 (s, 1H, Ar-H), 6.87 (d, J = 8.3 Hz, 1H, Ar-H), 6.85 – 6.82 (m, 2H, Ar-H, C_{10}-H), 4.47 (s, 2H, C_{13}-H), 3.91 (s, 3H, C_7-H), 3.90 (s, 3H,C_8-H), 3.79 (s, 3H, C_{20}-H). ^{13}C NMR (151 MHz, $CDCl_3$) δ 165.72, 163.14, 159.84, 150.86, 149.34, 138.57, 137.00, 129.87, 127.75, 121.85, 121.39, 114.62, 113.76, 111.18, 109.10, 107.38, 56.00, 55.92, 55.28, 36.83. HRMS (APCI, m/z): calcd. for $C_{20}H_{20}N_2O_4S^+$ ([M+H]$^+$) 385.1217, found 385.1210.

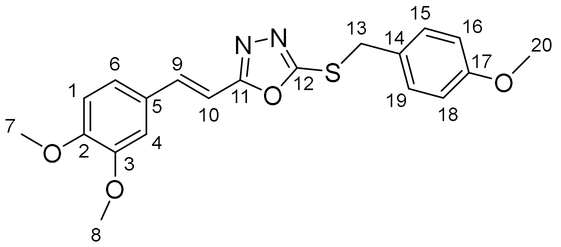

(E)-2-(3,4-dimethoxystyryl)-5-((4-methoxybenzyl)thio)-1,3,4-oxadiazole(6m):

White solid; yield, 81.6%; m.p. 72.7– 75.2°C; IR (KBr, cm^{-1}): 3055.91(Ar-H, C=C-H), 2999.88, 2941.79, 2833.89 (C-H), 1642.88 (C=N, C=C), 1600.38, 1584.78, 1510.09, 1470.66 (Ar-C=C), 1265.24 (C-O-C), 731.99 (C-S-C). ^{1}H NMR (600 MHz, $CDCl_3$) δ 7.38 – 7.33 (m, 3H,Ar-H, C_9-H), 7.07 (dd, J = 8.3, 2.0 Hz, 1H, Ar-H), 7.04 (d, J = 2.0 Hz, 1H, Ar-H), 6.88 – 6.85 (m, 2H, Ar-H), 6.85 – 6.81 (m, 2H, Ar-H, C_{10}-H), 4.45 (s,

2H, C_{13}-H), 3.91 (s, 3H, C_7-H), 3.90 (s, 3H, C_8-H), 3.77 (s, 3H, C_{20}-H). ^{13}C NMR (151 MHz, $CDCl_3$) δ 165.65, 163.27, 159.45, 150.86, 149.35, 138.53, 130.42, 127.77, 127.42, 121.83, 114.22, 111.20, 109.14, 107.41, 56.00, 55.93, 55.30, 36.50. HRMS (APCI, m/z): calcd. for $C_{20}H_{20}N_2O_4S^+$ ([M+H]$^+$) 385.1217, found 385.1211.

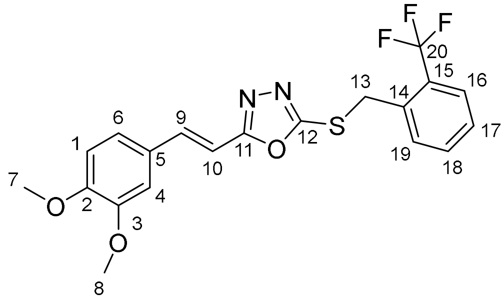

(E)-2-(3,4-dimethoxystyryl)-5-((2-(trifluoromethyl)benzyl)thio)-1,3,4-oxadiazole (6n): White solid; yield, 82.1%; m.p. 103.8 – 107.2 °C; IR (KBr, cm^{-1}): 3065.44(Ar-H, C=C-H), 2960.23, 2929.28, 2836.45 (C-H), 1648.14 (C=N, C=C), 1602.75, 1586.25, 1514.04, 1467.98 (Ar-C=C), 1276.09 (C-O-C), 681.86 (C-S-C). ^{1}H NMR (600 MHz, $CDCl_3$) δ 7.77 (d, J = 7.7 Hz, 1H, Ar-H), 7.67 (d, J = 7.9 Hz, 1H, Ar-H), 7.52 (t, J = 7.0 Hz, 1H, Ar-H), 7.41 (t, J = 7.7 Hz, 1H, Ar-H), 7.37 (d, J = 16.3 Hz, 1H, C_9-H), 7.08 (dd, J = 8.3, 2.0 Hz, 1H, Ar-H), 7.05 (d, J = 2.0 Hz, 1H, Ar-H), 6.87 (d, J = 8.3 Hz, 1H, Ar-H), 6.85 (d, J = 16.3 Hz, 1H, C_{10}-H), 4.69 (s, 2H, C_{13}-H), 3.92 (s, 3H, C_7-H), 3.91 (s, 3H, C_8-H). ^{13}C NMR (151 MHz, $CDCl_3$) δ 165.95, 163.06, 150.91, 149.36, 138.74, 134.48, 132.42, 132.05, 128.34, 127.70, 126.39 (q, J = 5.7 Hz), 125.13, 123.31, 121.90, 111.17, 109.09, 107.28, 56.00, 55.92, 33.18 (d, J = 2.0 Hz). HRMS (APCI, m/z): calcd. For $C_{20}H_{17}F_3N_2O_3S^+$ ([M+H]$^+$) 423.0985, found 423.0976.

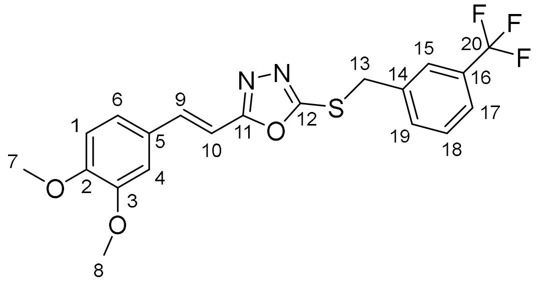

(E)-2-(3,4-dimethoxystyryl)-5-((3-(trifluoromethyl)benzyl)thio)-1,3,4-oxadiazole (6o): White solid; yield, 84.7%; m.p. 82.5–85.3 °C; IR (KBr, cm^{-1}): 3057.98 (Ar-H,

C=C-H), 2962.54, 2937.64, 2838.04 (C-H), 1640.81 (C=N, C=C), 1601.38, 1586.86, 1514.24, 1474.81 (Ar-C=C), 1275.62 (C-O-C), 702.94 (C-S-C). ^{1}H NMR (600 MHz, $CDCl_3$) δ 7.71 (s, 1H,Ar-H), 7.66 (d, J = 7.8 Hz, 1H, Ar-H), 7.55 (d, J = 7.9 Hz, 1H, Ar-H), 7.45 (t, J = 7.8 Hz, 1H, Ar-H), 7.36 (d, J = 16.4 Hz, 1H, C_9-H), 7.07 (dd, J = 8.3, 2.0 Hz, 1H, Ar-H), 7.03 (d, J = 2.0 Hz, 1H, Ar-H), 6.87 (d, J = 8.3 Hz, 1H, Ar-H), 6.82 (d, J = 16.3 Hz, 1H, C_{10}-H), 4.52 (s, 2H, C_{13}-H), 3.91 (s, 3H, C_7-H), 3.90 (s, 3H, C_8-H). ^{13}C NMR (151 MHz, $CDCl_3$) δ 165.93, 162.49, 150.92, 149.35, 138.78, 136.92, 132.59, 131.15 (q, J = 32.4 Hz), 129.32, 127.67, 125.86 (q, J = 3.8 Hz), 124.93 (q, J = 3.8 Hz), 122.97, 121.87, 111.17, 109.11, 107.23, 55.98, 55.91, 36.07. HRMS (APCI, m/z): calcd. For $C_{20}H_{17}F_3N_2O_3S^+$ ([M+H]$^+$) 423.0985, found 423.0977.

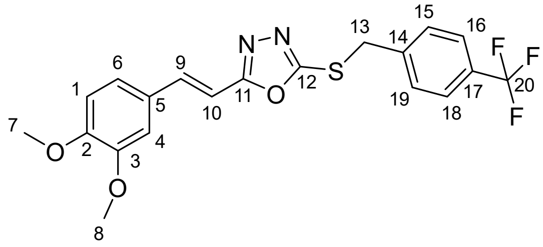

(E)-2-(3,4-dimethoxystyryl)-5-((4-(trifluoromethyl)benzyl)thio)-1,3,4-oxadiazole (6p): White solid; yield, 86.5%; m.p. 93.1 – 96.8 °C; IR (KBr, cm^{-1}): 3057.19(Ar-H, C=C-H), 2956.10, 2939.60, 2834.38 (C-H), 1652.26 (C=N, C=C), 1600.69, 1584.18, 1509.91, 1470.72 (Ar-C=C), 1262.35 (C-O-C), 725.96 (C-S-C).^{1}H NMR (600 MHz, $CDCl_3$) δ 7.59 (d, J = 2.1 Hz, 4H, Ar-H), 7.36 (d, J = 16.3 Hz, 1H, C_9-H), 7.08 (dd, J = 8.3, 2.0 Hz, 1H, Ar-H), 7.04 (d, J = 2.0 Hz, 1H, Ar-H), 6.87 (d, J = 8.3 Hz, 1H, Ar-H), 6.83 (d, J = 16.3 Hz, 1H, C_{10}-H), 4.51 (s, 2H, C_{13}-H), 3.92 (s, 3H, C_7-H), 3.91 (s, 3H, C_8-H). ^{13}C NMR (151 MHz, $CDCl_3$) δ 165.95, 162.50, 150.94, 149.36, 139.96, 138.81, 130.27 (q, J = 32.4 Hz), 129.52, 127.66, 125.74 (q, J = 3.8 Hz), 121.90, 111.17, 109.09, 107.23, 56.00, 55.92, 35.98. HRMS (APCI, m/z): calcd. For $C_{20}H_{17}F_3N_2O_3S^+$ ([M+H]$^+$) 423.0985, found 423.0977.

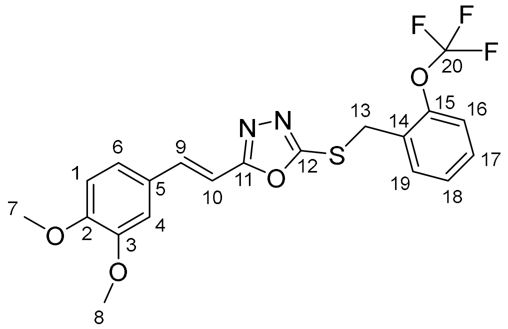

(E)-2-(3,4-dimethoxystyryl)-5-((2-(trifluoromethoxy)benzyl)thio)-1,3,4-oxadiazole (6q): White solid; yield, 79.1%; m.p. 84.5– 87.3°C; IR (KBr, cm^{-1}): 3057.98 (Ar-H, C=C-H), 2987.44, 2960.46, 2838.04 (C-H), 1638.73 (C=N, C=C), 1601.38, 1580.63, 1516.31, 1476.89 (Ar-C=C), 1265.24(C-O-C), 698.79(C-S-C). ^{1}H NMR (600 MHz, $CDCl_3$) δ 7.63 (dd, J = 7.6, 1.7 Hz, 1H,Ar-H), 7.37 (d, J = 16.4 Hz, 1H, C_9-H), 7.34 (dd, J = 8.3, 7.3, 1.7 Hz, 1H, Ar-H), 7.29 – 7.23 (m, 2H, Ar-H), 7.08 (dd, J = 8.3, 2.0 Hz, 1H, Ar-H), 7.04 (d, J = 2.0 Hz, 1H, Ar-H), 6.87 (d, J = 8.3 Hz, 1H, Ar-H), 6.84 (d, J = 16.4 Hz, 1H, C_{10}-H), 4.54 (s, 2H, C_{13}-H), 3.92 (s, 3H, C_7-H), 3.91 (s, 3H, C_8-H). ^{13}C NMR (151 MHz, $CDCl_3$) δ 165.91, 162.86, 150.91, 149.37, 147.60, 138.69, 131.68, 129.74, 128.37, 127.74, 126.92, 122.47 – 118.69 (q), 111.20, 109.12, 107.32, 56.00, 55.92, 30.93. HRMS (APCI, m/z): calcd. for $C_{20}H_{17}F_3N_2O_4S^+$ ($[M+H]^+$) 439.0934, found 439.0927.

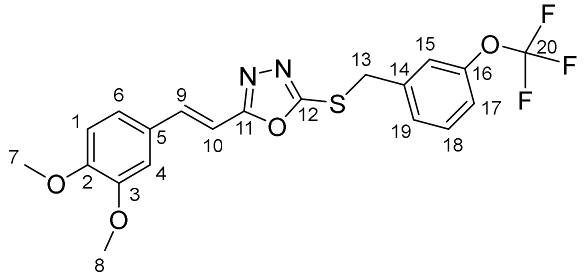

(E)-2-(3,4-dimethoxystyryl)-5-((3-(trifluoromethoxy)benzyl)thio)-1,3,4-oxadiazole (6r): White solid; yield, 80.2%; m.p. 70.3– 72.9°C; IR (KBr, cm^{-1}): 3033.74 (Ar-H, C=C-H), 2977.39, 2948.17, 2843.83 (C-H), 1652.17, 1633.39(C=N, C=C), 1602.09, 1587.48, 1518.61, 1476.87 (Ar-C=C), 1249.39 (C-O-C), 698.43 (C-S-C). ^{1}H NMR (600 MHz, $CDCl_3$) δ 7.41 – 7.36 (m, 2H, Ar-H, C_9-H), 7.35 (d, J = 2.9 Hz, 1H, Ar-H), 7.33 (d, J = 14.7 Hz, 1H, Ar-H), 7.15 (d, J = 8.0 Hz, 1H, Ar-H), 7.08 (dd, J =

8.4, 2.1 Hz, 1H, Ar-H), 7.04 (d, *J* = 2.1 Hz, 1H, Ar-H), 6.87 (d, *J* = 8.3 Hz, 1H, Ar-H), 6.83 (d, *J* = 16.4 Hz, 1H, C_{10}-H), 4.48 (s, 2H, C_{13}-H), 3.92 (s, 3H, C_7-H), 3.90 (s, 3H, C_8-H). ^{13}C NMR (151 MHz, $CDCl_3$) δ 165.90, 162.56, 150.93, 149.38 (d, *J* = 4.5 Hz), 138.75, 138.10, 130.18, 127.70, 127.53, 121.87, 121.72 – 119.30 (q), 111.19, 109.12, 107.27, 56.00, 55.91, 36.03. HRMS (APCI, m/z): calcd. for C20H17F3N2O4S$^+$ ([M+H]$^+$) 439.0934, found 439.0926.

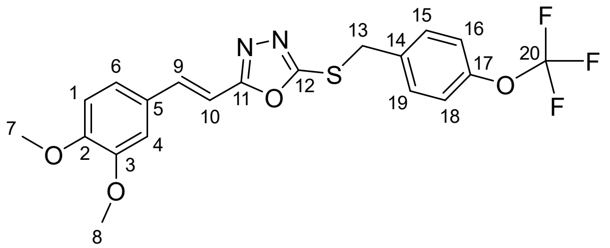

(E)-2-(3,4-dimethoxystyryl)-5-((4-(trifluoromethoxy)benzyl)thio)-1,3,4-oxadiazole (6s): White solid; yield, 76.5%; m.p. 78.9– 82.3°C; IR (KBr, cm^{-1}): 3048.94 (Ar-H, C=C-H), 2958.17, 2931.35, 2836.45 (C-H), 1637.82 (C=N, C=C), 1600.69, 1586.25, 1518.17, 1478.97 (Ar-C=C), 1252.03 (C-O-C), 709.46 (C-S-C). ^{1}H NMR (600 MHz, $CDCl_3$) δ 7.48 (d, *J* = 8.6 Hz, 2H,Ar-H), 7.36 (d, *J* = 16.3 Hz, 1H, C_9-H), 7.17 (d, *J* = 7.7 Hz, 2H, Ar-H), 7.08 (dd, *J* = 8.3, 2.0 Hz, 1H, Ar-H), 7.04 (d, *J* = 2.0 Hz, 1H, Ar-H), 6.87 (d, *J* = 8.3 Hz, 1H, Ar-H), 6.83 (d, *J* = 16.4 Hz, 1H, C_{10}-H), 4.48 (s, 2H, C_{13}-H), 3.91 (s, 3H, C_7-H), 3.90 (s, 3H, C_8-H). ^{13}C NMR (151 MHz, $CDCl_3$) δ 165.87, 162.71, 150.93, 149.15 (d, *J* = 67.3 Hz), 138.73, 134.54, 130.65, 127.69, 121.87, 121.21, 111.19, 109.12, 107.28, 55.99, 55.91, 35.82. HRMS (APCI, m/z): calcd. for $C_{20}H_{17}F_3N_2O_4S^+$ ([M+H]$^+$) 439.0934, found 439.0928.

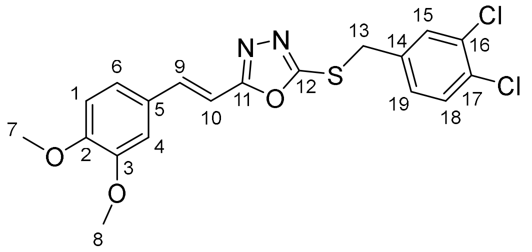

(E)-2-((3,4-dichlorobenzyl)thio)-5-(3,4-dimethoxystyryl)-1,3,4-oxadiazole (6t): White solid; yield, 75.8%; m.p. 110.1– 118.7 °C ;IR (KBr, cm^{-1}): 3048.94 (Ar-H, C=C-H), 2960.23, 2939.60, 2830.26 (C-H), 1639.89 (C=N, C=C), 1600.69, 1588.31, 1514.04,

1472.78 (Ar-C=C), 1270.60 (C-O-C), 740.40 (C-S-C). ^{1}H NMR (600 MHz, $CDCl_3$) δ 7.55 (d, J = 2.1 Hz, 1H,Ar-H), 7.39 (d, J = 8.3 Hz, 1H Ar-H), 7.36 (d, J = 16.3 Hz, 1H, C_9-H), 7.30 (dd, J = 8.3, 2.1 Hz, 1H, Ar-H), 7.08 (dd, J = 8.3, 2.0 Hz, 1H, Ar-H), 7.04 (d, J = 2.0 Hz, 1H, Ar-H), 6.87 (d, J = 8.3 Hz, 1H, Ar-H), 6.82 (d, J = 16.3 Hz, 1H, C_{10}-H), 4.41 (s, 2H, C_{13}-H) 3.92 (s, 3H, C_7-H), 3.91 (s, 3H, C_8-H). ^{13}C NMR (151 MHz, $CDCl_3$) δ 165.96, 162.38, 150.94, 149.35, 138.84, 136.16, 132.77, 132.29, 131.02, 130.71, 128.54, 127.65, 121.90, 111.18, 109.12, 107.21, 56.01, 55.94, 35.42. HRMS (APCI, m/z): calcd. for $C_{19}H_{16}Cl_2N_2O_3S^+$ ($[M+H]^+$) 423.0331, found 423.0325.

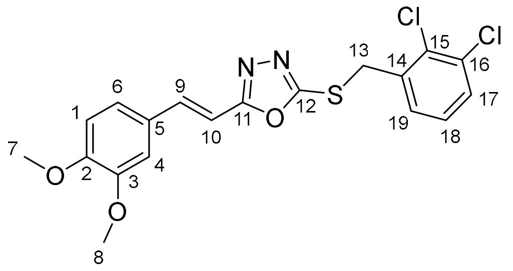

(E)-2-((2,3-dichlorobenzyl)thio)-5-(3,4-dimethoxystyryl)-1,3,4-oxadiazole(6u):

White solid; yield, 75.8%; m.p. 118.1– 122.3 °C; IR (KBr, cm^{-1}): 3076.38 (Ar-H, C=C-H), 2994.52, 2939.94, 2837.08 (C-H), 1634.29 (C=N, C=C), 1600.80, 1588.10, 1516.73, 1472.65 (Ar-C=C), 1273.24 (C-O-C), 725.36 (C-S-C). ^{1}H NMR (600 MHz, $CDCl_3$) δ 7.54 (dd, J = 7.7, 1.6 Hz, 1H,Ar-H), 7.40 (dd, J = 8.1, 1.6 Hz, 1H, Ar-H), 7.35 (d, J = 16.3 Hz, 1H, C_9-H), 7.15 (t, J = 7.9 Hz, 1H, Ar-H), 7.07 (dd, J = 8.3, 2.0 Hz, 1H, Ar-H), 7.03 (d, J = 2.0 Hz, 1H, Ar-H), 6.86 (d, J = 8.3 Hz, 1H, Ar-H), 6.82 (d, J = 16.4 Hz, 1H, C_{10}-H), 4.60 (s, 2H, C_{13}-H), 3.91 (s, 3H, C_7-H), 3.90 (s, 3H, C_8-H). ^{13}C NMR (151 MHz, $CDCl_3$) δ 165.95, 162.80, 150.91, 149.34, 138.75, 136.14, 133.56, 132.68, 130.33, 129.65, 127.68, 127.43, 121.90, 111.17, 109.10, 107.24, 56.01, 55.93, 35.20. HRMS (APCI, m/z): calcd. for $C_{19}H_{16}Cl_2N_2O_3S^+$ ($[M+H]^+$) 423.0331, found 423.0327.

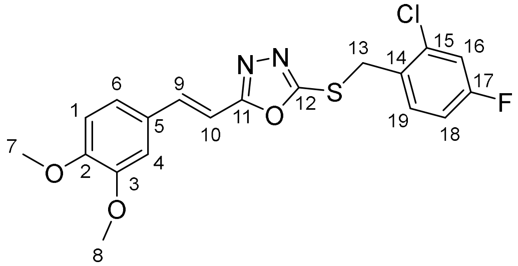

(E)-2-((2-chloro-4-fluorobenzyl)thio)-5-(3,4-dimethoxystyryl)-1,3,4-oxadiazole

(6v):White solid; yield, 75.1%; m.p. 117.8–121.3 °C; IR (KBr, cm^{-1}): 3059.25 (Ar-H, C=C-H), 2935.47, 2912.78, 2834.38 (C-H), 1654.33 (C=N, C=C),1639.89 1600.69, 1588.31, 1514.04, 1474.84 (Ar-C=C), 1276.79 (C-O-C), 732.15(C-S-C). ^{1}H NMR (600 MHz, $CDCl_3$) δ 7.61 (dd, J = 8.6, 6.0 Hz, 1H,Ar-H), 7.36 (d, J = 16.3 Hz, 1H, C_9-H), 7.14 (dd, J = 8.4, 2.6 Hz, 1H, Ar-H), 7.07 (dd, J = 8.3, 2.0 Hz, 1H, Ar-H), 7.03 (d, J = 2.0 Hz, 1H, Ar-H), 6.94 (td, J = 8.3, 2.6 Hz, 1H, Ar-H), 6.86 (d, J = 8.3 Hz, 1H, Ar-H), 6.82 (d, J = 16.3 Hz, 1H, C_{10}-H), 4.55 (s, 2H, C_{13}-H), 3.91 (s, 3H, C_7-H), 3.90 (s, 3H, C_8-H). ^{13}C NMR (151 MHz, $CDCl_3$) δ 165.91, 162.99 (d, J = 36.3 Hz), 161.44, 150.90, 149.35, 138.72, 135.12 (d, J = 10.5 Hz), 132.64 (d, J = 9.0 Hz), 129.84 (d, J = 3.7 Hz), 127.68, 121.89, 117.22 (d, J = 24.9 Hz), 114.33 (d, J = 21.4 Hz), 111.17, 109.08, 107.26, 56.00, 55.92, 33.84. HRMS (APCI, m/z): calcd. For $C_{19}H_{16}ClFN_2O_3S^+$ ($[M+H]^+$) 407.0627, found 407.0621.

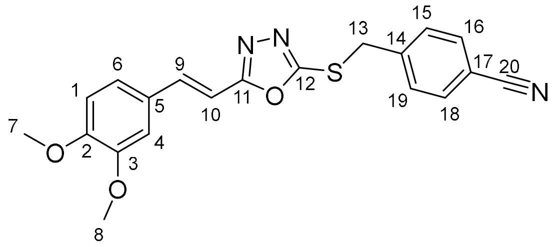

(E)-4-(((5-(3,4-dimethoxystyryl)-1,3,4-oxadiazol-2-yl)thio)methyl)benzonitrile

(6w): White solid; yield, 71.3%; m.p. 168.5–172.4 °C; IR (KBr, cm^{-1}): 3070.09 (Ar-H, C=C-H), 2964.36, 2929.28, 2830.26 (C-H),2229.91 (-C≡N), 1639.89 (C=N, C=C), 1602.75, 1588.31, 1514.04, 1474.84 (Ar-C=C), 1268.54 (C-O-C), 666.13 (C-S-C).^{1}H NMR (600 MHz, $CDCl_3$) δ 7.64 – 7.55 (m, 4H,Ar-H), 7.35 (d, J = 16.4 Hz, 1H,C_9-H), 7.07 (dd, J = 8.3, 2.0 Hz, 1H, Ar-H), 7.03 (d, J = 2.0 Hz, 1H, Ar-H), 6.87 (d, J = 8.3 Hz, 1H, Ar-H), 6.82 (d, J = 16.3 Hz, 1H, C_{10}-H), 4.49 (s, 2H, C_{13}-H), 3.91 (s, 3H, C_7-H), 3.90 (s, 3H, C_8-H). ^{3}C NMR (151 MHz, $CDCl_3$) δ 166.05, 162.22, 150.98, 149.36, 141.45, 138.95, 132.52, 129.92, 127.59, 121.97, 118.46, 111.95, 111.19, 109.09, 107.11, 56.02, 55.95, 35.96. HRMS (APCI, m/z): calcd. for $C_{20}H_{17}N_3O_3S^+$ ($[M+H]^+$) 380.1063, found 380.1056.

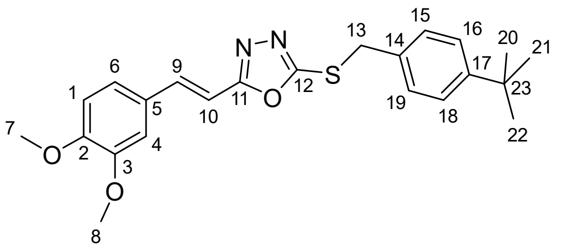

(E)-2-((4-(tert-butyl)benzyl)thio)-5-(3,4-dimethoxystyryl)-1,3,4-oxadiazole (6x): White solid; yield, 71.3%; m.p. 117.1 – 122.3 °C; IR (KBr, cm^{-1}): 3055.91 (Ar-H, C=C-H), 2958.39, 2931.41, 2840.12 (C-H), 1647.03 (C=N, C=C), 1601.38, 1584.78, 1508.01, 1472.74 (Ar-C=C), 1265.24 (C-O-C), 702.94 (C-S-C). ^{1}H NMR (600 MHz, $CDCl_3$) δ 7.40 – 7.34 (m, 5H, Ar-H, C_9-H), 7.09 (dd, J = 8.4, 2.0 Hz, 1H, Ar-H), 7.05 (d, J = 2.0 Hz, 1H, Ar-H), 6.89 – 6.83 (m, 2H, Ar-H, C_8-H), 4.49 (s, 2H, C_{13}-H), 3.92 (s, 3H, C_7-H), 3.91 (s, 3H, C_8-H), 1.31 (s, 9H, $C_{20,21,22}$-H). ^{13}C NMR (151 MHz, $CDCl_3$) δ 165.66, 163.34, 151.20, 150.85, 149.35, 138.48, 132.36, 128.88, 127.79, 125.79, 121.83, 111.18, 109.08, 107.47, 56.01, 55.93, 36.53, 34.61, 31.29. HRMS (APCI, m/z): calcd. for $C_{23}H_{26}N_2O_3S^+$ ($[M+H]^+$) 411.1737, found 411.1730.

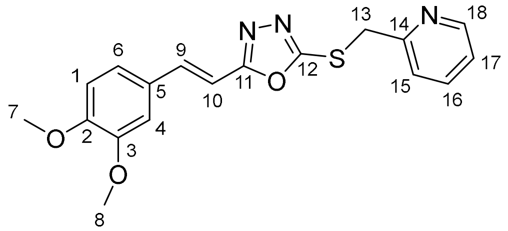

(E)-2-(3,4-dimethoxystyryl)-5-((pyridin-2-ylmethyl)thio)-1,3,4-oxadiazole (6y): White solid; yield, 76.4%; m.p. 105.3 – 109.5 °C; IR (KBr, cm^{-1}): 3079.65 (Ar-H, C=C-H), 2984.17, 2929.39, 2833.39 (C-H), 1645.91 (C=N, C=C), 1600.00, 1583.30, 1506.17, 1474.78 (Ar-C=C), 1268.17 (C-O-C), 729.74 (C-S-C). ^{1}H NMR (600 MHz, $CDCl_3$) δ 8.56 (d, J = 4.9 Hz, 1H, Ar-H), 7.64 (td, J = 7.7, 1.8 Hz, 1H, Ar-H), 7.50 (d, J = 7.8 Hz, 1H, Ar-H), 7.35 (d, J = 16.3 Hz, 1H, C_9-H), 7.19 (dd, J = 6.5, 4.8 Hz, 1H, Ar-H), 7.06 (dd, J = 8.3, 2.0 Hz, 1H, Ar-H), 7.02 (d, J = 2.0 Hz, 1H, Ar-H), 6.85 (d, J = 8.3 Hz, 1H Ar-H), 6.83 (s, 1H, C_{10}-H), 4.62 (s, 2H, C_{13}-H), 3.89 (s, 3H, C_7-H), 3.88 (s, 3H, C_8-H). ^{13}C NMR (151 MHz, $CDCl_3$) δ 165.81, 163.24, 155.47, 150.84, 149.68, 149.32, 138.60, 136.90, 127.72, 123.49, 122.78, 121.84, 111.16, 109.09, 107.32, 55.99, 55.91, 38.30.

HRMS (APCI, m/z): calcd. for $C_{18}H_{17}N_3O_3S^+$ ($[M+H]^+$) 356.1063, found 356.1057.

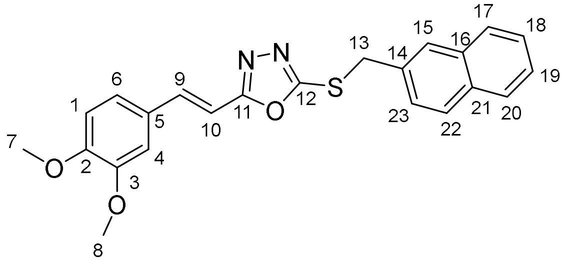

(E)-2-(3,4-dimethoxystyryl)-5-((naphthalen-2-ylmethyl)thio)-1,3,4-oxadiazole (6z): White solid; yield, 72.9%; m.p. 133.6 – 138.9 °C; IR (KBr, cm^{-1}): 3047.00 (Ar-H, C=C-H), 2963.03, 2939.94, 2828.69 (C-H), 1648.98 (C=N, C=C), 1600.70, 1586.01, 1510.44, 1474.75 (Ar-C=C), 1264.84(C-O-C), 731.66 (C-S-C). ^1H NMR (600 MHz, $CDCl_3$) δ 8.10 (d, J = 8.4 Hz, 1H, Ar-H), 7.88 (d, J = 7.5 Hz, 1H, Ar-H), 7.83 (d, J = 8.2 Hz, 1H, Ar-H), 7.63 (d, J = 5.9 Hz, 1H, Ar-H), 7.60 – 7.56 (m, 1H, Ar-H), 7.54 – 7.51 (m, 1H, Ar-H), 7.42 (dd, J = 8.3, 7.0 Hz, 1H, Ar-H), 7.35 (d, J = 16.4 Hz, 1H, C_9-H), 7.07 (dd, J = 8.3, 2.0 Hz, 1H, Ar-H), 7.04 (d, J = 2.0 Hz, 1H, Ar-H), 6.87 (d, J = 2.1 Hz, 1H, Ar-H), 6.85 (d, J = 5.9 Hz, 1H, C_{10}-H), 5.00 (s, 2H, C_{13}-H), 3.92 (s, 3H, C_7-H), 3.90 (s, 3H, C_8-H). ^{13}C NMR (151 MHz, $CDCl_3$) δ 165.75, 163.36, 150.87, 149.34, 138.60, 133.98, 131.31, 130.69, 129.35, 129.05, 128.41, 127.75, 126.79, 126.17, 125.43, 123.40, 121.87, 111.18, 109.10, 107.38, 56.01, 55.93, 34.87. HRMS (APCI, m/z): calcd. For $C_{23}H_{20}N_2O_3S^+$ ($[M+H]^+$) 405.1267, found 405.1259.

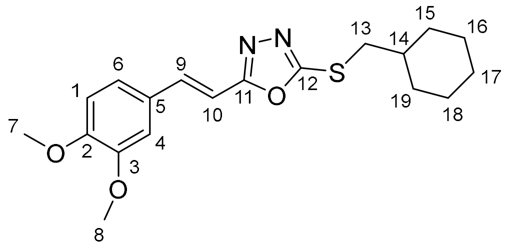

(E)-2-((cyclohexylmethyl)thio)-5-(3,4-dimethoxystyryl)-1,3,4-oxadiazole (6aa): White solid; yield, 76.3%; m.p. 83.8 – 86.7 °C; IR (KBr, cm^{-1}): 3055.13 (Ar-H, C=C-H), 2963.80, 2927.78, 2829.20 (C-H), 1641.95 (C=N, C=C), 1600.69, 1582.12, 1506.71, 1472.52 (Ar-C=C), 1265.28 (C-O-C), 735.43 (C-S-C). ^1H NMR (600 MHz, $CDCl_3$) δ 7.36 (d, J = 16.3 Hz, 1H,C_9-H), 7.07 (dd, J = 8.3, 2.0 Hz, 1H, Ar-H), 7.04 (d, J = 2.0 Hz, 1H, Ar-H), 6.87 – 6.82 (m, 2H, Ar-H, C_{10}-H), 3.91 (s, 3H, C_7-H), 3.89 (s, 3H, C_8-

H), 3.17 (d, J = 6.9 Hz, 2H, C_{13}-H), 1.89 (d, J = 11.6 Hz, 2H, C_{15}-H), 1.73 (dt, J = 13.6, 3.6 Hz, 3H, $C_{16,17}$-H), 1.67 – 1.62 (m, 1H, C_{14}-H), 1.29 – 1.20 (m, 2H, C_{18}-H), 1.19 – 1.10 (m, 1H, C_{17}-H), 1.02 (qd, J = 12.3, 3.5 Hz, 2H, C_{19}-H). ^{13}C NMR (151 MHz, $CDCl_3$) δ 165.47, 164.24, 150.79, 149.32, 138.27, 127.82, 121.80, 111.16, 109.04, 107.52, 56.00, 55.91, 39.47, 37.47, 32.38, 26.11, 25.85. HRMS (APCI, m/z): calcd. for $C_{19}H_{24}N_2O_3S^+$ ([M+H]$^+$) 361.1580, found 361.1575.

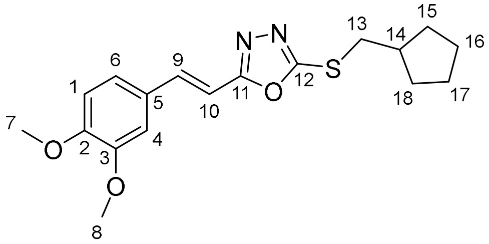

(E)-2-((cyclopentylmethyl)thio)-5-(3,4-dimethoxystyryl)-1,3,4-oxadiazole (6ab): White solid; yield, 75.6%; m.p. 68.1 – 75.8 °C; IR (KBr, cm^{-1}): 3013.87 (Ar-H, C=C-H), 2968.48, 2937.54, 2832.32 (C-H), 1635.76 (C=N, C=C), 1598.62, 1588.31, 1516.10, 1478.97 (Ar-C=C), 1264.41 (C-O-C), 771.35 (C-S-C). ^{1}H NMR (600 MHz, $CDCl_3$) δ 7.37 (d, J = 16.4 Hz, 1H, C_9-H), 7.07 (dd, J = 8.3, 2.0 Hz, 1H, Ar-H), 7.04 (d, J = 2.0 Hz, 1H, Ar-H), 6.88 – 6.82 (m, 2H, Ar-H, C_{10}-H), 3.91 (s, 3H, C_7-H), 3.89 (s, 3H, C_8-H), 3.28 (d, J = 7.4 Hz, 2H, C_{13}-H), 2.36 – 2.25 (m, 1H, C_{14}-H), 1.93 – 1.85 (m, 2H, C_{15}-H), 1.70 – 1.62 (m, 2H, C_{18}-H), 1.61 – 1.53 (m, 2H, C_{16}-H), 1.30 (dq, J = 12.7, 8.2 Hz, 2H, C_{17}-H). ^{13}C NMR (151 MHz, $CDCl_3$) δ 165.47, 164.07, 150.77, 149.31, 138.24, 127.80, 121.77, 111.15, 109.03, 107.52, 55.99, 55.90, 39.37, 38.34, 32.22, 25.22. HRMS (APCI, m/z): calcd. for $C_{18}H_{22}N_2O_3S^+$ ([M+H]$^+$) 347.1424, found 347.1416.

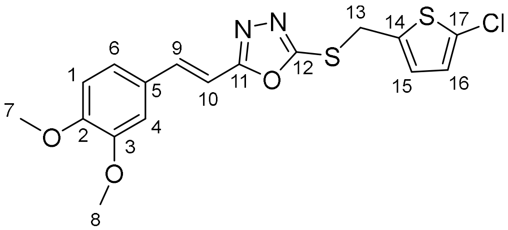

(E)-2-(((5-chlorothiophen-2-yl)methyl)thio)-5-(3,4-dimethoxystyryl)-1,3,4-oxadiazole (6ac): White solid; yield, 77.2%; m.p. 107.8 – 110.3 °C; IR (KBr, cm^{-1}): 3051.00 (Ar-H, C=C-H), 2958.17, 2931.35, 2840.57 (C-H), 1641.95 (C=N, C=C),

1602.75, 1586.25, 1509.91, 1476.91 (Ar-C=C), 1268.54 (C-O-C), 703.27 (C-S-C). ^{1}H NMR (600 MHz, $CDCl_3$) δ 7.38 (d, J = 16.3 Hz, 1H, C_9-H), 7.09 (dd, J = 8.4, 2.1 Hz, 1H, Ar-H), 7.05 (d, J = 2.0 Hz, 1H, Ar-H), 6.91 – 6.88 (m, 1H, Ar-H), 6.87 (d, J = 8.3 Hz, 1H, Ar-H), 6.84 (d, J = 16.3 Hz, 1H, C_9-H), 6.73 (d, J = 3.8 Hz, 1H, Ar-H), 4.60 (s, 2H, C_{13}-H), 3.92 (s, 3H, C_7-H), 3.91 (s, 3H, C_8-H). ^{13}C NMR (151 MHz, $CDCl_3$) δ 166.01, 162.33, 150.93, 149.36, 138.87, 136.81, 130.35, 127.68, 127.42, 125.95, 121.92, 111.18, 109.11, 107.24, 56.02, 55.94, 31.66. HRMS (APCI, m/z): calcd. for $C_{17}H_{15}ClN_2O_3S_2^+$ ($[M+H]^+$) 395.0285, found 395.0280.

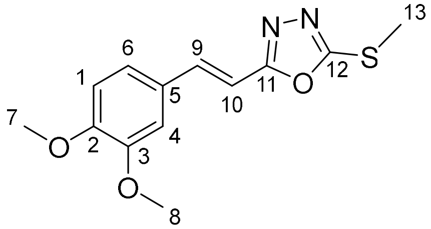

(E)-2-(3,4-dimethoxystyryl)-5-(methylthio)-1,3,4-oxadiazole(6ad): White solid; yield, 85.3%; m.p.123.3– 131.5 °C ; IR (KBr, cm^{-1}): 3046.40 (Ar-H, C=C-H), 2943.23, 2928.79, 2850.39 (C-H), 1639.23(C=N, C=C), 1602.09, 1587.65, 1511.31, 1474.17 (Ar-C=C), 1282.28 (C-O-C), 710.75 (C-S-C). ^{1}H NMR (600 MHz, $CDCl_3$) δ 7.37 (d, J = 16.4 Hz, 1H, C_9-H), 7.07 (dd, J = 8.2, 2.0 Hz, 1H, Ar-H), 7.04 (d, J = 2.0 Hz, 1H, Ar-H), 6.88 – 6.81 (m, 2H, Ar-H, C_9-H), 3.91 (s, 3H, C_7-H), 3.89 (s, 3H, C_8-H), 2.73 (s, 3H, C_{13}-H). ^{13}C NMR (151 MHz, $CDCl_3$) δ 165.70, 164.31, 150.82, 149.33, 138.38, 127.76, 121.80, 111.17, 109.06, 107.43, 56.00, 55.91, 14.57. HRMS (APCI, m/z): calcd. for $C_{13}H_{14}N_2O_3S^+$ ($[M+H]^+$) 279.0798, found 279.0793.

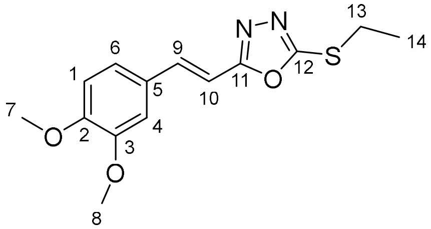

(E)-2-(3,4-dimethoxystyryl)-5-(ethylthio)-1,3,4-oxadiazole (6ae): White solid; yield, 80.6%; m.p. 93.1 – 96.8 °C; IR (KBr, cm-1): 3053.07 (Ar-H, C=C-H), 2965.15, 2925.65, 2831.18 (C-H), 1644.92 (C=N, C=C), 1599.84, 1586.87, 1505.91, 1471.31 (Ar-C=C), 1264.76 (C-O-C), 605.39 (C-S-C). ^{1}H NMR (600 MHz, $CDCl_3$) δ 7.37 (d, J = 16.4 Hz,

1H, C_9-H), 7.07 (dd, J = 8.3, 2.0 Hz, 1H,Ar-H), 7.04 (d, J = 2.0 Hz, 1H, Ar-H), 6.87 (d, J = 2.7 Hz, 1H, Ar-H), 6.85 (d, J = 5.4 Hz, 1H, C_{10}-H), 3.91 (s, 3H, C_7-H), 3.89 (s, 3H. C_8-H), 3.28 (q, J = 7.4 Hz, 2H, C_{13}-H), 1.48 (t, J = 7.4 Hz, 3H, C_{14}-H). ^{13}C NMR (151 MHz, $CDCl_3$) δ 165.57, 163.64, 150.81, 149.32, 138.40, 127.78, 121.82, 111.16, 109.06, 107.46, 56.00, 55.92, 26.97, 14.74. HRMS (APCI, m/z): calcd. for $C_{14}H_{16}N_2O_3S^+$ ([M+H]$^+$) 293.0954, found 293.0948.

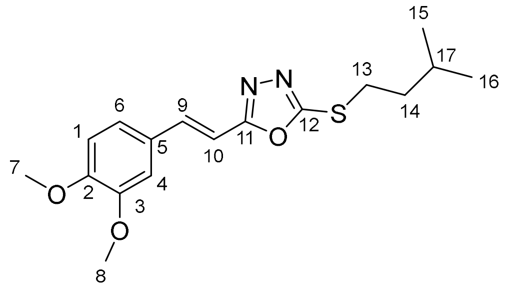

(E)-2-(3,4-dimethoxystyryl)-5-(isopentylthio)-1,3,4-oxadiazole(6af): White solid; yield, 78.5%; m.p. 73.6 – 77.4 °C;IR (KBr, cm-1): 3051.00 (Ar-H, C=C-H), 2960.23, 2937.54, 2838.51 (C-H), 1644.01 (C=N, C=C), 1600.69, 1588.31, 1509.91, 1472.78 (Ar-C=C), 1266.48(C-O-C), 728.02 (C-S-C). ^1H NMR (600 MHz, $CDCl_3$) δ 7.37 (d, J = 16.3 Hz, 1H, C_9-H), 7.07 (dd, J = 8.3, 2.0 Hz, 1H, Ar-H), 7.04 (d, J = 1.9 Hz, 1H, Ar-H), 6.88 – 6.82 (m, 2H, Ar-H, C_{10}-H), 3.91 (s, 3H, C_7-H), 3.90 (s, 3H, C_8-H), 3.30 – 3.24 (m, 2H, C_{13}-H), 1.74 (dt, J = 13.0, 6.5 Hz, 1H, C_{17}-H), 1.71 – 1.66 (m, 2H, C_{14}-H), 0.94 (d, J = 6.5 Hz, 6H, $C_{15,16}$-H). ^{13}C NMR (151 MHz, $CDCl_3$) δ 165.51, 163.84, 150.80, 149.33, 138.29, 127.80, 121.80, 111.16, 109.04, 107.51, 55.99, 55.91, 37.97, 30.69, 27.40, 22.15. HRMS (APCI, m/z): calcd. for $C_{17}H_{23}N_2O_3S^+$ ([M+H]$^+$) 335.1424, found 335.1418.

2.The FT-IR, 1H NMR, 13C NMR, and HRMS spectra of the target compounds 6a~6af

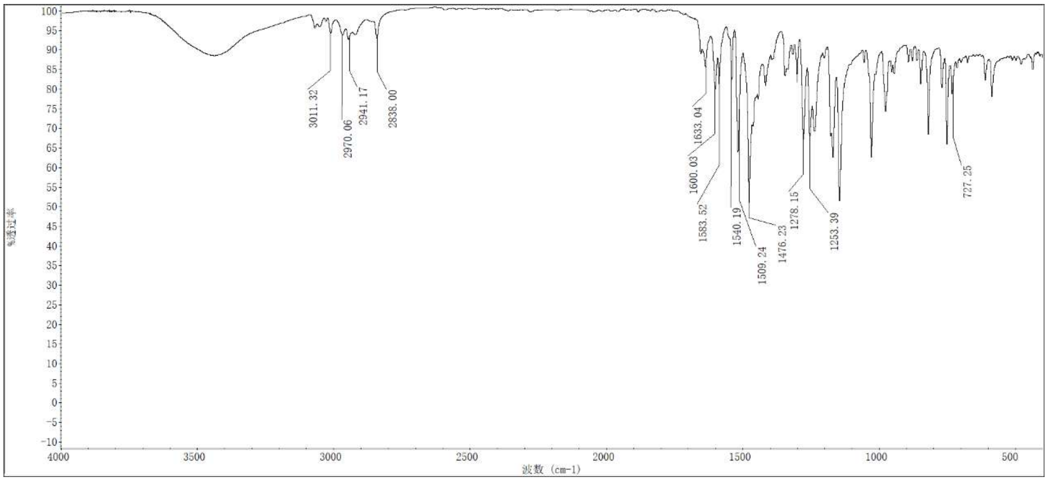

Fig. S1 The FT-IR spectrum of the target compound **6a**

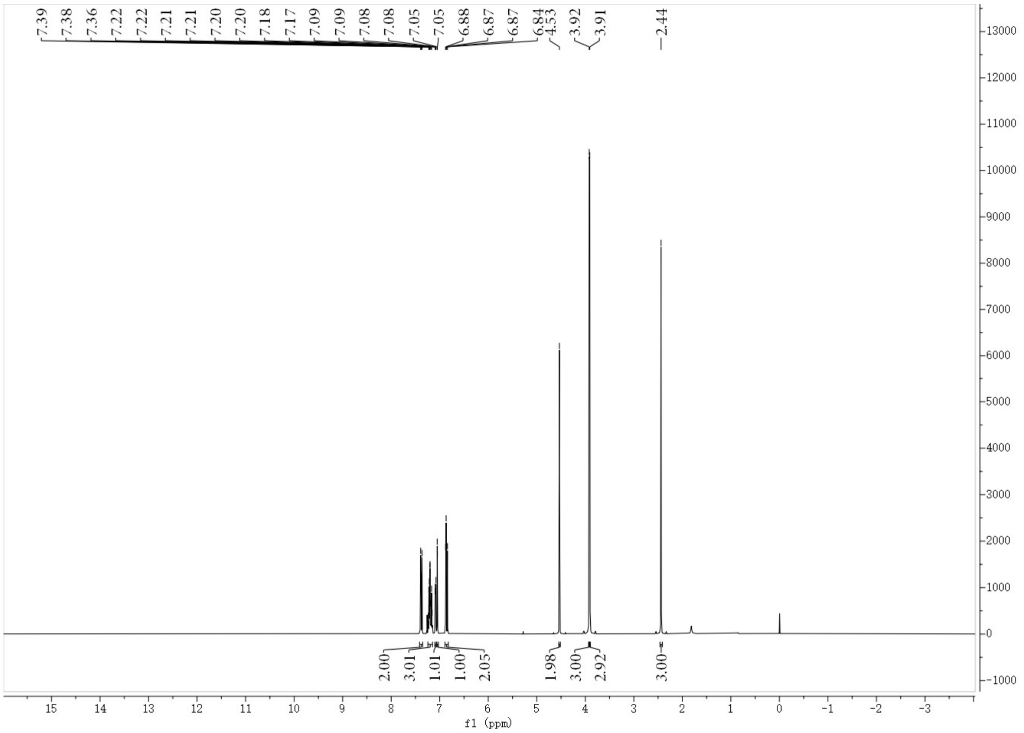

Fig. S2 The ^{1}H NMR spectrum of the target compound **6a**

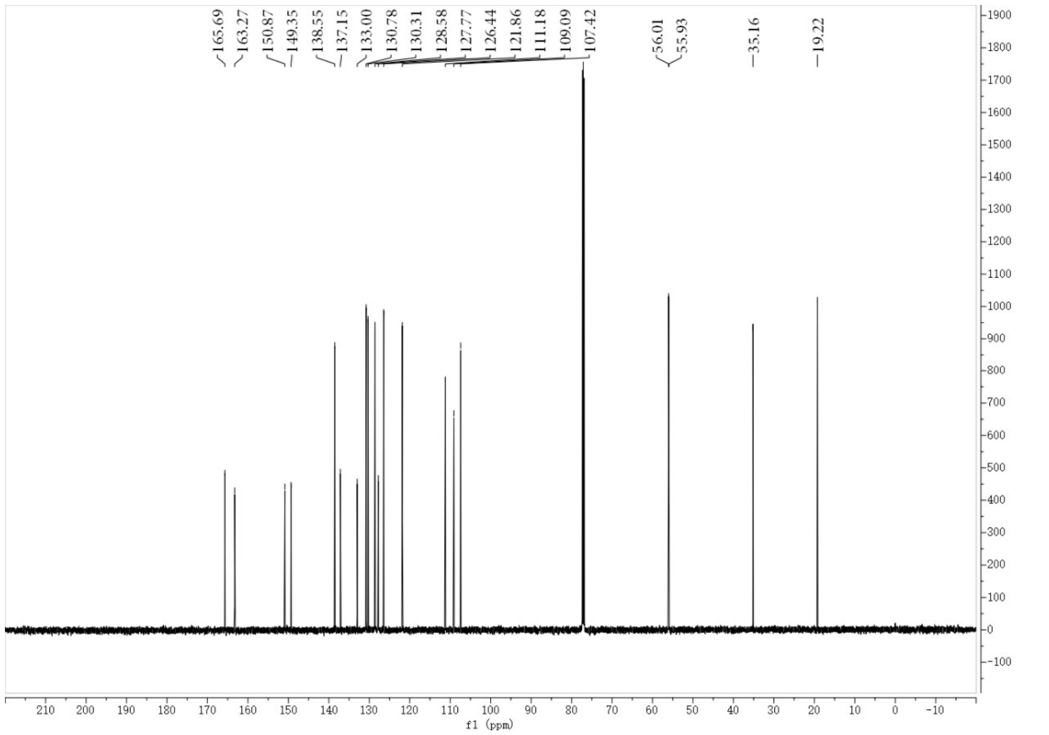

Fig. S3 The ^{13}C NMR spectrum of the target compound **6a**

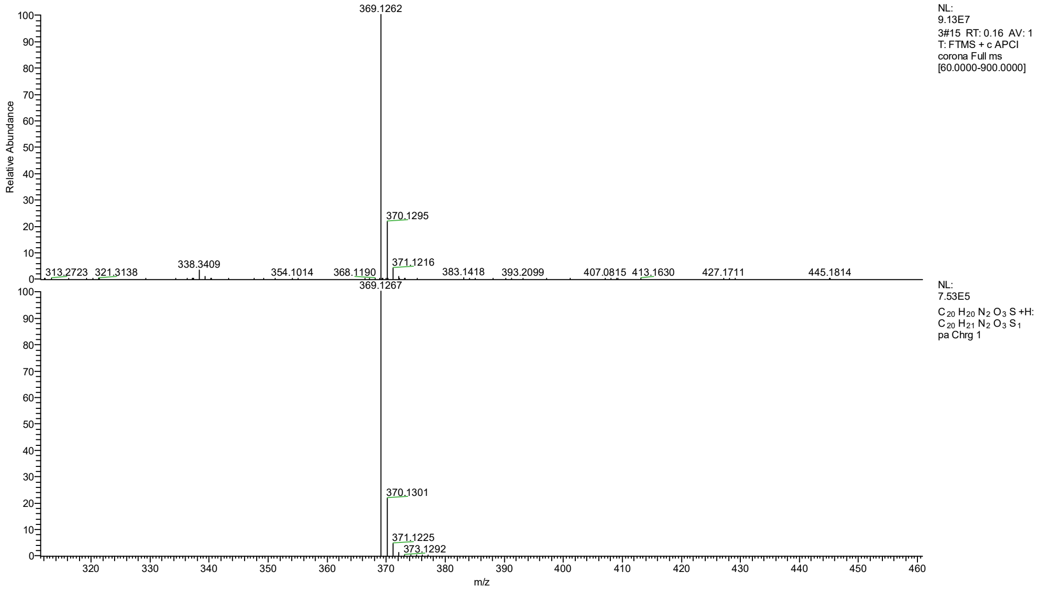

Fig. S4 The HRMS spectrum of the target compound **6a**

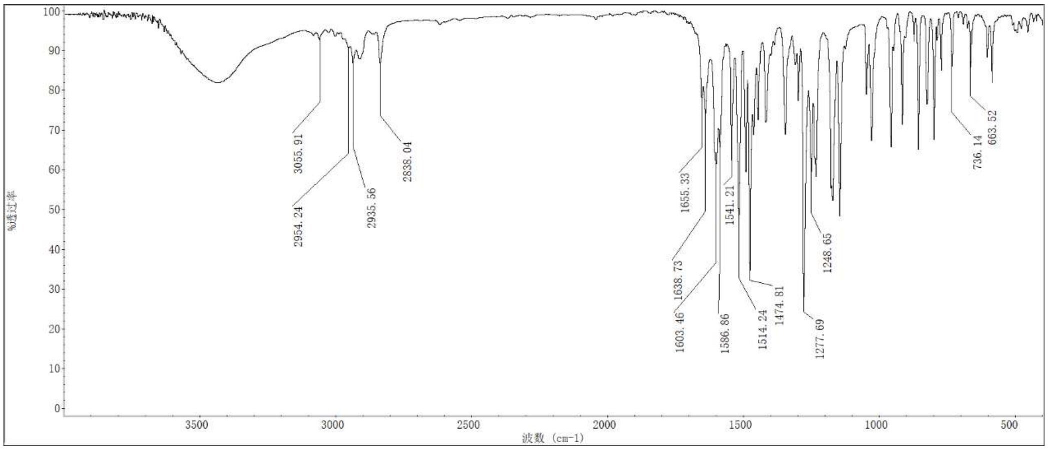

Fig. S5 The FT-IR spectrum of the target compound **6b**

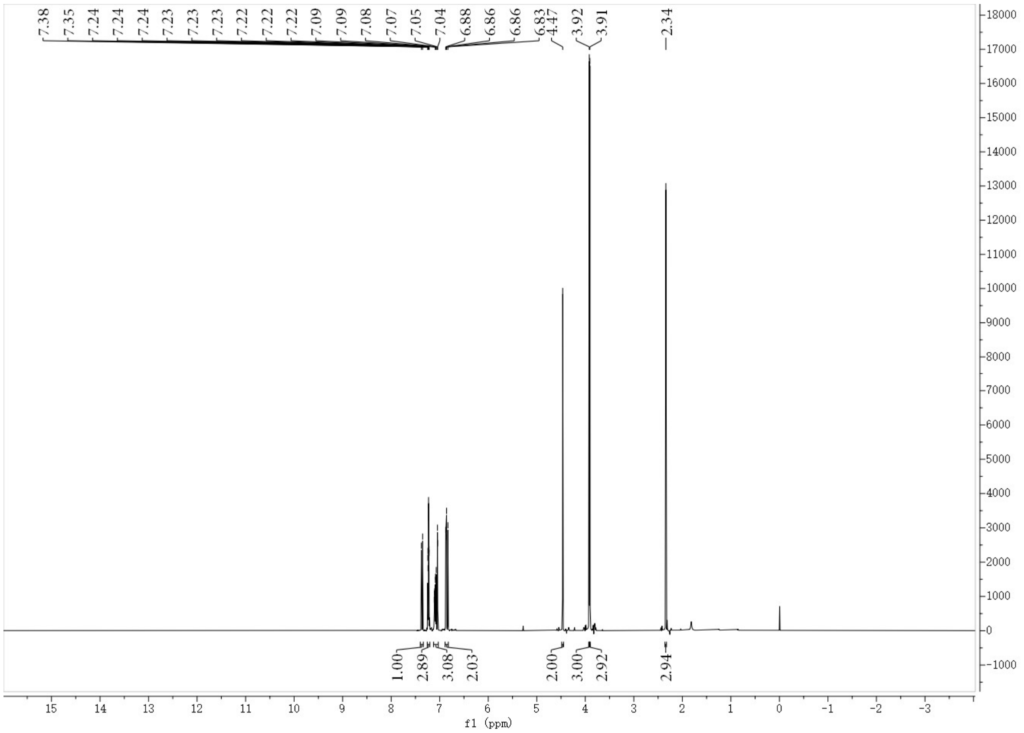

Fig. S6 The ^{1}H NMR spectrum of the target compound **6b**

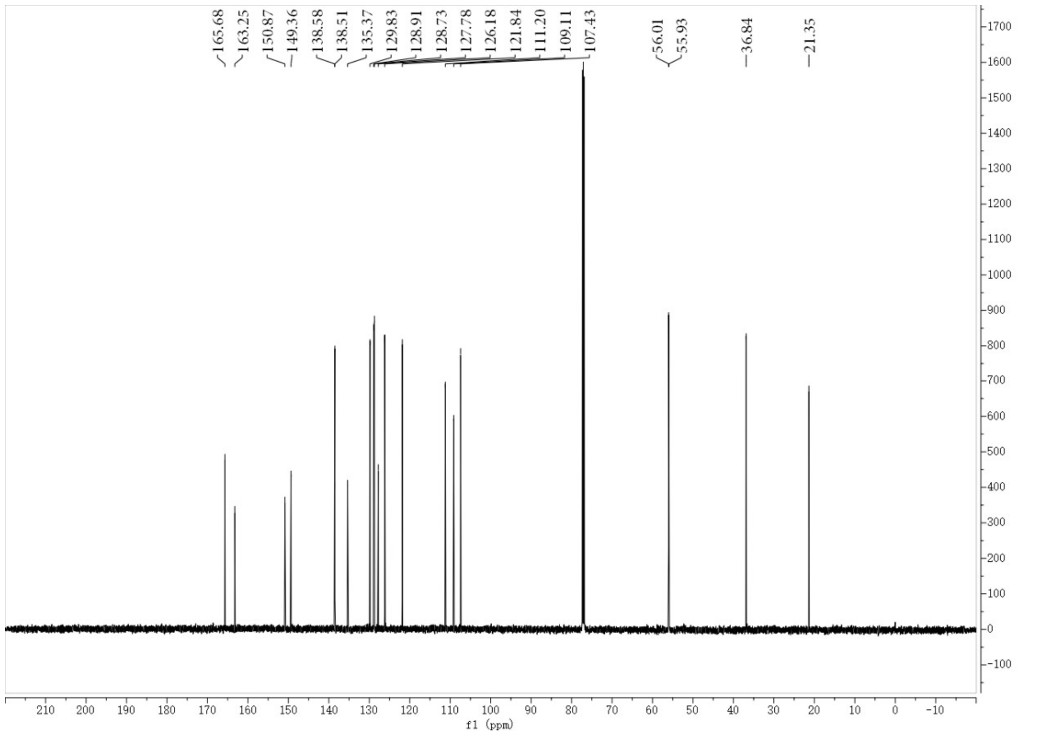

Fig. S7 The ^{13}C NMR spectrum of the target compound **6b**

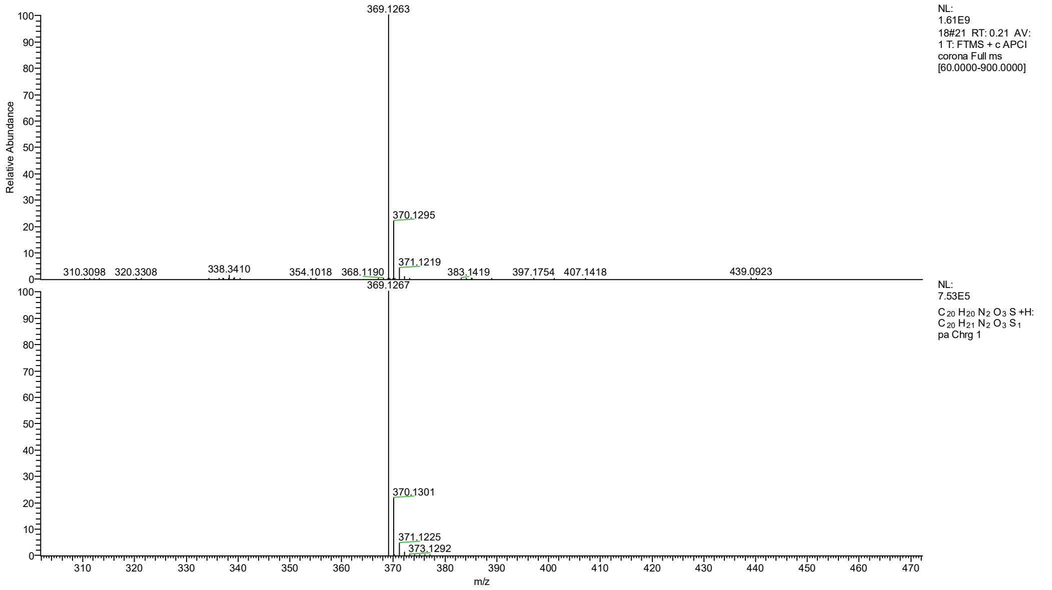

Fig. S8 The HRMS spectrum of the target compound **6b**

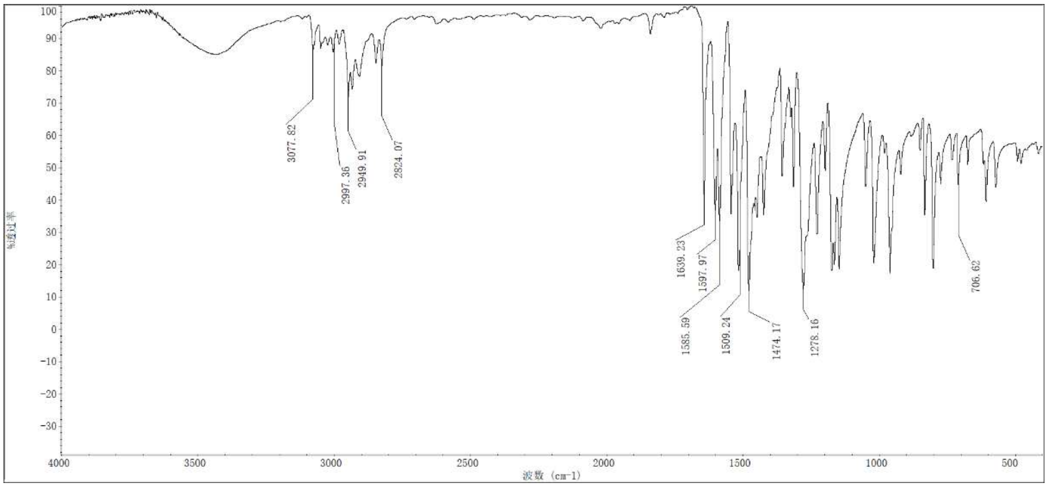

Fig. S9 The FT-IR spectrum of the target compound **6c**

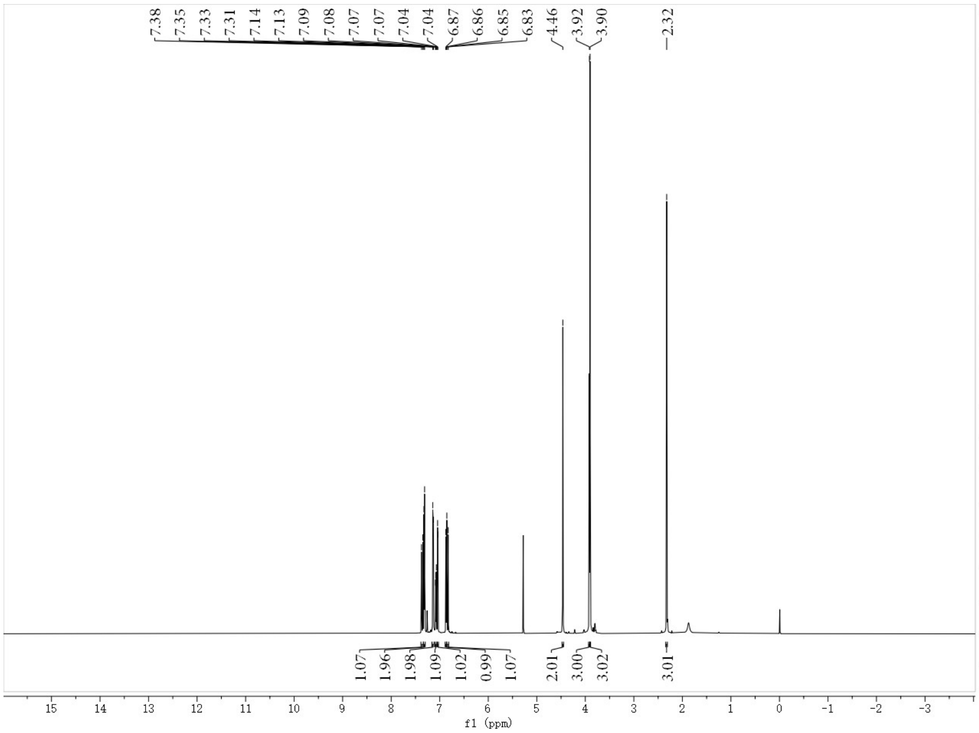

Fig. S10 The ^{1}H NMR spectrum of the target compound **6c**

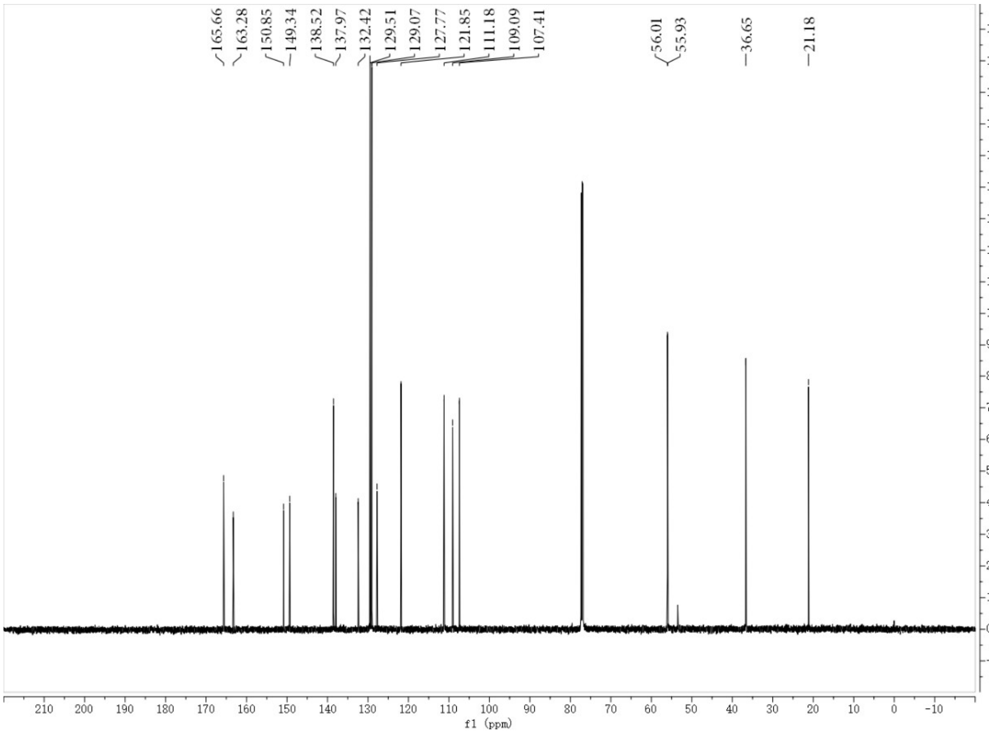

Fig. S11 The ^{13}C NMR spectrum of the target compound **6c**

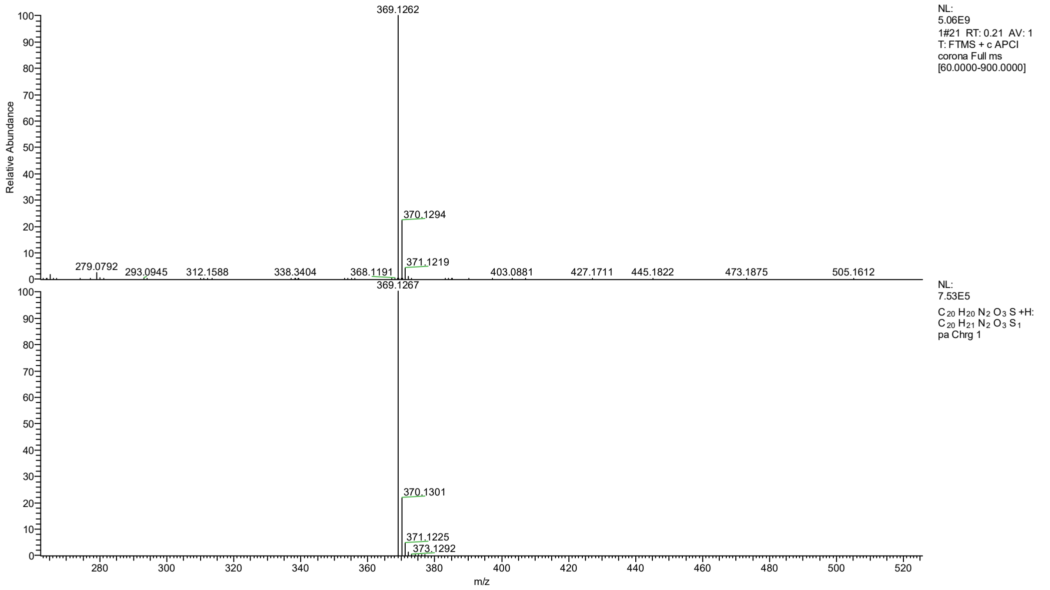

Fig. S12 The HRMS spectrum of the target compound **6c**

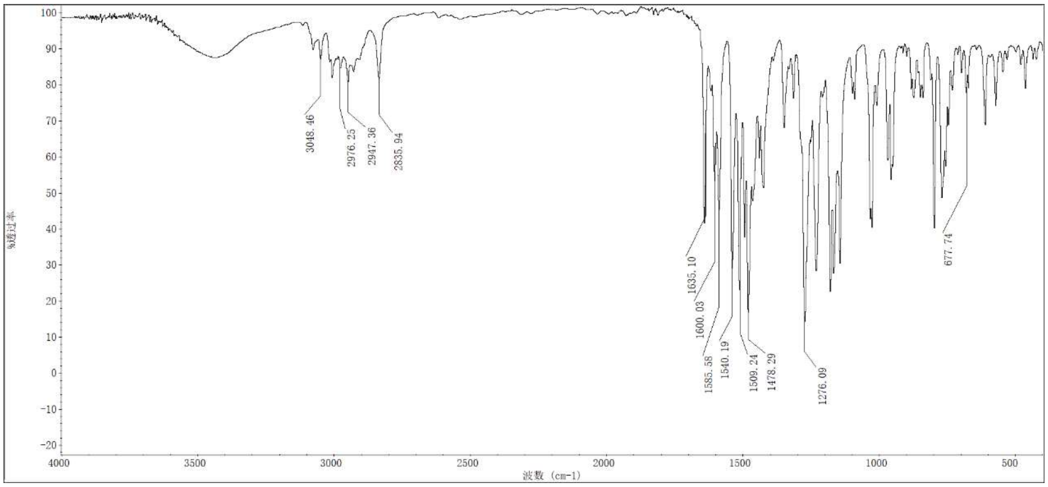

Fig. S13 The FT-IR spectrum of the target compound **6d**

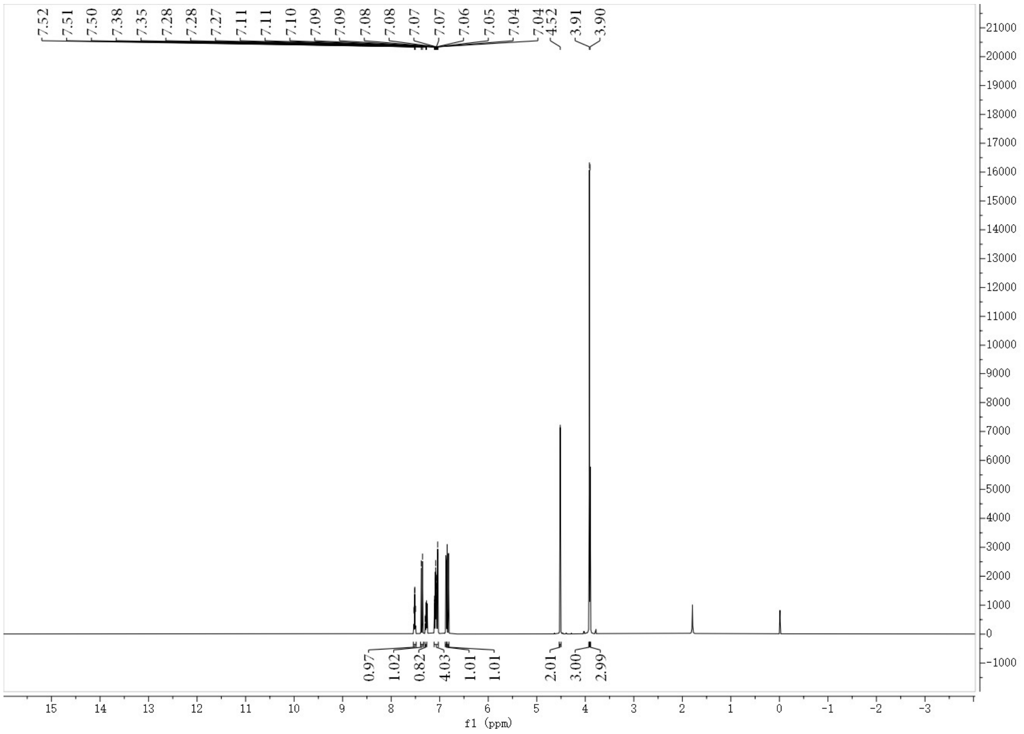

Fig. S14 The ^{1}H NMR spectrum of the target compound **6d**

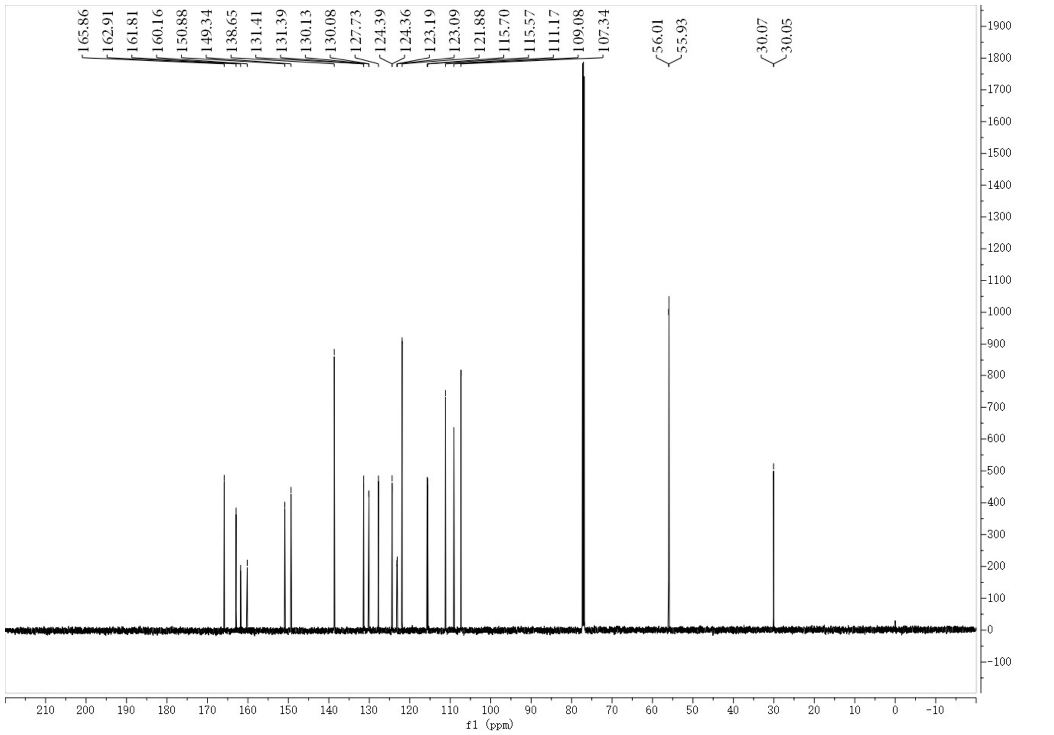

Fig. S15 The ^{13}C NMR spectrum of the target compound **6d**

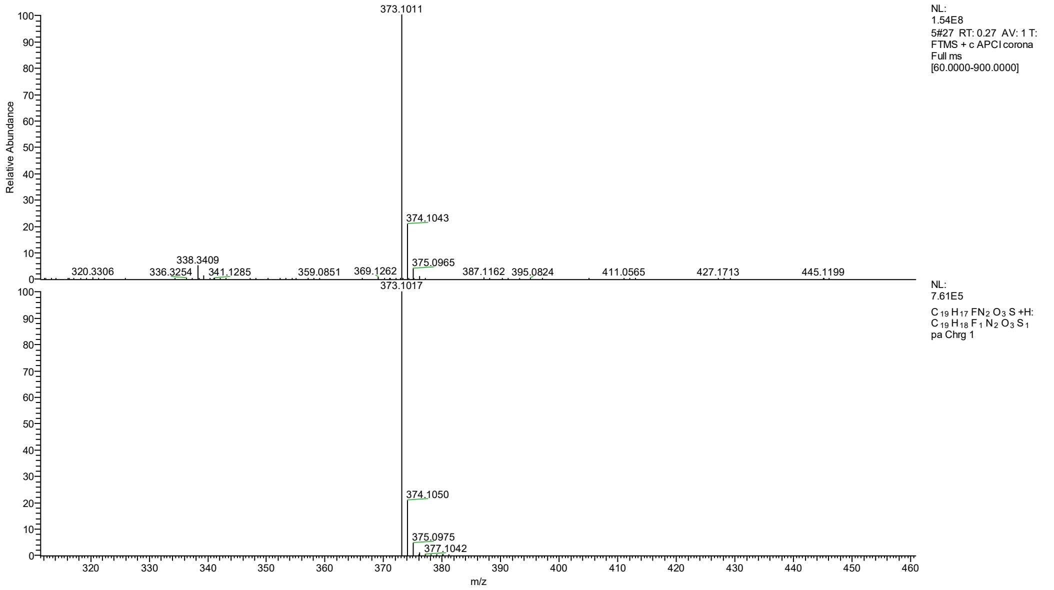

Fig. S16 The HRMS spectrum of the target compound **6d**

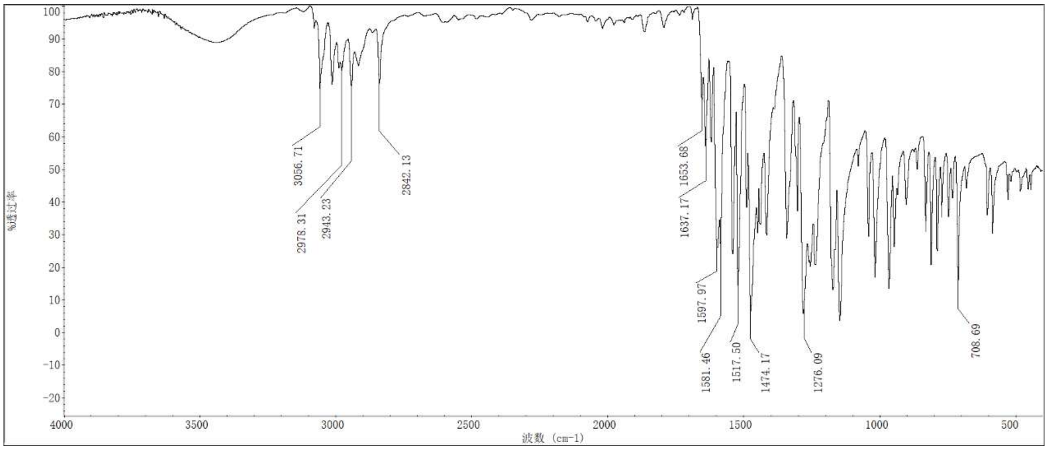

Fig. S17 The FT-IR spectrum of the target compound **6e**

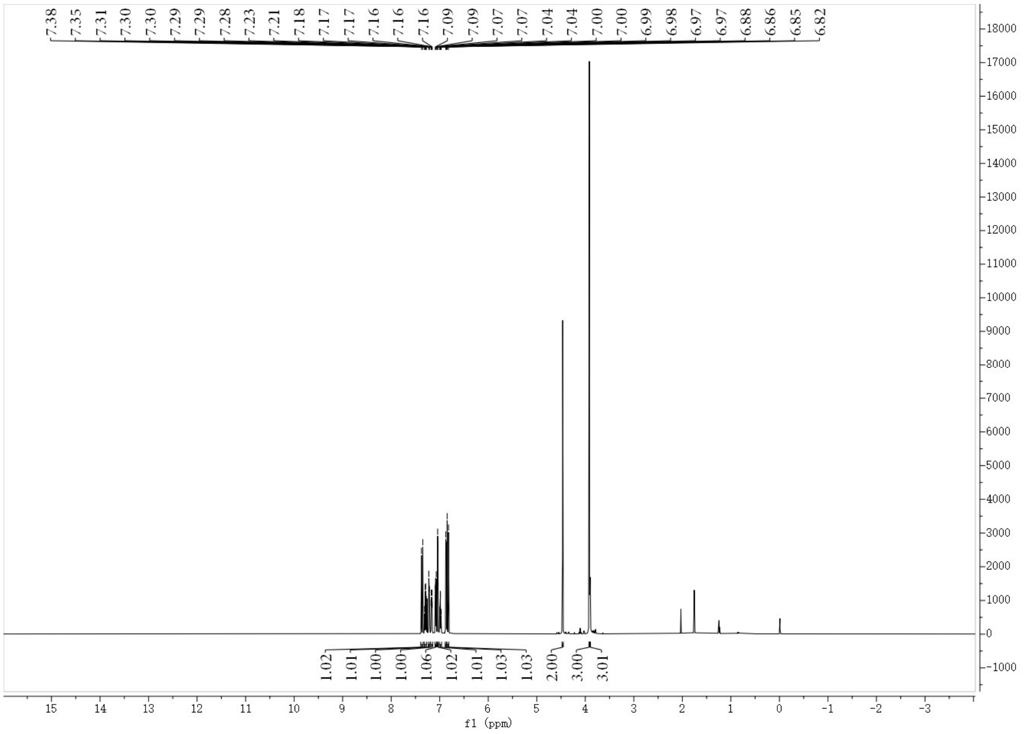

Fig. S18 The ^{1}H NMR spectrum of the target compound **6e**

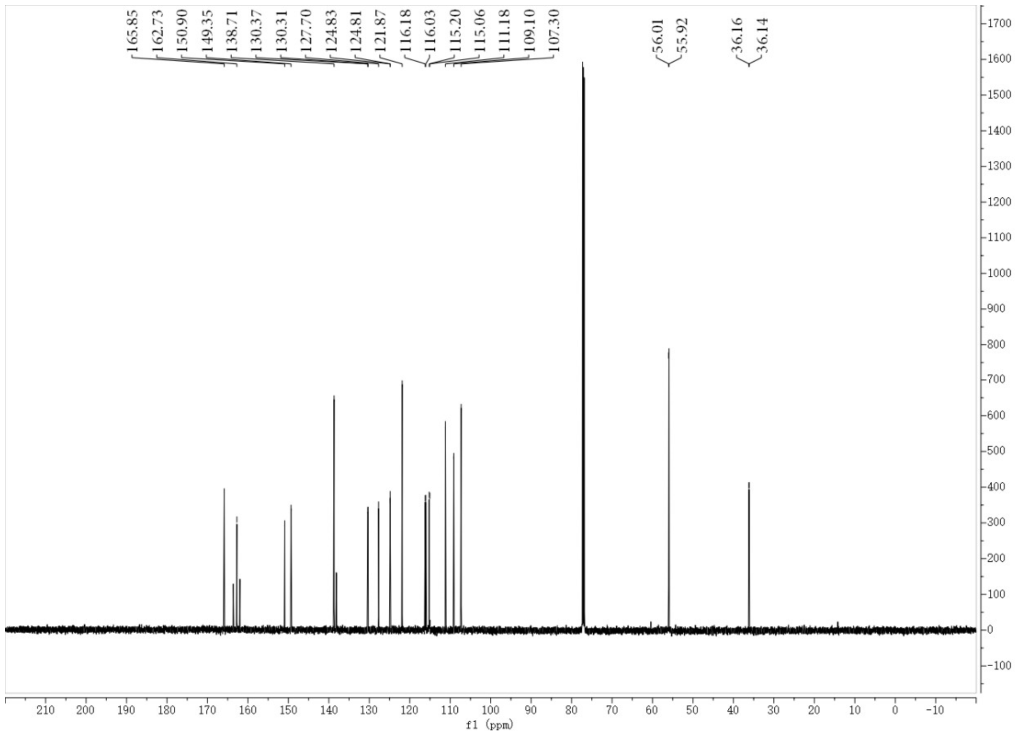

Fig. S19 The ^{13}C NMR spectrum of the target compound **6e**

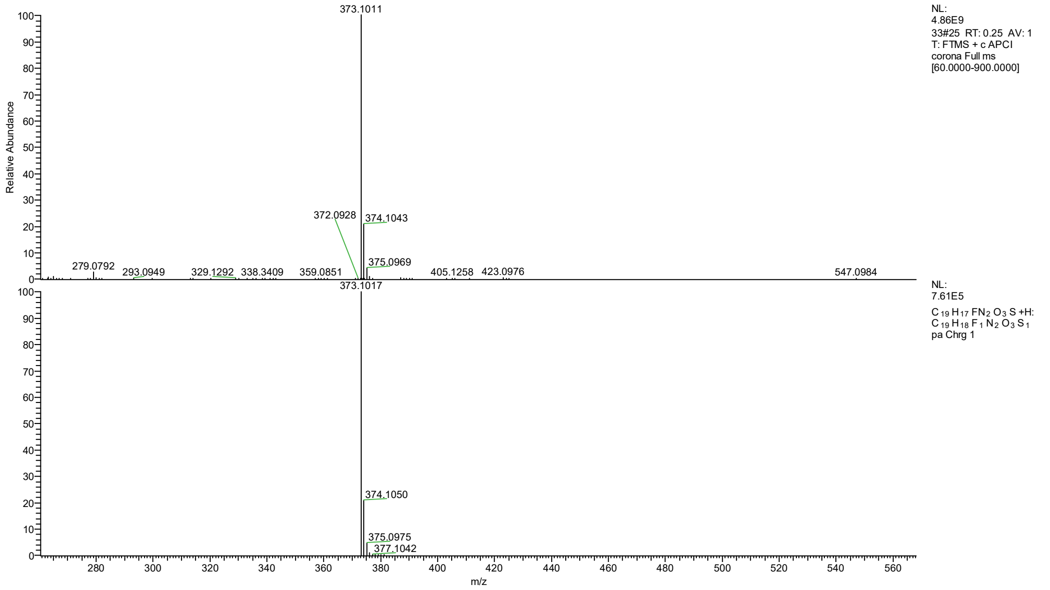

Fig. S20 The HRMS spectrum of the target compound **6e**

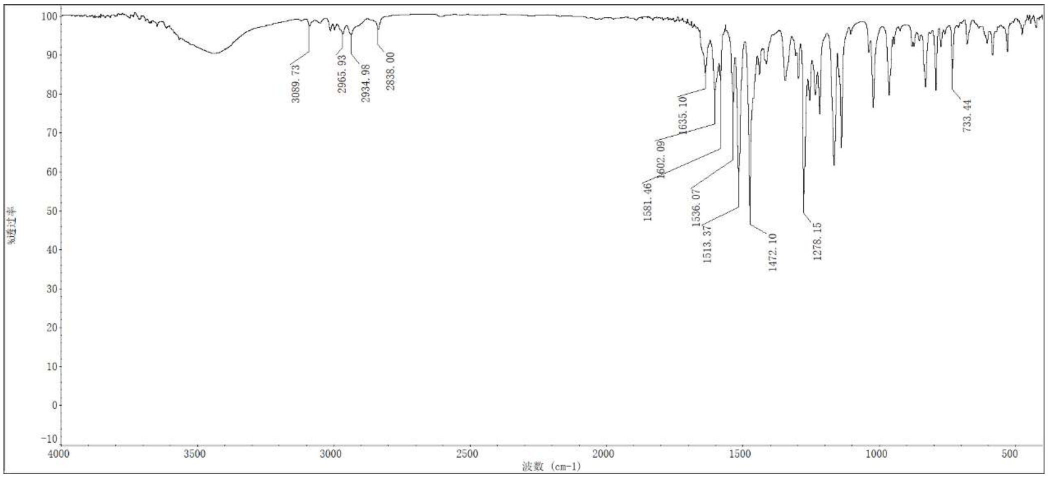

Fig. S21 The FT-IR spectrum of the target compound **6f**

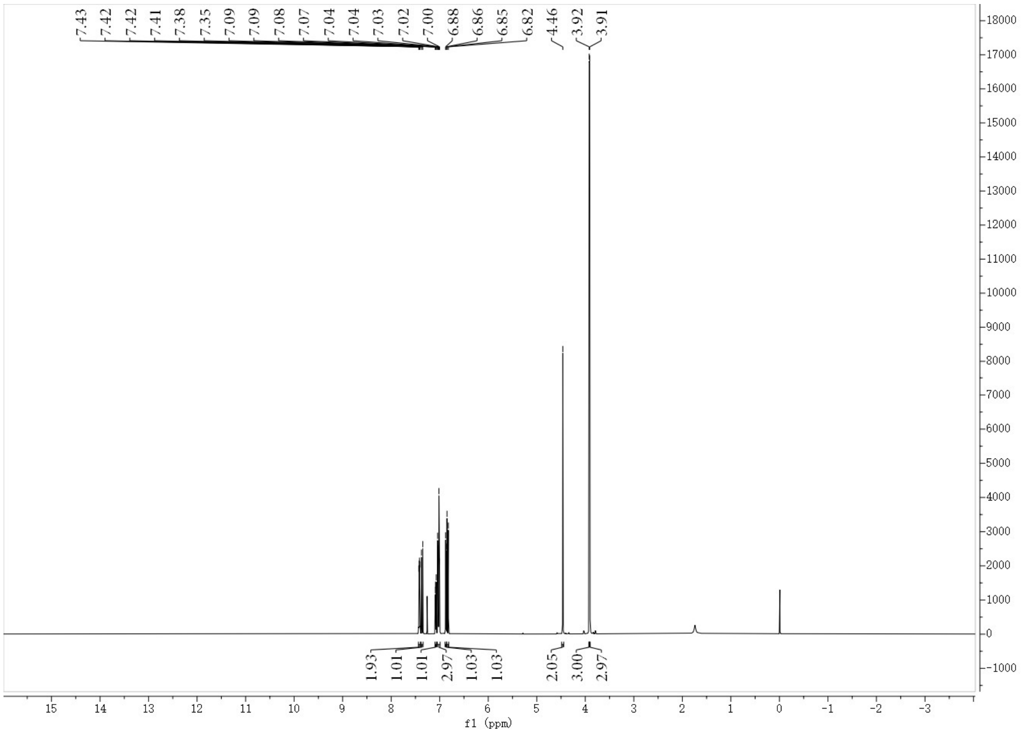

Fig. S22 The ^{1}H NMR spectrum of the target compound **6f**

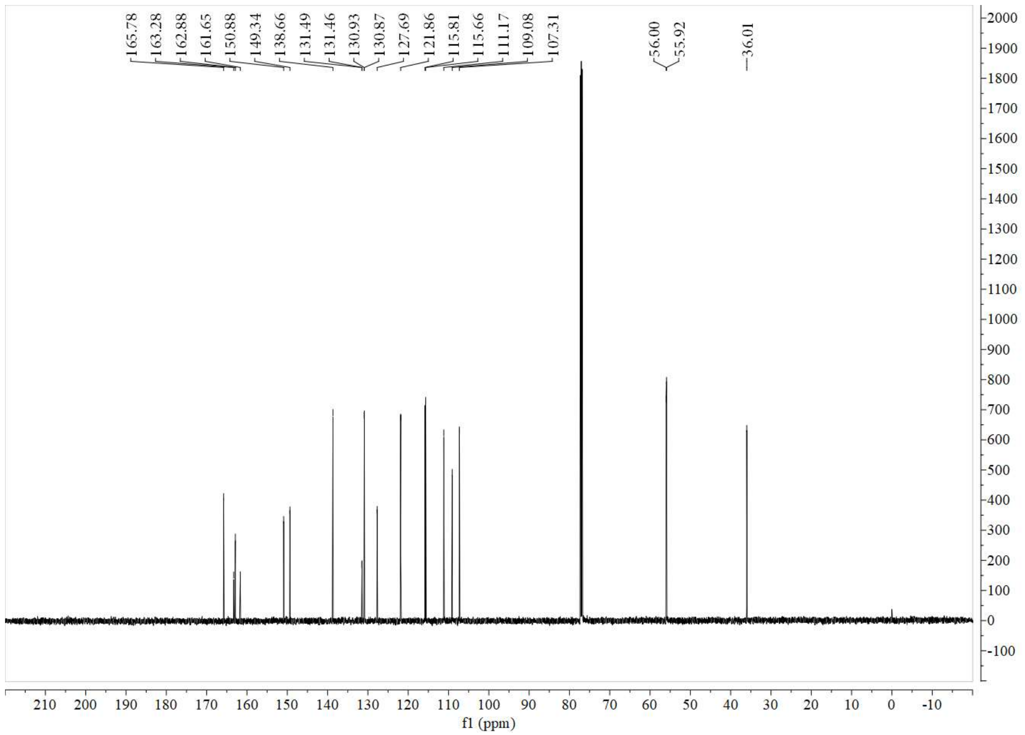

Fig. S23 The ^{13}C NMR spectrum of the target compound **6f**

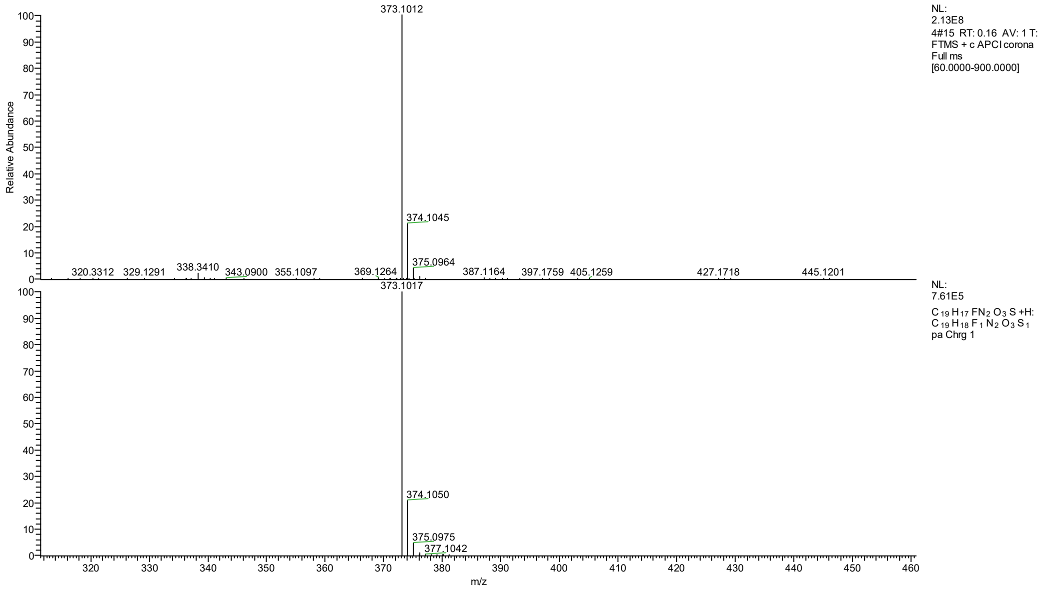

Fig. S24 The HRMS spectrum of the target compound **6f**

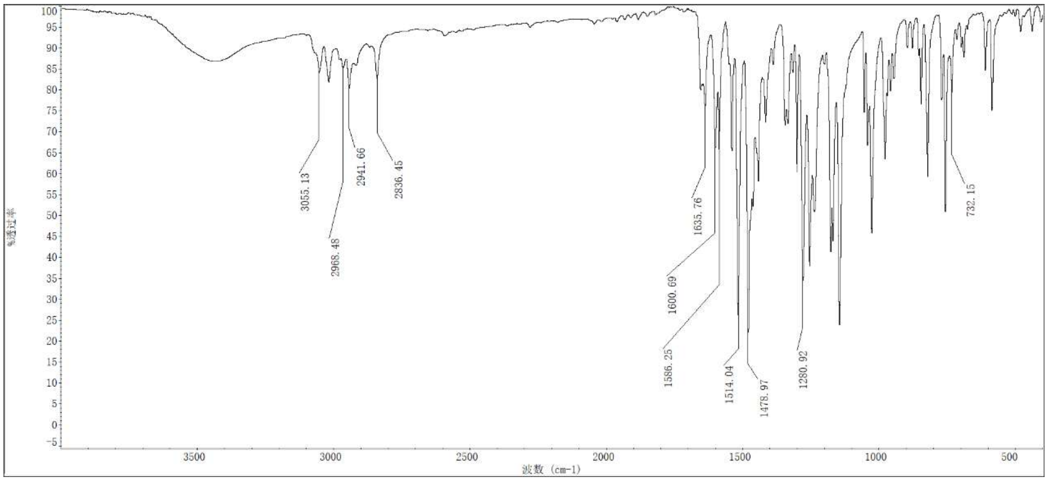

Fig. S25 The FT-IR spectrum of the target compound **6g**

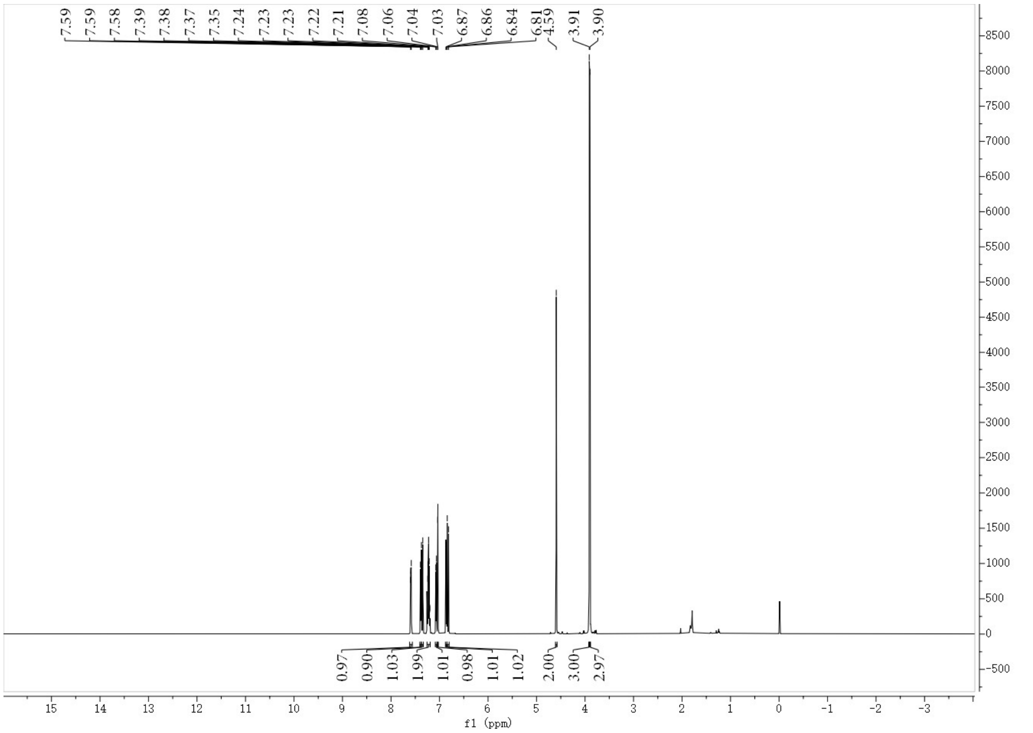

Fig. S26 The ^{1}H NMR spectrum of the target compound **6g**

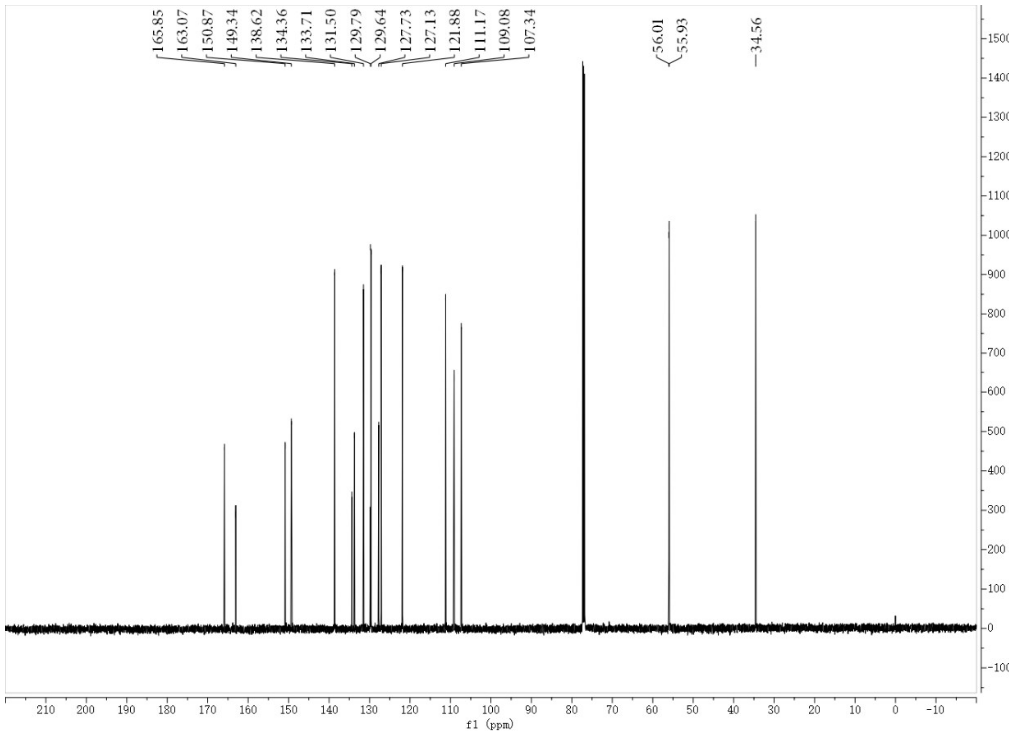

Fig. S27 The ^{13}C NMR spectrum of the target compound **6g**

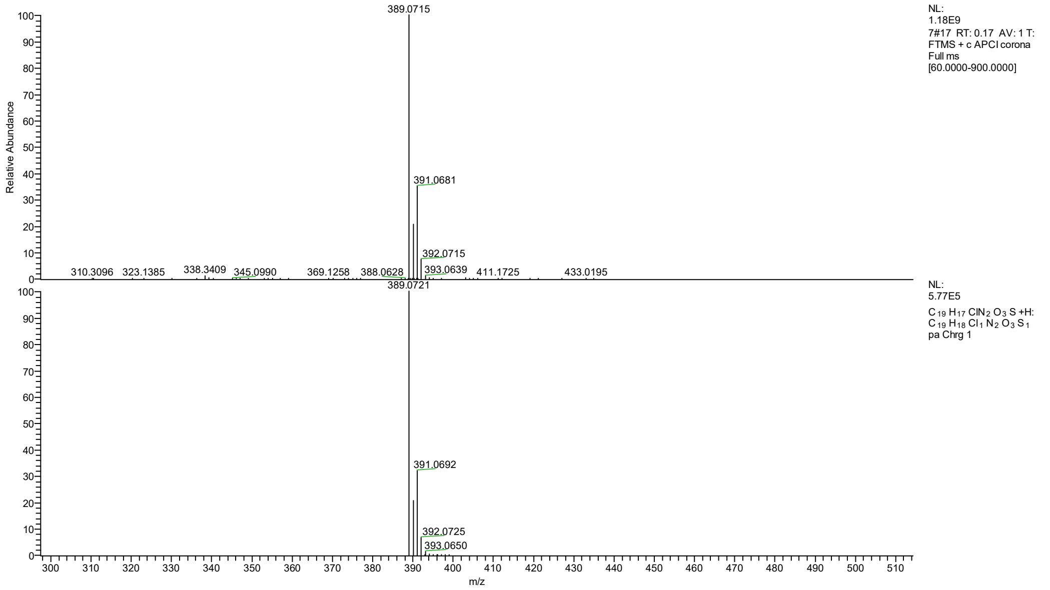

Fig. S28 The HRMS spectrum of the target compound **6g**

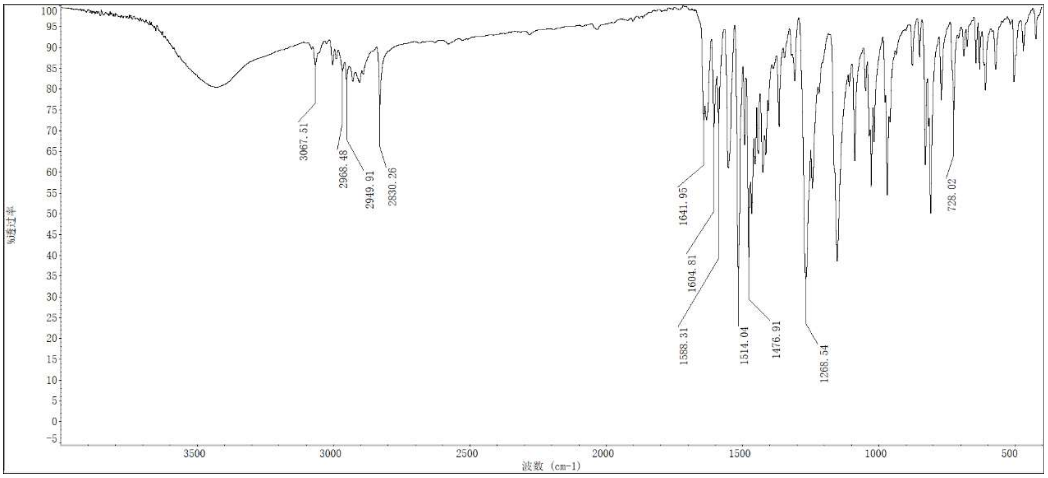

Fig. S29 The FT-IR spectrum of the target compound **6h**

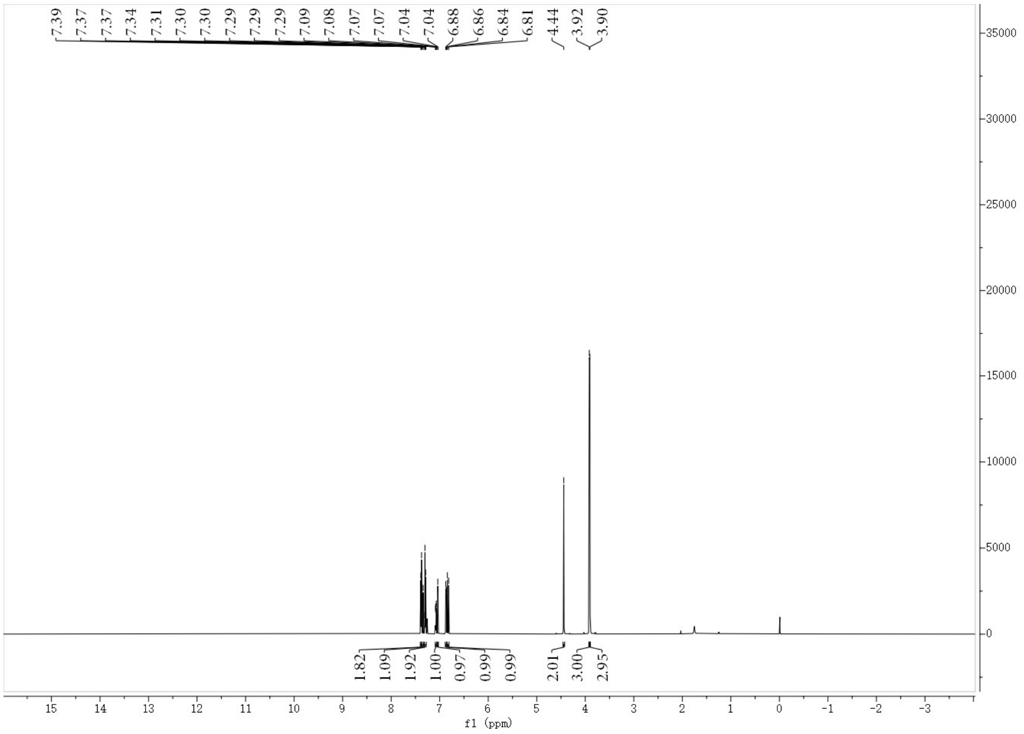

Fig. S30 The ^{1}H NMR spectrum of the target compound **6h**

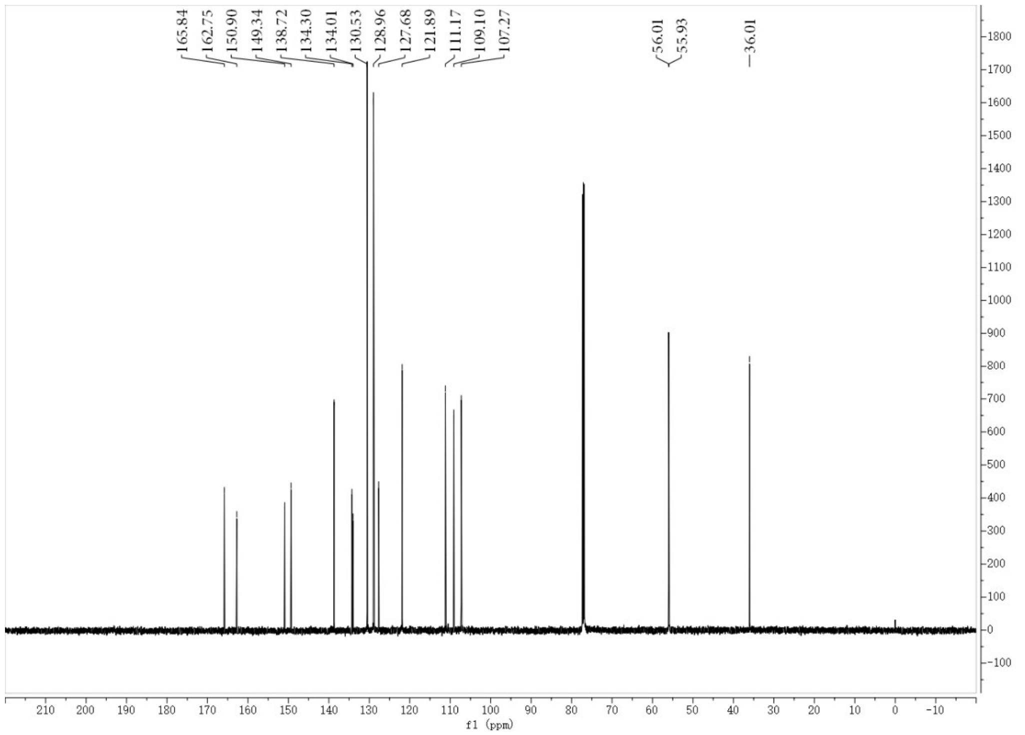

Fig. S31 The ^{13}C NMR spectrum of the target compound **6h**

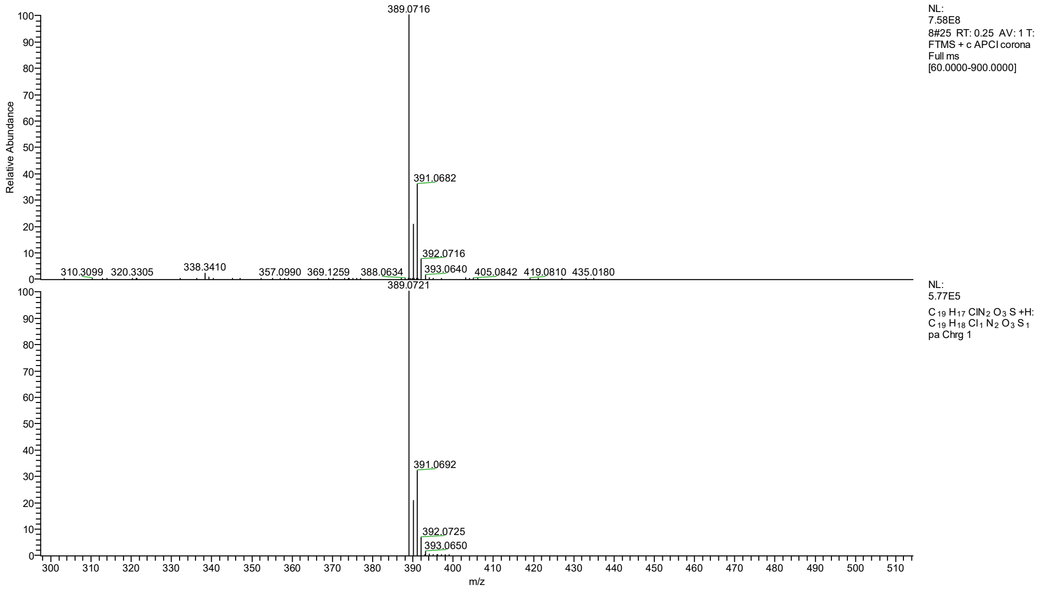

Fig. S32 The HRMS spectrum of the target compound **6h**

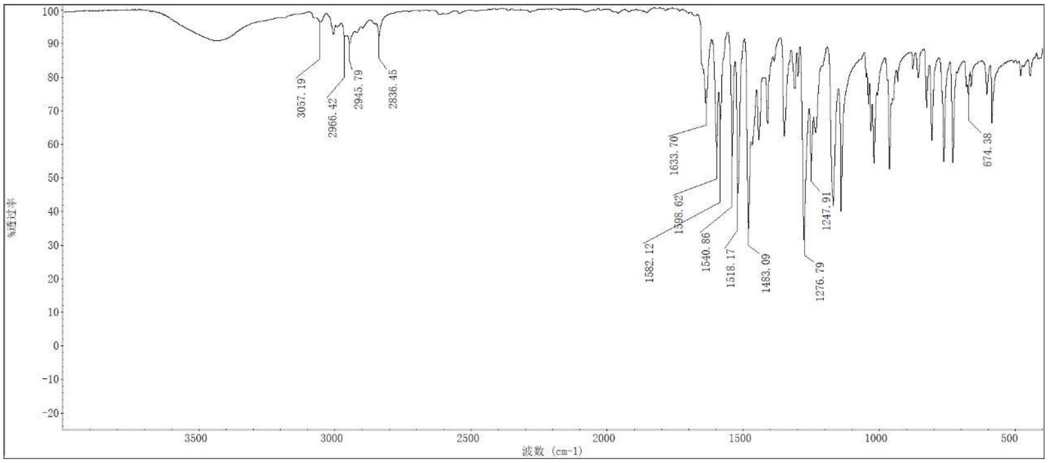

Fig. S33 The FT-IR spectrum of the target compound **6i**

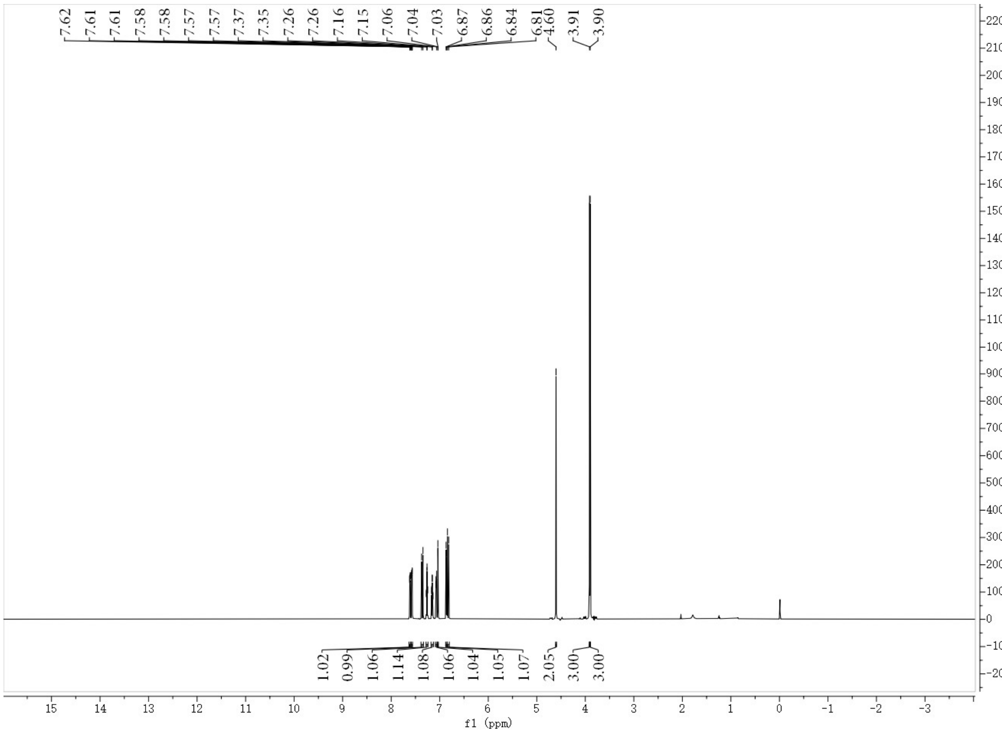

Fig. S34 The ^{1}H NMR spectrum of the target compound **6i**

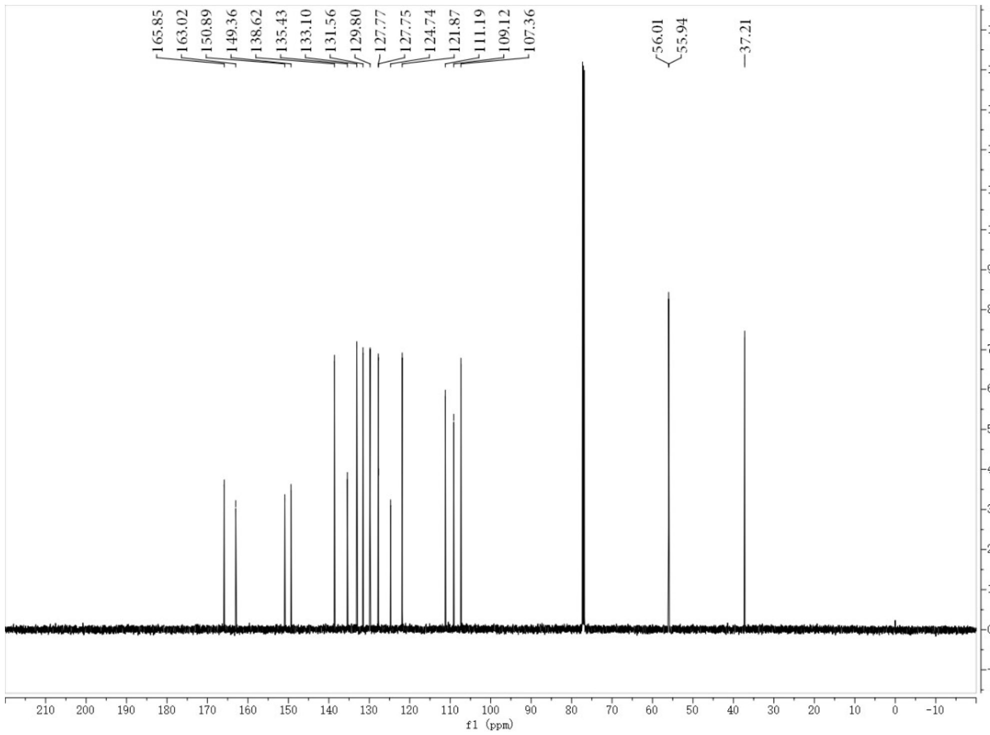

Fig. S35 The ^{13}C NMR spectrum of the target compound **6i**

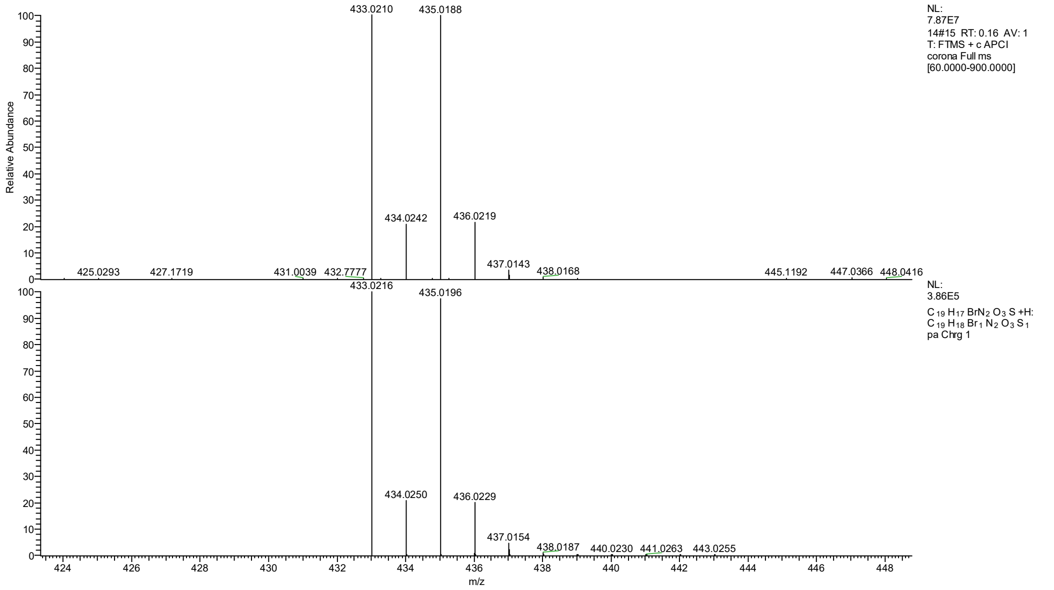

Fig. S36 The HRMS spectrum of the target compound **6i**

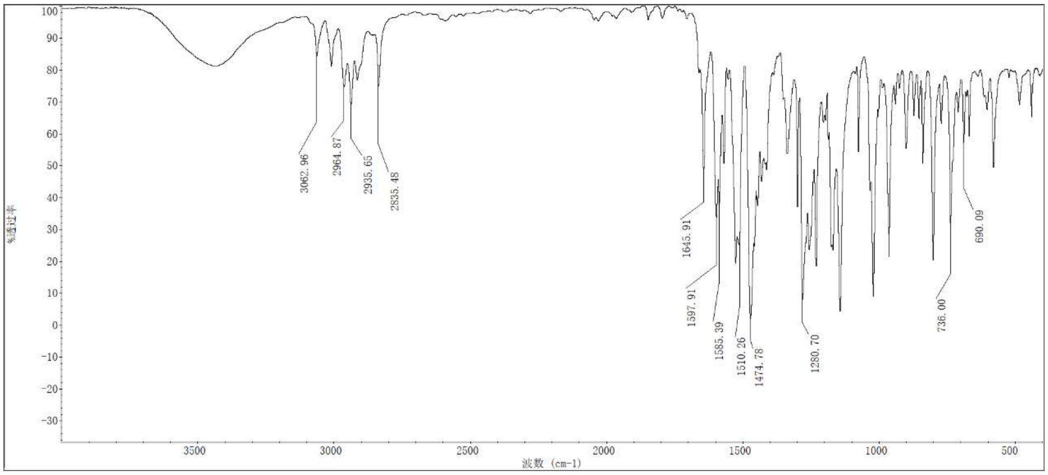

Fig. S37 The FT-IR spectrum of the target compound **6j**

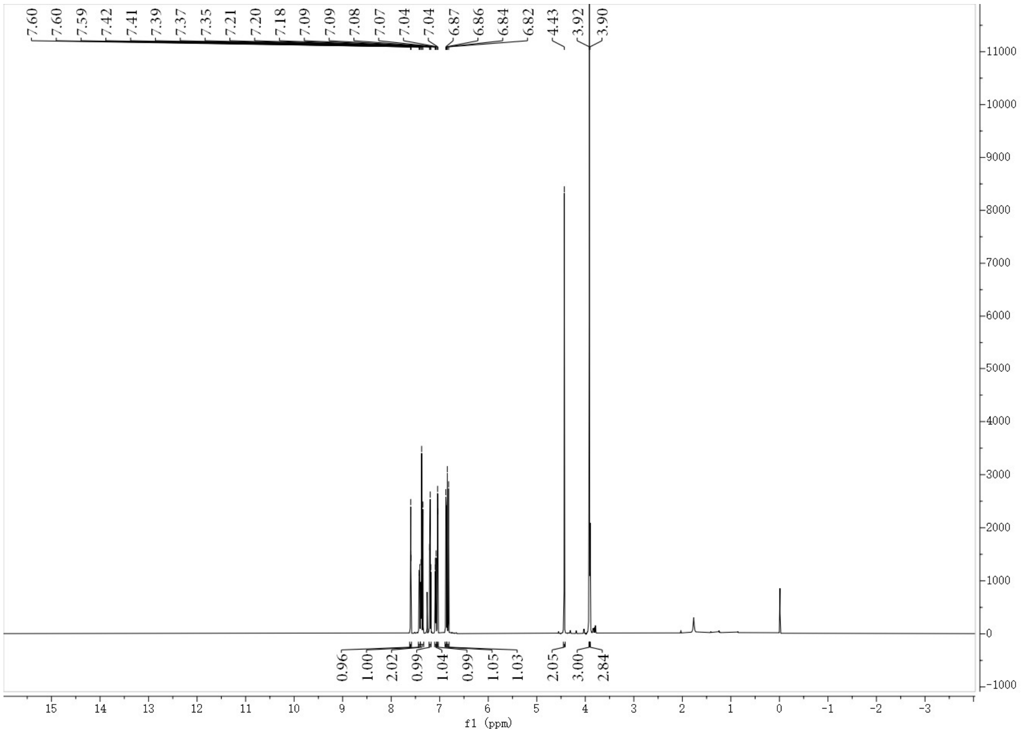

Fig. S38 The ^{1}H NMR spectrum of the target compound **6j**

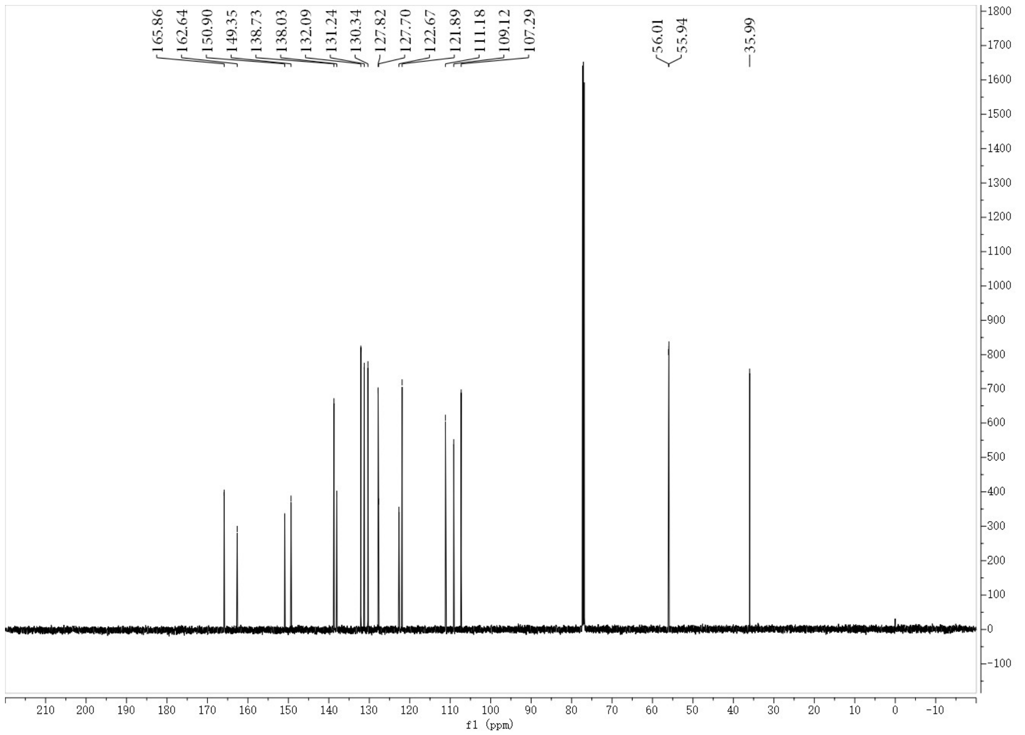

Fig. S39 The ^{13}C NMR spectrum of the target compound **6j**

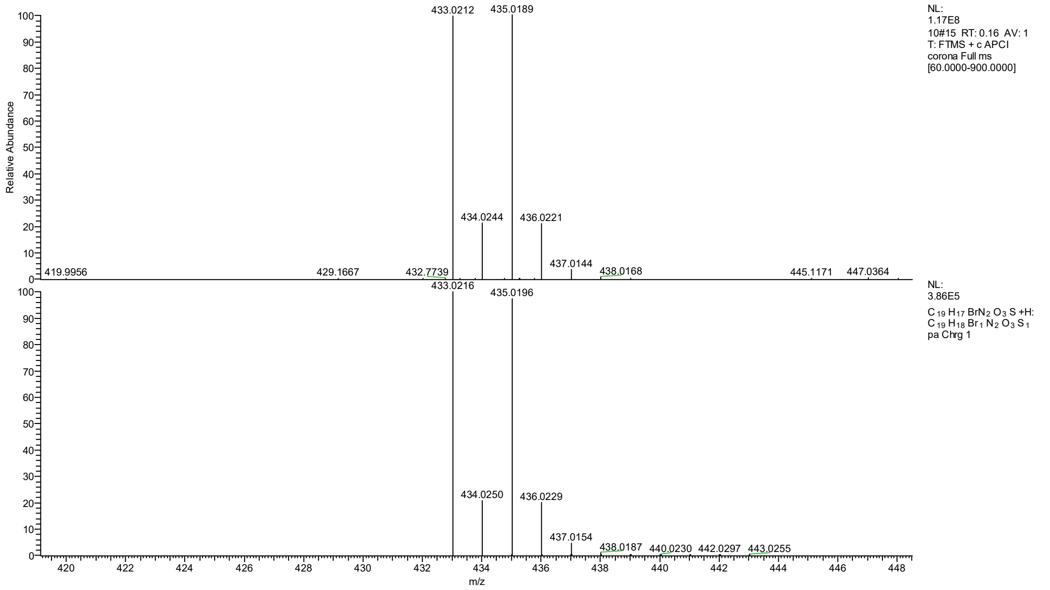

Fig. S40 The HRMS spectrum of the target compound **6j**

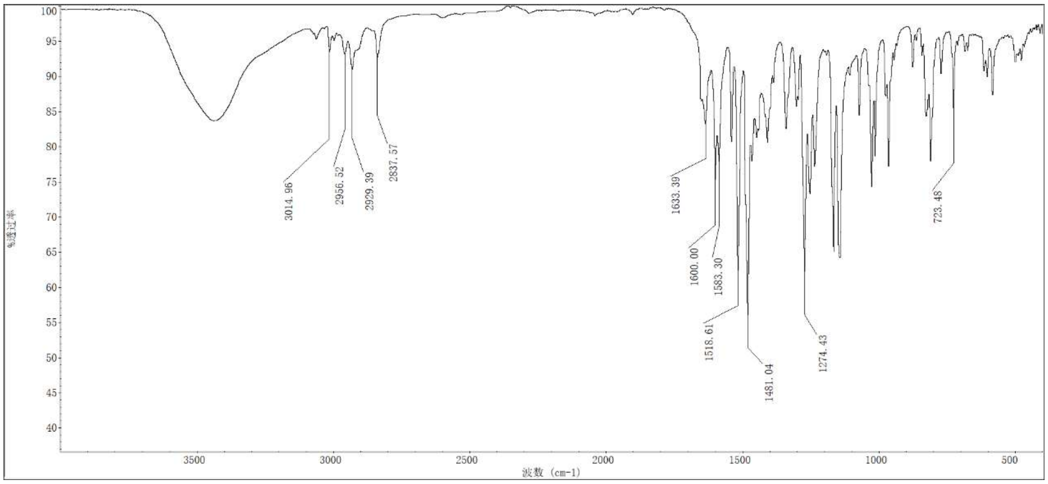

Fig. S41 The FT-IR spectrum of the target compound **6k**

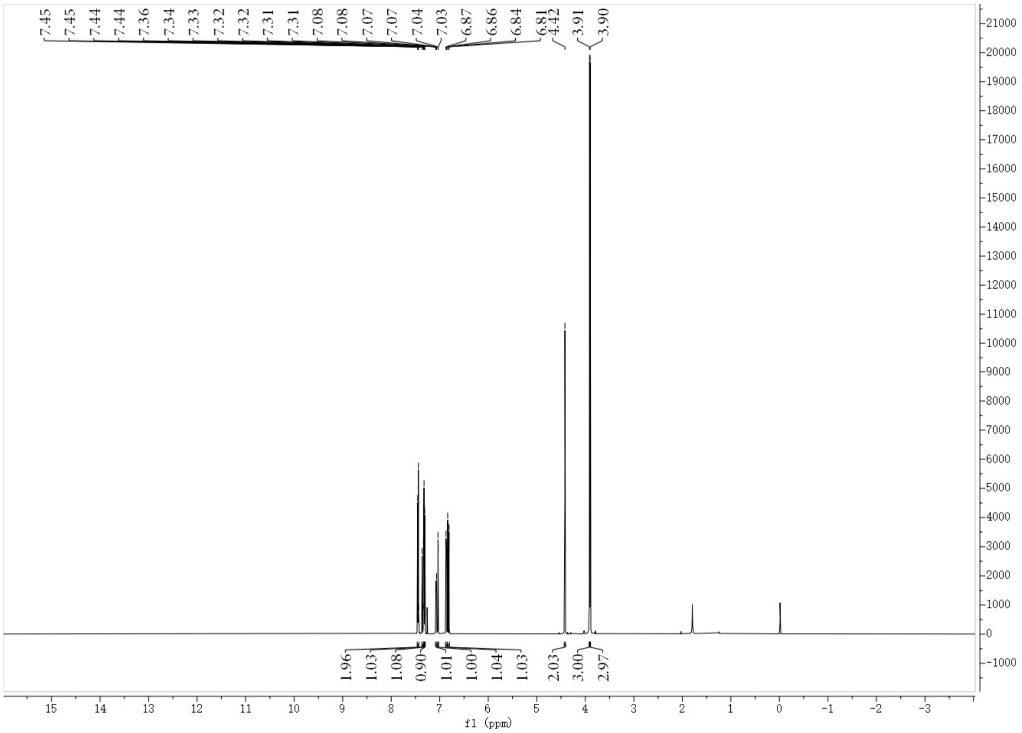

Fig. S42 The ^{1}H NMR spectrum of the target compound **6k**

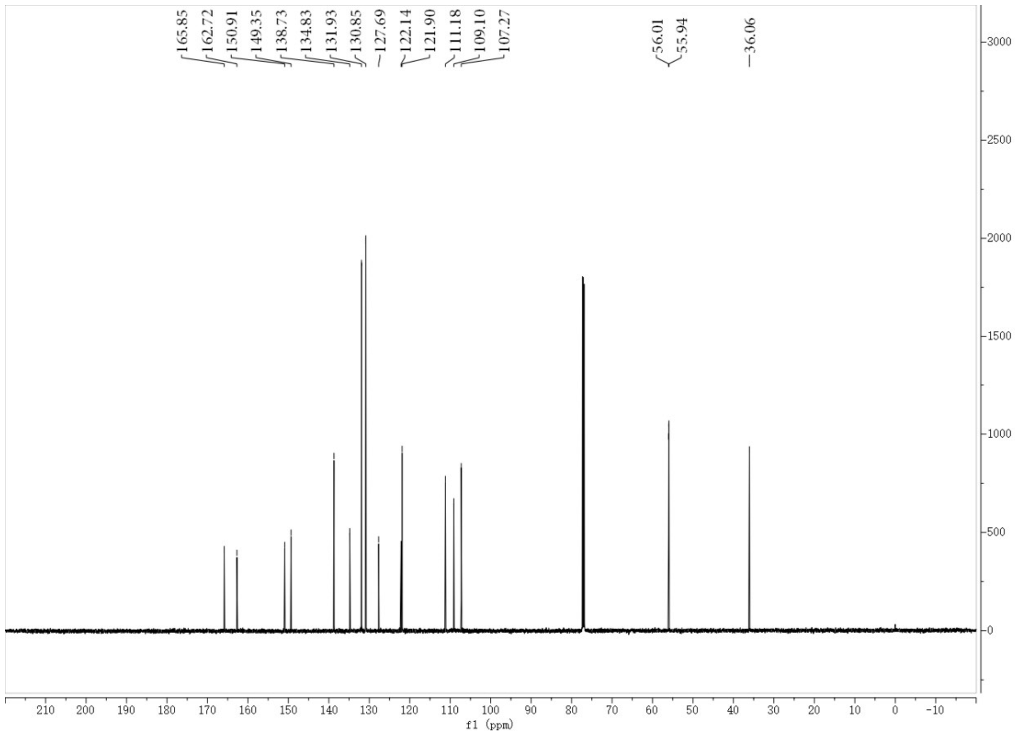

Fig. S43 The ^{13}C NMR spectrum of the target compound **6k**

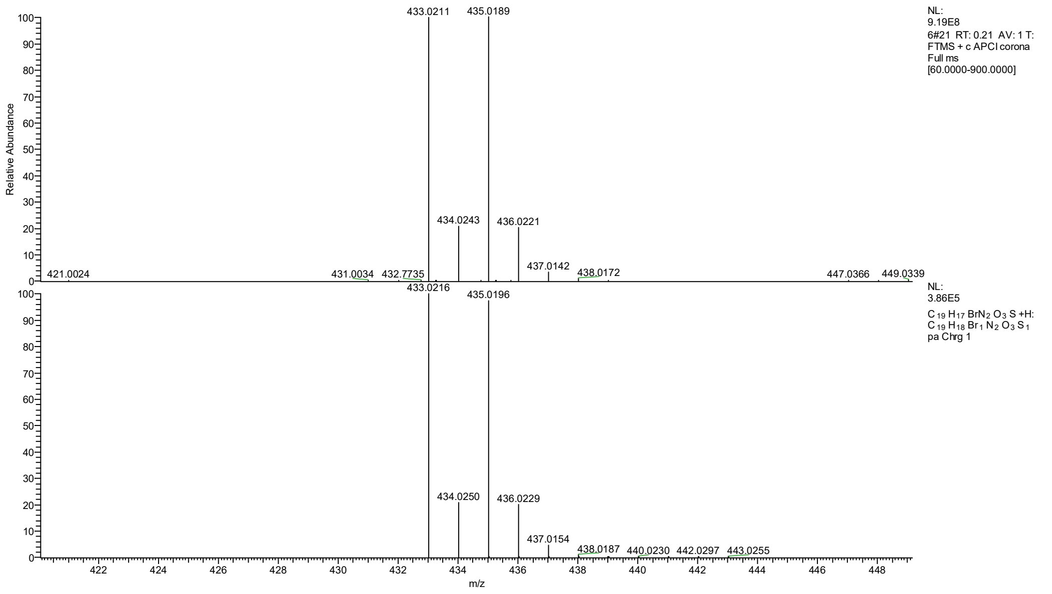

Fig. S44 The HRMS spectrum of the target compound **6k**

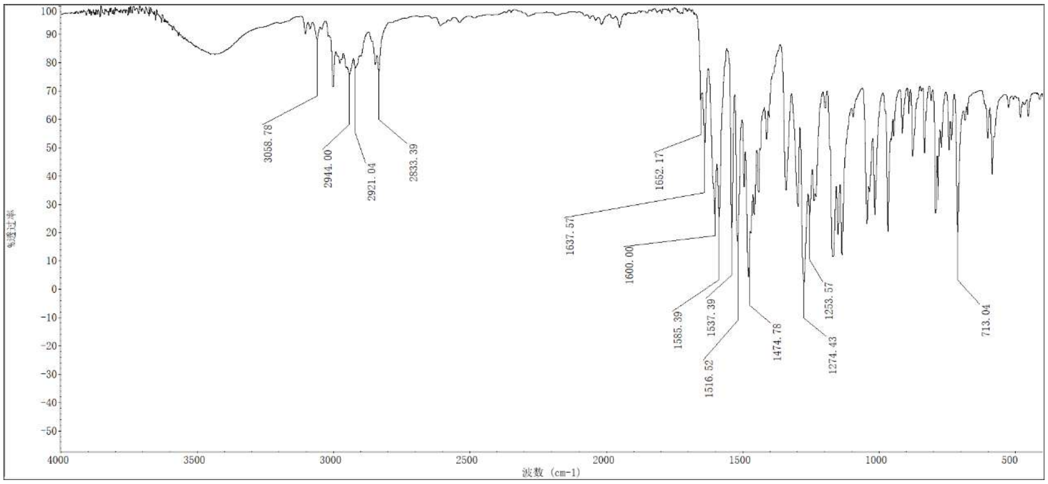

Fig. S45 The FT-IR spectrum of the target compound **6l**

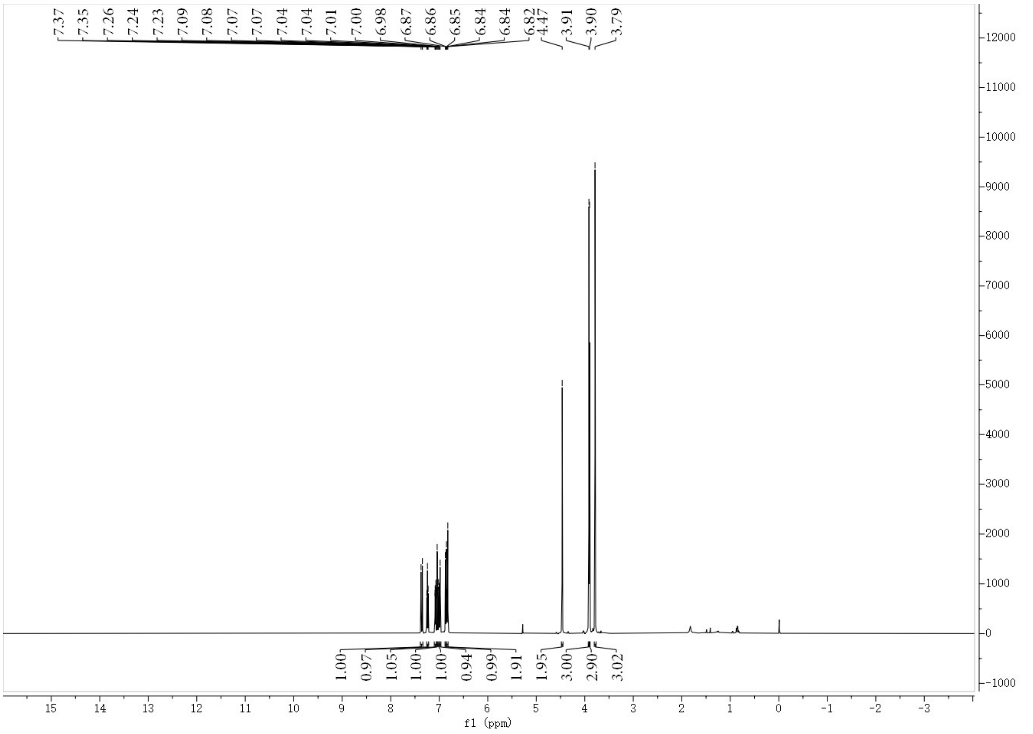

Fig. S46 The ^{1}H NMR spectrum of the target compound **6l**

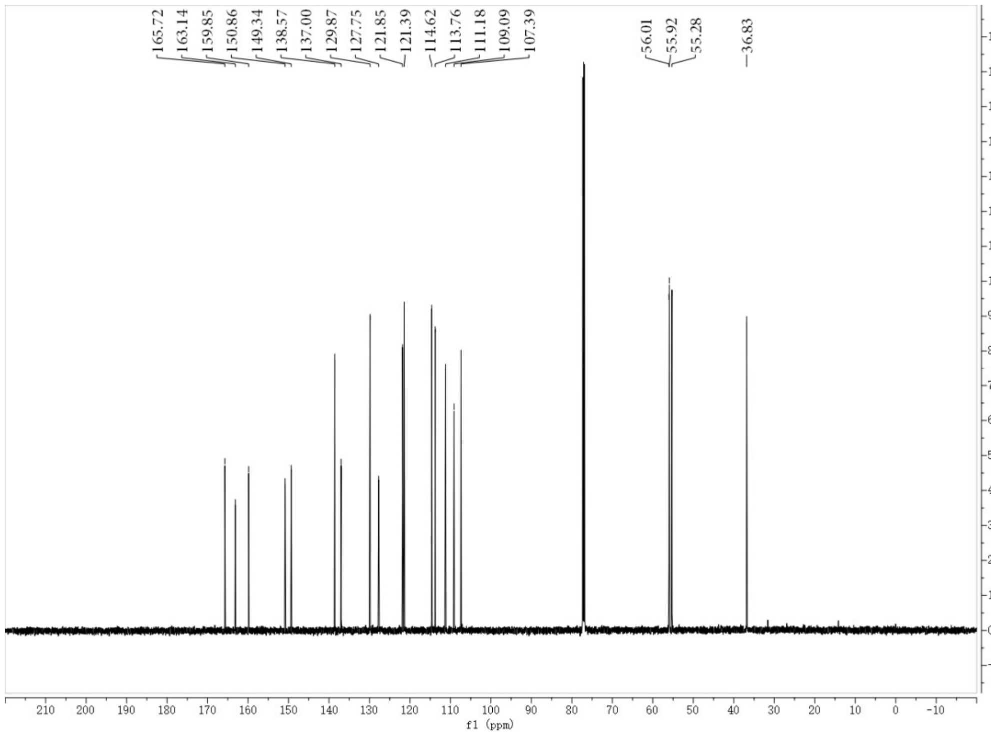

Fig. S47 The ^{13}C NMR spectrum of the target compound **6l**

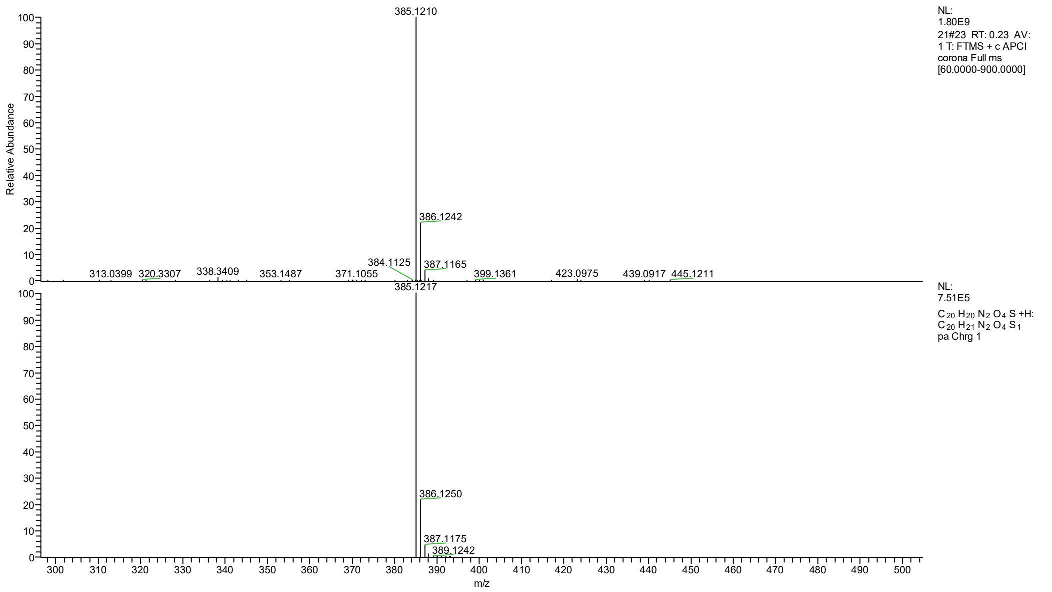

Fig. S48 The HRMS spectrum of the target compound **6l**

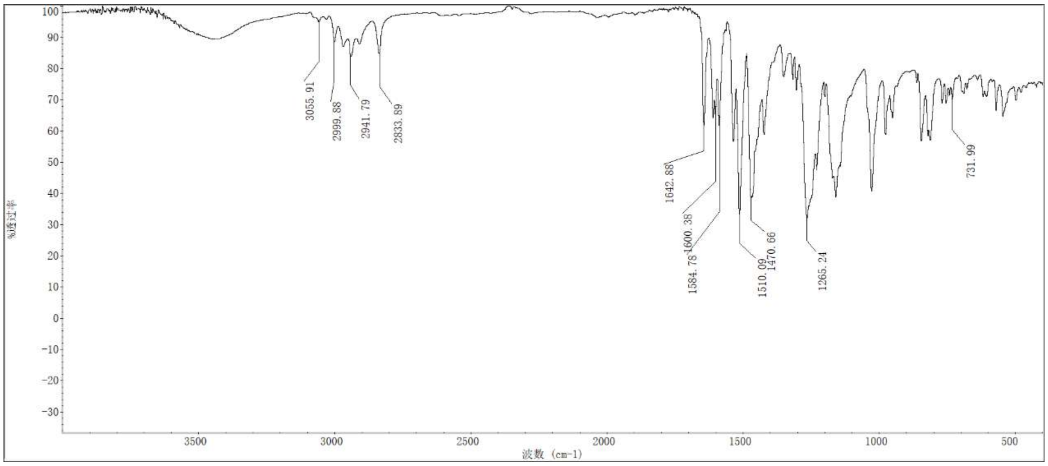

Fig. S49 The FT-IR spectrum of the target compound **6m**

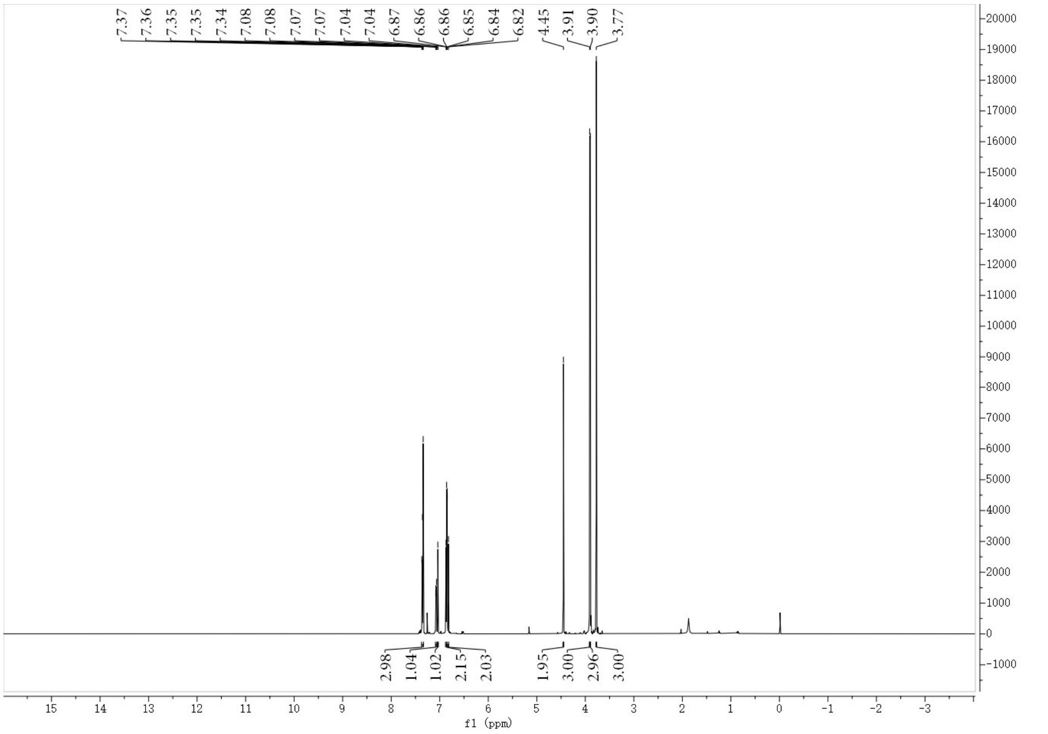

Fig. S50 The ^{1}H NMR spectrum of the target compound **6m**

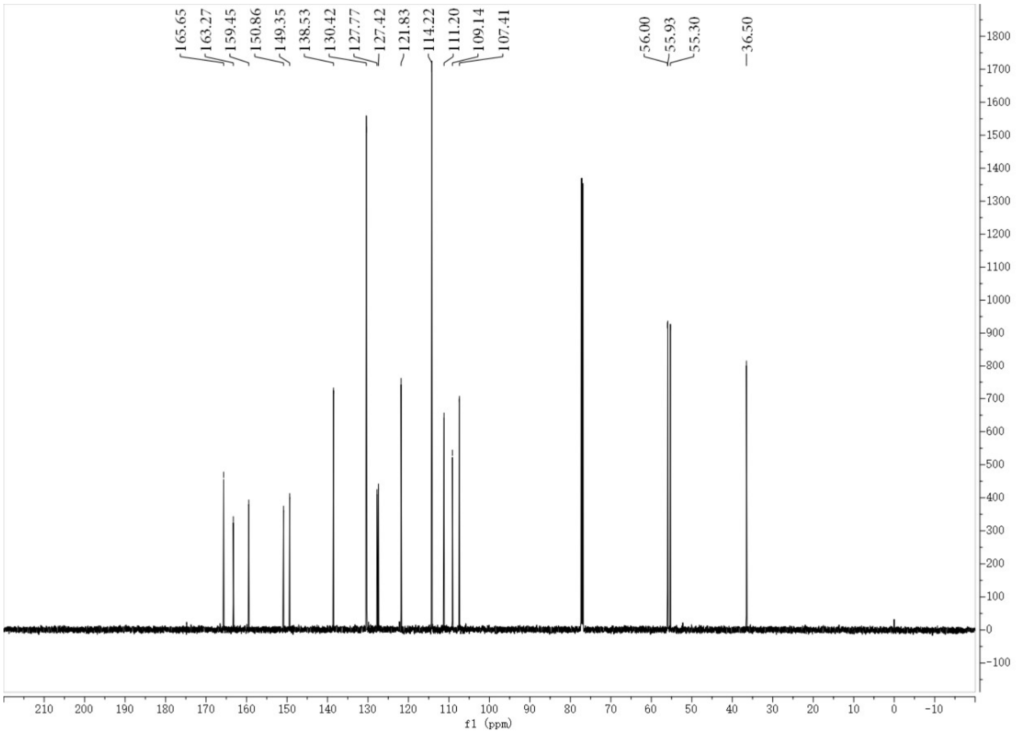

Fig. S51 The ^{13}C NMR spectrum of the target compound **6m**

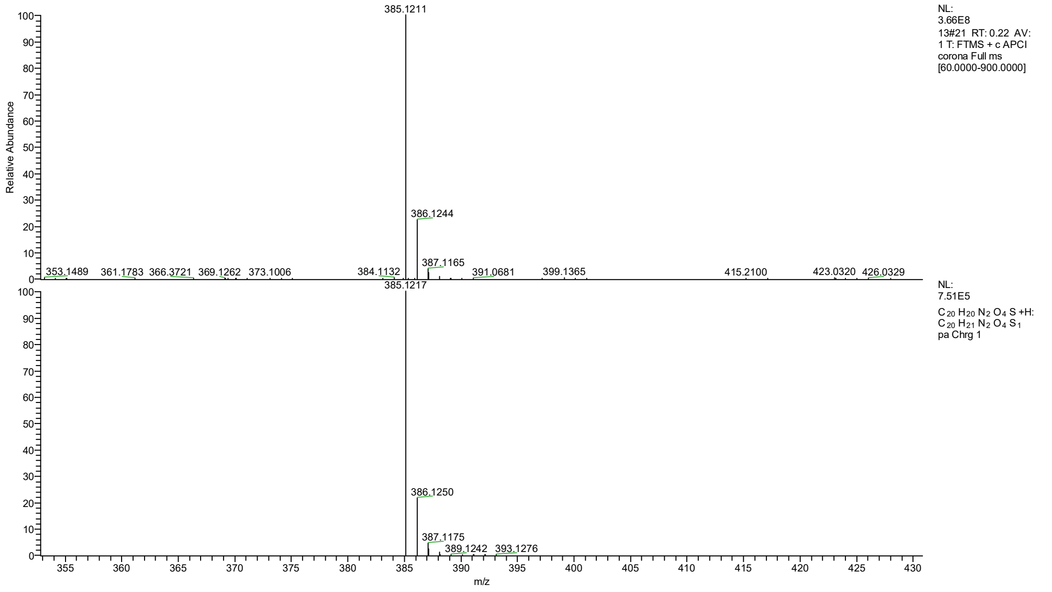

Fig. S52 The HRMS spectrum of the target compound **6m**

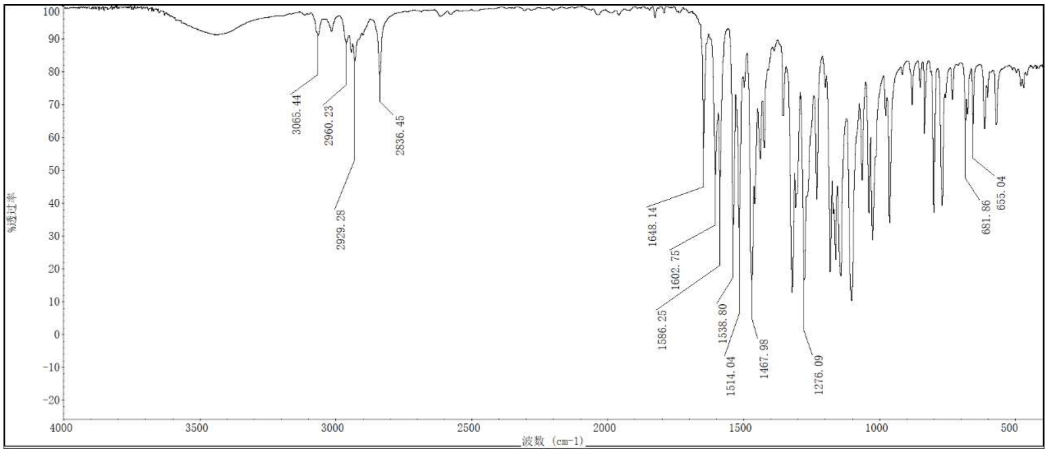

Fig. S53 The FT-IR spectrum of the target compound **6n**

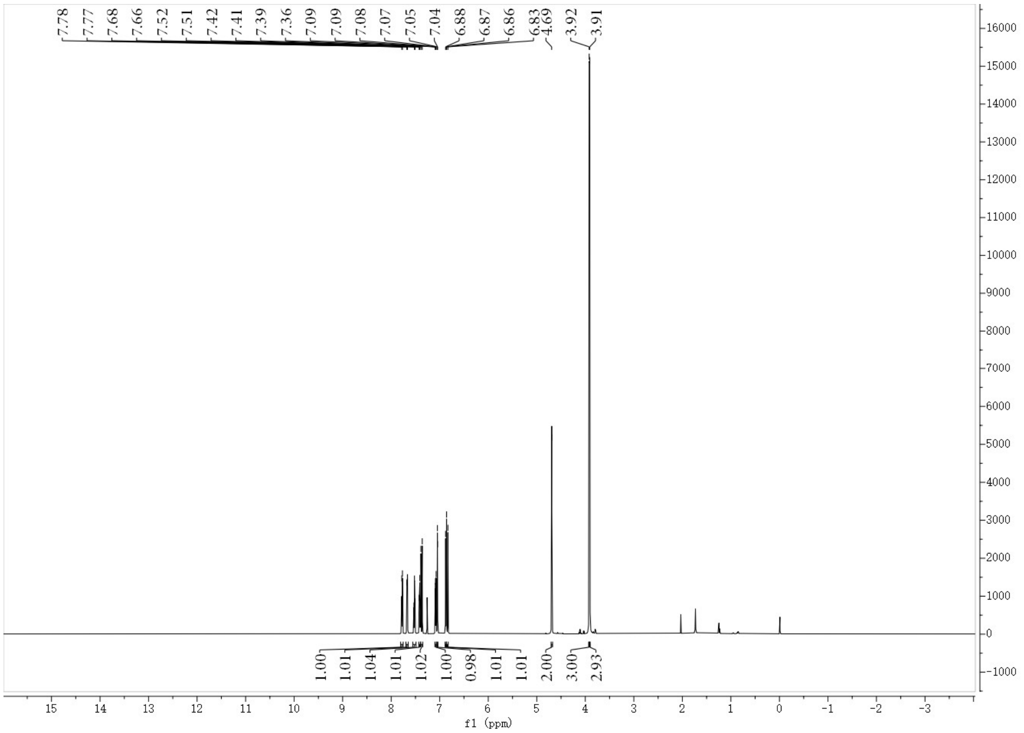

Fig. S54 The ^{1}H NMR spectrum of the target compound **6n**

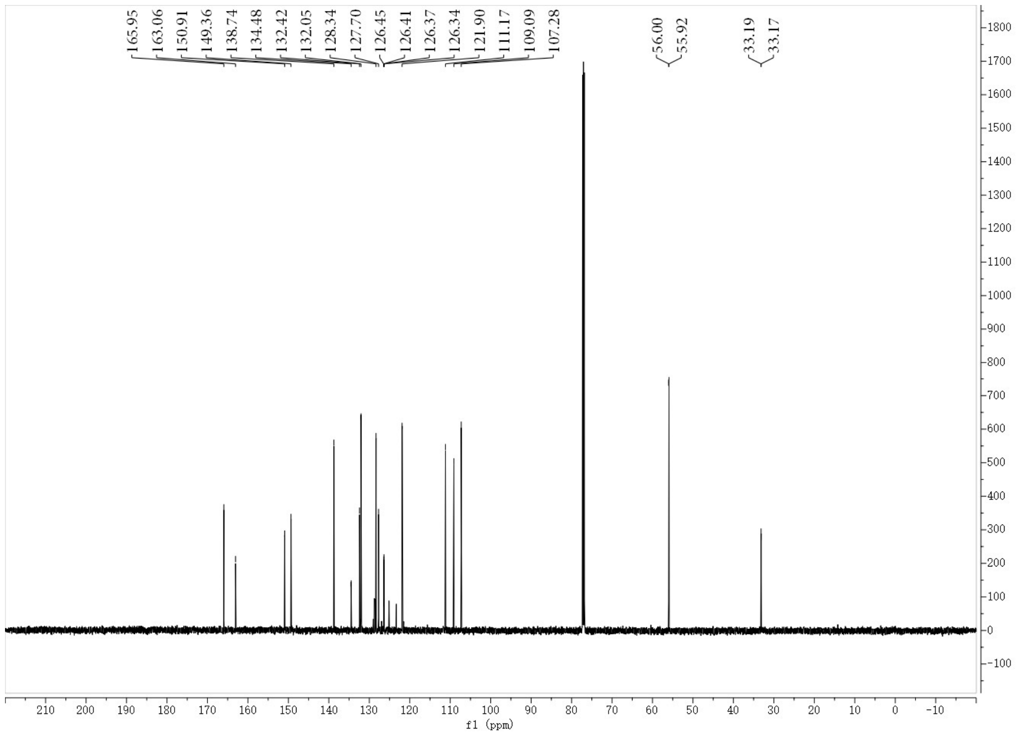

Fig. S55 The ^{13}C NMR spectrum of the target compound **6n**

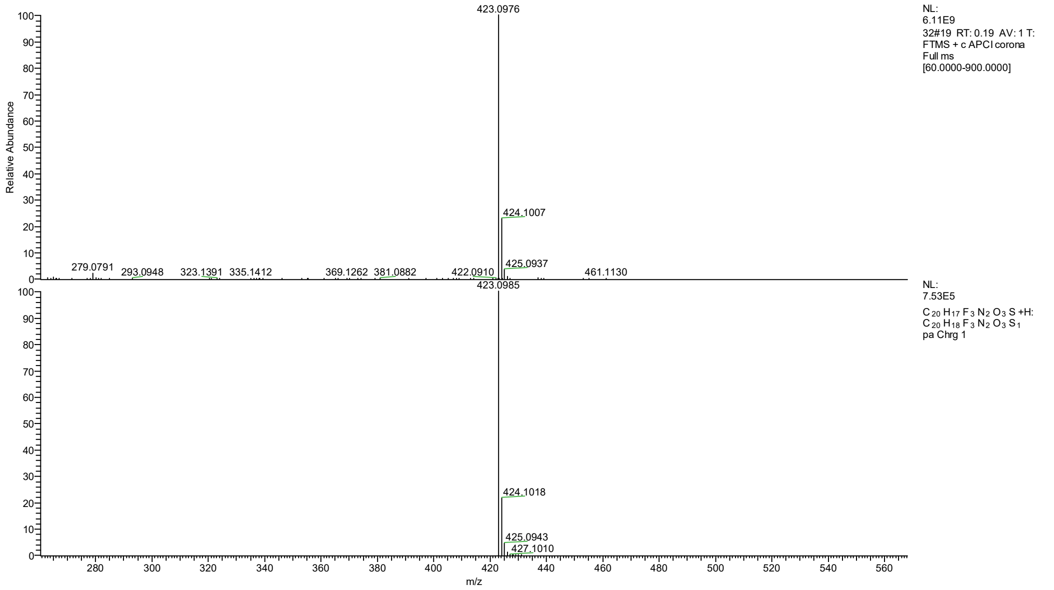

Fig. S56 The HRMS spectrum of the target compound **6n**

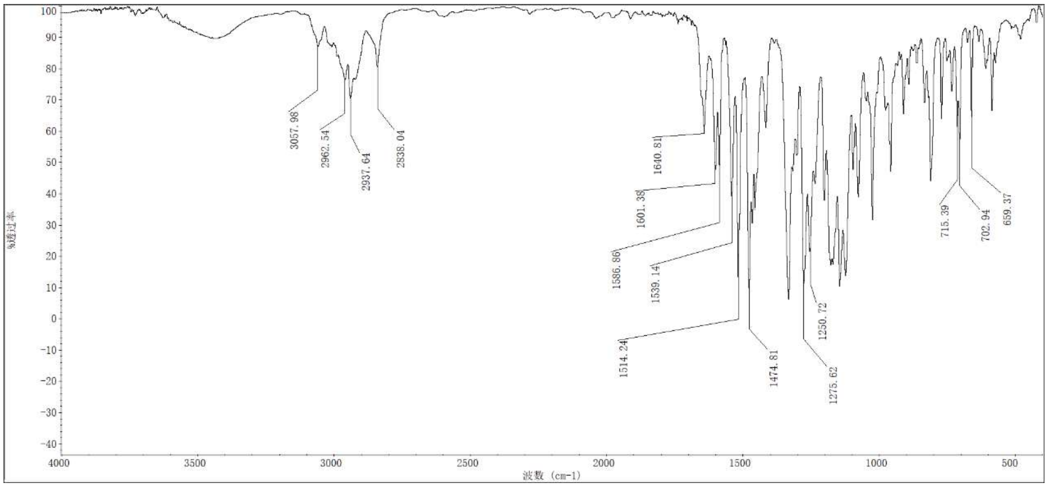

Fig. S57 The FT-IR spectrum of the target compound **6o**

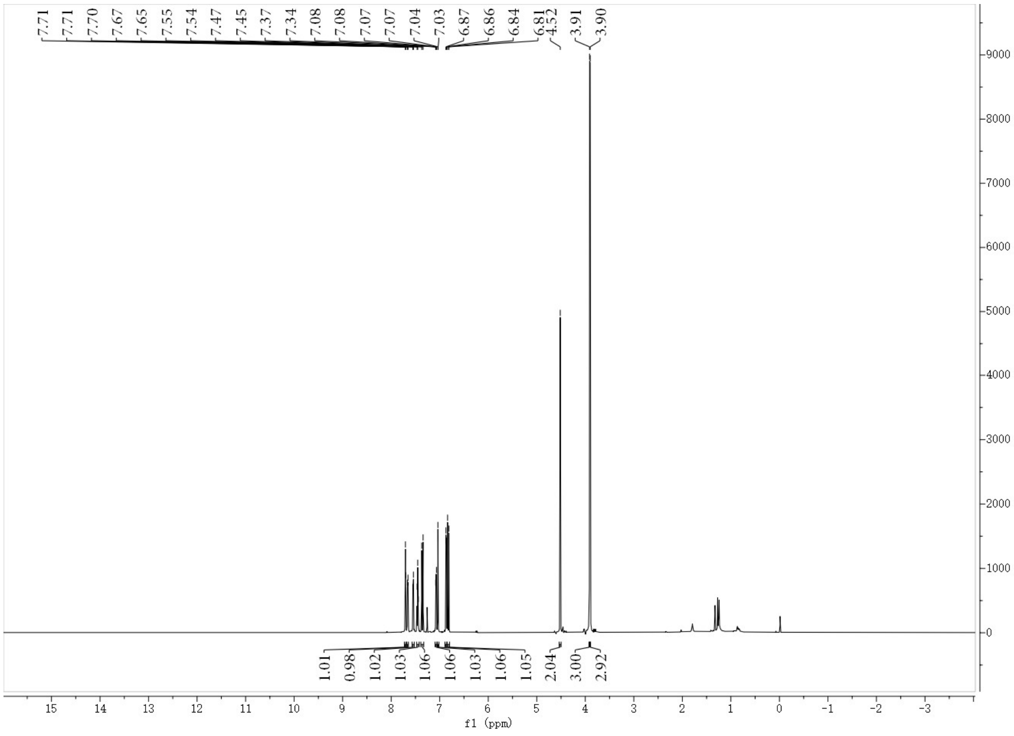

Fig. S58 The ^{1}H NMR spectrum of the target compound **6o**

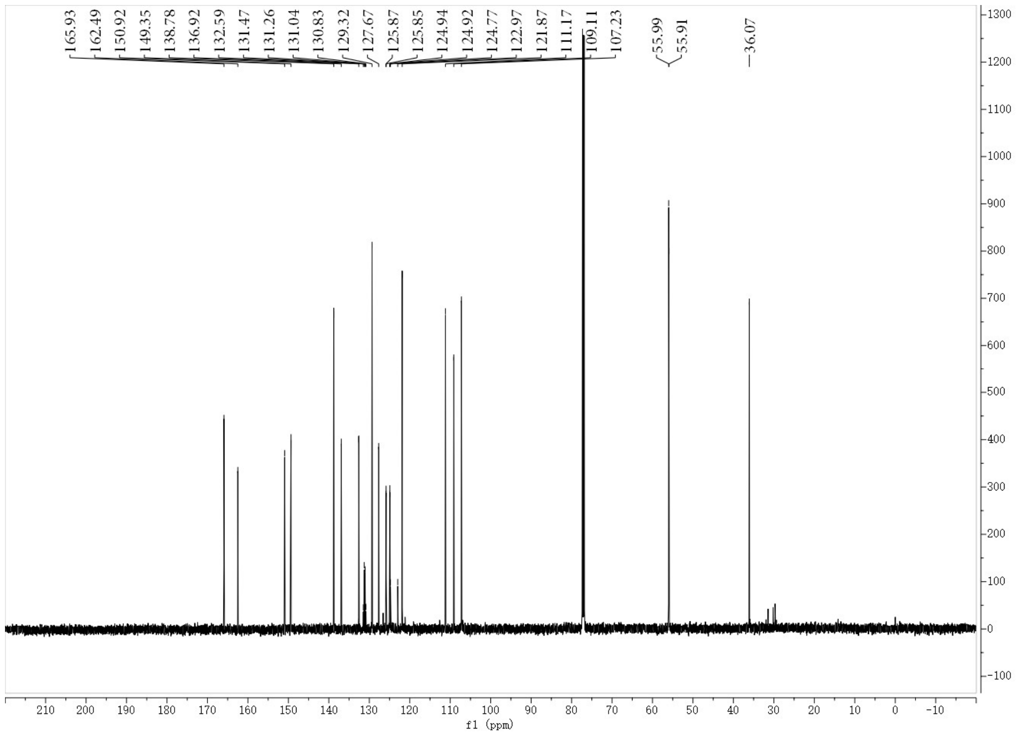

Fig. S59 The ^{13}C NMR spectrum of the target compound **6o**

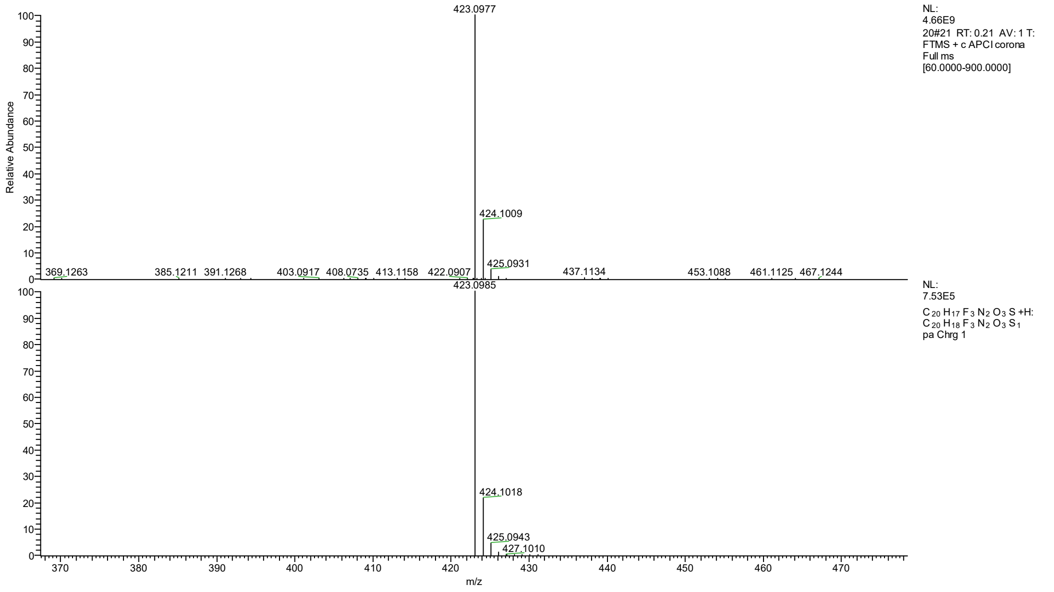

Fig. S60 The HRMS spectrum of the target compound **6o**

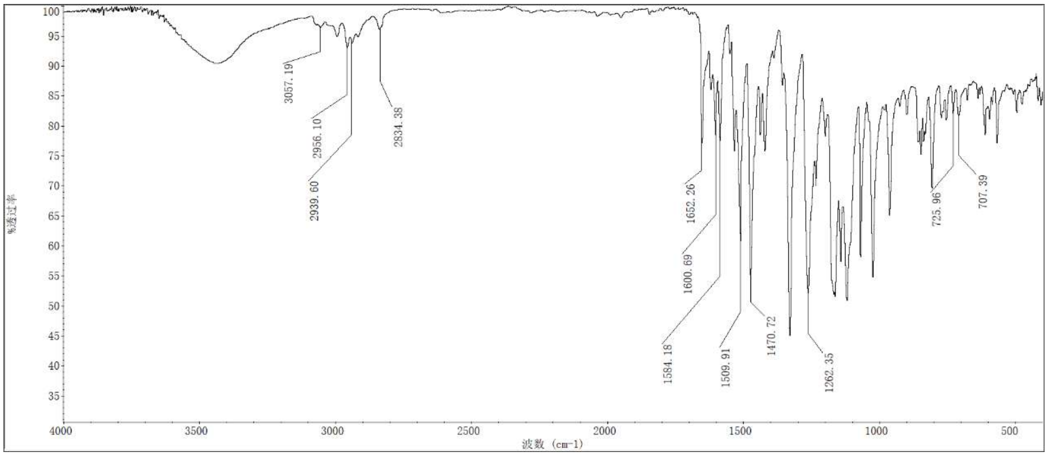

Fig. S61 The FT-IR spectrum of the target compound **6p**

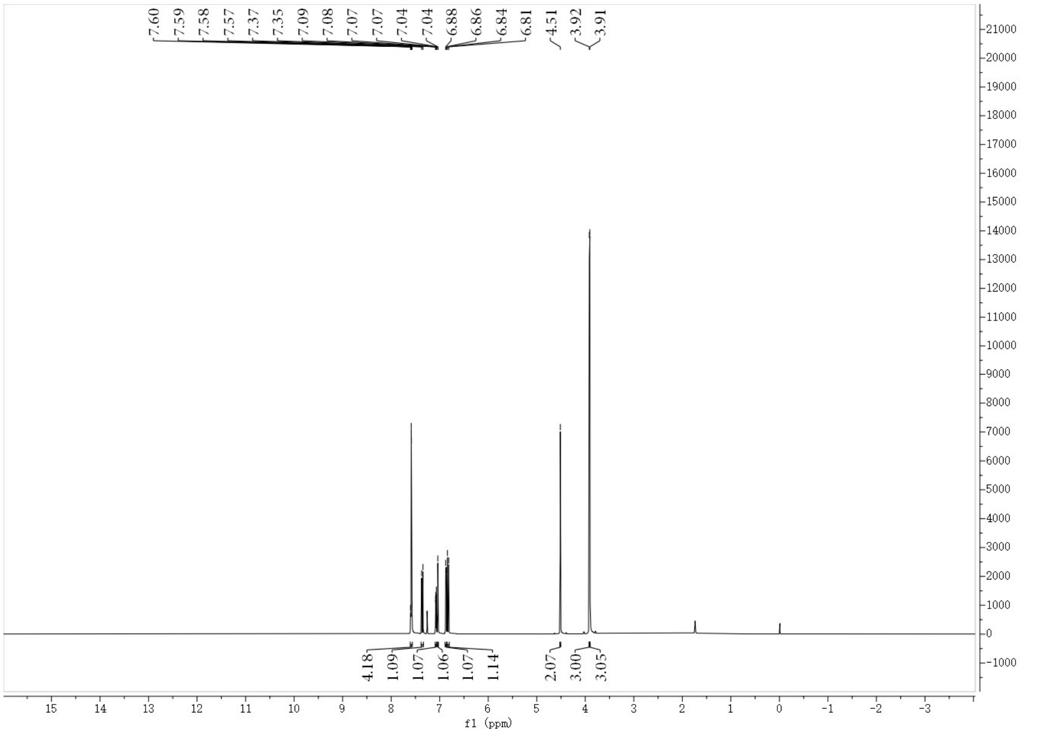

Fig. S62 The ^{1}H NMR spectrum of the target compound **6p**

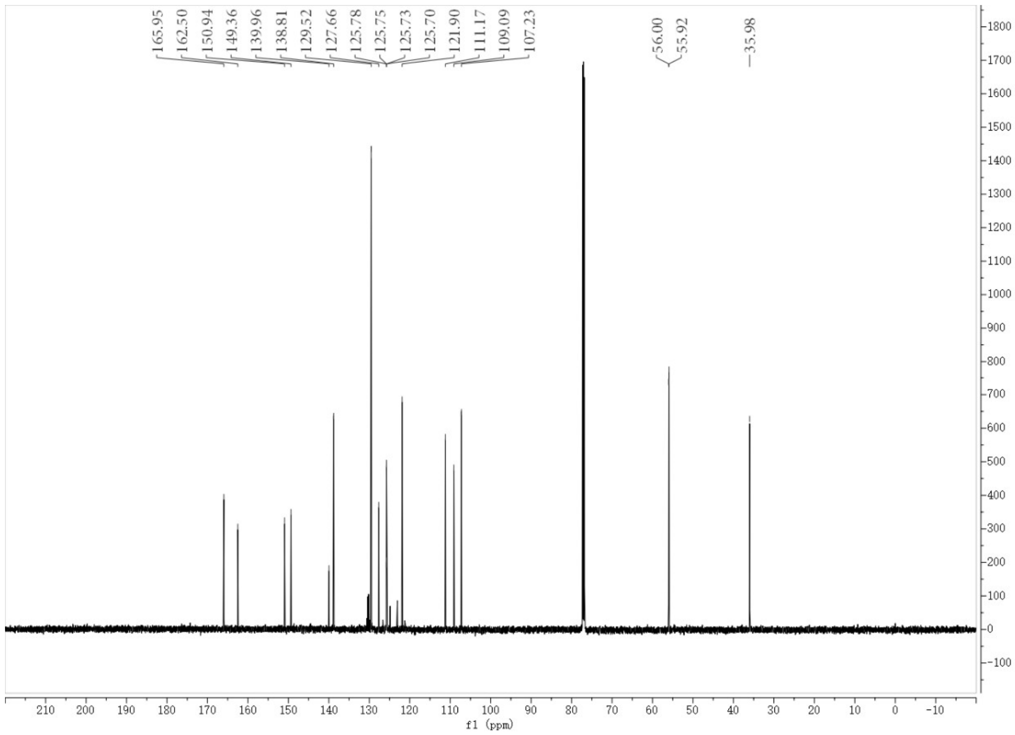

Fig. S63 The ^{13}C NMR spectrum of the target compound **6p**

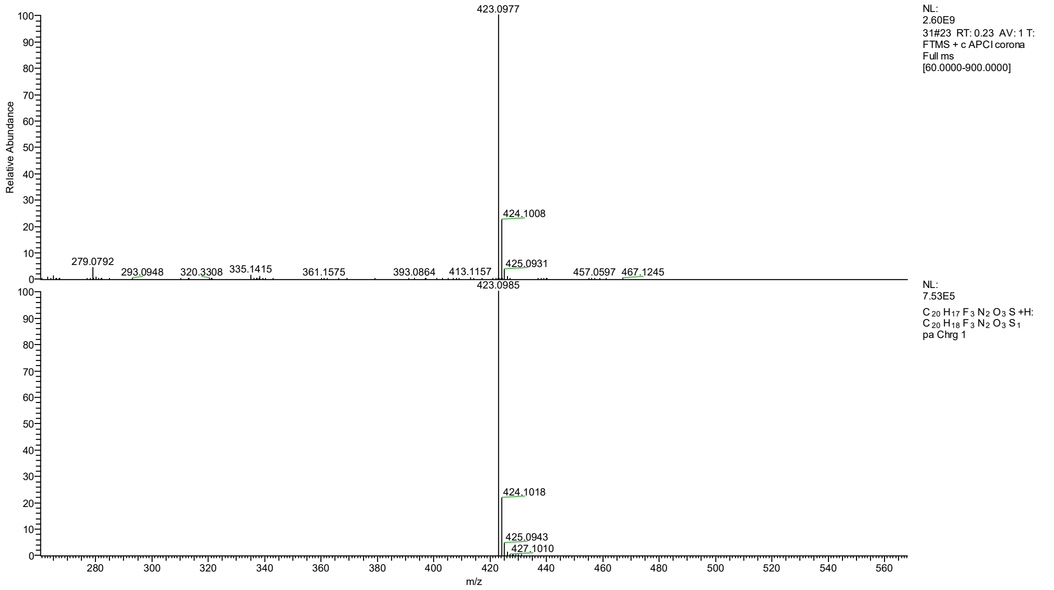

Fig. S64 The HRMS spectrum of the target compound **6p**

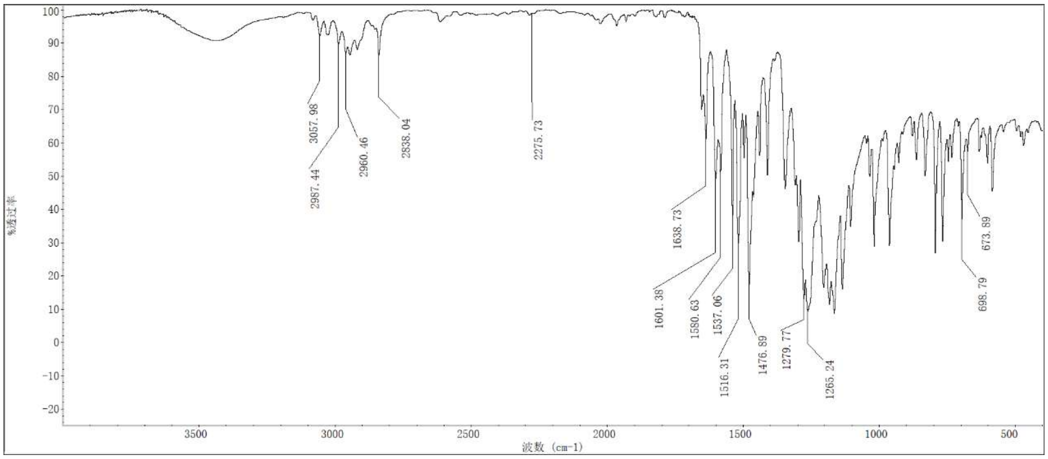

Fig. S65 The FT-IR spectrum of the target compound **6q**

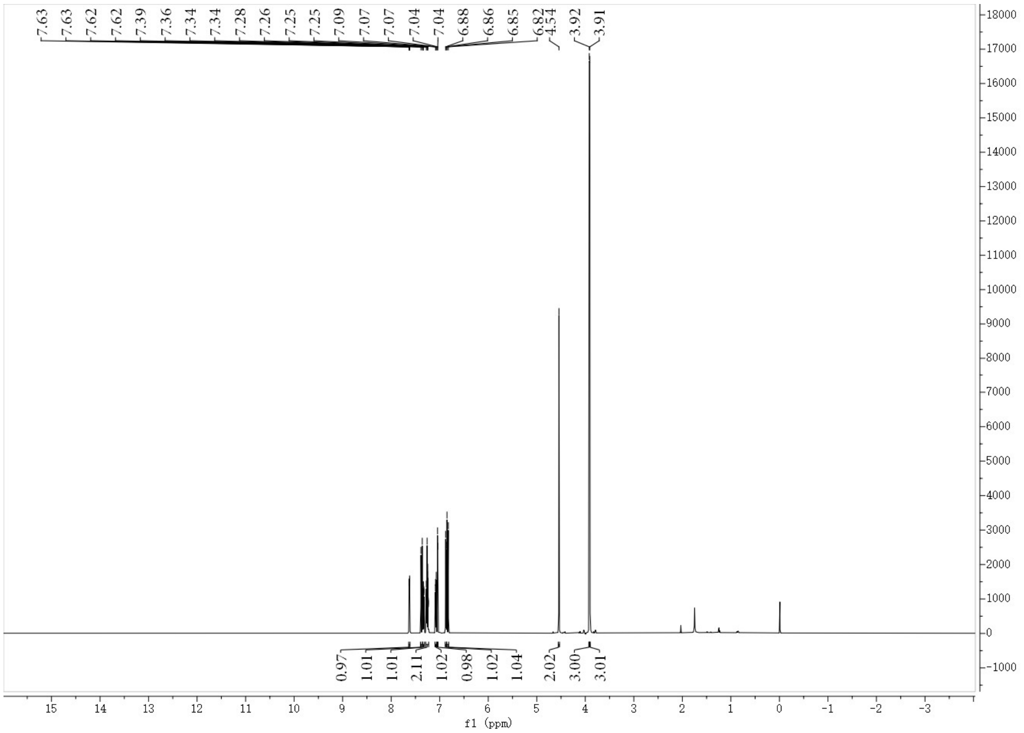

Fig. S66 The ^{1}H NMR spectrum of the target compound **6q**

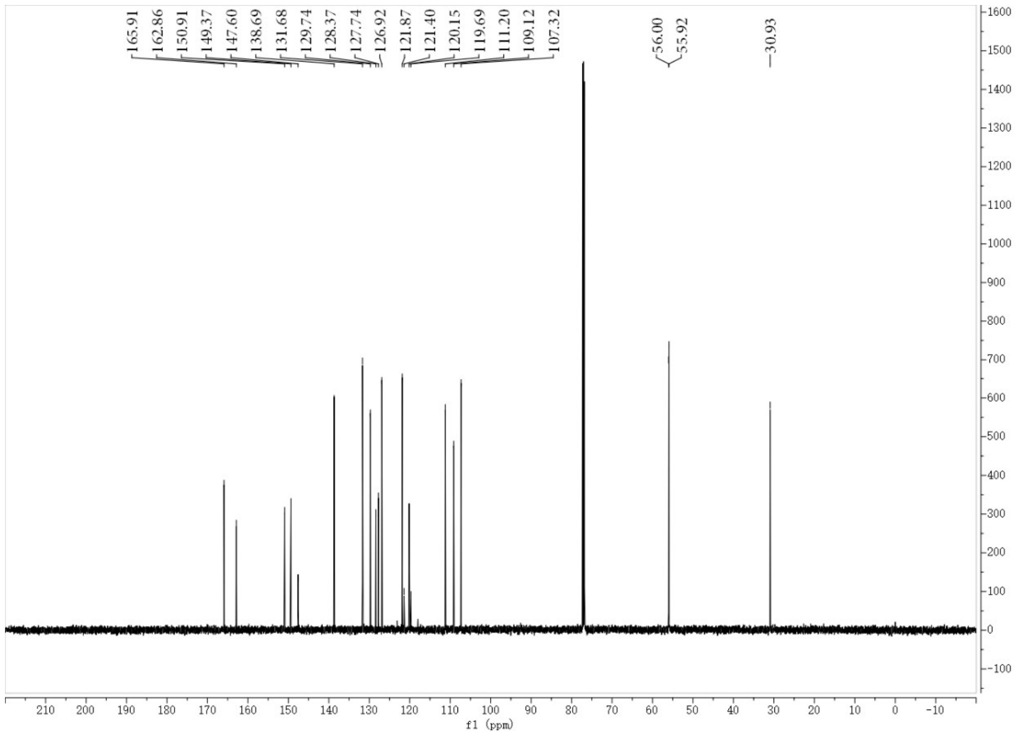

Fig. S67 The ^{13}C NMR spectrum of the target compound **6q**

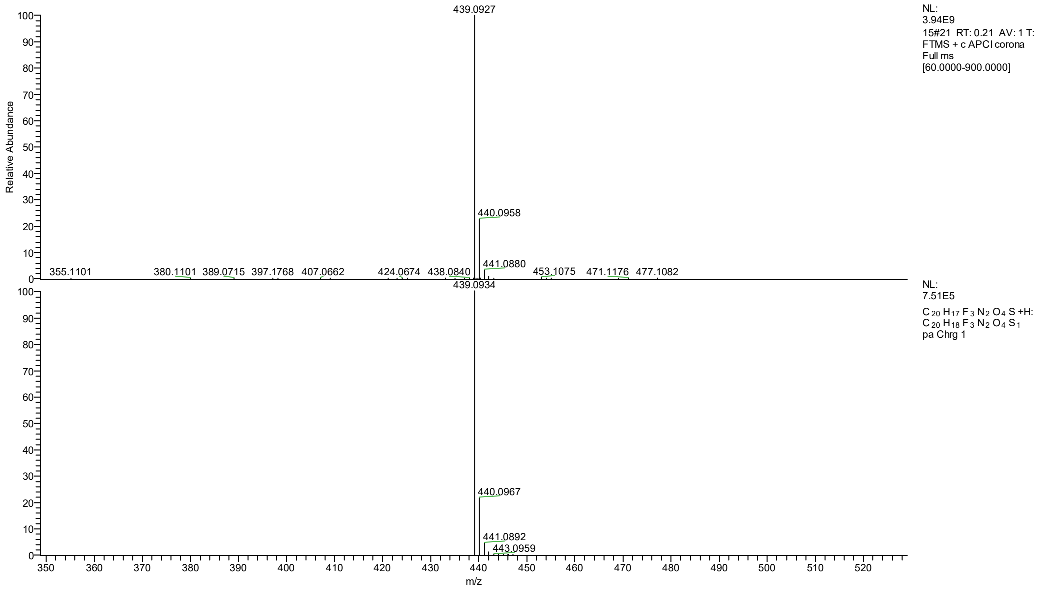

Fig. S68 The HRMS spectrum of the target compound **6q**

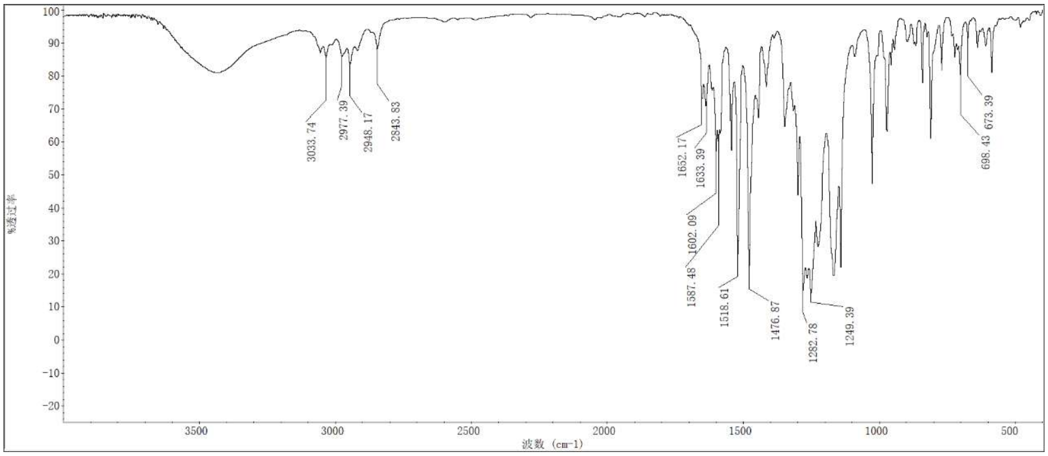

Fig. S69 The FT-IR spectrum of the target compound **6r**

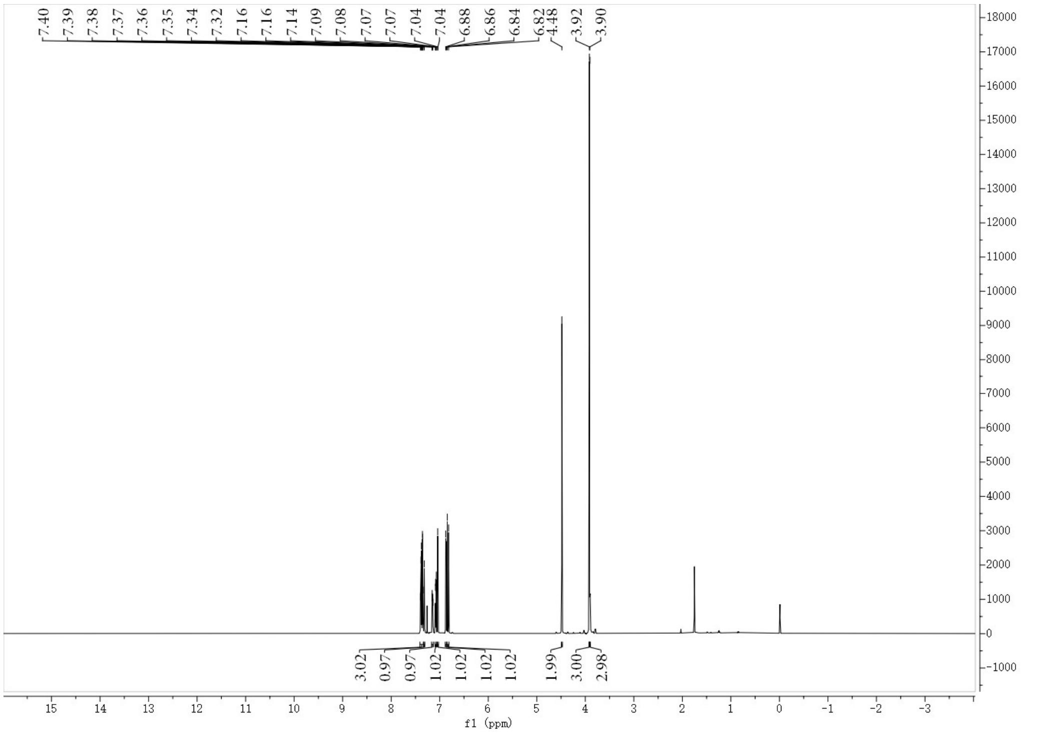

Fig. S70 The ^{1}H NMR spectrum of the target compound **6r**

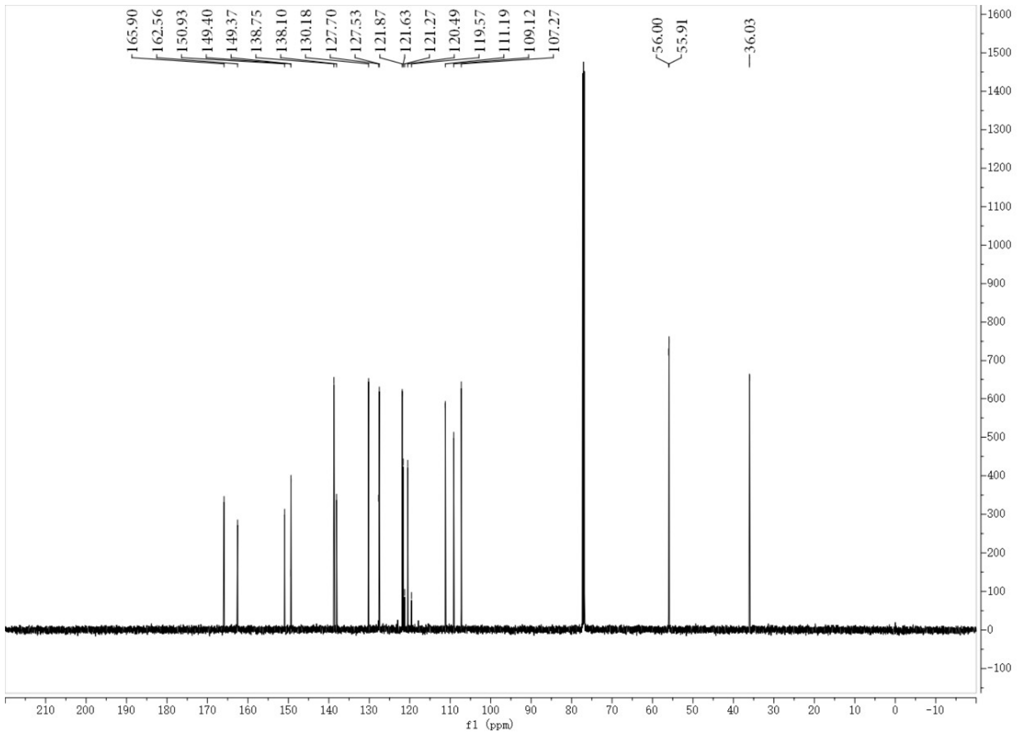

Fig. S71 The ^{13}C NMR spectrum of the target compound **6r**

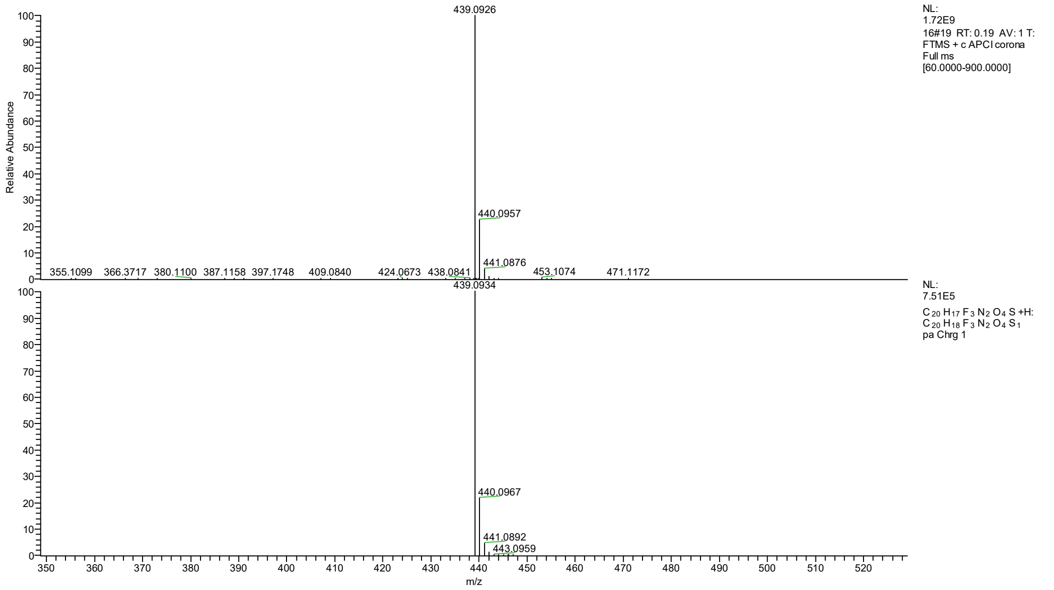

Fig. S72 The HRMS spectrum of the target compound **6r**

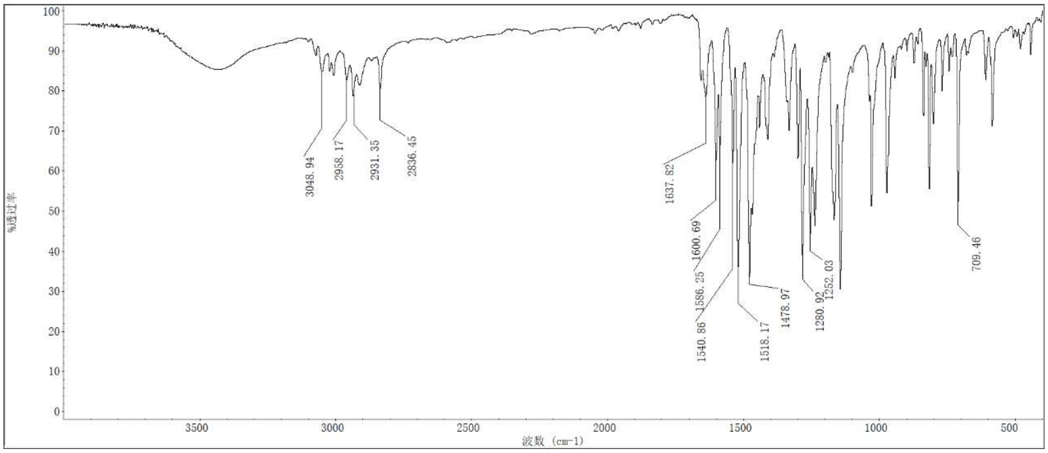

Fig. S73 The FT-IR spectrum of the target compound **6s**

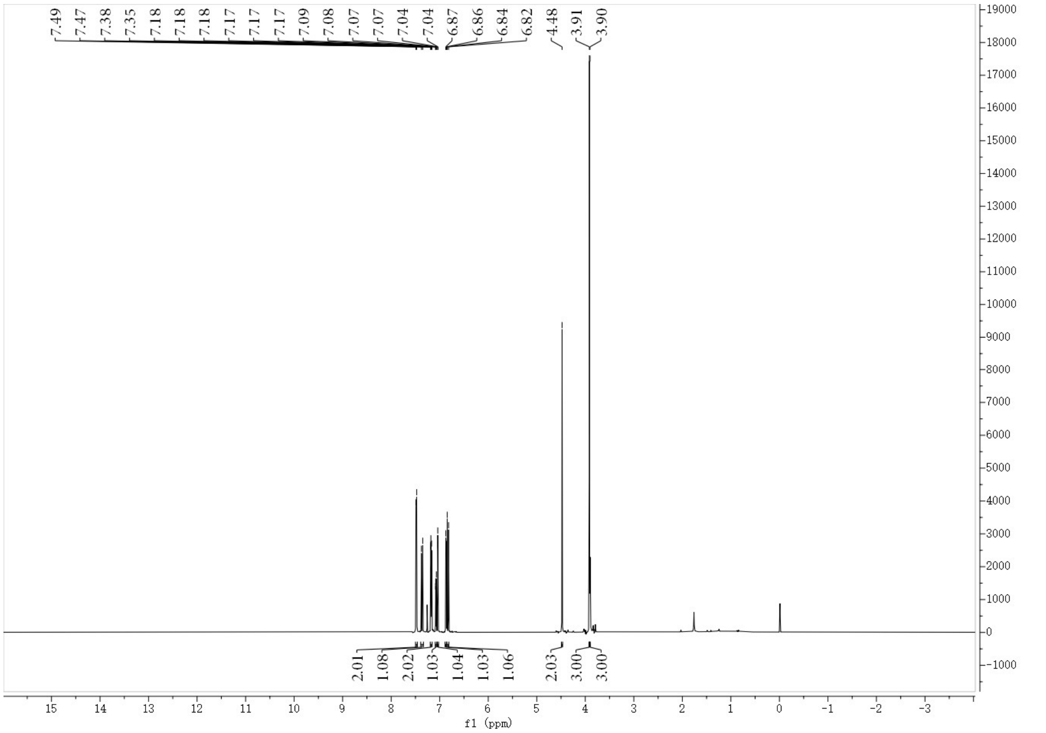

Fig. S74 The ^{1}H NMR spectrum of the target compound **6s**

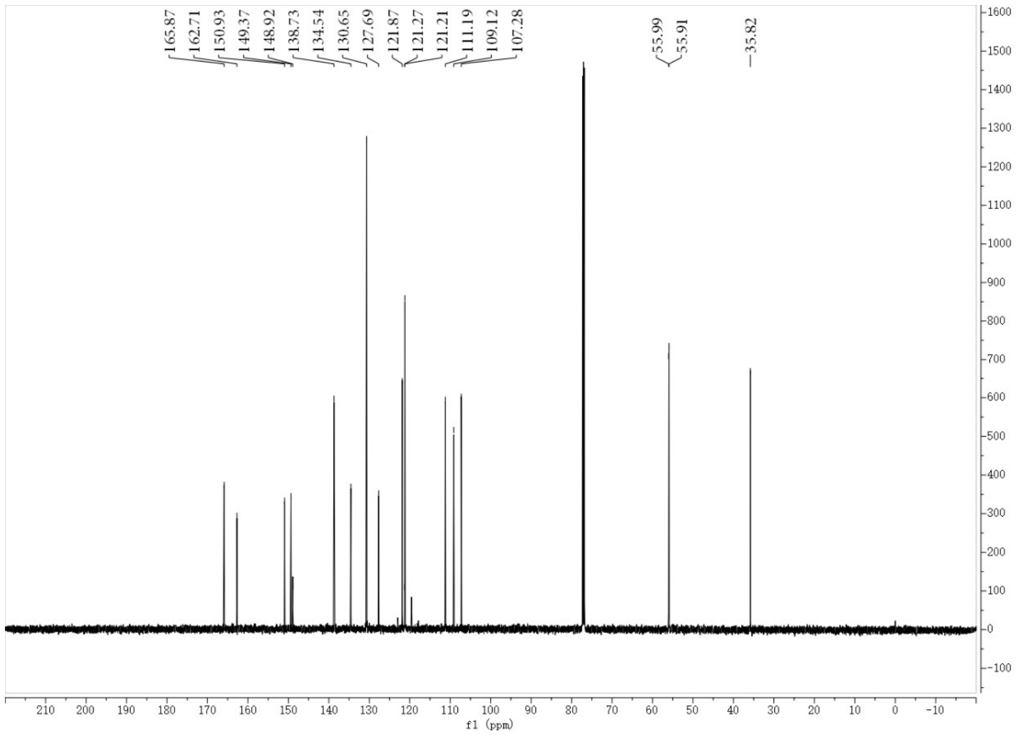

Fig. S75 The ^{13}C NMR spectrum of the target compound **6s**

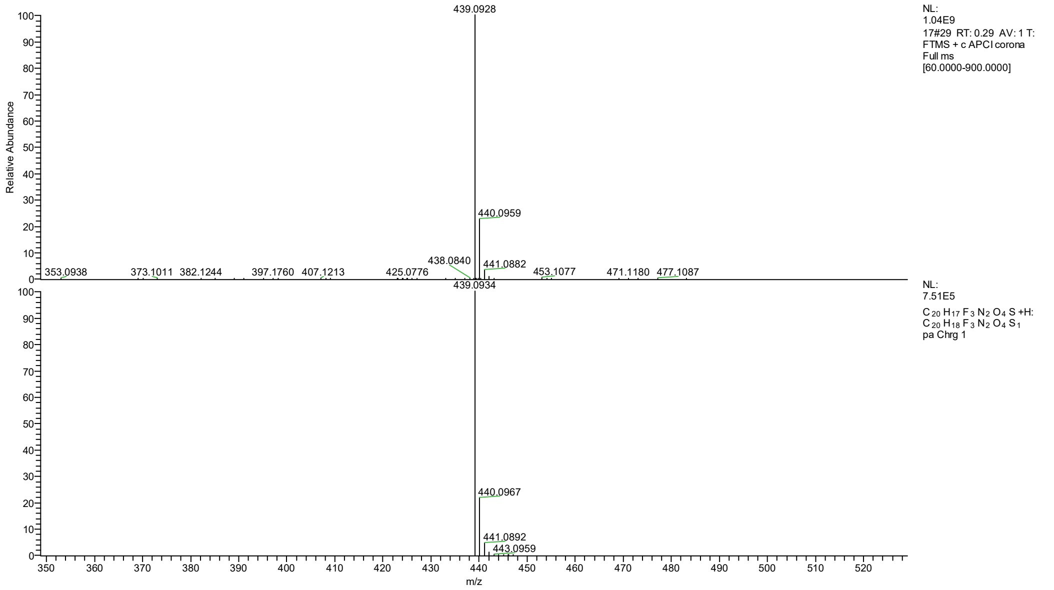

Fig. S76 The HRMS spectrum of the target compound **6s**

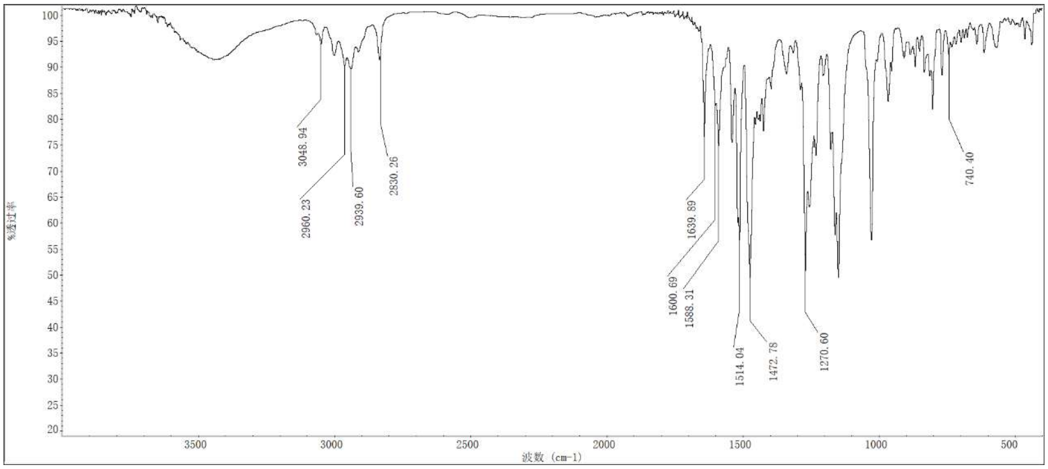

Fig. S77 The FT-IR spectrum of the target compound **6t**

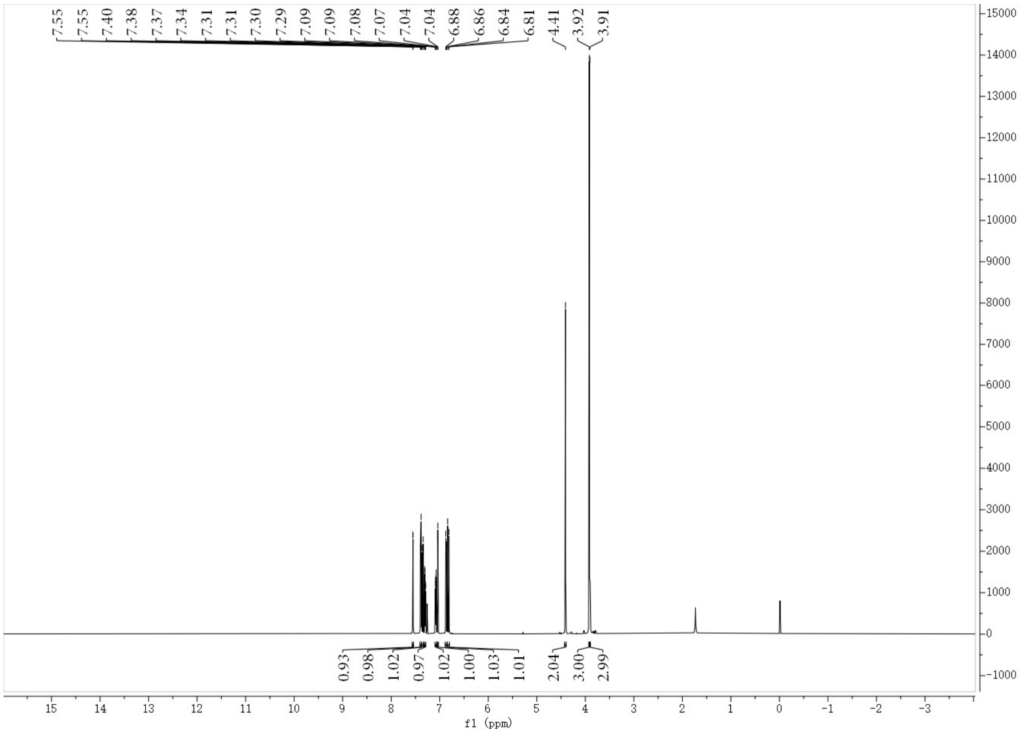

Fig. S78 The ^{1}H NMR spectrum of the target compound **6t**

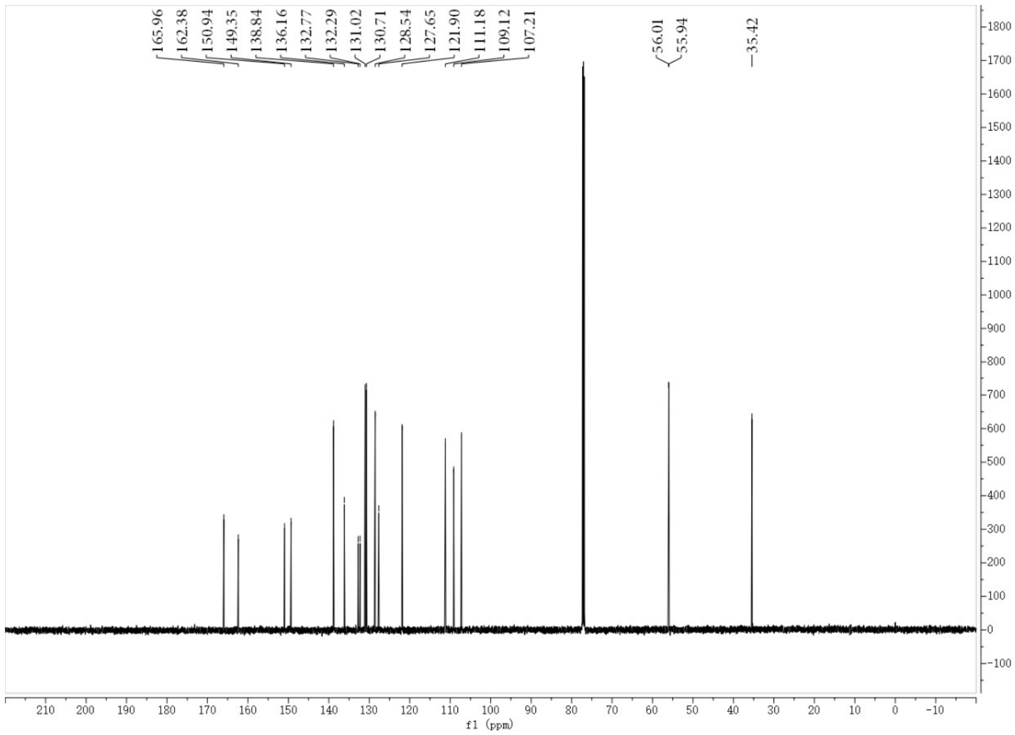

Fig. S79 The ^{13}C NMR spectrum of the target compound **6t**

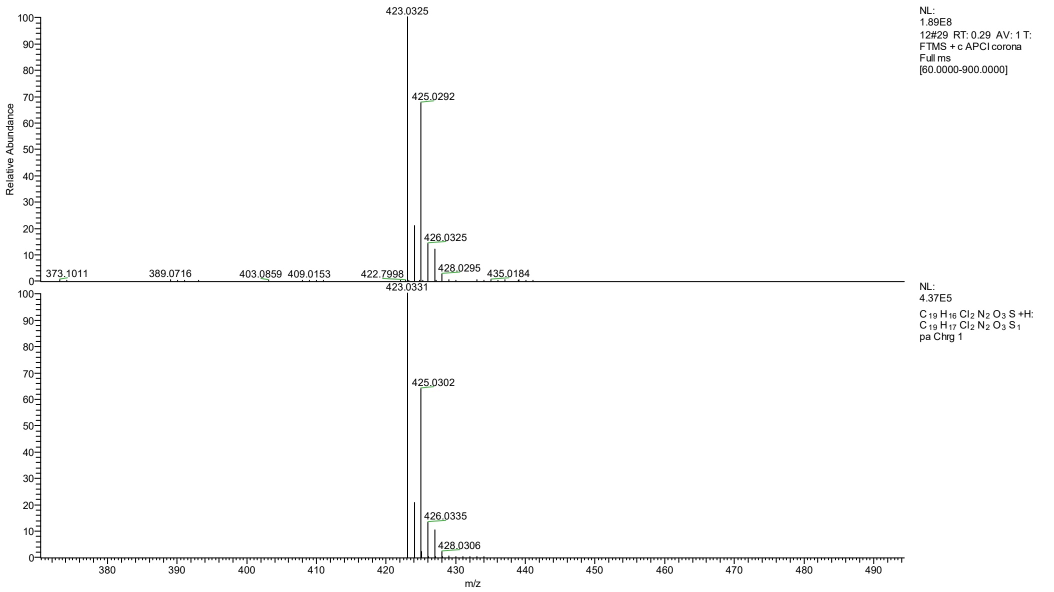

Fig. S80 The HRMS spectrum of the target compound **6t**

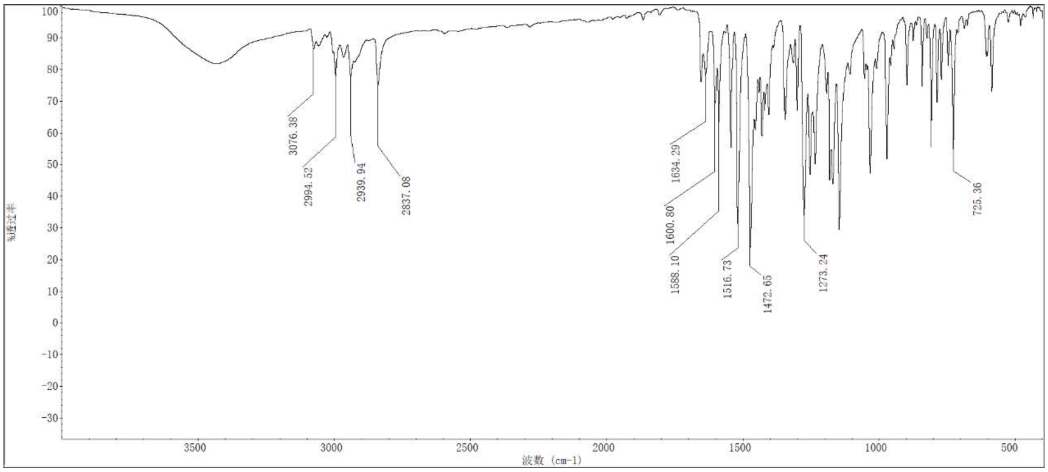

Fig. S81 The FT-IR spectrum of the target compound **6u**

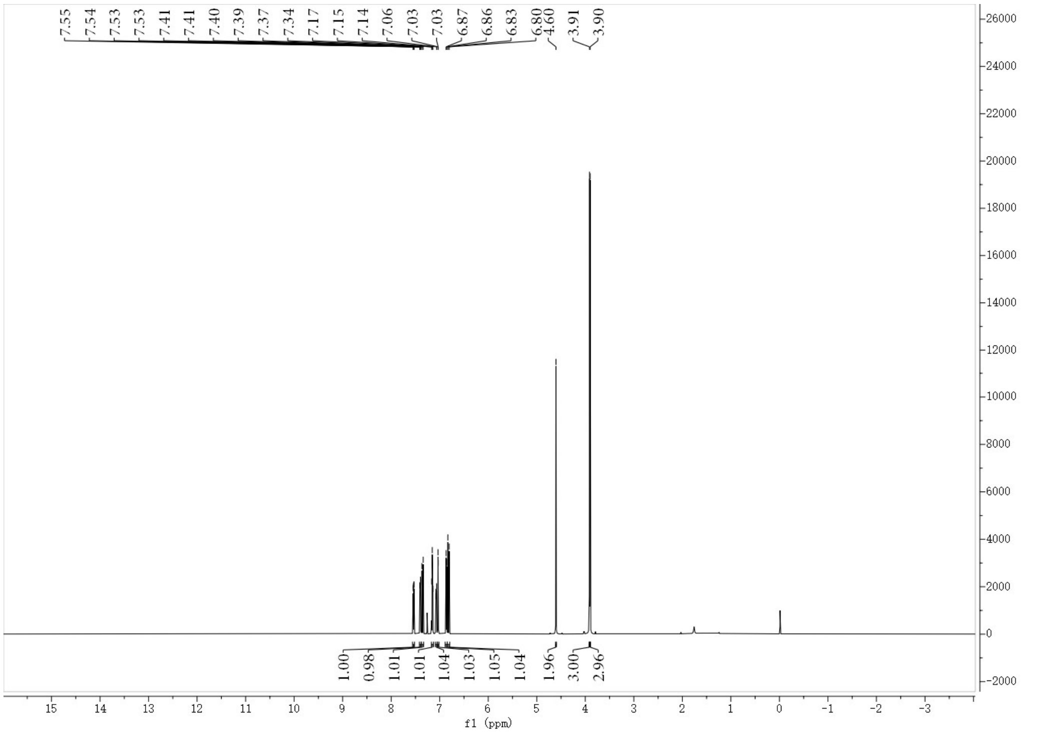

Fig. S82 The ^{1}H NMR spectrum of the target compound **6u**

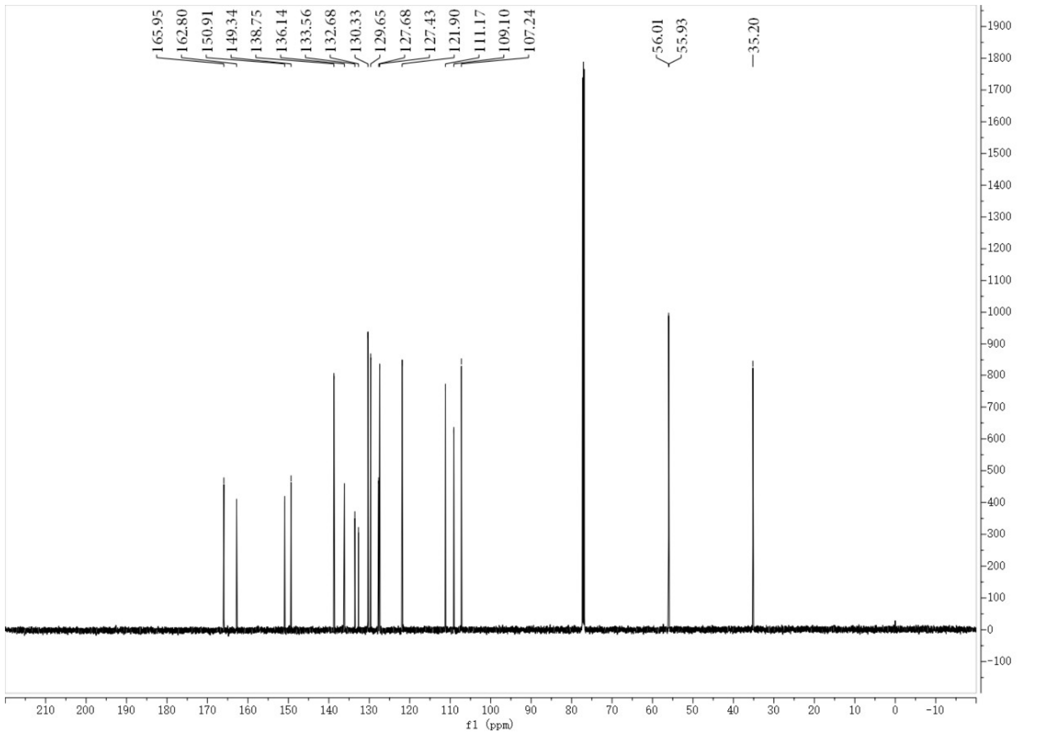

Fig. S83 The ^{13}C NMR spectrum of the target compound **6u**

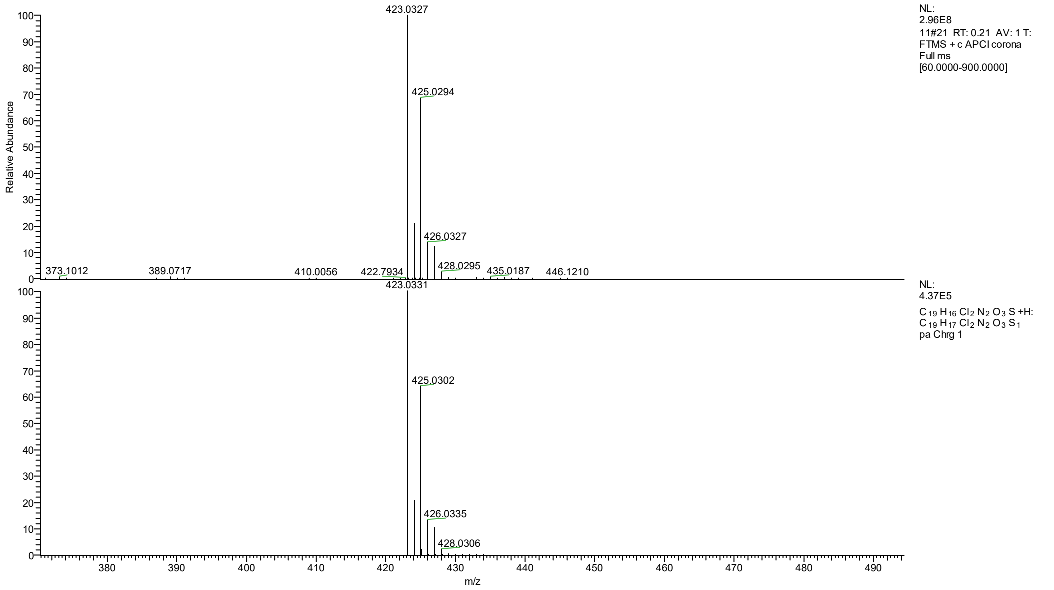

Fig. S84 The HRMS spectrum of the target compound **6u**

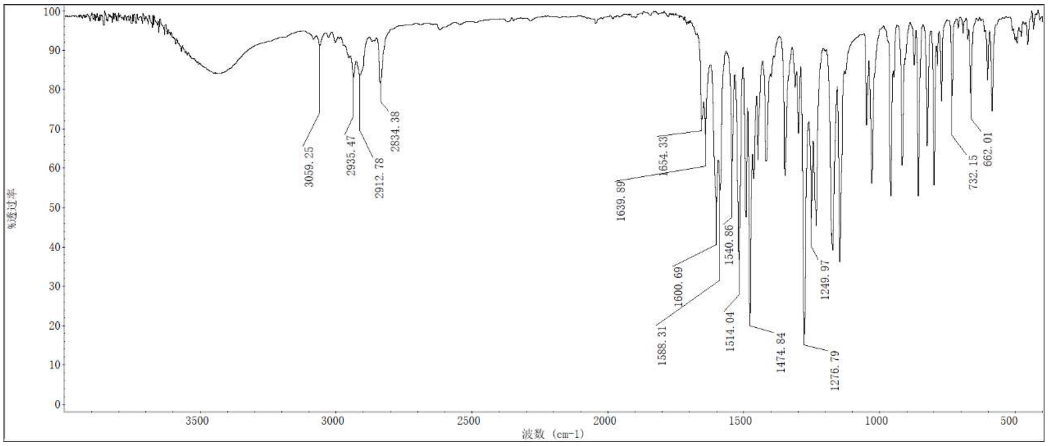

Fig. S85 The FT-IR spectrum of the target compound **6v**

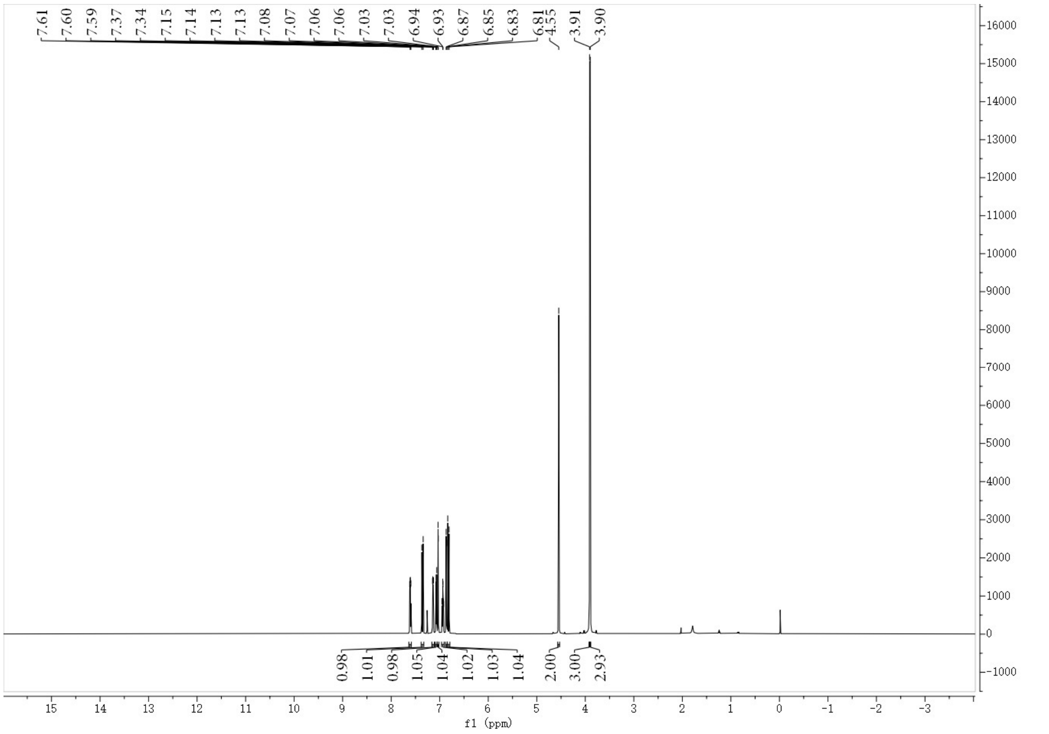

Fig. S86 The ^{1}H NMR spectrum of the target compound **6v**

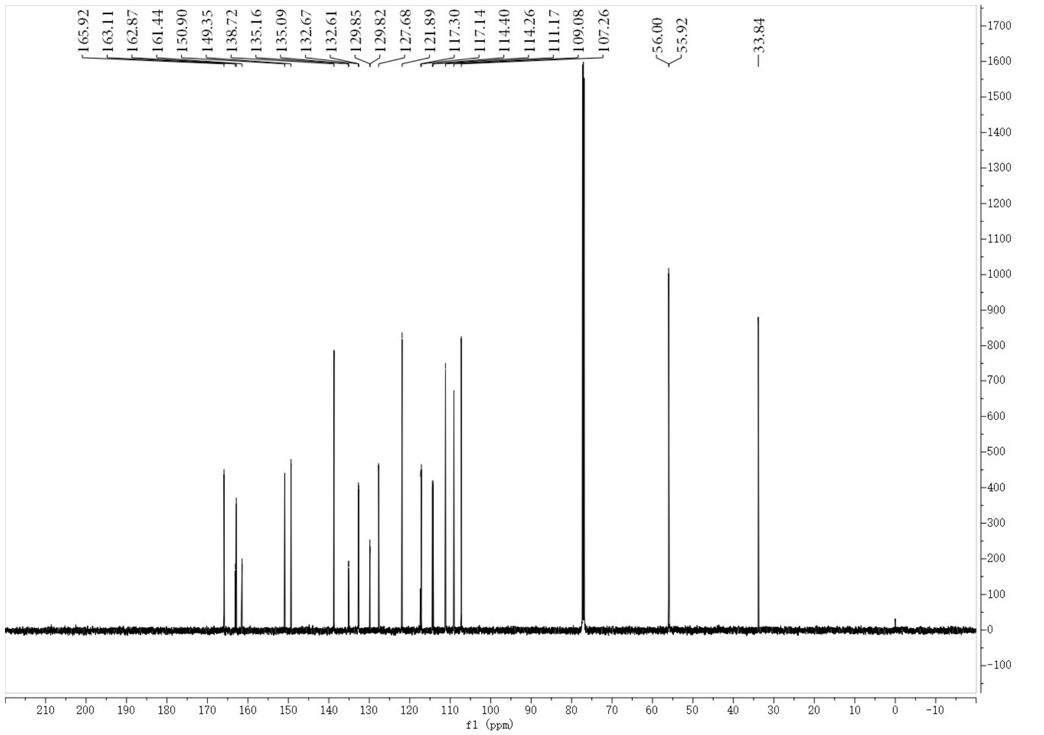

Fig. S87 The ^{13}C NMR spectrum of the target compound **6v**

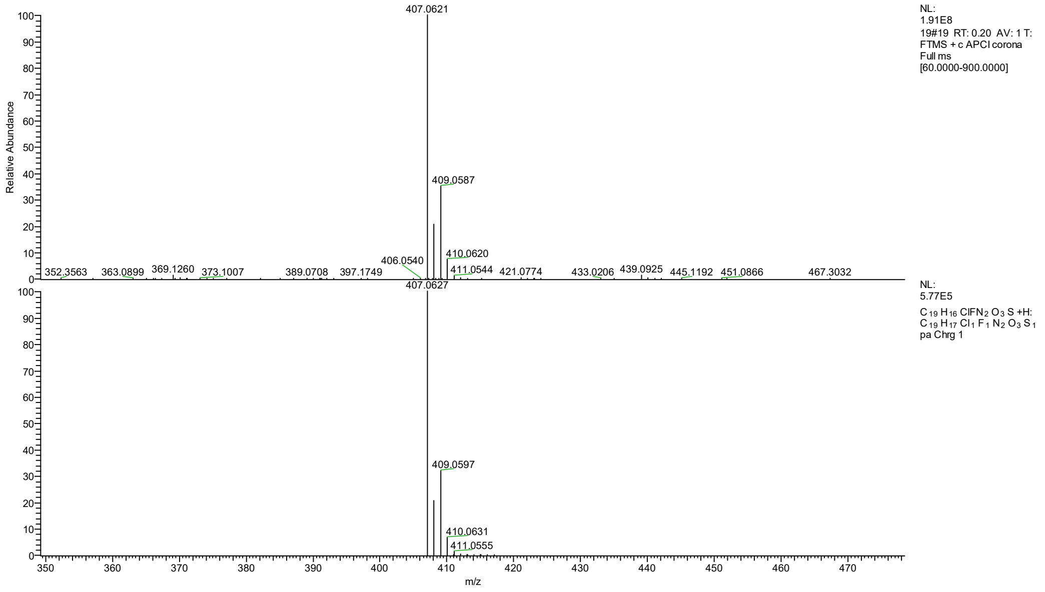

Fig. S88 The HRMS spectrum of the target compound **6v**

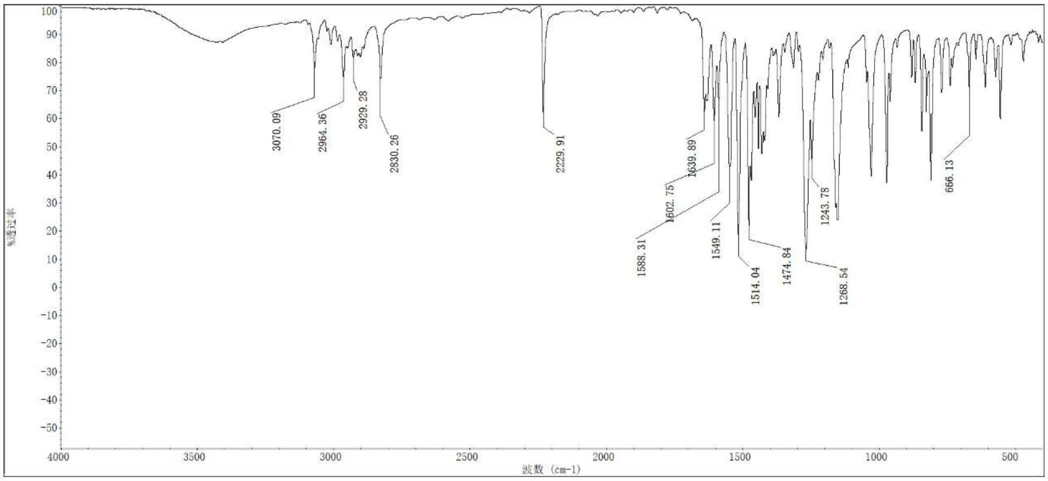

Fig. S89 The FT-IR spectrum of the target compound **6w**

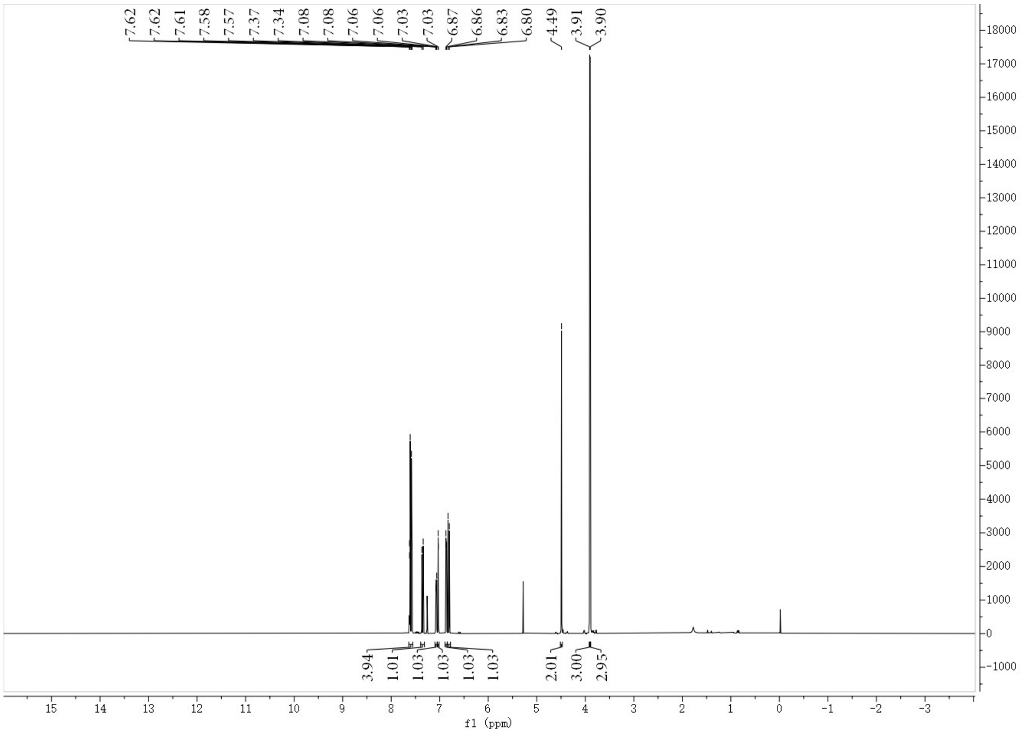

Fig. S90 The ^{1}H NMR spectrum of the target compound **6w**

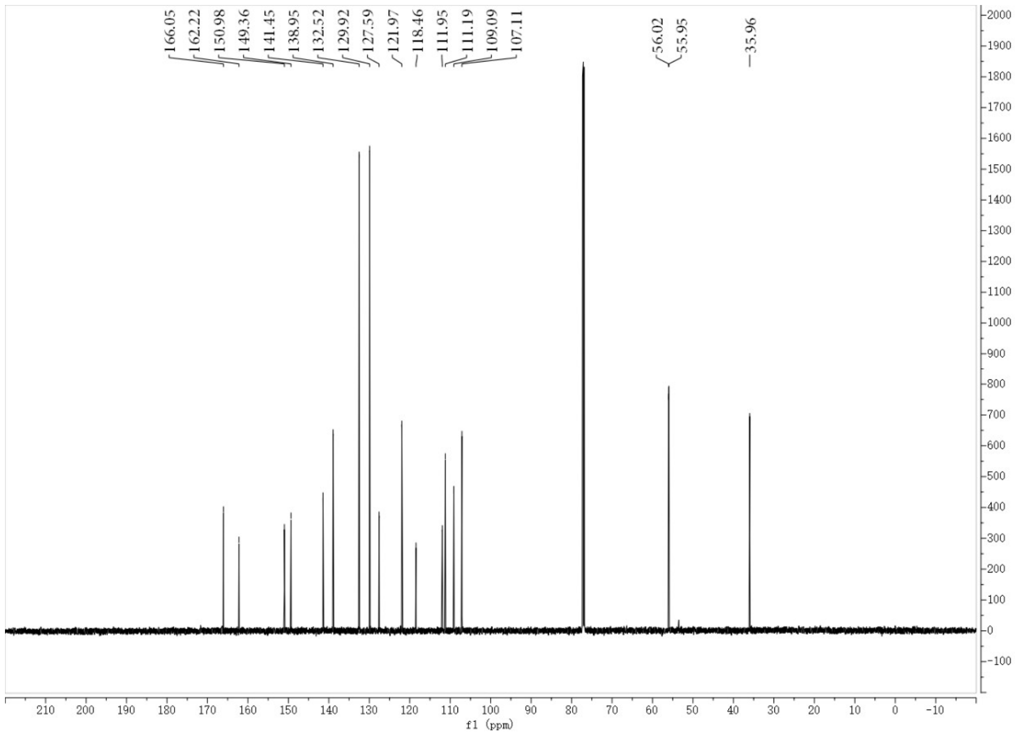

Fig. S91 The ^{13}C NMR spectrum of the target compound **6w**

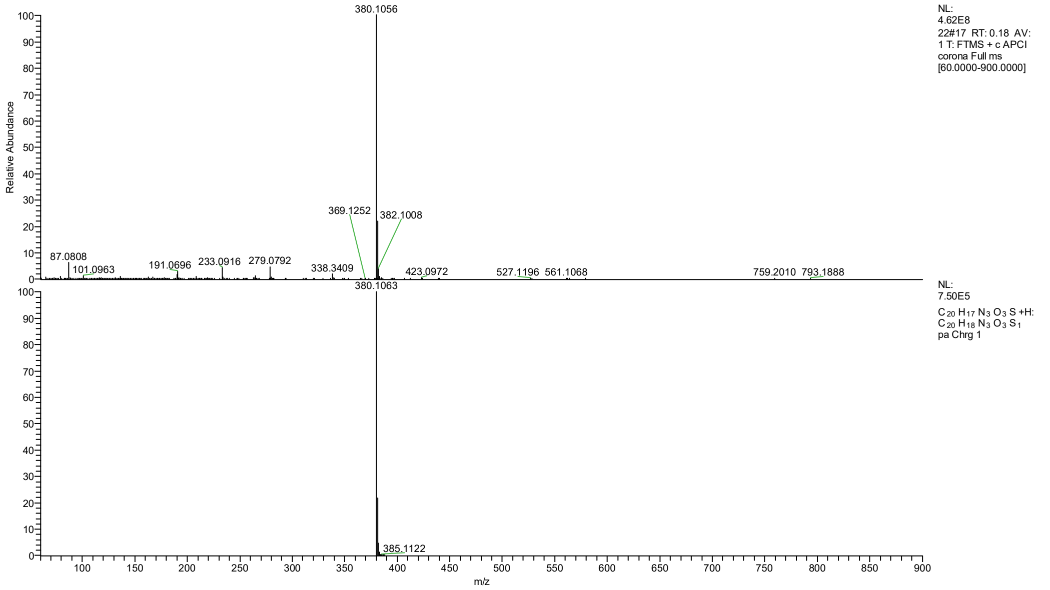

Fig. S92 The HRMS spectrum of the target compound **6w**

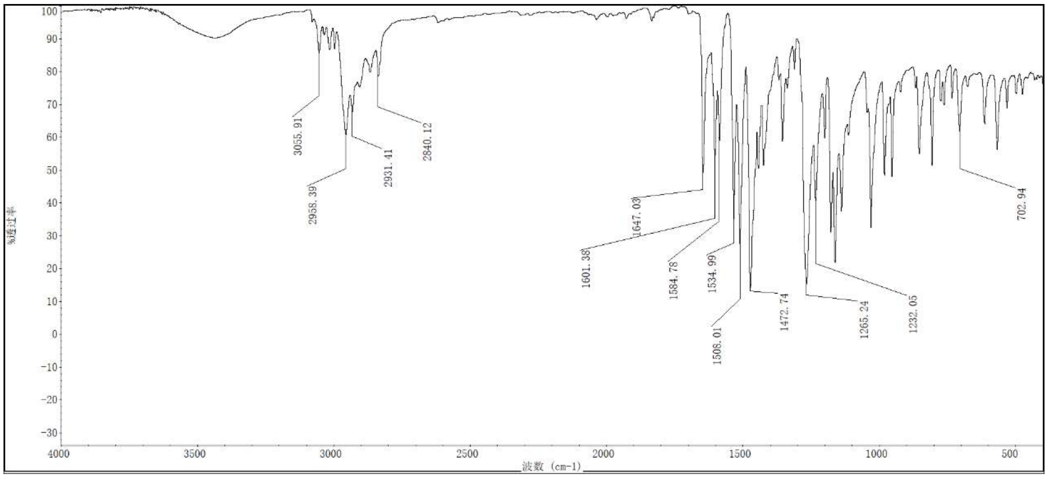

Fig. S93 The FT-IR spectrum of the target compound **6x**

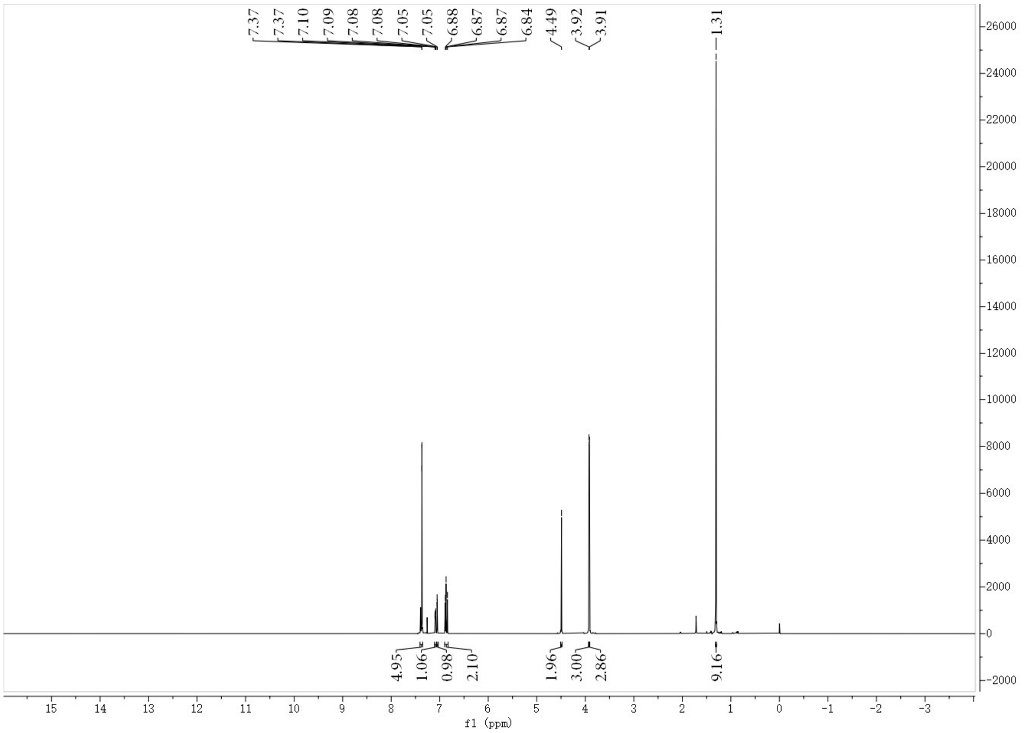

Fig. S94 The ^{1}H NMR spectrum of the target compound **6x**

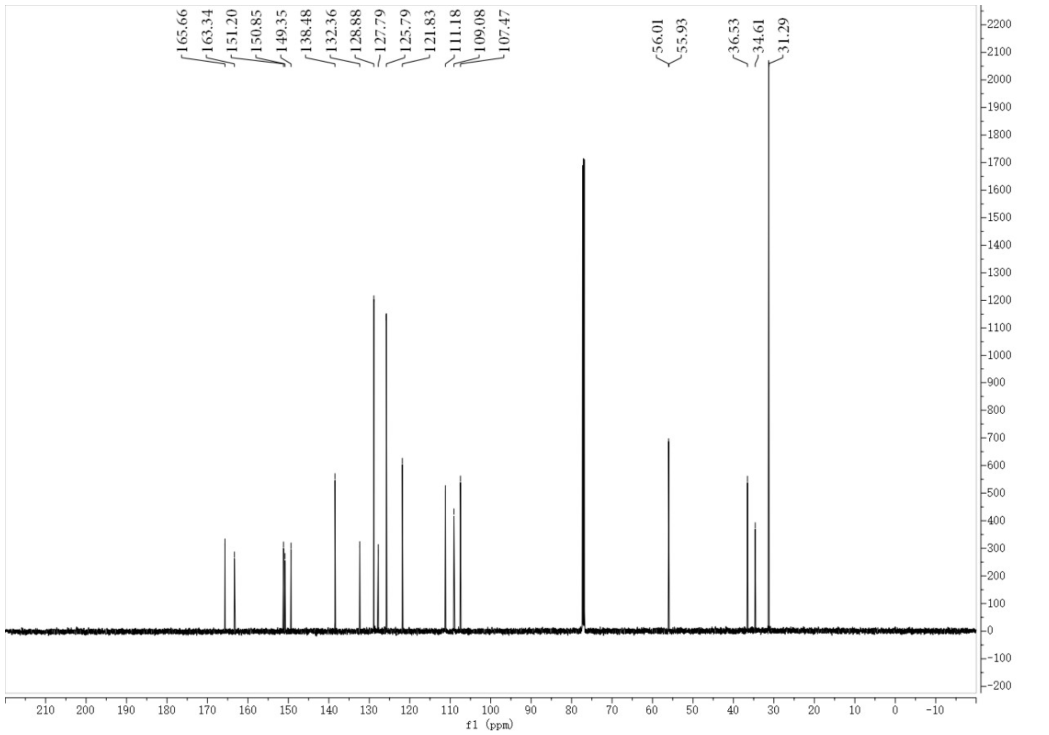

Fig. S95 The ^{13}C NMR spectrum of the target compound **6x**

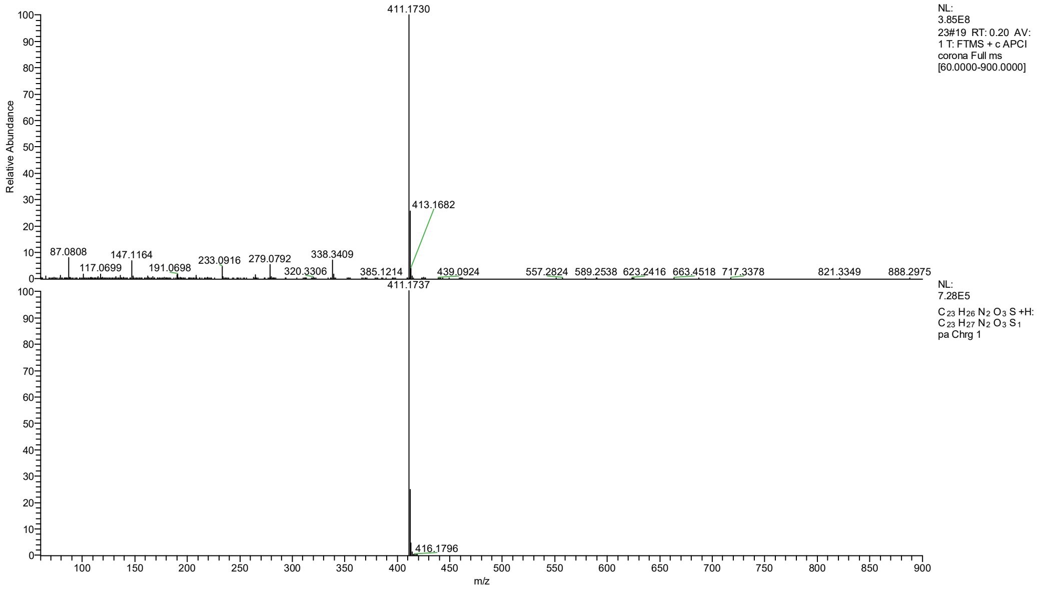

Fig. S96 The HRMS spectrum of the target compound **6x**

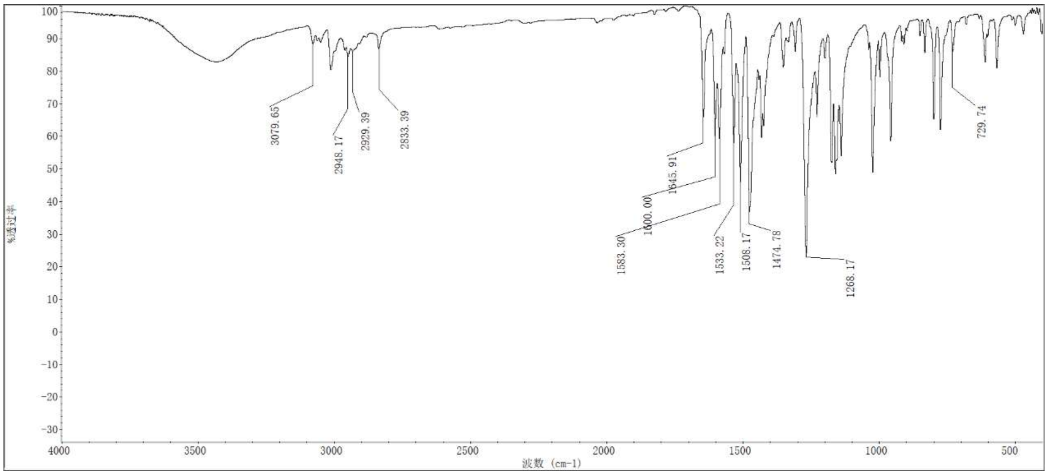

Fig. S97 The FT-IR spectrum of the target compound **6y**

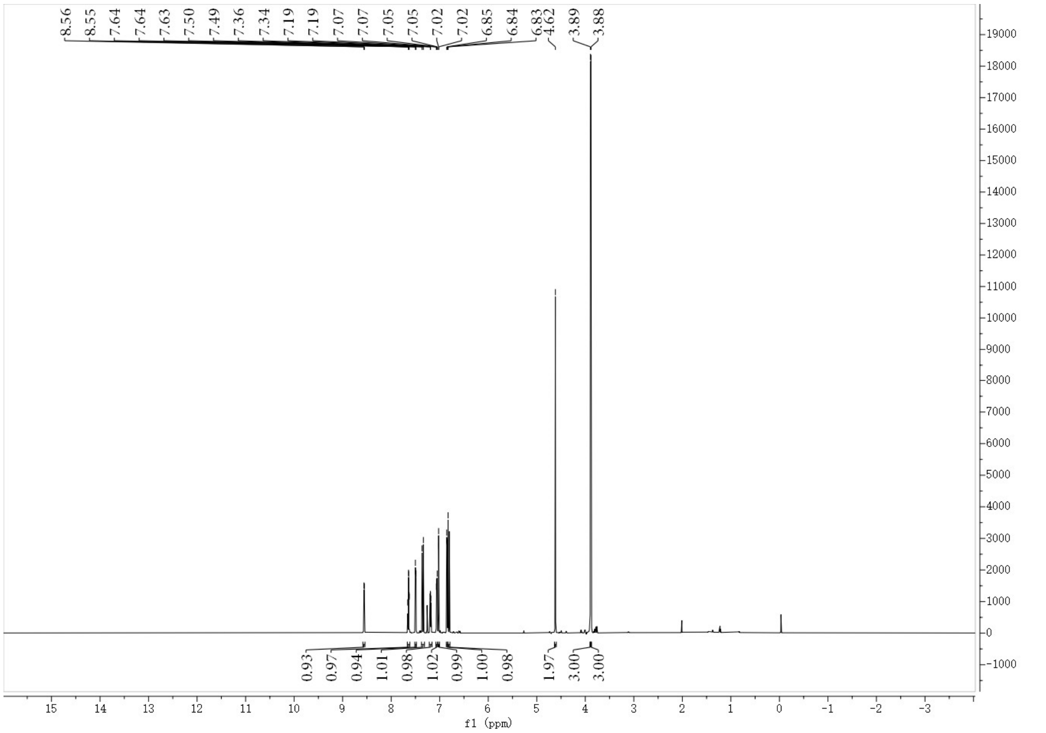

Fig. S98 The ^{1}H NMR spectrum of the target compound **6y**

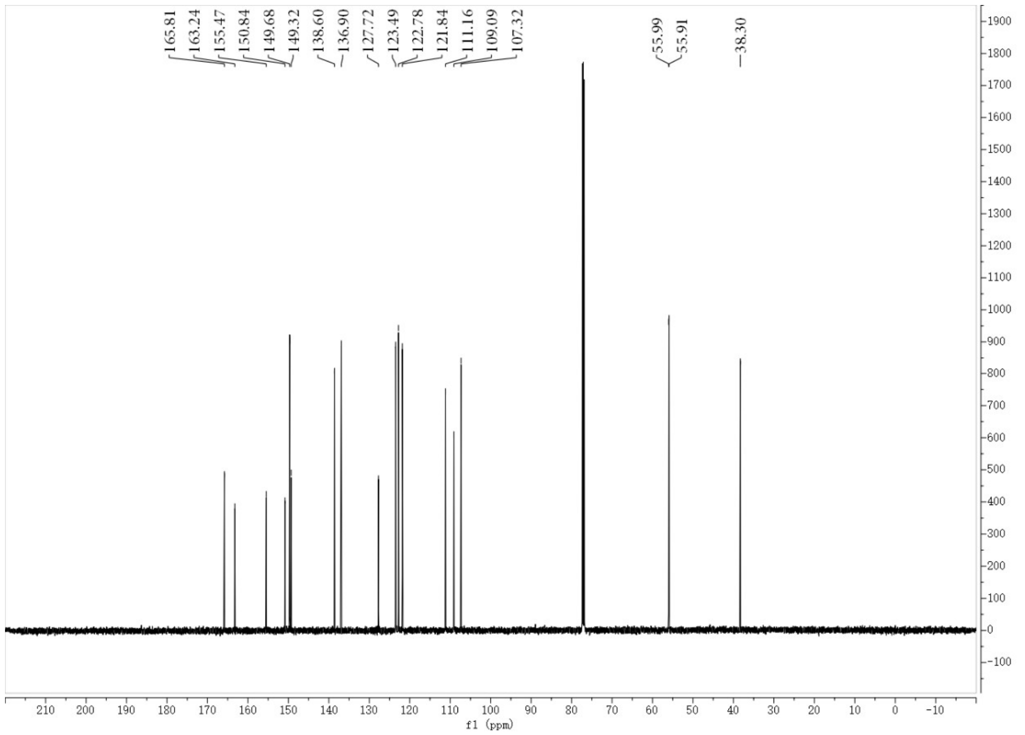

Fig. S99 The ^{13}C NMR spectrum of the target compound **6y**

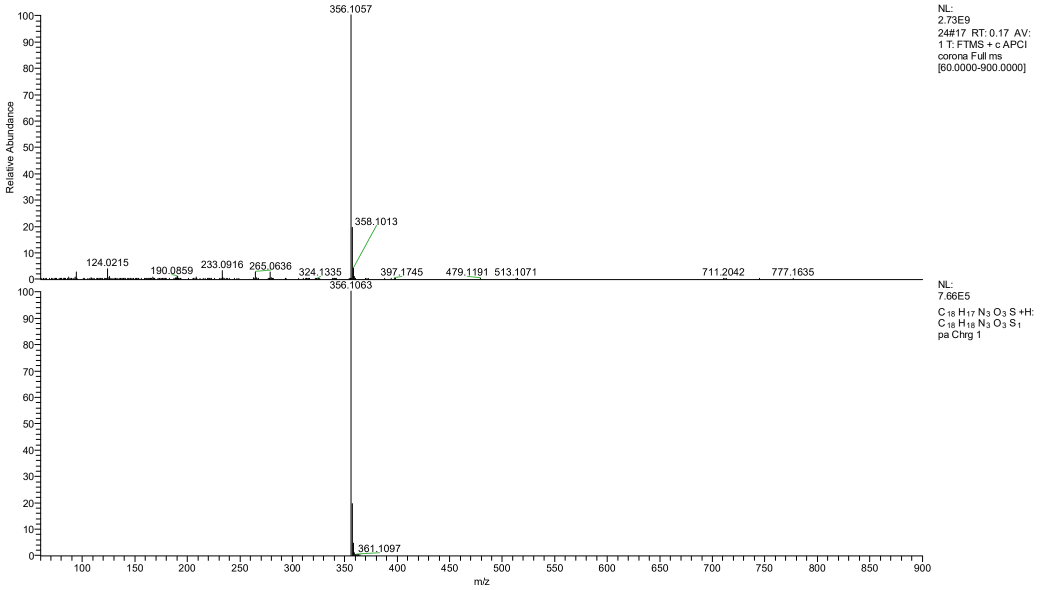

Fig. S100 The HRMS spectrum of the target compound **6y**

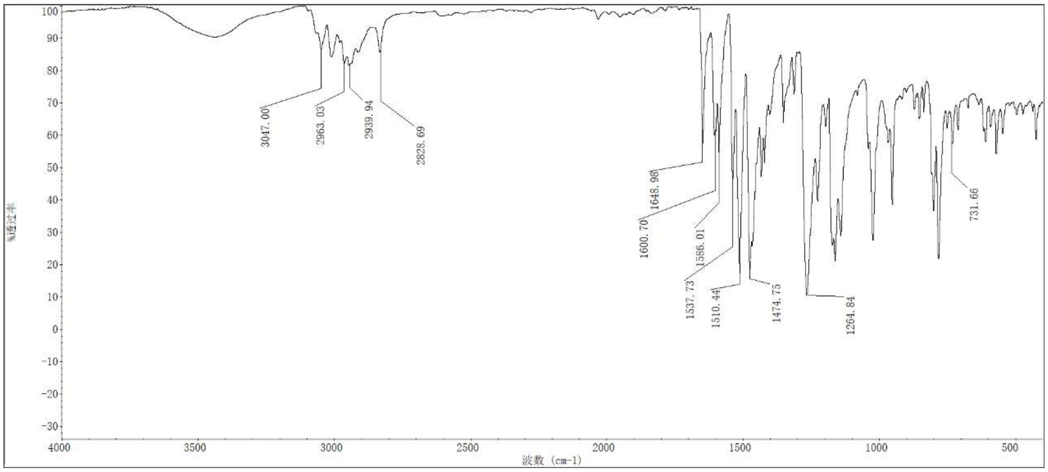

Fig. S101 The FT-IR spectrum of the target compound **6z**

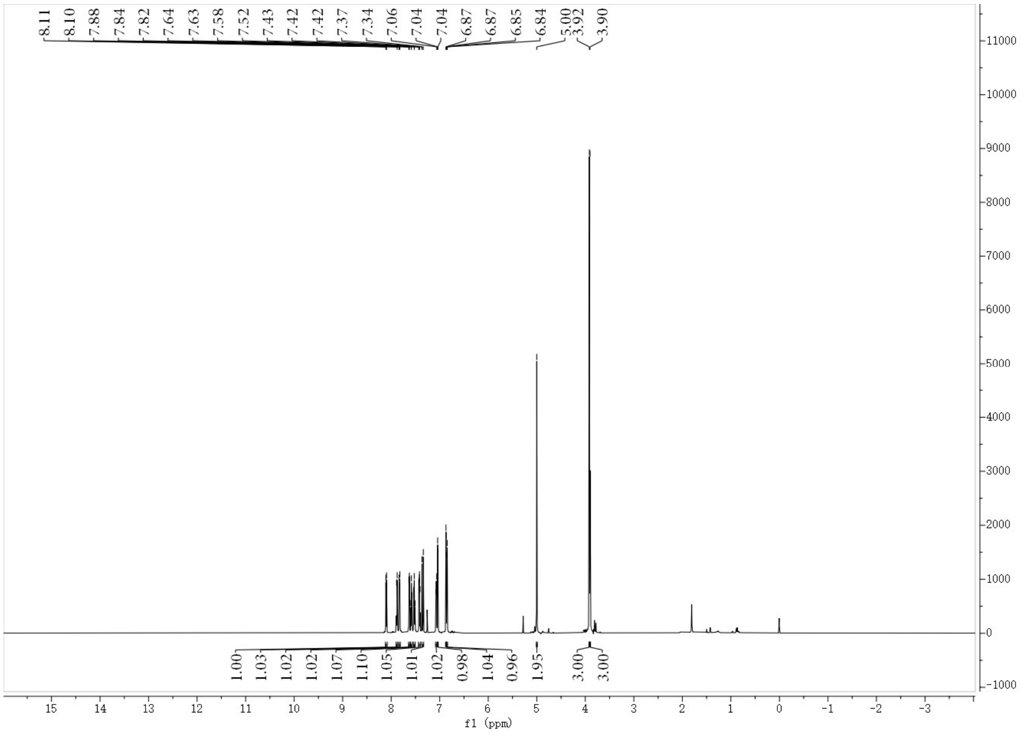

Fig. S102 The ^{1}H NMR spectrum of the target compound **6z**

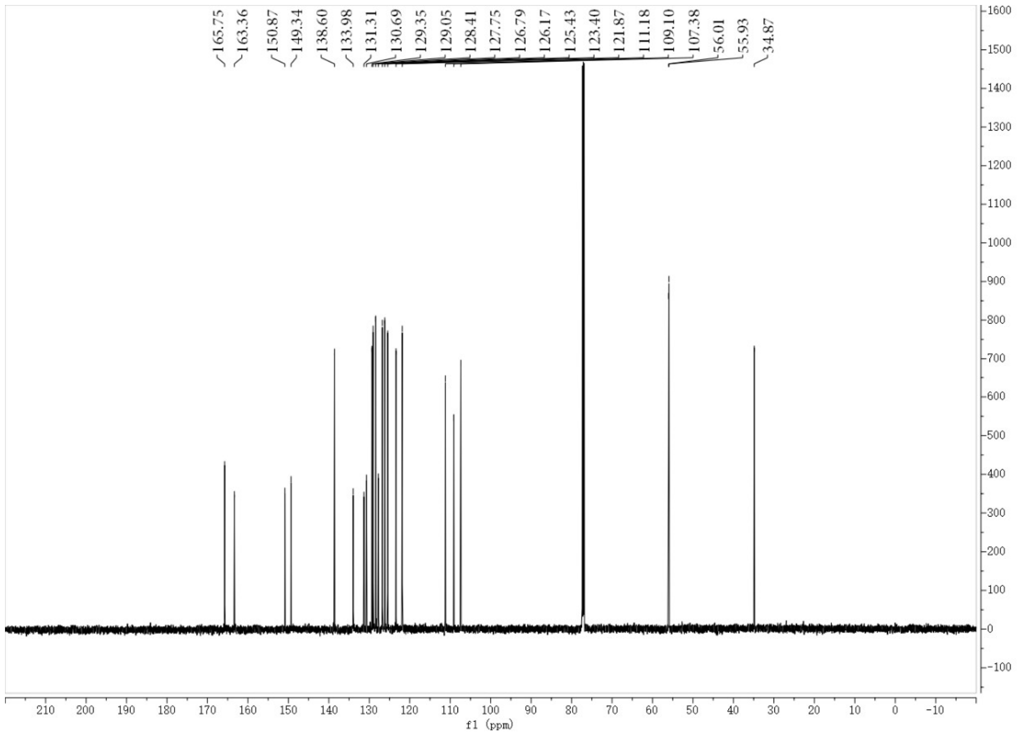

Fig. S103 The ^{13}C NMR spectrum of the target compound **6z**

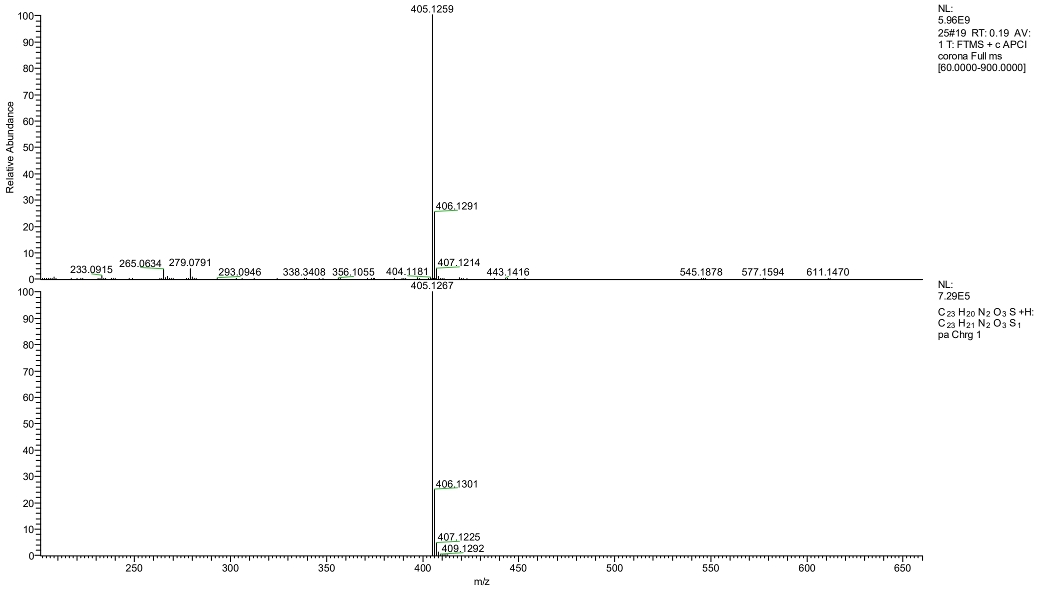

Fig. S104 The HRMS spectrum of the target compound **6z**

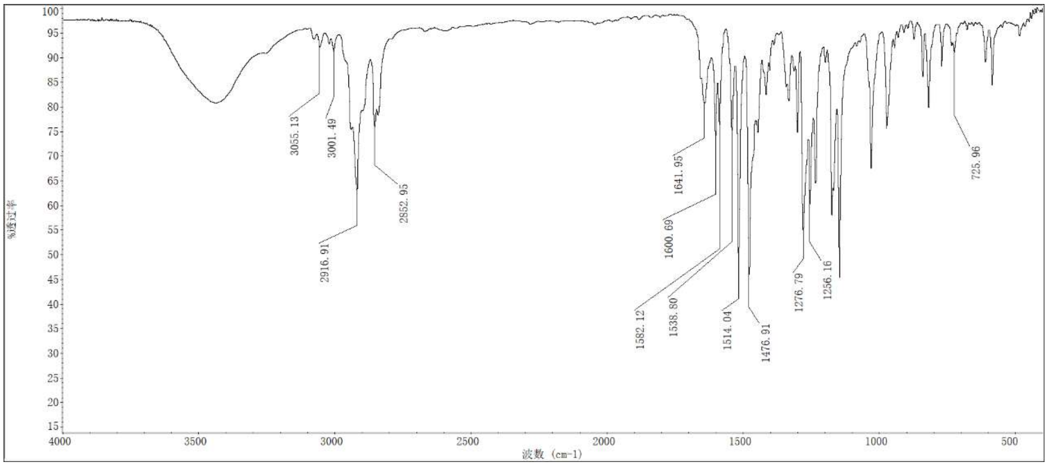

Fig. S105 The FT-IR spectrum of the target compound **6aa**

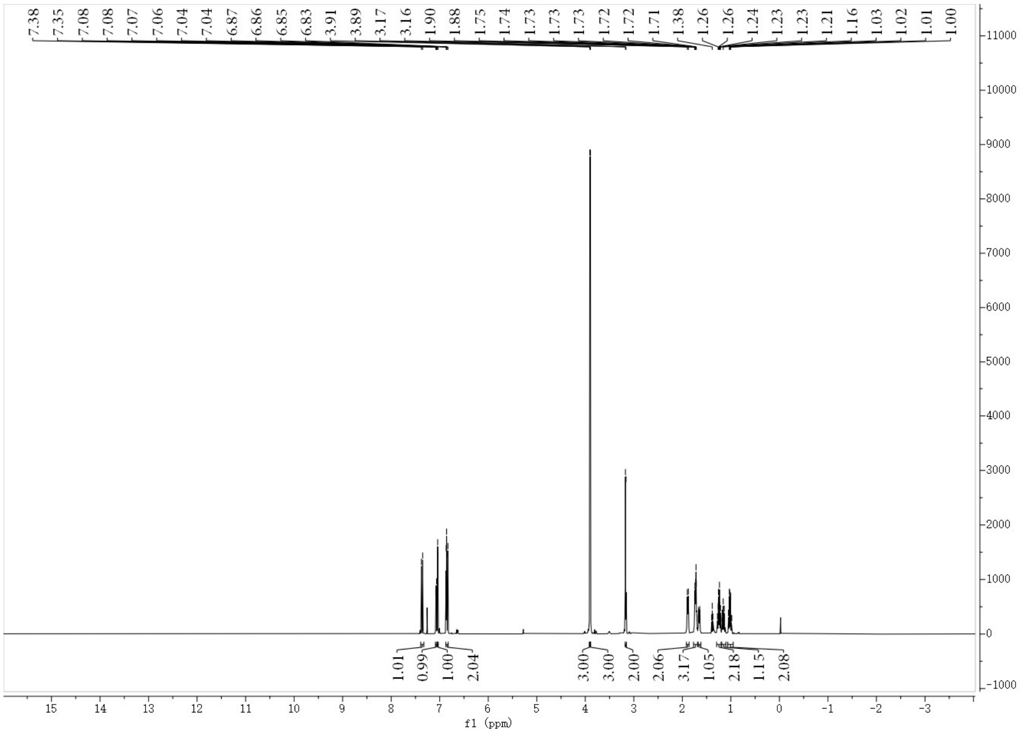

Fig. S106 The ^{1}H NMR spectrum of the target compound **6aa**

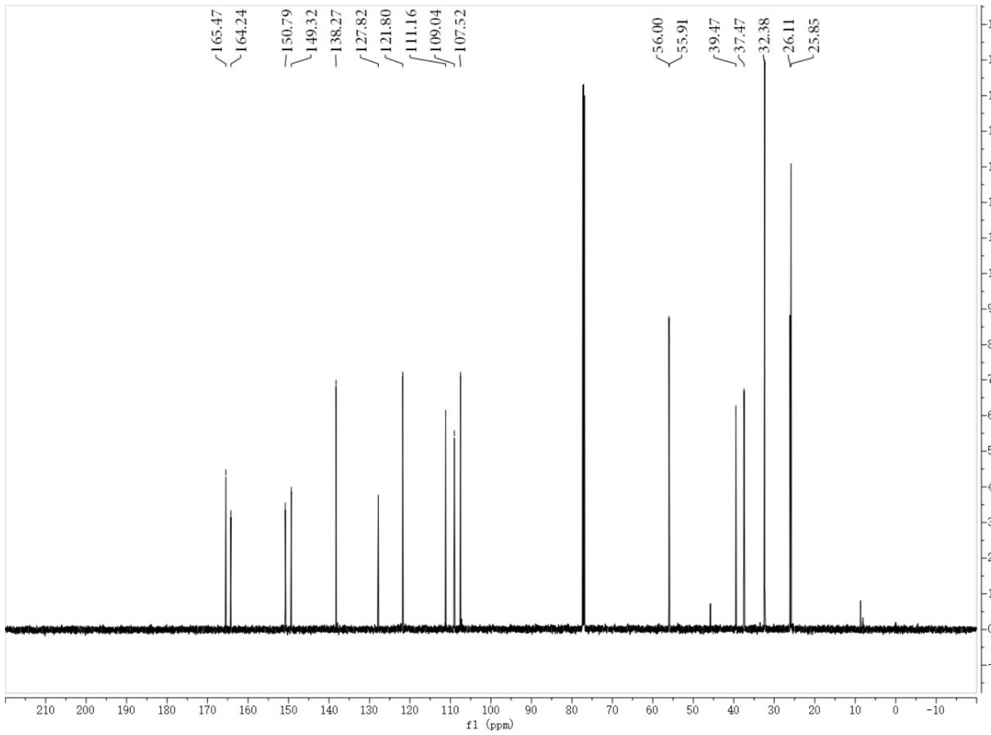

Fig. S107 The ^{13}C NMR spectrum of the target compound **6aa**

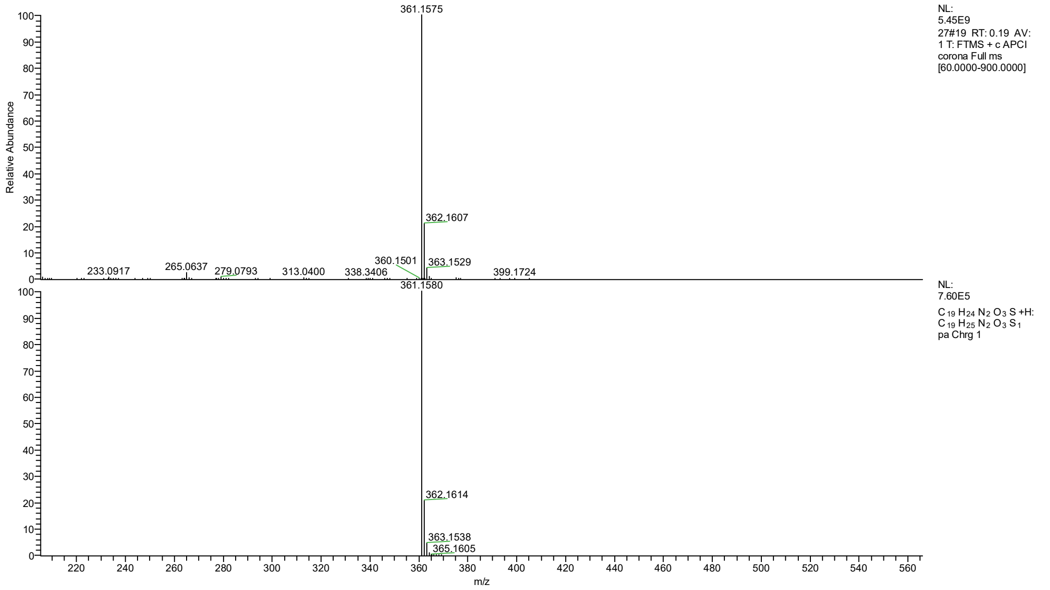

Fig. S108 The HRMS spectrum of the target compound **6aa**

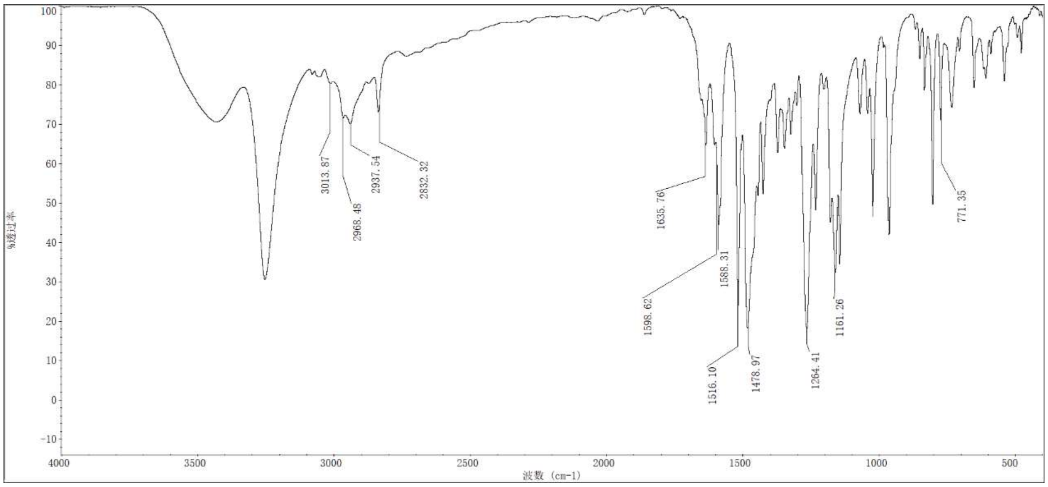

Fig. S109 The FT-IR spectrum of the target compound **6ab**

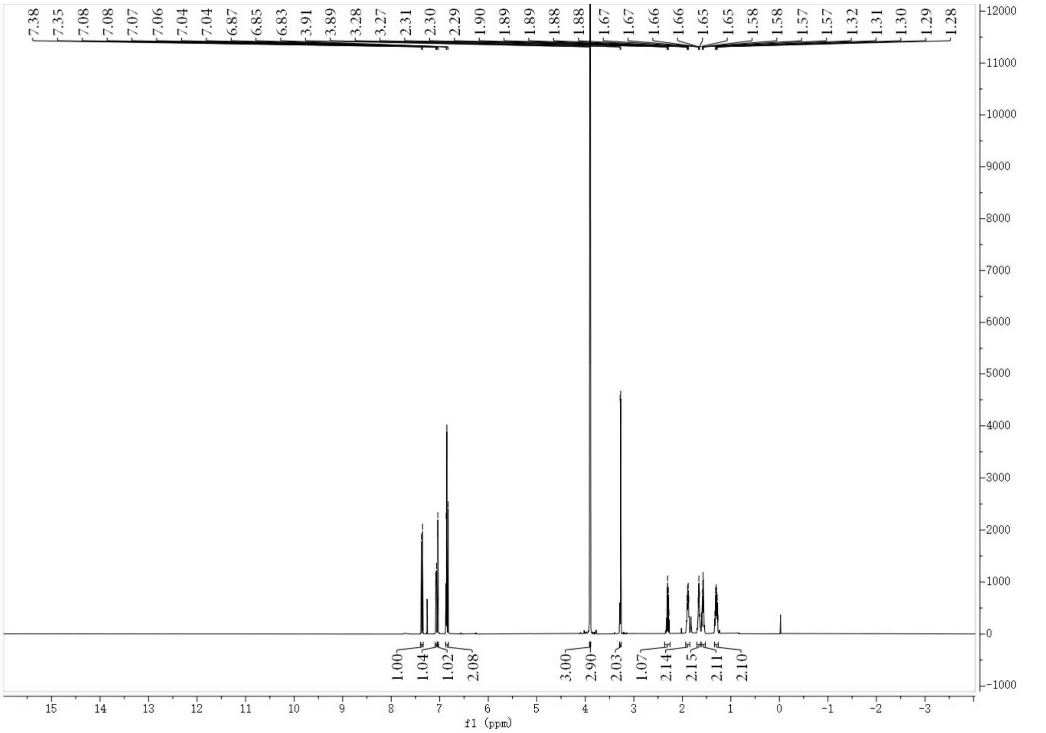

Fig. S110 The ^{1}H NMR spectrum of the target compound **6ab**

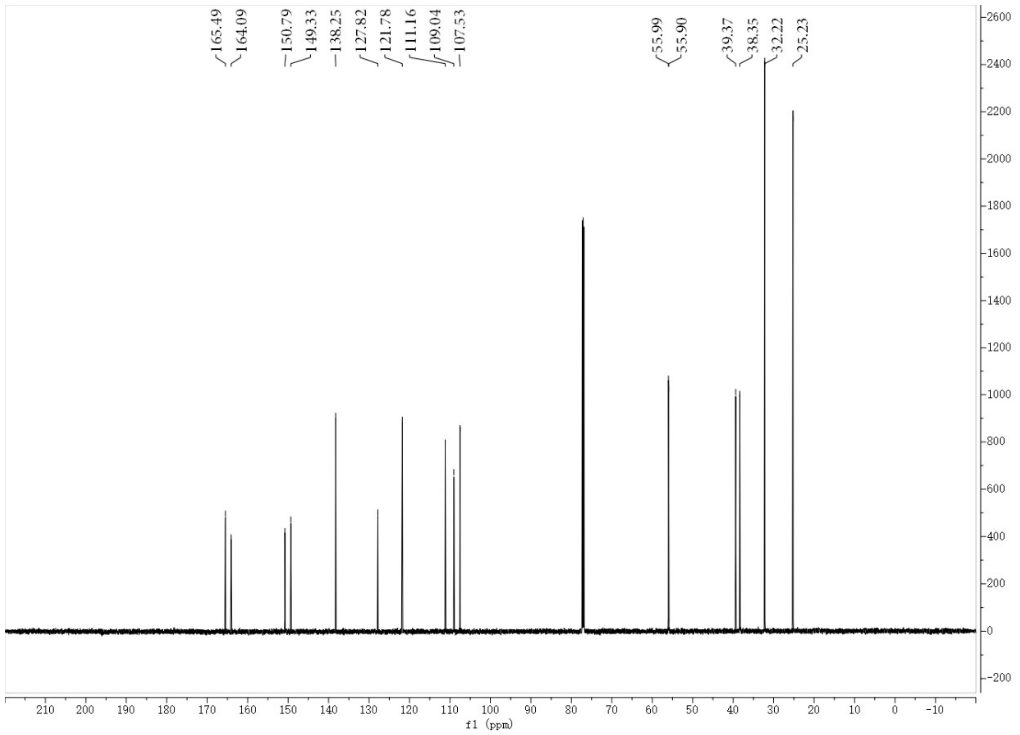

Fig. S111 The ^{13}C NMR spectrum of the target compound **6ab**

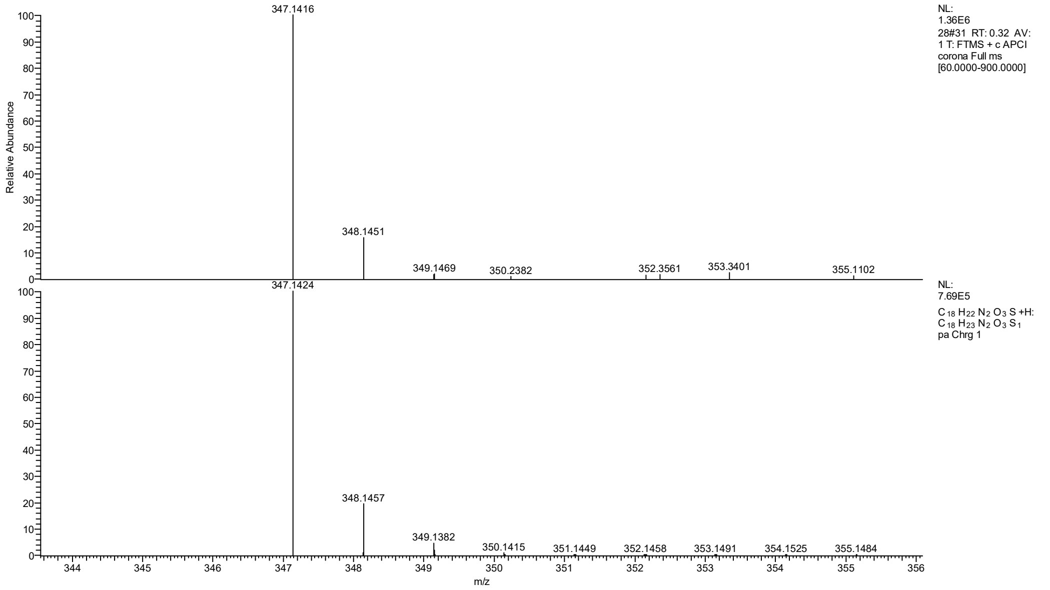

Fig. S112 The HRMS spectrum of the target compound **6ab**

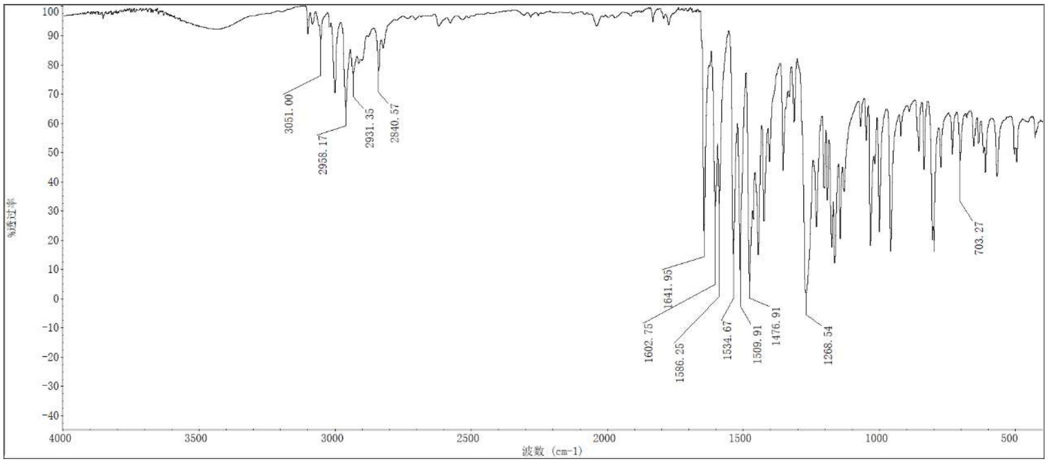

Fig. S113 The FT-IR spectrum of the target compound **6ac**

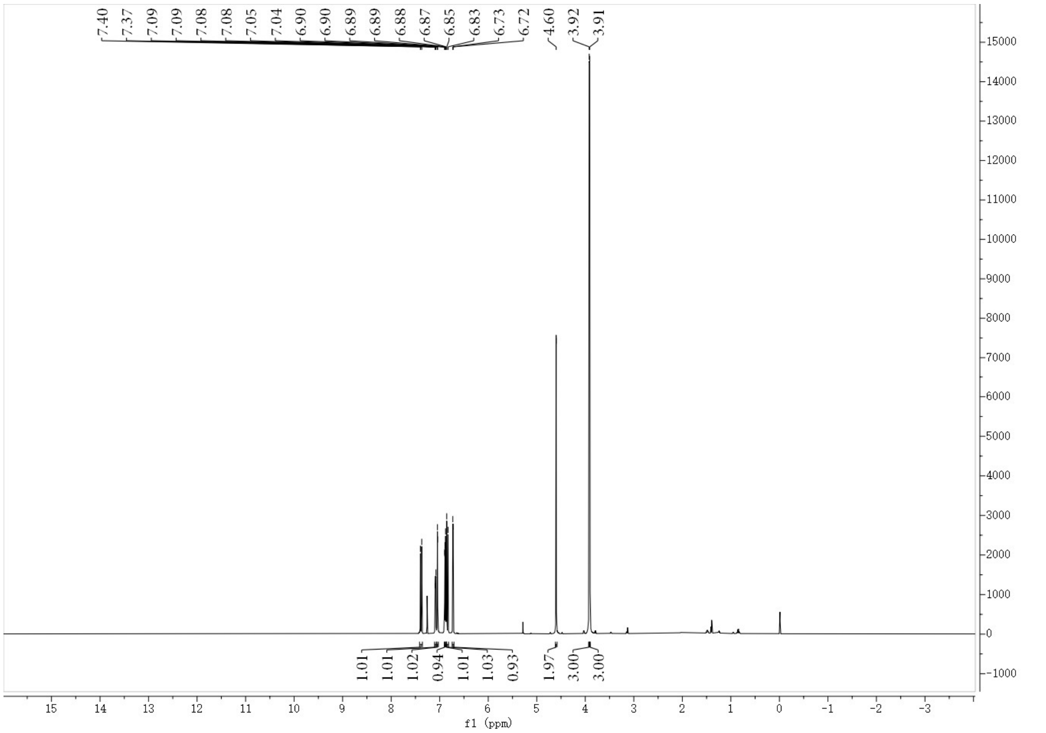

Fig. S114 The ^{1}H NMR spectrum of the target compound **6ac**

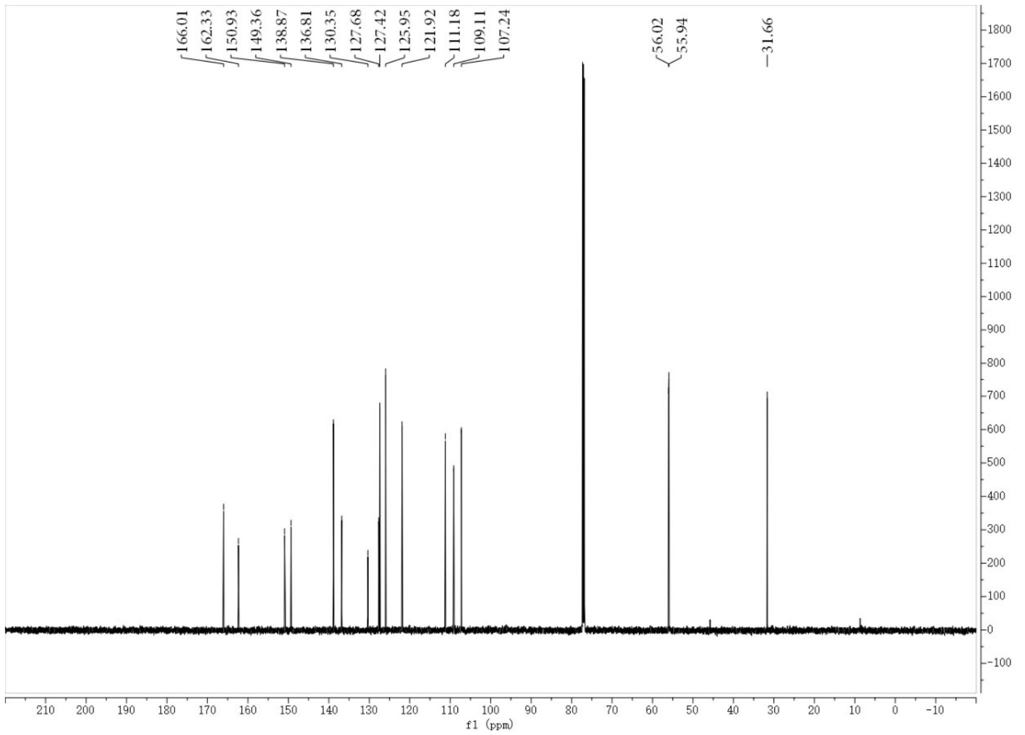

Fig. S115 The ^{13}C NMR spectrum of the target compound **6ac**

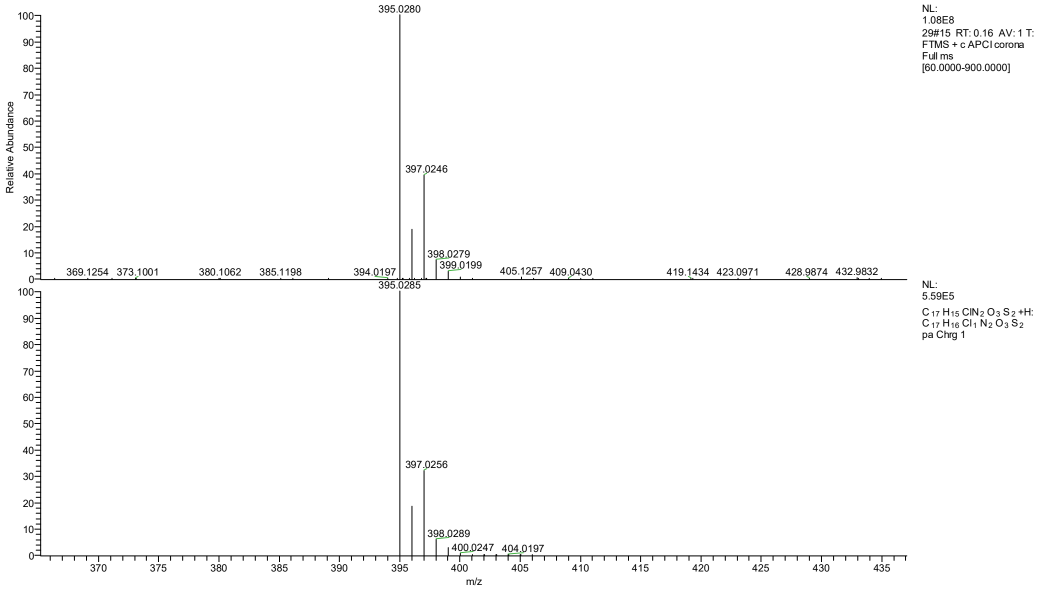

Fig. S116 The HRMS spectrum of the target compound **6af**

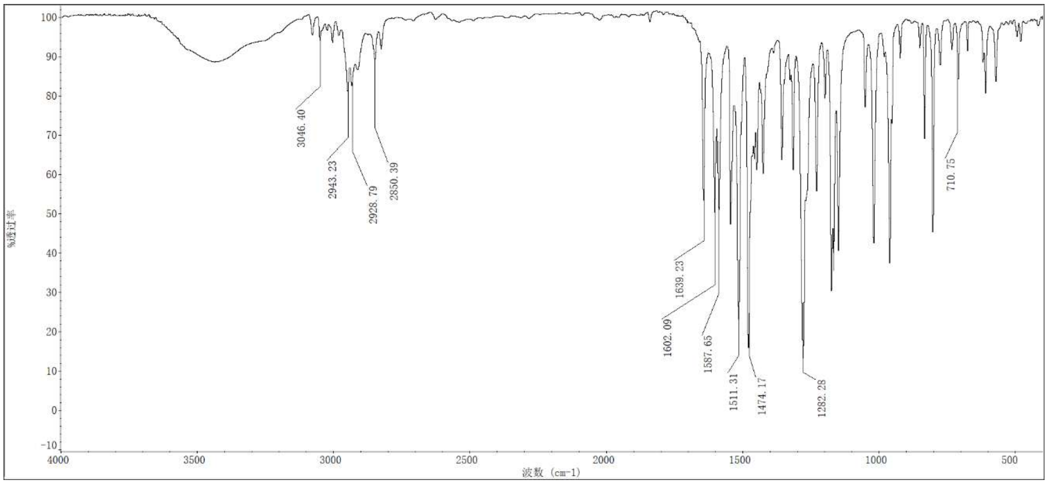

Fig. S117 The FT-IR spectrum of the target compound **6ad**

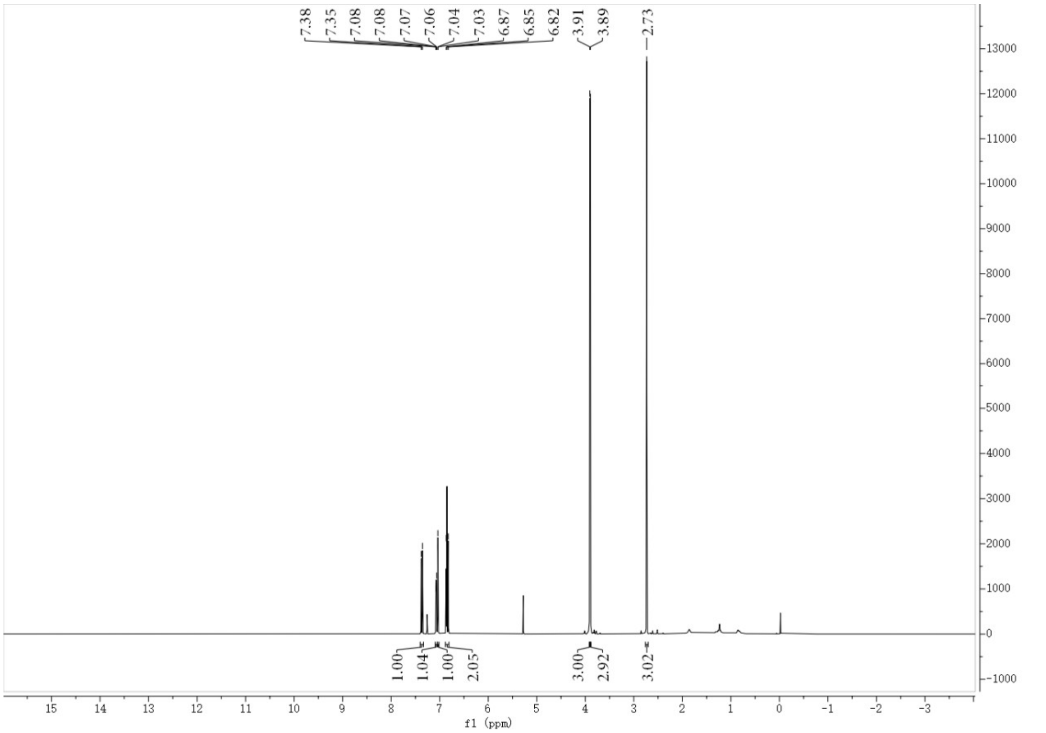

Fig. S118 The ^{1}H NMR spectrum of the target compound **6ad**

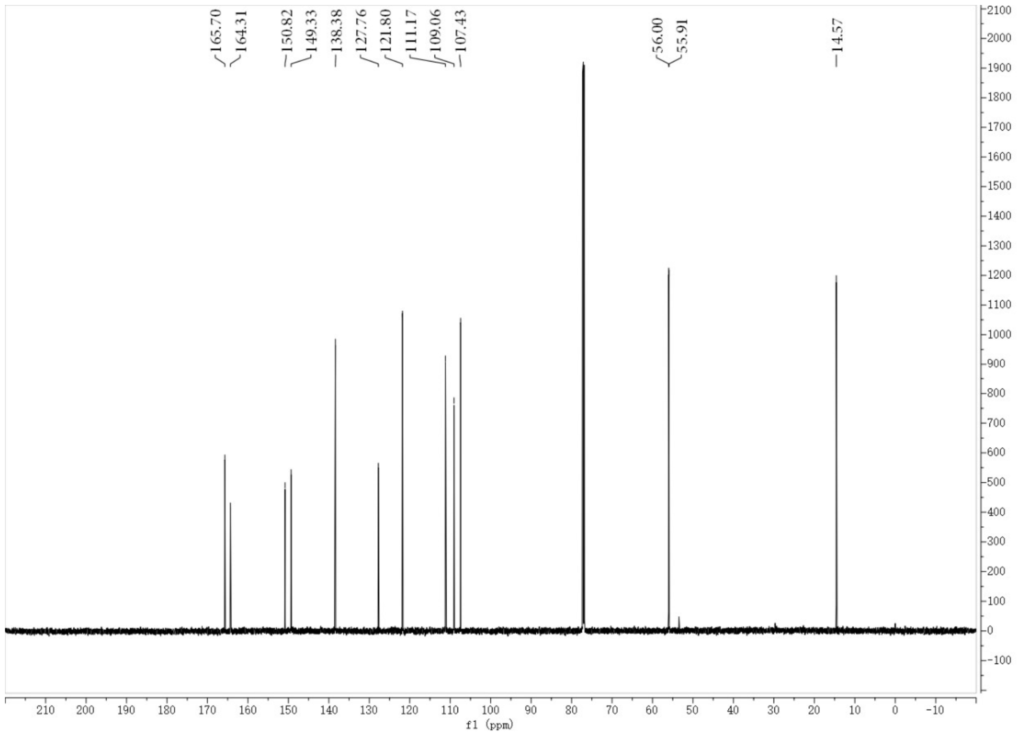

Fig. S119 The ^{13}C NMR spectrum of the target compound **6ad**

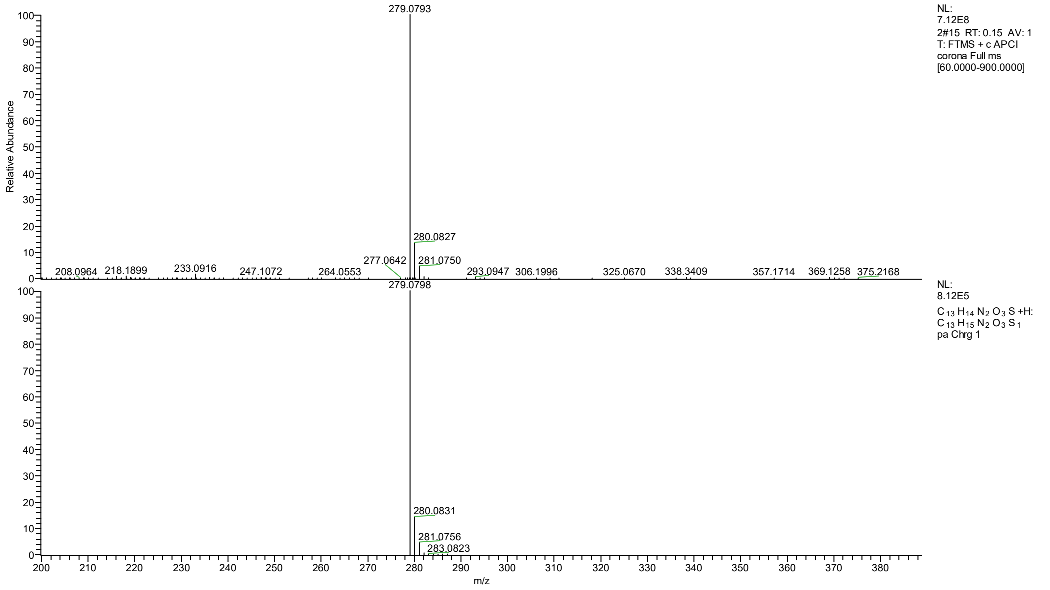

Fig. S120 The HRMS spectrum of the target compound **6ad**

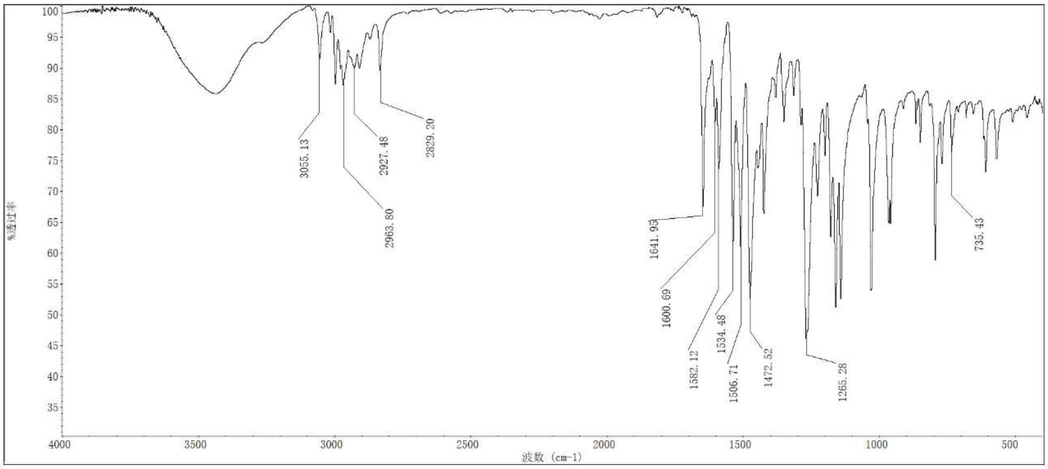

Fig. S121 The FT-IR spectrum of the target compound **6ae**

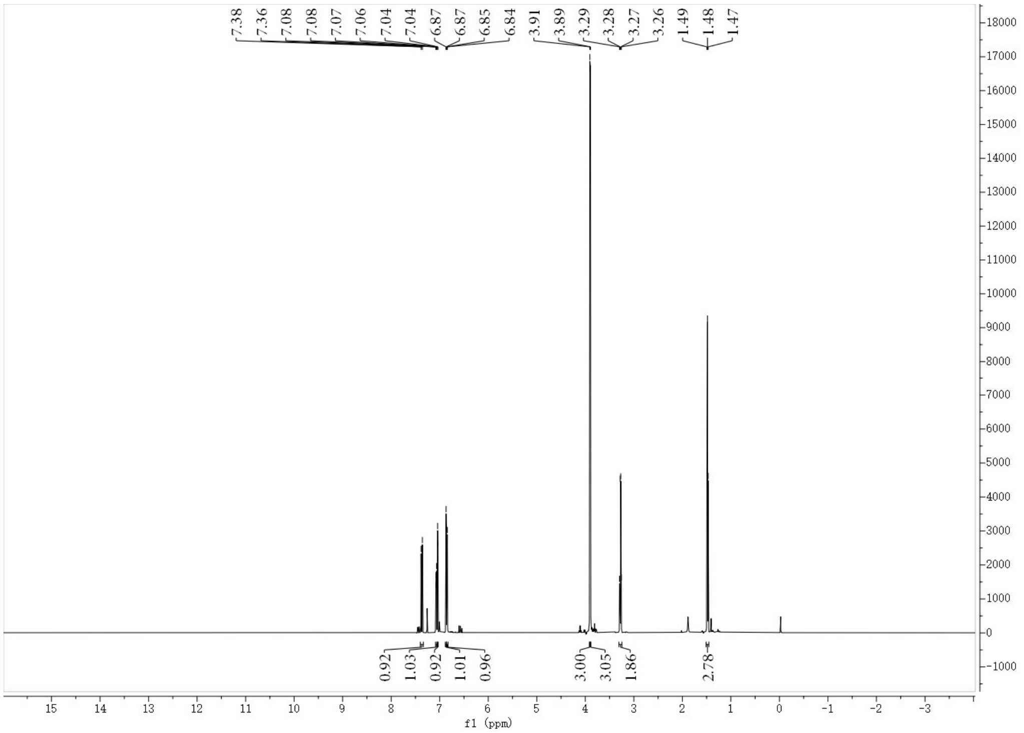

Fig. S122 The ^{1}H NMR spectrum of the target compound **6ae**

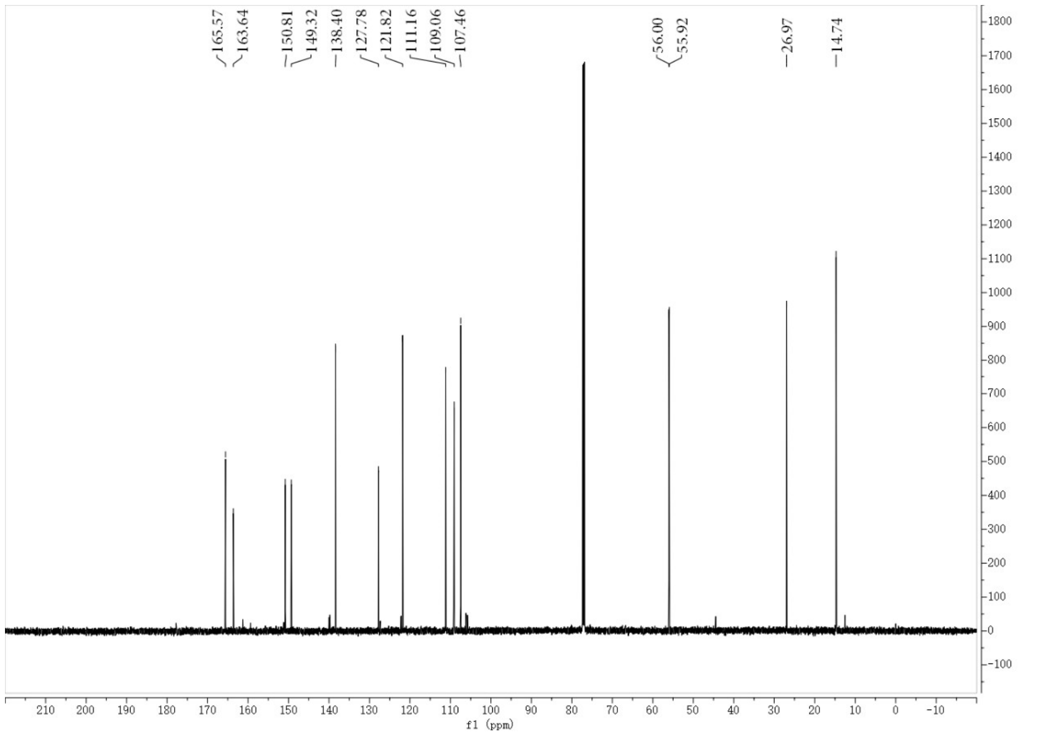

Fig. S123 The ^{13}C NMR spectrum of the target compound **6ae**

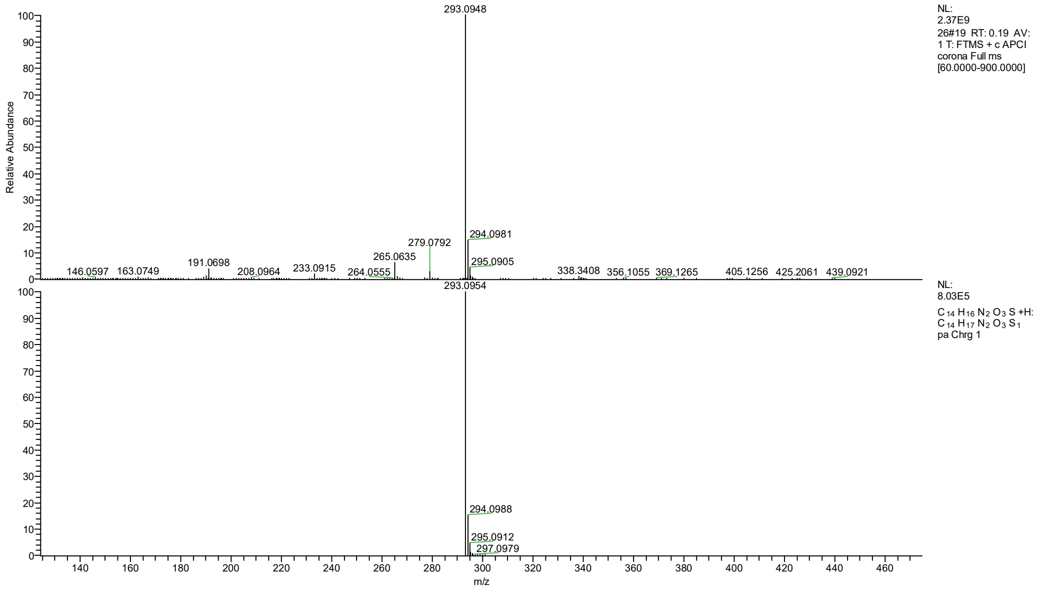

Fig. S124 The HRMS spectrum of the target compound **6ae**

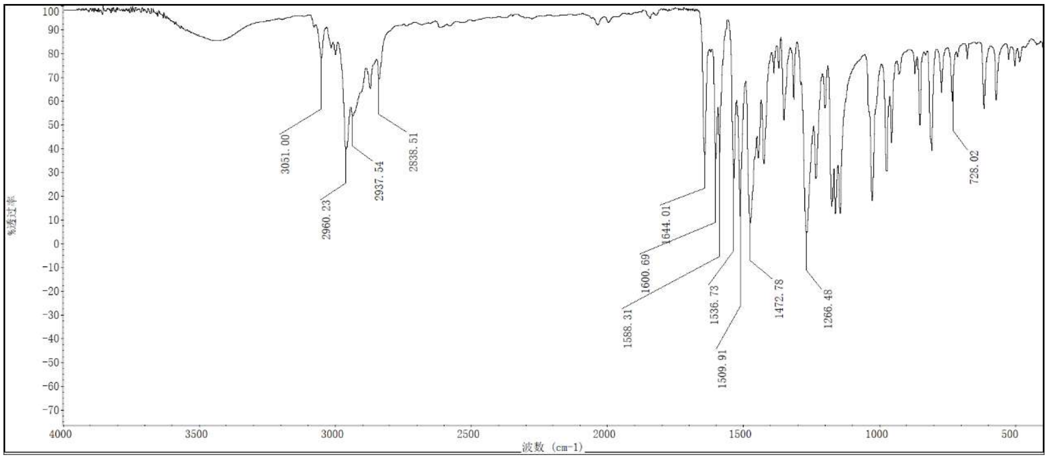

Fig. S125 The FT-IR spectrum of the target compound **6af**

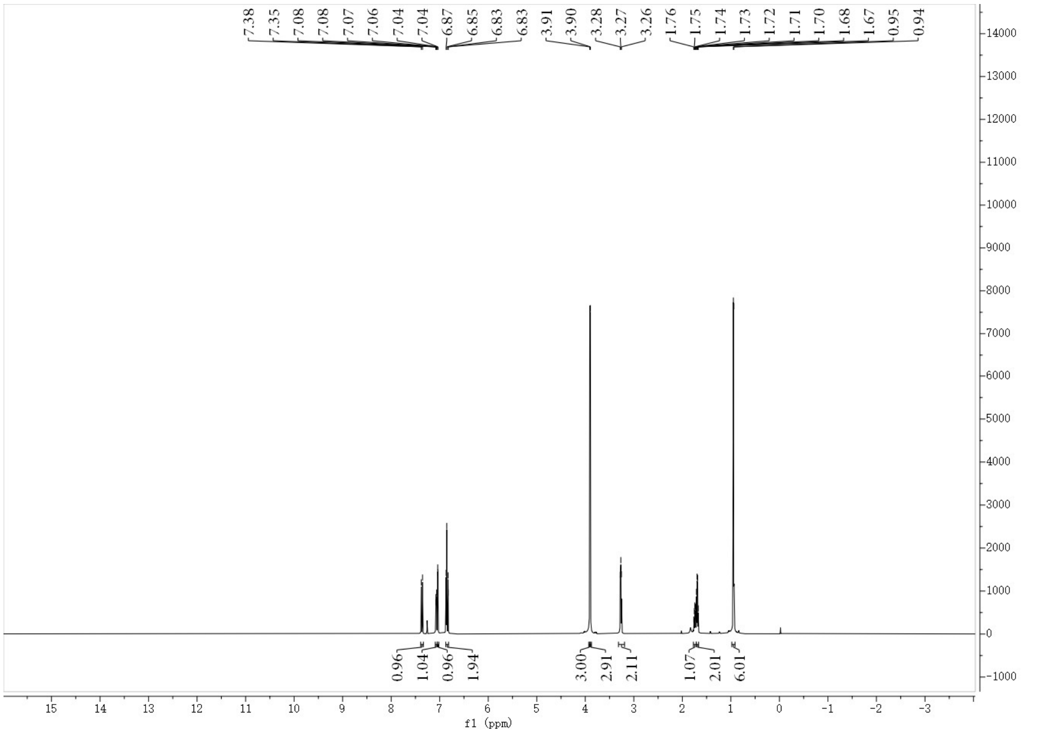

Fig. S126 The ^{1}H NMR spectrum of the target compound **6af**

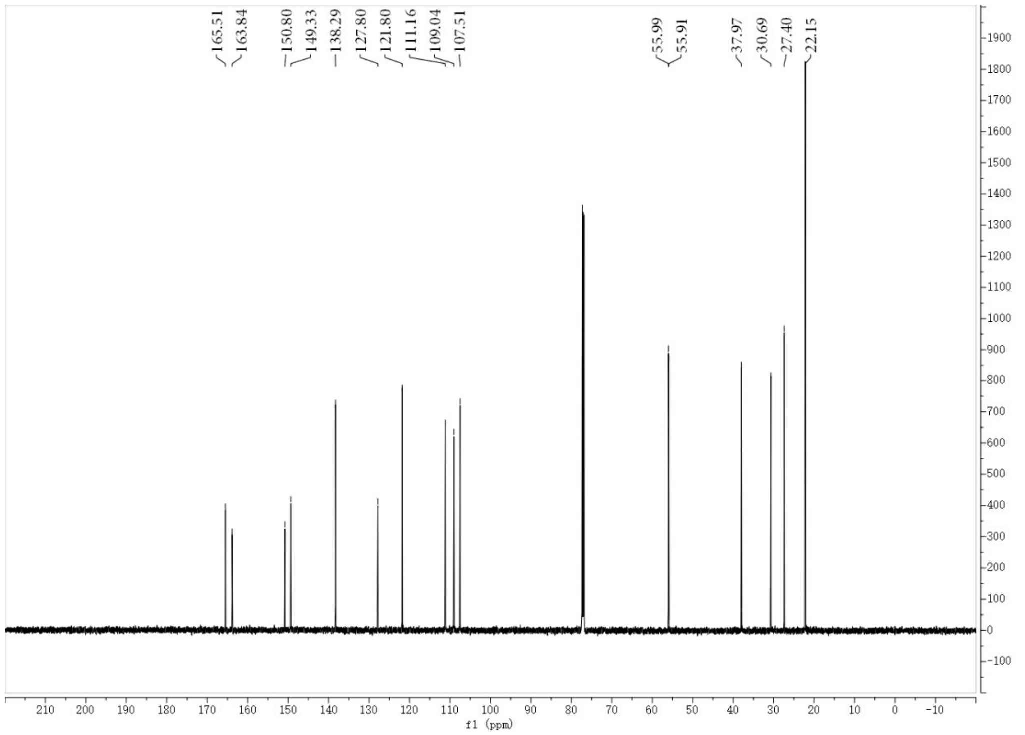

Fig. S127 The ^{13}C NMR spectrum of the target compound **6af**

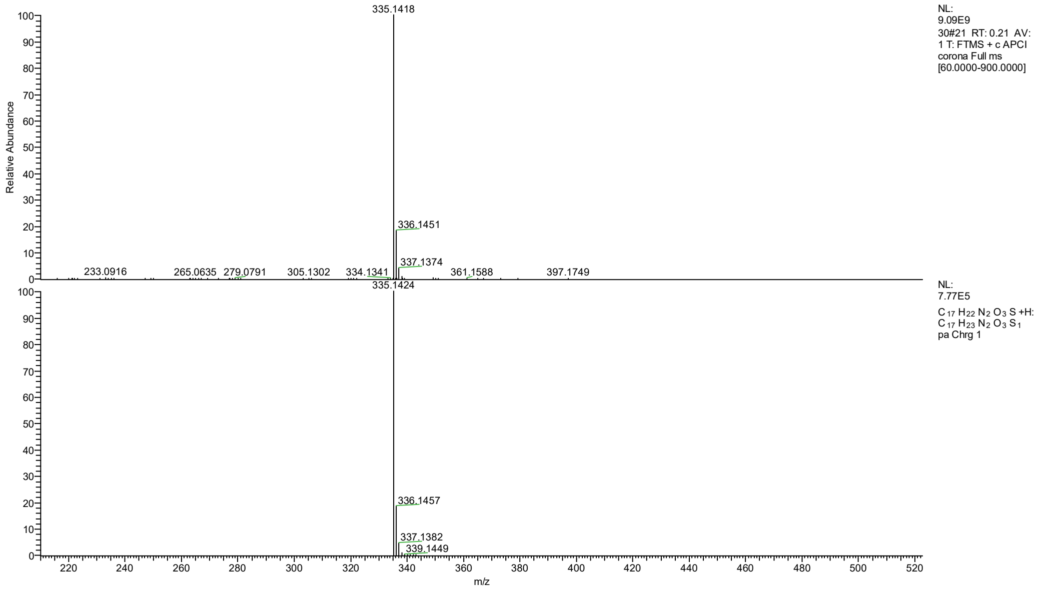

Fig. S128 The HRMS spectrum of the target compound **6af**
